# Geographic population structure of the honeybee microsporidian parasite *Vairimorpha* (*Nosema*) *ceranae* in the South West Indian Ocean

**DOI:** 10.1038/s41598-023-38905-0

**Published:** 2023-07-26

**Authors:** Nicolas Blot, Johanna Clémencet, Cyril Jourda, Pierre Lefeuvre, Natapot Warrit, Olivier Esnault, Hélène Delatte

**Affiliations:** 1 Université Clermont Auvergne, CNRS, “Laboratoire Microorganismes: Génome et Environnement”, Clermont-Ferrand, France; 2 Université de la Réunion, UMR Peuplements Végétaux et Bio-agresseurs en Milieu Tropical, 97410 Saint-Pierre, La Réunion France; 3 CIRAD, UMR Peuplements Végétaux et Bio-agresseurs en Milieu Tropical, 97410 Saint-Pierre, La Réunion France; 4 Center of Excellence in Entomology, Department of Biology, Faculty of Sciences, Chulalongkorn University, Bangkok, Thailand; 5 Groupement de Défense Sanitaire de la Réunion, La Plaine des Cafres, France; 6 CIRAD, UMR Peuplements Végétaux et Bio-agresseurs en Milieu Tropical, 101 Antananarivo, Madagascar

**Keywords:** Population genetics, Parasite evolution, Parasite genomics, Population genetics

## Abstract

The microsporidian *Vairimorpha (Nosema) ceranae* is one of the most common parasites of the honeybee. A single honeybee carries many parasites and therefore multiple alleles of *V. ceranae* genes that seem to be ubiquitous. As a consequence, nucleotide diversity analyses have not allowed discriminating genetic structure of parasite populations. We performed deep *loci*-targeted sequencing to monitor the haplotype frequencies of genome markers in isolates from discontinuous territories, namely the tropical islands of the South West Indian Ocean. The haplotype frequency distribution corroborated the suspected tetraploidy of the parasite. Most major haplotypes were ubiquitous in the area but with variable frequency. While oceanic isolates differed from European and Asian outgroups, parasite populations from distinct archipelagoes also differed in their haplotype distribution. Interestingly an original and very divergent Malagasy isolate was detected. The observed population structure allowed formulating hypotheses upon the natural history of *V. ceranae* in this oceanic area. We also discussed the usefulness of allelic distribution assessment, using multiple informative *loci* or genome-wide analyses, when parasite population is not clonal within a single host.

## Introduction

Microsporidia are unicellular and obligate intracellular parasites that constitute a highly divergent phylum whose taxonomic position beside or among *fungi* remains debated^1,2^. The adult Western honeybee (*Apis mellifera*) can be infected by several microsporidian species: the long known *Vairimorpha apis*^3^, the emergent *Vairimorpha ceranae*^4–6^ and the recently described *Vairimorpha neumani*^7^. These species were included in the genus *Nosema* until the recent phylogenetic redefinition of the *Vairimorpha* and *Nosema* genera^8^. These parasites are transmitted by fecal–oral route and infect the epithelial cells of the midgut of the adult honeybee^9,10^. *V. ceranae* is nowadays the most prevalent microsporidian parasite and one of the most common pathogens of the honeybee. It can induce physiological and behavioral changes in infected individuals (immune suppression, oxidative stress, alteration of metabolism, premature foraging, etc.) and eventually reduce their survival, with possible deleterious interactions with other stressors^5,10,11^. At the colony level, *V. ceranae* is associated with type C nosemosis that shows reduced clinical symptoms compared to *V. apis* infection such as lesser dysentery^12^. While *V. ceranae* has been associated with colony collapse, its role in the worldwide colony losses has been debated^11,13,14^.

*V. ceranae* spores have been detected in several *Apis* and *Bombus* species^15^, including the Asian honeybees *Apis dorsata, Apis florea* and *Apis cerana*. *V. ceranae* is thought to have experienced a recent host-jump from *A. cerana* to *A. mellifera*^4,16^. The timing and localization of such host-switch are unknown. The oldest infected *A. mellifera* conserved sample has been dated back to 1975 in California^17^. Once emerged in *A. mellifera*, the invasive *V. ceranae* would have experienced both a geographic expansion and an increase in its prevalence that could be still in progress^18,19^. As a consequence, *V. ceranae* has become the most prevalent and widespread microsporidian parasite of the honeybee and seems to have replaced *V. apis* in some area, possibly due to asymmetric competition^4,15,19–22^.

Phylogenetic analyses of polar tube protein (PTP) encoding genes showed a differentiation between *V. ceranae* populations infecting different *Apis* species^23,24^. Within the single *A. mellifera* host, sequence analyses have not allowed the discrimination of parasite variants or subpopulations, whatever the marker: the rDNA whose multiple copies in the genome may not be identical^25–28^, single-copy coding regions including fast-evolving virulence genes such as PTP genes^23,24,29,30^, and even *multilocus*^31–34^ and genome-wide analyses^35^. Only phylogenomics studies allowed the distinction of parasite clades, clustering isolates from *A. cerana* and *A. mellifera* colonies in Thailand, i.e. in the native area of the parasite, apart from other isolates worldwide, but without any phylogeographic pattern^36^. Genome comparison showed that most *V. ceranae* alleles were shared by honeybee colonies that were thousands of kilometers away but that specific polymorphic sites were found in Thai *A. cerana* and *A. mellifera* colonies^35,36^.

The absence of cultural approach allowing the isolation of clonal *V. ceranae* strains imposes the analysis of parasite populations, the smallest sampling unit being the population infecting a single honeybee, hereafter defined as an isolate. A single honeybee carries multiple alleles of the single-copy genes of *V. ceranae*. Most of the nucleotide variation occurs within a single host individual or within a single colony of *A. mellifera*, but not between colonies, neither from geographically distant locations, nor from different host lineages as it is the case for *V. apis*^24,30–34^. All studies have depicted high nucleotide polymorphism with an excess of infrequent haplotypes or singletons over neutral expectation that could be linked to a population expansion^24,32,33,35^. Several hypotheses have been proposed to explain this intra-hive and intra-individual variability: the systematic multiple infection with diverse *V. ceranae* populations, recurrent recombination events, a sexual reproduction, a clonal reproduction of polyploid genome^31,32,34,35^. *V. ceranae* is dikaryotic, with an unclear ploidy and heterozygosity levels between and within nuclei. Genomic data suggested at least a tetraploid set of chromatids^35^, but possibly diploidy only in discrete clusters of parasites^36^. The excess of intra-hive diversity and heterozygosity may alter analyses when only one or a few haplotypes among those present in an isolate are randomly sequenced, possibly resulting in the underestimation of the allelic diversity and in wrong topological phylogenetic assertions. Consequently, the observed absence of distinct *V. ceranae* subpopulations could reflect a homogenous parasite population infecting *A. mellifera* worldwide or conversely an incomplete assessment of the parasite diversity.

**Figure 1:**
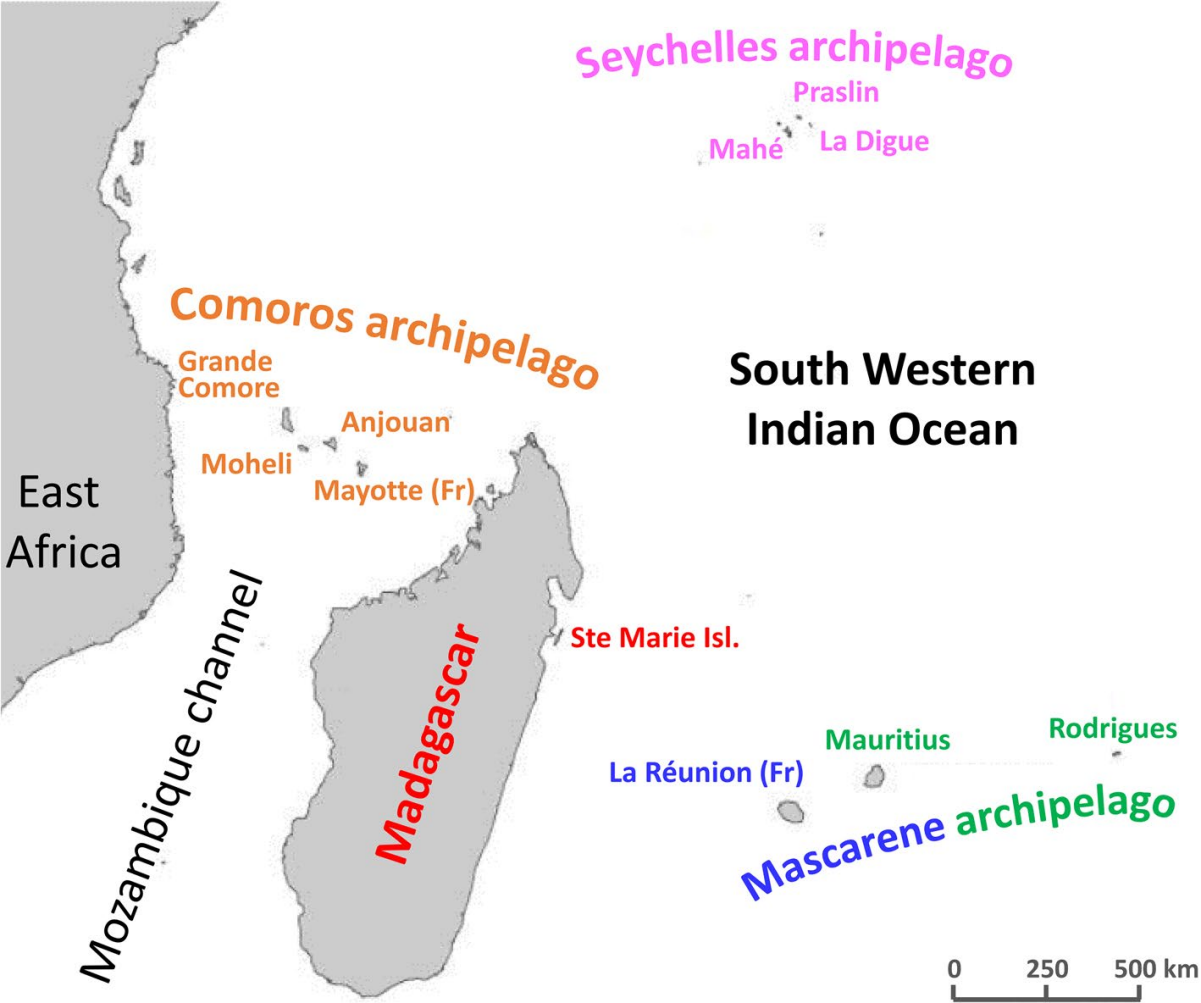
Map of the South West Indian Ocean (SWIO) showing the studied islands. Color-codes indicate the grouping of isolates throughout the forthcoming figures and tables.

To test if *V. ceranae* populations differed among discrete populations of *A. mellifera*, we performed genotyping by deep *loci*-targeted sequencing and we studied discontinuous territories, focusing on the tropical islands of the South West Indian Ocean (SWIO, Fig. 1). The SWIO constitutes a vulnerable hotspot of biodiversity with high rates of endemism^37^. It comprises the microcontinent of Madagascar and several archipelagoes: the Mascarenes Archipelago (La Réunion, Mauritius, and Rodrigues islands), the Seychelles Archipelago (Mahé, Praslin, La Digue main islands) and the Comoros Archipelago (Grande Comore, Mohéli, Anjouan and Mayotte islands). These islands differ by their environmental conditions and apicultural practices. On most islands, honeybee genotypes belong to the native A-lineage, with variable rates of hybridization with European lineages following the introduction of allochthonous queens or hives^38^. The prevalence of *V. ceranae* also varies. While one tenth of Malagasy colonies are carriers of the parasite, *V. ceranae* prevalence is very high in the small islands (79.6 to 100%)^39^. As outgroups, we aimed to include *A. mellifera* isolates from continental Africa and Europe, as well as isolates from other *Apis* host species in Thailand. Here, we assessed the haplotype frequencies by the high throughput sequencing of 42 coding and non-coding regions within *V. ceranae* genome in single honeybee isolates, avoiding the multicopy rDNA region. We demonstrated that the haplotype frequency distribution enabled the detection of parasite population structure. To our knowledge, it is the first population study depicting such a fine geographically structured populations of a microsporidian parasite.

## Results

*A. mellifera* individuals were collected in the SWIO and in continental Africa and screened for the presence of *Vairimorpha* spores (Table S1). Except two individuals from one colony in Senegal, no spores were detected in continental Africa. In contrast, spores were commonly found in honeybees in the SWIO, all identified as *V. ceranae*. Twenty-eight honeybee isolates from different hives were eventually selected in the SWIO, representing the different geographic areas (Table 1). The Mascarenes archipelago was divided into two areas: La Réunion on one side, Mauritius and Rodrigues on the other, corresponding to known contrasted apicultural regions^38^. The honeybees had been genotyped by Techer et al.^38^. Most were from the A lineage, including one from its sublineage Z. Six individuals were from C lineage and one from M lineage. Three European infected honeybees were chosen as a geographical outgroup. One isolate from *A. cerana* and one from *A. dorsata* were chosen as a host outgroup.

**Table 1:**
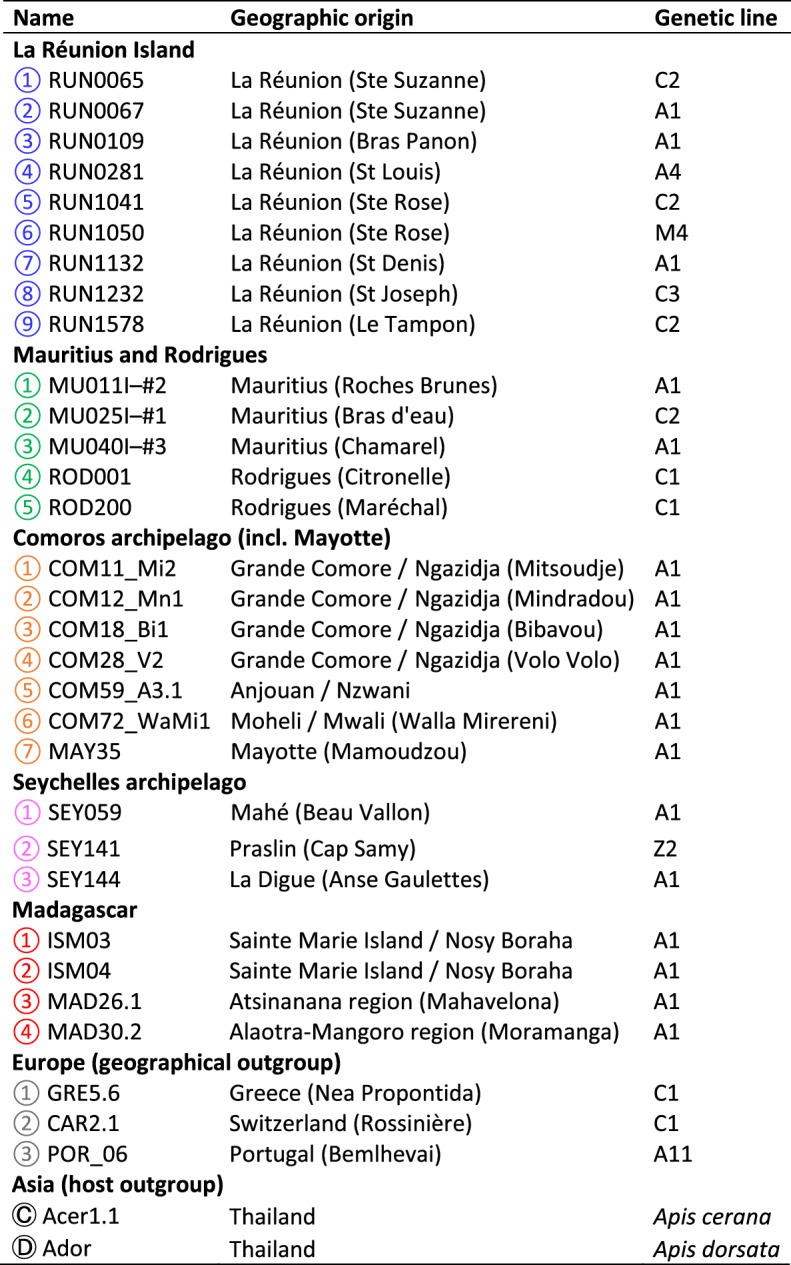
Honeybees studied in the present work.

All individuals were sampled from distinct colonies by Techer et al.^38^, except for MAD26.1 and MAD30.2 (courtesy of O. Esnault), GRE5.6 (F. Hatjina) and Thai isolates (N. Warrit).

### The major haplotypes represented 96% of the detected reads

Forty-two primer pairs were selected to amplify *V. ceranae* coding and non-coding regions, including microsatellites (Table S2). All markers were amplified by PCR but amplification was less efficient for several markers of the MAD26.1 isolate. The 052A amplification products showed two bands on electropherograms. Sequencing showed that these amplicons mapped on two *loci* on *V. ceranae* genome and the marker was kept for analysis. Following Illumina sequencing, the data provided by the subcontractor showed essentially mismatching paired reads, denoting wrong pairing. Thus, the two ends of each amplicon were treated separately as independent markers. The 0386AR marker (in EnP1B gene) was not exploitable due to large INDELS of a repeated motif^31^ that exceeded the reads length. As a consequence, 83 markers were considered and a total of 636 haplotypes were obtained following filtering (Table S3). One marker (001DR) as well as the clonal control did not show any variation in any isolate, validating the polymorphism in other markers.

**Figure 2:**
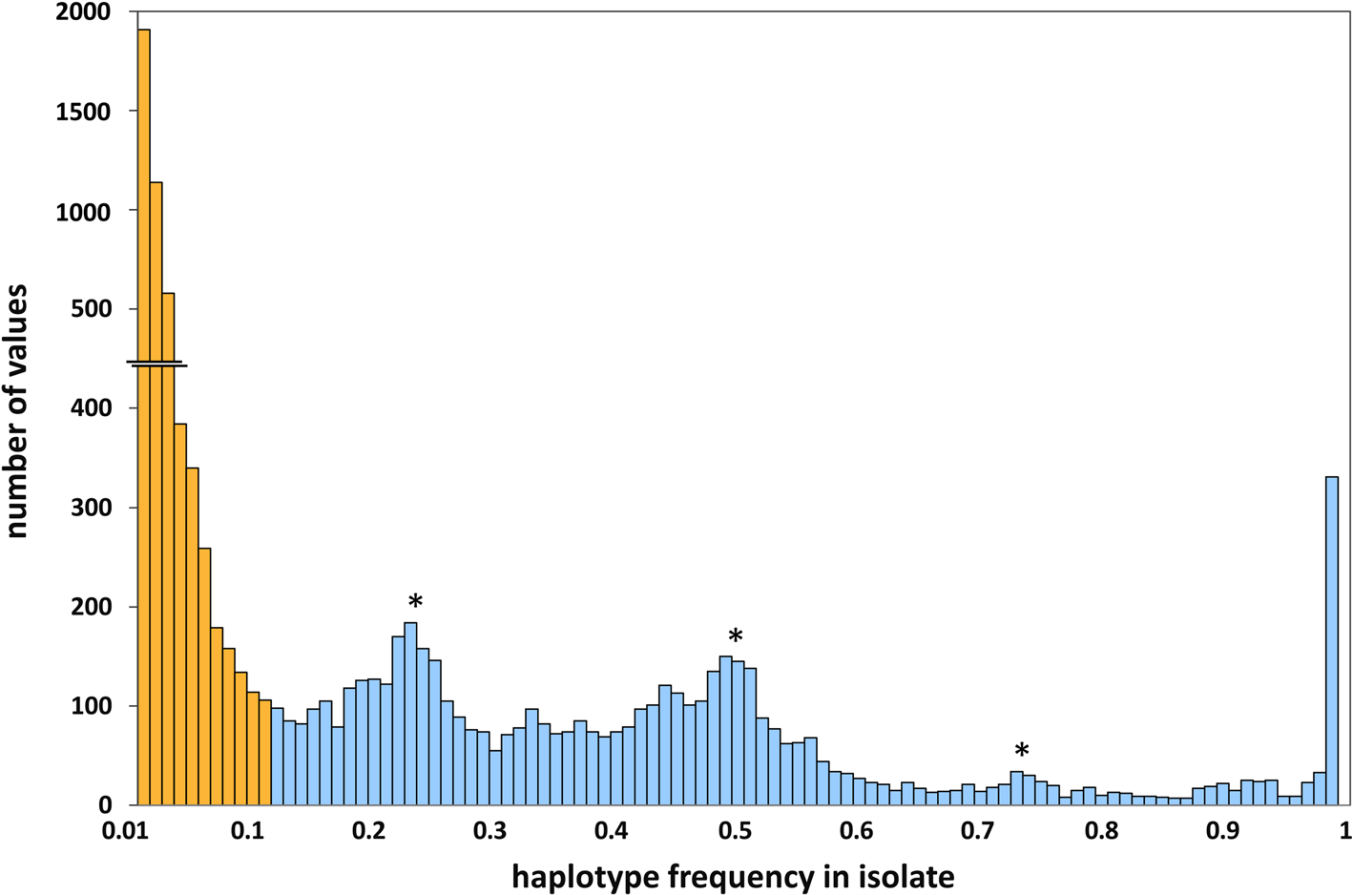
Distribution of haplotype frequencies, showing the number of values with a frequency from 0.01 to 1, with increasing intervals of 0,01 (n = 10,942 values). Haplotypes whose frequency was below and above 12% were classified as “rare” (yellow) or “major” (blue), respectively. Peaks at frequency close to 0.25, 0.5 and 0.75 are indicated by stars.

The distribution of frequencies of the 636 haplotypes in the 33 isolates (28 in the SWIO and 5 for outgroups) showed numerous values with low frequencies (Fig. 2). We considered haplotypes with a frequency higher than 0.12 in at least one isolate as “major haplotypes”, and other haplotype, with a frequency higher than 0.01 in one isolate but always lower than 0.12, as “rare haplotypes”. The high numbers of frequencies close to one represented highly dominant haplotypes. Interestingly, the frequency distribution also showed peaks at 0.25, 0.5 and 0.75.

**Figure 3:**
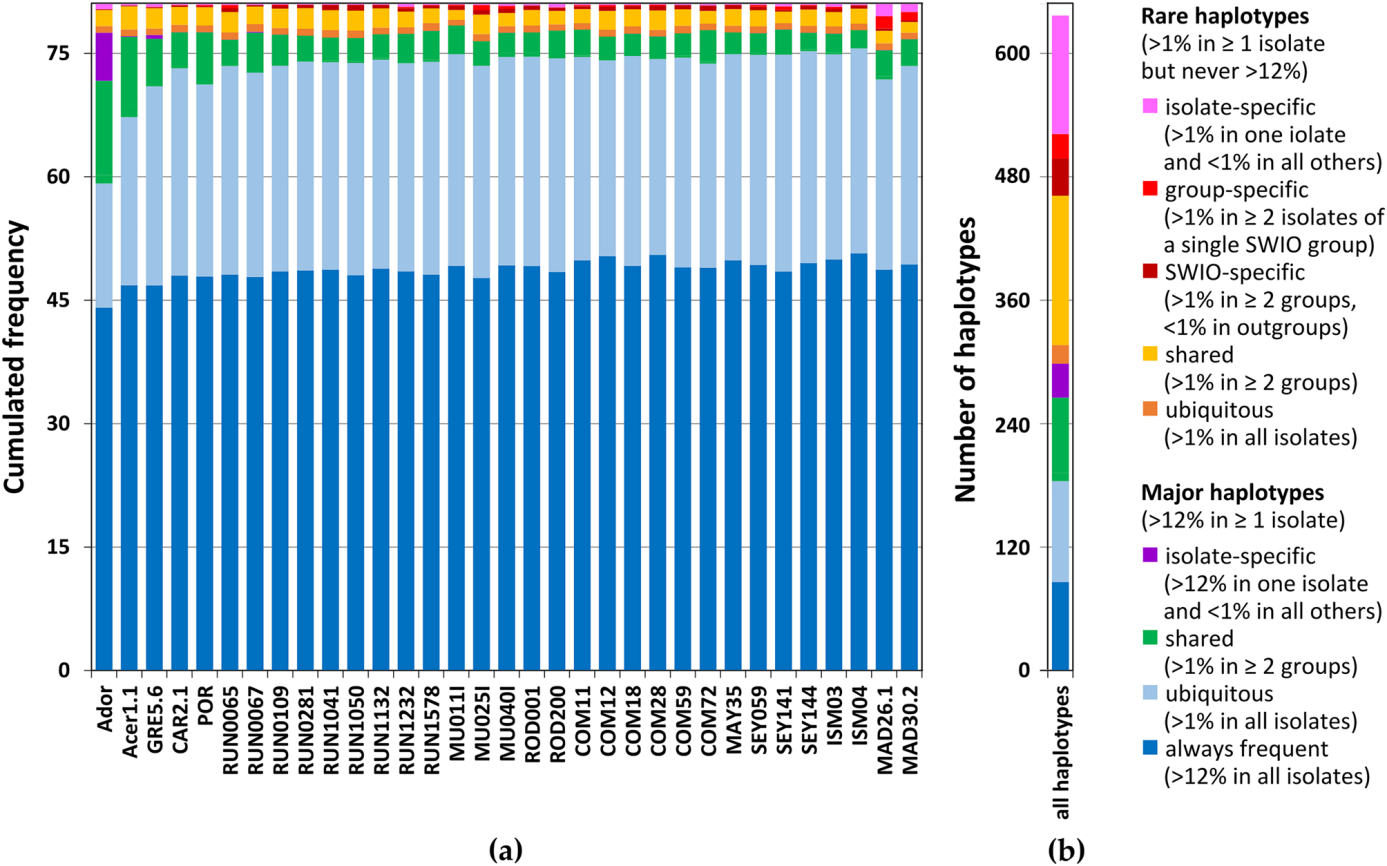
Cumulated frequencies of the 636 haplotypes from 83 markers in isolates (**a**) and their distribution in selected categories (**b**). Since markers were independently studied, the cumulative frequency that equaled 1 for each marker reached 83 over the dataset. A haplotype was considered present with a frequency higher than 1% in one isolate and ubiquitous with a frequency higher than 1% in all isolates. Haplotypes with a frequency higher than 12% in at least one isolate were considered as “major”. Haplotypes with a frequency higher than 1% in at least one isolate but never higher than 12% were considered as “rare”. If rare haplotypes were numerous (**b**), their cumulated frequency was low (**a**). Ubiquitous major haplotypes appear in blue, including those that are frequent in all isolates (dark blue). Shared major haplotypes, defined as present in at least two groups, appear in green. Isolate-specific major haplotypes are in purple. Ubiquitous rare haplotypes are in yellow. Shared rare haplotypes in orange. SWIO-specific, group-specific and isolate-specific rare haplotypes are in dark red, red and pink respectively. There was no SWIO-specific or group-specific major haplotype.

Three hundred and five haplotypes were considered as major haplotypes. They represented 95.7% of the reads. These included 86 haplotypes whose frequency where higher than 0.12 in all the isolates, representing 60.1% of the reads (Fig. 3). The 331 rare haplotypes represented only 4.3% of the reads. Twenty-four major haplotypes were specific to one isolate, always *A. dorsata*, denoting the distance of that isolate. Among rare haplotypes, 115 were specific to one isolate, including 100 that were specific to one honeybee in the SWIO, i.e. they were present in only one SWIO isolate. Nine out of the 28 SWIO isolates carried at least one specific haplotype. Interestingly the continental Malagasy honeybees carried most of the specific haplotypes: 32 for MAD26.1 and 31 for MAD30.2. MAD26.1 was the most divergent SWIO isolate in term of presence or absence of haplotypes. Thirty-five other major or rare haplotypes where present in only one archipelago (mainly Madagascar) and 33 in the SWIO only. There was no clear differentiation of isolates or groups of isolates when considering only the presence or absence of haplotypes (Figure S1). This changed once considering their frequency.

### The South West Indian Ocean archipelagoes differed in their haplotype distribution

The haplotypes distribution across the samples was illustrated in the Fig. S2 (see also Table S3). The parasite haplotype distribution differed in honeybee from the SWIO compared to outgroups for many markers (e.g. 001B, 001CR, 010DF, 011BR, 019AF, 185A) suggesting different parasite populations in the SWIO. Three isolates clearly stood out from the others: *A. dorsata*, *A. cerana* and the malagasy *A. mellifera* MAD26.1 isolate. The later differed from all other samples in several coding and non-coding markers (e.g. 001BR, 001DF, 003AR, 003B, 005BF, 020AF, 025AR). Other haplotypes showed distinct distribution for one island or one archipelago, e.g. 001CF and 005BR for the Seychelles, 013AF for Mauritius and Rodrigues, 014AR and 143AR for Mauritius only, 001EF and 019A for Reunion Island, 016B and 174AR for Madagascar (with the exception of the ISM04 isolate), 011CR for the Comoros. Some haplotypes were specific for geographic areas. The haplotypes 001CF03, 011AR09, 019AF07 and 143AF11 were present in the three honeybees from Seychelles but in none of the other isolates. The haplotypes 006AF08, 041AR06 and 143AR06 were only present in the three Mauritian honeybees. At last, a proximity between the Portuguese isolate and *A. ceranae* was observed for several markers (e.g. 001DF, 003A, 003B, 004AF, 004B, 005BF, 014AR, 019A, 052A).

**Figure 4:**
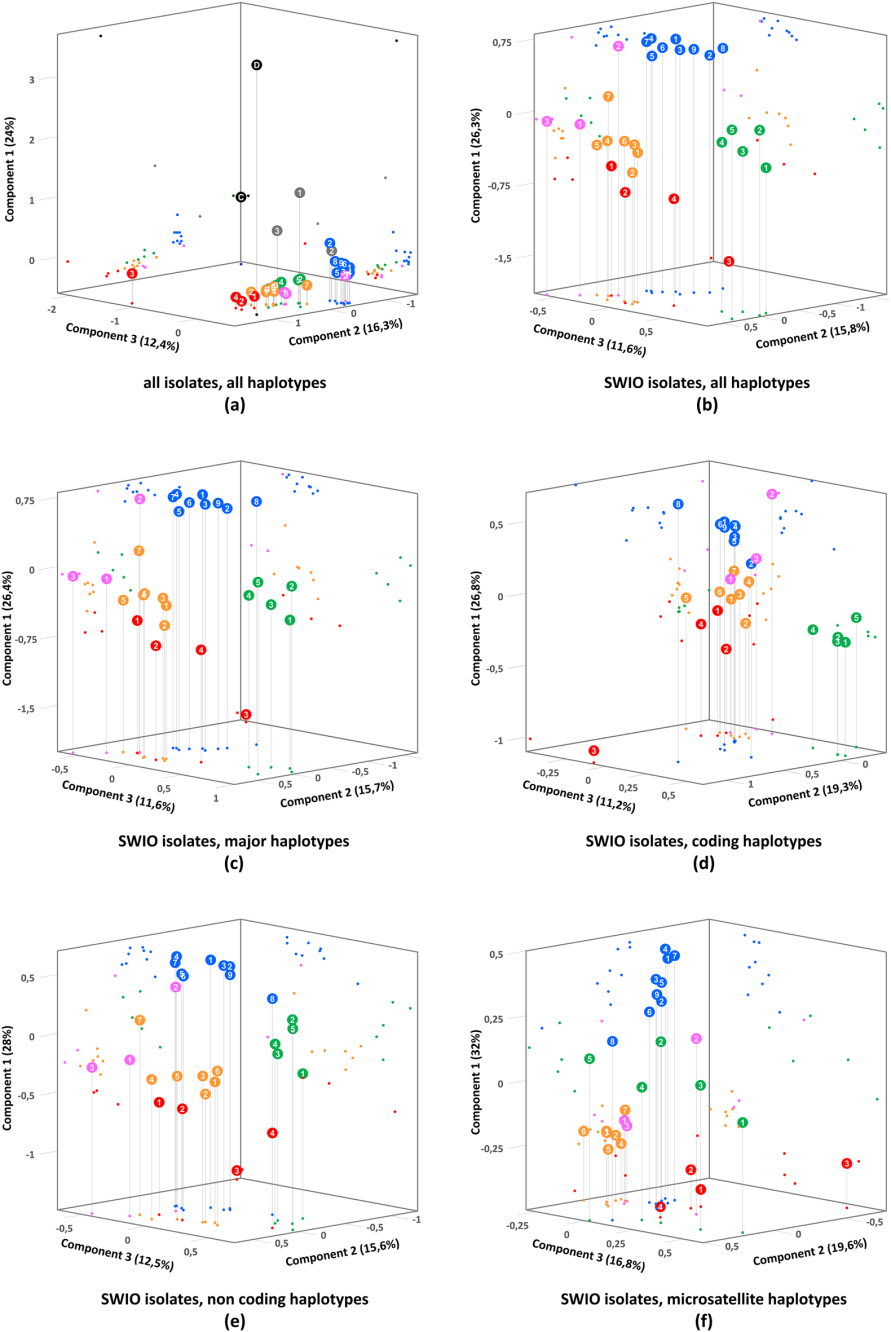
Principal component analyses (PCAs) of the *N. ceranae* haplotypes frequencies in *A. mellifera* isolates from the South West Indian Ocean (SWIO). Honeybees were sampled in La Réunion (blue), Mauritius and Rodrigues (green), the Comoros archipelago (orange), the Seychelles archipelago (pink) and Madagascar (red). Outgroup from Europe (grey) and from *A. ceranae* and *A. dorsata* in Thailand (black) were included. For details on the isolates, see Table 1. (**a**) PCA of all the 636 haplotypes, whatever their frequency, representing 83 coding and non-coding genetic markers from the 7 considered groups. The three first components represented 52.7% of the variance. (**b**) PCA (53.7% of the variance represented) of the 636 haplotypes from the SWIO isolates, i.e. without the outgroups. (**c**) PCA (53.7% of the variance) of the 305 major haplotypes from the SWIO isolates. (**d**) PCA (57.3% of the variance) of the 274 haplotypes, whatever their frequency, representing 43 coding markers from the SWIO isolates. (**e**) PCA (56.1% of the variance) of the 362 haplotypes, whatever their frequency, representing 29 non-coding markers from the SWIO isolates. (**f**) PCA (68.4% of the variance) of the 142 haplotypes, whatever their frequency, representing 11 microsatellites markers from the SWIO isolates.

Pairwise distances between isolates were calculated using the frequency data, showing lower distances within geographic groups and within SWIO isolates than between SWIO isolates and outgroups (data not shown). To visualize how the haplotype frequencies organized the parasite populations, principal component analyses (PCAs) were also performed. All parasite populations from honeybees collected in the SWIO, except MAD26.1, and the one from Switzerland formed a distinct population compared to the outgroups (Fig. 4a). As the outgroups affected the distribution, PCAs were performed on SWIO isolates only, showing geographic groups (Fig. 4b). All the Reunion isolates were grouped together with the Swiss isolate. The Mauritius and Rodrigues islands isolates also formed a distinct group. The other SWIO isolates, except three, formed a group that could itself be separated between two Seychellois isolates, the Comoros isolates and the Malagasy isolates. One Seychellois isolate was close to the Reunion/Swiss group and the Mayotte isolate was located midway. The MAD26 isolate was clearly a single separated isolate, demonstrating its divergence from the other parasite populations.

Although clonal control did not show any sequence variation, some rare haplotypes may still be originated from amplification or sequencing errors^36^. In order to rule out any bias, the same analyses were performed removing all rare haplotypes (Fig. 4c). An almost identical distribution was observed, demonstrating that the structure of the parasite population is poorly affected by rare haplotypes. Thus, at this scale of deep *multilocus* sequencing, the artifactual or technical variations of the haplotypes that may have occurred did not change the observed structure.

As coding and non-coding regions may be submitted to different evolutionary processes, PCAs were also performed separately on the coding markers (Fig. 4d), the non-coding markers (including microsatellites, Fig. 4e) and on microsatellites only (Fig. 4f). While the isolates distribution varied, groups were mostly maintained, showing that both coding and non-coding regions, including microsatellites only, allowed structuring populations.

NJ clustering showed that all Reunion island and one Seychellois isolates were grouped together, as well as the isolates from Mauritius and Rodrigues and the isolates from Madagascar (Figure S3). The two other Seychellois isolates and the ones from Comoros formed a last group. When including the outgroups, the Malagasy group was included in a Madagascar-Comoros-Seychelles group. The Swiss and the Reunion isolates were grouped together. The other outgroup isolates and MAD26.1 form a group with long branches.

Not considering the outgroups, MANOVA and ANOSIM showed uncorrected *p*-values that were below 0.05 between all the SWIO subgroups expect between Seychelles and Comoros and Seychelles and Madagascar for ANOSIM. However, differences were considered significant for Bonferroni-corrected *p*-values. Significant difference was observed between La Réunion and Mauritius/Rodrigues (*p* = 0.010), La Réunion and the Comoros archipelago (including Mayotte, *p* = 0.002), La Réunion and Madagascar (*p* = 0.030), Mauritius and the Comoros (*p* = 0.027). Removing the divergent MAD26 isolate did not change the results. Similar results were also obtained excluding the rare haplotypes or using the coding or non-coding markers only. A supplementary significant difference was observed between the Comoros and Madagascar using the coding regions or the microsatellites only. To get into more details, fixation indices were calculated for every marker with and without the divergent MAD26.1 isolate (Table S4). Except the invariable 001DR marker, all markers significantly differed within the whole populations (FST) and between groups (FSC) whereas significance varied with marker when comparing populations among groups (FCT). Altogether, these results confirmed the distribution depicted with PCAs and the geographical grouping.

## Discussion

The present study allowed the differentiation on *V. ceranae* isolates based on the high throughput sequencing of numerous genomic *loci*. It demonstrated that allele or haplotype frequency, whatever their nucleotide diversity, can be used to study *Vairimorpha* population structure. The data explained, at least partly, why the former cloning-sequencinq approaches could not resolve sub-populations. The major haplotypes represented 95.7% of the reads, including 86 haplotypes whose frequency where higher than 0.12 in all the isolates. As many major haplotypes are present in most or all individuals, the sequencing of a limited number of haplotypes in one isolate cannot resolve any differentiation. For instance, single cloning procedure would most probably lead to the sequencing of one major allele, and lead to wrong topology assertions. Moreover, as most alleles were ubiquitous, no topology could be established based on their sequences alone.

In accordance with the genome analysis of isolates performed by Peters et al.^36^, the data confirmed that discrimination of *V. ceranae* sub-populations can be performed through *multilocus* and genome-wide analyses*.* In the present work, the coverage was much higher (several thousand reads per marker and per isolate) than in previous whole genome analyses^35,36,40^, possibly allowing finest differentiation. Yet, as the frequency of major haplotype was sufficient to assess population structure (Fig. 4c), one may wonder if such high coverage was necessary. Estimations have been made to test which reduced datasets may still assess the population structure. A discriminating PCA distribution appeared with a coverage as low as 20 reads per marker and per isolate, and the removal of singletons (Fig. S4). Noteworthy, no structuration appeared using the 10 most polymorphic markers with a coverage of 200, supporting the need for genome-wide analysis. Sequence quality, e.g. performing high fidelity amplification, ought to be of importance as it influences SNP distribution, and consequently haplotype frequency, and was shown to erase evidences for recombination^36^. For instance, a comparative analysis of *V. ceranae* genomes suggested that some SNPs were study-specific and thus did not depict true population differentiation^36^. Fortunately, the observed population structure was maintained analyzing the major haplotypes only, i.e. removing all possibly biased haplotypes.

In conclusion, the frequency-based differentiation of isolates should rely on multiple informative *loci*, ideally genome-wide analyses, and a moderate sequencing coverage. Genotyping By Sequencing (GBS) could also be a method of choice as its strategy differs from the present one only in the unchosen *loci* that are linked to restriction sites.

The three *Vairimorpha* species infecting the honeybee have been detected in some African regions^7,41,42^ but no ascertained data are available concerning their prevalence. Here a very limited number of honeybees sampled in continental Africa carried *Vairimorpha* spores (Table S1) with only 2 individuals from the same colony in Senegal. The data suggested that the parasite had a low prevalence in continental Africa, while it was common in the SWIO, reaching 100% of the colonies in La Réunion^39^. More comprehensive data would be needed to test hypotheses that can be formulated upon these findings.

Honeybee subspecies in Africa mostly belong to the A-lineage, with other lineages present in the north of the continent^43,44^. If the genetic background of the A-lineage would confer resistance to *V. ceranae* and a limitation of its spread, its absence would also be in some of the SWIO islands, such as Madagascar and the Comoros, were only the A-lineage is present.

Climate and temperature could also influence *V. ceranae* prevalence and infection^45–47^. While the parasite is absent in some African areas^48,49^, its prevalence varies from few percent to more than 60% in other continental areas^50,51^. Interestingly, it reaches almost 100% in Hawaiian Islands in the pacific and in the Caribbean island of Dominica^52,53^. One hypothesis is that the parasite may benefit from a reduced seasonal variability due to tropical climates.

Some African areas could also have been preserved from the parasite due to limited introduction of allochtonous honeybees or apicultural materials, in contrast to regions such as North Africa, South Africa and the SWIO area. The exchange of colonies, together with contaminated hives materials, could have constituted the main route of introduction of *V. ceranae* in the SWIO islands. In the Mascarenes, exchanges of honeybees have been demonstrated through the presence of hybridization with European lineages^54^. The import of colonies has been prohibited in La Réunion since 1982 only. In Mauritius, regular and recent introductions of colonies from undocumented origin have occurred^38^. A recent introduction of Malagasy colonies to Grande Comore has also been suspected, reflecting exchanges within the SWIO. Thus, the parasite could have then spread both within and between the islands. The global allelic similarity, the accumulation of rare haplotypes and the few isolate-specific haplotypes support a recent demographic expansion^35^.

Haplotype frequencies showed a higher distance between SWIO and European isolates than within SWIO isolates and an even higher divergence with the Thai isolates in *A. dorsata* and *A. ceranae* (Fig. 4a). Such a distinction of *V. ceranae* genotypes was previously achieved through the genome-wide analysis of Single Nucleotide Polymorphism, showing a Thai population cluster in *A. mellifera* and *A. ceranae* that differed from other global population in *A. mellifera*^36^. The local reduced diversity with specific variation did not show any geographical patterns in Thailand but suggested that the Thai populations of parasites evolved independently from other global population. The present data showed that clustering of parasite populations could emerge in other part of the world than Southeastern Asia.

The main difference in allele content concerned *A. dorsata* and *A. cerana* hosts. Within *A. mellifera* isolates, most haplotypes were common to all isolates, with only few specific of one isolate or one geographic area (Fig. 4, S1 and S2). These was in good accordance with the genome analyses of Peters et al.^36^. The strong divergence between the SWIO and outgroups isolates suggested that the *V. ceranae* population in the SWIO may have a common origin and had enough time to diverge in allele frequency since its introduction. As the number of isolates in outgroups was restrained it was not possible to propose a geographic origin for this introduction. However, the proximity of the Swiss isolate with the population from la Reunion could be a hint towards a European origin (Fig. 4a).

The data also revealed, for the first time, fine local geographical patterns, despite the genetic proximity between SWIO isolates. The population structure within the SWIO appeared very robust as it was maintained with or without the outgroups, with or without the divergent Malagasy isolate, using all haplotypes or only the major ones, using all markers or only the coding or non-coding ones (Fig. 4). Samples were mostly grouped by island or Archipelago, with the exception of one Seychellois isolate, one strongly divergent Malagasy isolate and the Mayotte isolate. Each insular group seemed to possess allelic frequencies differentiated from another and the organization of the insular populations was coherent with the geography of the SWIO. Such a genetic clustering was observed for honeybee populations in the SWIO as well as other archipelagos^38^, suggesting a similar insular evolution of both hosts and parasites. The discrimination of insular groups suggested that (1) there has been enough time since the parasite introduction to install divergences in the allelic frequency between areas and that (2) if there were gene flows between areas or from outside the SWIO through new introductions, they have not been sufficient to erase these divergences.

Gene flow between islands could not be demonstrated by the present work, but the proximity of one Seychellois isolate and of the Mayotte isolate with those from la Réunion (the last two are French islands) could suggest such exchanges. However, the apparent absence of parasite substructure within islands or archipelagos may indicate that gene flow within these areas, i.e. through the exchange of parasite between colonies, had been sufficient to maintain allelic homogeneity. For instance, the closer proximity of isolates within La Réunion (Fig. 4) could be linked to a transfer of parasites facilitated by the small size of the island, the high colony density (> 20,000 colonies declared in 2020 for 2 512 km^2^^55^), the absence of overwintering and apicultural practices such as the transhumance of beehives following resource cycles. In contrast, in the Comoros Archipelago, where beekeeping is poorly developed and honeybee populations are structured by island^38^, *V. ceranae* isolates showed higher within-group distances.

The malagasy MAD26.1 isolate strongly differed from all other SWIO isolates (Fig. 4). Its divergence might even have been underestimated as PCR amplification seemed less efficient for many markers. It is possible that primers used were not optimal for this isolate and did not allow the amplification of all haplotypes. Thus it cannot be excluded that, as for *A. dorsata*, there would be specific allele content in this isolate and that the observed frequencies might have been misevaluated. The clear divergence of this Malagasy isolate from the other SWIO isolates, with a distance comparable to the ones of the *A. ceranae* and *A. dorsata* isolates, suggested that it could belong to a distinct cluster of *V. ceranae* parasites. The origin of such variation could reflect an independent introduction of *V. ceranae* in this area of Madagascar, that originated from a source distinct from the rest of the SWIO and that erased the original SWIO signature if *V. ceranae* was already present. It could also reflect an accumulation of divergence in allele frequency in an isolated area of this huge continental island where transhumance is hardly practiced.

The distribution of haplotype frequencies, with three peaks at 0.25, 0.5 and 0.75 (Fig. 2) was similar to the distribution previously observed in genome data by Pelin et al.^35^, confirming that *V. ceranae* may be polyploid, at least tetraploid. Another genome analysis of parasite isolated in *A. mellifera* and *A. ceranae* in Thailand, showed only a high peak at 0.5, suggesting diploidy^36^. In our data the Thai isolates from *A. ceranae* and *A. dorsata* did not show such a unique peak at 0.5 but rather three as mentioned above. The results are not incompatible. Tetraploidy does not necessitate all chromatids to differ, as shown for some markers, and local evolutionary processes may result in a reduction of allele diversity toward a *bi*-allelic content of genes. The available data thus lean towards a tetraploid organism with two heterozygous diploid nuclei, with possible loss of heterozygosity^36^.

The present work depicted a mixed similarity, with few major ubiquitous haplotypes, and diversity, with numerous but rare haplotypes. The allele content, in term of presence, appeared stable, as the major haplotypes were ubiquitous in *A. mellifera*. This clearly explained that the nucleotide diversity is present within a single colony or a single honeybee as well as between isolates, as observed in many previous studies^24,30–34^. Only fine frequency assessment allowed geographical clustering. On the other hand, the numerous rare haplotypes that represented only 4.3% of the reads, highlighted a variable but small portion of allele content.

The analysis of haplotypes sequences showed 485 polymorphic sites in the dataset, counting microsatellites as single sites (Table S5). As expected more transitions than transversions were observed, especially in coding regions (Table S6). In coding region, there were lots of non-synonymous polymorphic sites but most of them were observed in rare haplotypes, supporting the difference between major and rare haplotypes. Taking frequencies into consideration there were more synonymous than non-synonymous polymorphic sites. Very few induced a stop codon or a reading frame shift, almost all in rare haplotypes from *A. dorsata*. To explain this variability, several authors suggested a multiple infection with different strains on *V. ceranae*^24,27,32,34^. This implies that repeated infection events would be systematical. The ubiquity and frequency distribution (Fig. 2) of the major haplotypes could also be explained by a single infection with a tetraploid heterozygous parasite that experiences recombination and mutation events. Peters et al.^36^ demonstrated that biased polymorphic sites may also be generated during the procedure, especially through low polymerase proofreading activity and sequencing quality. Such mistakes could not be excluded in the present data, as suggested by the introduction of stop codons or reading frame shifts in a few coding haplotypes. However, rare haplotypes could not be only originated from mistakes as stringent filters were applied to avoid them as much as possible and as a clonal plasmid control was used to assess the fidelity of the procedure.

The origin of this variation remains unclear. In an evolutionary perspective, authors have debated upon the spread of a sexual population of *V. ceranae*, as suggested by linkage disequilibrium and recombination estimations^27,32–34^, or a clonal mode of evolution linked to polyploidy^31,36^. Recurrent recombination events, e.g. between heterozygous chromatids, has been suspected to be abundant^24,27,32,33^ and could generate a flow of diversity. However, such abundance has been questioned^31,56^, up to the elimination of any evidence of recombination using high quality data^36^. In contrast, the ubiquitous presence of a limited number of major haplotypes could be explained by the spread of a clonal tetraploid organism^36^.

Microsporidia are thought to evolve rapidly at the sequence level^57^. The similar proportion of rare haplotypes towards isolates could involve the continuous apparition of diversity through mutation. The numerous non-synonymous polymorphic sites would thus reflect a high mutation rate rather than diversifying selection. Negative selection pressure would eliminate deleterious alleles and keep the emerging haplotype content at low frequency compared to the major ones. Noteworthy, as recalled by Maside et al.^33^, most mutations present in an expanding population have a recent origin and, therefore, are rare. The rare haplotypes could also reflect a spreading population.

As the major coding haplotypes are present everywhere, including outside the SWIO, and carry mostly non-synonymous polymorphic sites, they ought to be under selection pressure. Such strong selection would also explain the single haplotype observed for 001DR. However, if the relative pressure between alleles is similar, their variation in frequency should be under no or neutral selection, i.e. close to equilibrium.

As a conclusion, the allele diversity of *V. ceranae* is mostly present within a single honeybee host and can be explained by the maintenance of major haplotypes and the continuous generation of rare alleles mostly subjected to negative selection pressure. The frequency of haplotypes—and the most frequent are sufficient—can differentiate parasite subpopulations. Island populations offered a good opportunity to study parasite population structures, showing clustering linked to the archipelagos geography. At last, the data strongly suggested that *V. ceranae* development in the honeybee can be explained by a single infection with a tetraploid and possibly clonal parasite. Although population structure could be resolved in the SWIO, the ubiquitous major alleles confirmed the close proximity of the *V. ceranae* populations worldwide in *A. mellifera*. Thus similar parasites seem globally present although their natural history may be highlighted through allele frequency.

## Methods

### Sampling and screening for infected honeybees

*A. mellifera* individuals were collected in 2011–2014 in the SWIO area. Subsequently, samples were collected in continental Africa and Europe to form outgroups, but the latter sampling was not designed to assess the prevalence of *V. ceranae* in these regions (Table S1). Individuals of *A. florea, A. dorsata* and *A. ceranae* were also collected in Thailand. Honeybees were kept at -20 °C dry or in 90% ethanol. For samples frozen in ethanol, single abdomens were removed using flame-sterilized forceps, dried for 30 min at 37 °C and ground in 200 µL ATL buffer (QIAGEN) using a sterile microtube pestel. For fresh samples, single abdomens were ground in 100 µL TE buffer and the mixtures were complemented with 100 µL ATL buffer after the screening of spores.

The presence of microsporidian parasites was first screened by the observation of spores under a phase contrast microscope (× 400). In the SWIO, 18.6% (n = 349) of the honeybees were parasitized but the presence of spores was low in Madagascar compared to the rest of the SWIO area (0.9 vs 51.6%; Table S1). Similarly, only 0.6% (n = 363) of honeybees carried *Vairimorpha* spores in continental Africa, with two positive bees from a same colony in Senegal. In Europe, taken as a geographical outgroup, 4.8% (n = 104) of bees were infected. Spores were detected in one *A. dorsata* and two *A. cerana* individuals.

Positive samples were boiled 10 min to lyse the resistant parasite spores and incubated in the presence of 2 mg/mL proteinase K for 2 h at 56 °C. The resulting liquid was transferred into a new tube to get rid of cuticle pieces and boiled again for 10 min. DNA was extracted using the QIAamp DNA Mini Kit (QIAGEN), with two final elutions with 100 µL EB. The presence of *V. ceranae* was verified by PCR using the primers MICROmid-F 5′-GGAGTGGATTGTGCGGCTT-3′ and MICROCE-R 5′-TGCTAATGGTTCTCCAAC-AGC-3′^9^ using Type-it Microsatellite PCR Kit (Qiagen). The absence of *Vairimorpha apis* was checked using the Na_D1 5′-GCATGTCTTTGACGTACTATGTAC-3′ and Na_R1 5′-CGTTTAAAATGTGAAACAACTATG-3′ primers^58^.

Twenty-eight *A. mellifera* isolates from different hives were selected in the SWIO, together with three European isolates, one *A. cerana* and one *A. dorsata* isolates (Table 1). As the further amplifications failed for the infected isolates from Senegal, there was no continental African outgroup in the study.

### Markers screening and primer design

Genetic markers were selected using the 454 pyrosequencing data of Cornman et al.^40^ and the Illumina sequencing data of Pelin et al.^35^. The SRA files were extracted and mapped on the *V. ceranae* PA08 1199 genome^35^ using the BWA-MEM v0.7.12 algorithm^59^. Mappings were visualized using Tablet software^60^. All targeted regions were checked visually to choose markers with polymorphic sites and conserved adjacent regions for the design of primers. Three types of markers were designed (Table S2). For markers in coding regions, virulence genes encoding Polar Tube Proteins (PTP1, PTP2, PTP3), Spore Wall Proteins (SWP30) or Endospore Proteins (EnPB1) were selected as they are known to show variability between or within microsporidian species^23,29,31,61^. Some of them showed INDELS within the mapped sequences. Genes used for fungal or microsporidian phylogenetic analyses (EF-2, RPB1, α-tubulin, hexokinase) were also chosen^62–64^. Other genes showing nucleotide polymorphism in genome data were visually selected. Putative microsatellite sequences, with tandem repeats of di- to penta-nucleotides, were screened in *V. ceranae* genome using MsatCommander^65^. Sites at the ends of scaffolds and inside coding regions were sorted out. Sites with adjacent sequences represented in several scaffolds were also removed to avoid duplicated *loci*. Eleven microsatellites were eventually selected. At last, eight non-coding sites were selected by screening for intergenic regions showing polymorphism in genome data.

Primers were designed using the primer3 tool and manually modified to be localized outside the variable regions in the mapped genome data. Only primers showing low self-complementation and high specificity were selected. Their theoretical specificity was checked on *V. ceranae* and *A. mellifera* genomes, and by BLAST search on the *nr* and microbial genome databases. For each primer couple, the specificity of the PCR was verified on agarose gel electrophoresis on both infected and uninfected samples. Forty-two primer pairs were eventually selected (Table S2).

### Amplification and sequencing

For amplification, all primers were carrying a 4-nt tag at their 5′ end: GTCA (tag0), CGAT (tag5) or TGTG (tag6). Tags varied with samples but the same tag was carried by both the forward and reverse primers for a same sample. PCR amplification were carried out using 0.5 pmol of each primer, 5–220 ng of total DNA and 7.5 µL of 2 × Type-it Master Mix (Type-it Microsatellite PCR Kit, Qiagen) in a total volume of 15 µL. The PCR program consisted in an initial step at 94 °C for 15 min, and 35 cycles at 94 °C for 30 s, 58 °C for 30 s and 72 °C for 30 s, and a final step at 72 °C for 7 min. Negative controls without DNA were included in each reaction set and a clonal control amplification was performed using pGEM®-T (PROMEGA) plasmid template to amplify a fragment of the ampicillin resistance *bla* gene using the TEM-F 5′-CCGCATACACTATTCTCAGAATG-3′ and TEM-R 5′-ACGCTCACCGGCTCCAGATT-3′ primers derived from Monstein et al.^66^. PCR reactions were performed in triplicate and their end products were pooled together. Amplification products were checked by electrophoresis on both agarose gel and QIAxcel Advanced automate (QIAGEN). PCR products were quantified on the QIAxcel phoregrams relative to standards of known concentrations. For each isolate, all 42 amplicons eventually carried the same tag at both ends and an equimolar mixture of the amplification products was prepared To prepare libraries, 0.9 µg of the mixtures form three isolates, that carried three different tags, were pooled together with 1.7 ng of the clonal PCR control (Table S7). Libraries were multiplexed and sequenced on Illumina HiSeq2000 system using 2 × 250 bp paired end protocol by Genewiz (Cambridge, MA, USA).

### Sequence analyses

Sequencing resulted in 91,655,818 paired reads. Sequence quality was checked using the FastQC tool^67^. Reads with uncalled bases were removed using the vsearch tool^68^. Remaining reads were assigned to each sample using the FASTX Barcode Splitter tool from the FASTX-Toolkit^69^. The first 5 bases of demultiplexed sequences were trimmed using the fastx_trimmer tool to remove barcode sequences. Each library had over 80% of reads at Q30 after de-multiplexing, with few biases between libraries.

The obtained data clearly exhibited 5′ and 3′ tags that were randomly spread instead of being identical for paired reads, showing that the pairing of reads was unsuccessful. We thus renounced joining paired ends and worked with single reads, i.e. the reads corresponding to each extremity of the amplicons had to be considered as separate genetic markers. In order to improve the quality of data, the last 50/75 nt of demultiplexed reads were trimmed and the resulting sequences were mapped on the marker sequences (genome version of Pelin et al.^35^) using the BWA-MEM algorithm with a minimum 100 bp-long alignment option^59^. 166,993,099 demultiplexed cleaned single reads were mapped on the 42 markers. For alignments, complex sequences with INDEL were manually realigned for a maximum parsimony. Noteworthy, there was a good correlation between the coverage of both ends of markers. A list of haplotypes present in the whole dataset was created, keeping only those that covered more than 1% of the reads of the corresponding marker in at least one honeybee isolate. Using these conditions, the control reads for the clonal *bla* gene exhibited only a single haplotype for each side of the marker. These resulted in 98,226,822 exploited reads. Except for a very limited number of cases, several thousands of reads were obtained per marker and per isolate, with an average of 36,694 reads per marker and per isolate and more than 1200 reads in 99% of cases. Haplotypes frequencies were calculated per marker and per sample.

### Statistical analyses

Analyses of molecular variance (AMOVA) were performed using Arlequin software^70^ using the conventional F-statistics (haplotype frequencies) with 10,000 permutations. Principal component analyses (PCA), Neighbor-Joining (NJ) clustering and multivariate analysis of variance (MANOVA) and analysis of similarities (ANOSIM) were performed using PAST software^71^. Groups of isolates were defined earlier (Table 1). PCAs were performed separately on the 43 coding markers, the 40 non-coding marker (including microsatellites) and the 11 microsatellite markers. They were performed on variance–covariance matrix, either disregarding groups or between groups with eigenanalysis carried out on the group means. NJ clustering was performed using Euclidean similarity indices matrix and 10,000 bootstraps. Non-parametric MANOVA and ANOSIM were performed using Euclidean similarity index and 1,000,000 permutations.

## Supplementary Information

Supplementary Information.

## Data Availability

Demultiplexed SRA data are available in NCBI (BioProject ID PRJNA796498).
