# Supplementary material for: Geographic population structure of the honeybee microsporidian parasite Vairimorpha (Nosema) ceranae in the South West Indian Ocean
Source: Sci Rep. 2023 Jul 26;13:12122. doi: 10.1038/s41598-023-38905-0 (PMC10372035; doi:10.1038/s41598-023-38905-0)
Supplement: Supplementary file 1 — Supplementary Information. [file 41598_2023_38905_MOESM1_ESM.pdf]

Supplementary Information

Geographic population structure of the honeybee microsporidian parasite *Vairimorpha* (*Nosema*) *ceranae* in the South West Indian Ocean

N. Blot*, J. Clémencet, C. Jourda, P. Lefeuvre, N. Warrit, O. Esnault and H. Delatte

* Correspondence: nicolas.blot@uca.fr

Supplementary Information

The document includes the following supporting materials, in their order of citation in the corresponding article:

Table S1: Screening for *Vairimorpha* parasites in honeybees .... p 2
Table S2: Markers and primers used in the study .... p 3
Table S3: Frequency of 636 haplotypes of 83 *N. ceranae* markers in honeybees .... p 4-13
Figure S1: Presence of haplotype in isolates .... p 14
Figure S2: Haplotypes distribution in the isolates .... p 15-56
Figure S3: Neighbor-Joining clustering of isolates .... p 57
Table S4: AMOVA analyses of 83 markers in the 28 SWIO isolates .... p 58
Figure S4: Effect of the reduction of sequencing coverage on isolates distribution .... p 59
Table S5: List of nucleotide changes in haplotypes .... p 60-67
Table S6: Types of polymorphic sites in isolates .... p 68
Table S7: Tags and libraries .... p 69

Table S1. Screening for Vairimorpha parasites in honeybees in the South Western Indian Ocean (SWIO), in continental Africa and Europe (geographic outgroups) and in Asia (host outgroup). Parasites were screened by the microscopic observation of spores and by PCR. Honeybees were *A. mellifera* species except when indicated in Thailand. n: total number of screened bees.

**Country (number of colonies)**	**% infected bees**	
South Western Indian Ocean		
La Réunion (10)	75	(n=20)
Mauritius		
Mauritius (4)	75	(n=24)
Rodrigues (7)	50	(n=8)
Comoros		
Grande Comore / Ngazidja (8)	76,9	(n=13)
Anjouan / Nzwani (6)	25	(n=8)
Moheli / Mwali (3)	37,5	(n=8)
Mayotte (8)	14,3	(n=14)
Seychelles		
Mahé (6)	14,3	(n=7)
Praslin (5)	20	(n=5)
La Digue (2)	71,4	(n=7)
Madagascar		
Ste Marie (2)	25	(n=8)
Continental (26)	0,9	(n=227)
Continental Africa		
Burkina Faso (1)	0	(n=8)
Central Africa (6)	0	(n=34)
Chad (1)	0	(n=6)
Gabon (nd)	0	(n=20)
Kenya (nd)	0	(n=12)
Mozambique (1)	0	(n=1)
Senegal (2)	5,3	(n=38)
South Africa (2)	0	(n=22)
Tanzania (11)	0	(n=197)
Uganda (nd)	0	(n=20)
Zimbabwe (1)	0	(n=5)
Europe		
France (5)	8,3	(n=12)
Spain (2)	0	(n=3)
Portugal (3)	15,4	(n=13)
Greece (6)	2,3	(n=76)
**Asia**		
Laos (2)	0	(n=8)
Thailand		
Apis florea (5)	0	(n=60)
Apis dorsata (3)	2	(n=50)
Apis cerana (9)	2,4	(n=83)

Table S2. Markers and primers used in the study. Two markers per amplification were considered due to unpaired sequence reads.

Marker	Type [1]	Genomic context of locus		Primer 5'→3'	Amplicon
		Contig [2]	Remarks [3]	(without tag)	size (bp) [4]
001AF	CR	P 001	in ORF AAJ76_1000110873 (translation elongation factor EF-2)	GGTGTCATGTAGTTCTTCAGATC	409
001AR	CR	C 0003		CATGACAGGTGCACTCTCTG	
001BF	CR	P 001	in ORF AAJ76_1000109660 (polar tube protein PTP3)	CATAGTCCAATACCGAAATCAGC	395
001BR	CR	C 0003		GCTACGTGTGCTGCAATTTGC	
001CF	CR	P 001	in ORF AAJ76_100013594 (translation initiation factor EIF2a)	GGTAATCAGCCTTCATATGTTGC	431
001CR	CR	C 0040		GGAACATAAGCTTCTTCGATTGTC	
001DF	MS	P 001	F: 86 first nt at the end of ORF AAJ76_100020621 then non coding. R : in ORF	CATTCCTACACTTCTATTATCCAC	356
001DR	CR	C 0040	AAJ76_100020621	GAATCTAGGACTCCTGTTATGG	
001EF	MS	P 001	between the divergent ORFs AAJ76_1000166313 and 1000166727	CAATCTTATGGTTCATTTACAAGGG	440
001ER	NC	C 0128	(ribonucleoprotein)	GAAATGCGCATAAAGGGCTATG	
002AF	CR	P 002	in ORF AAJ76_2000141845 (spore wall protein SWP30)	TGCGGGAATATGAACATCCTAG	453
002AR	CR	C 0032		TGCATCGGTAAAAGTGGCTAGA	
003AF	CR	P 003	F: in ORF AAJ76_3000124483. R: 27 last nt of AAJ76_3000121139 (kinase) then	GATATTAACAACGGATGGACTAGT	420
003AR	MS	C 0263	non coding	CAAAAAGTAACTGAAGCACTAGTG	
003BF	NC	P 003	between the ORFs AAJ76_300029679 (aminopeptidase) and AAJ76_300030593	GTTTACTTGCTTACAACATTGGTTG	505
003BR	NC	C 0289	(origin recognition)	TGGAAATGTGCGGCAGGACA	
004AF	NC	P 004	in intergenic region, far between the ORFs AAJ76_400047493 (MutS) and	CTGTGTATAGTTTTGTCCTGCC	383
004AR	NC	C 0231	AAJ76_400056398	CATTGGAAACATTTAGTTATGTACTC	
004BF	MS	P 004	in long intergenic region, far between the ORFs AAJ76_400062887 and	CAGTTAAGGCATGAATACTACAAC	327
004BR	NC	C 1027	AAJ76_400068918	GTCGCTTTGCTTAAATACAGATTC	
005AF	CR	P 005	in ORF AAJ76_500027606 (phosphoacetyl-glucosamine mutase)	TGACACAGTGCGATTTACCAGT	429
005AR	CR	C 0012		CTCCTGTCTGCACCATTTCAG	
005BF	CR	P 005	in ORF AAJ76_500092411 (hypothetical actin)	GACTCTGGTGATGGTGTCTC	415
005BR	CR	C 0222		CTTTTCTAATATCCACATCACAGG	
006AF	NC	P 006	between the ORFs AAJ76_6000109428 (histone h2b) and AAJ76_6000110666	TTGCTGATCGTTTCATATCAATAC	341
006AR	NC	C 0054	(protein S-S isomerase)	GATGATCGTTTTACAAATGTGATC	
009AF	CR	P 009	in ORF AAJ76_900012560	CAGTAGGAAGATTCATCAAACAGC	412
009AR	CR	C 0221		GTAAGTTGCCTTTGCCACCAAG	
011AF	NC	P 011	in long intergenic region, far between the ORFs AAJ76_1100028057 and	GTACACGACATTCACTACTGAG	490
011AR	NC	C 0624	AAJ76_1100021216	GTCGAGCACATTGTCGTAGC	
011BF	NC	P 011	in intergenic region, far from ORFs AAJ76_1100074951 and upstream of	GATGAAAAGTAGATTAGGTAGTTAG	413
011BR	NC	C 0923	AAJ76_1100079808	CCTAACATAAGAGTAAAAGCGATC	
013AF	CR	P 013	in ORF AAJ76_1300036493	GTGTGATGCCTTTAGCTTTGTC	373
013AR	CR	C 0011		CATTCTTTGAGTGTCTCGCAGA	
013BF	NC	P 013	in long intergenic region, far between the ORFs AAJ76_130004424 and	GCTGCTTAGAATGATTAACAGTTC	464
013BR	NC	C 0143	AAJ76_130006800	CACACGCCTCTCAATTCTCAC	
014AF	CR	P 014	F: in ORF AAJ76_140009109 (zinc finger protein). R: between the ORFs	CGGCATTTATAGCAGCCAACC	417
014AR	NC	C 0001	AAJ76_140009109 and AAJ76_140009564 (transcription factor)	GAGGGGAGGTTGATATTCATC	
016AF	CR	P 016	F: in ORF AAJ76_1600025098 , except the last 8 nt. R: between the following	TGAAGCAGGGGTCAGTTTAG	327
016AR	NC	C 0002	ORFs AAJ76_1600025098 and AAJ76_1600025958	TGTTGAACTTAATTTATGCCACAG	
016BF	CR	P 016	in ORF AAJ76_1600034241 (AAA ATPase)	TGAATATGAAGACGGCACAGGA	487
016BR	CR	C 0002		AACTGCTTCTTCGCATGTCTGA	
017AF	NC	P 017	between the following ORFs AAJ76_1700032044 (histone-binding protein)	GGTGCATAGCAGATTGTACATG	335
017AR	NC	C 0035	and AAJ76_1700031022	GTAGTAGATGTTGTTGGTTATAAGG	
019AF	CR	P 019	in ORF AAJ76_1900011547 (transcription initiation factor subunit)	AGATCCTTACGAACTGCGTGT	411
019AR	CR	C 0057		GGAACATTAGTAGGGACGAAG	
019BF	CR	P 019	in ORF AAJ76_1900028347 (polar tube protein PTP1)	GATTGTATTGTAGGCGGAGGAC	332
019BR	CR	C 0197		GCAGGAACCGCTGATTGTAC	
019CF	CR	P 019	in ORF AAJ76_1900025375 (polar tube protein PTP2)	TGGTAGCCAAGTTGCCACCT	361
019CR	CR	C 0197		GTTGGCTGACTAATAGCATCGTT	
020AF	CR	P 020	F: in the highly putative ORF AAJ76_2000058386. R: between ORFs	CCCACGCCGTGAAAGCAC	462
020AR	NC	C 0566	AAJ76_2000058386 and AAJ76_4310001130	CATCCACCCTTATTGTAACTCC	
021AF	CR	P 021	in ORF AAJ76_210003439 (Mn/Fe-superoxide dismutase)	CTCTAGAGCCTTATATAAGTGAAG	351
021AR	CR	C 0038		ACCACACCCATCCTGATCCA	
021BF	CR	P 021	in ORF AAJ76_2100035271 (RNA polymerase II largest subunit RPB1)	GGTAAAGAAGGTCGTGTTAGAG	389
021BR	CR	C 0247		ACTCGAACGAAATGTCCCATCA	
025AF	CR	P 025	in ORF AAJ76_2500021695 (Asn synthetase)	TCTTAAGTCAGGGCCATCAGTT	407
025AR	CR	C 0004		CCGCTTTGTATTCCTTCACACA	
034AF	NC	P 034	F: upstream of ORF AAJ76_3400023038, including ribosome binding site. R :	GTATATGTTTACTGGTTGTTCTCC	365
034AR	CR	C 0020	in ORF AAJ76_3400023038 , with 5nt upstream	CGACCAGGTACCTAAAAGGC	
036AF	CR	P 036	in ORF AAJ76_3600037707 (alpha-tubulin)	GCAAGGGGTCATTACACTGTTG	397
036AR	CR	C 0262		AAGATACTACTTGTGCCACCAC	
041AF	CR	P 041	in ORF AAJ76_410006816 (hexokinase HK)	TGGCTTTAGCAAAACATTGGTGC	414
041AR	CR	C 0094		GGCCACATTTATAGCCATTTCCT	
043AF	MS	P 043	between the divergent ORFs AAJ76_430009908 and AAJ76_4300012768	CGAAAAGCGAGAAGGGGATG	344
043AR	NC	C 0543	(ribonuclease HII)	ATTGTTACATTCTTGGACACAATGT	
049AF	CR	P 049	in AAJ76_4900027520 (heat shock protein 70)	AGCTGTTTATGATCTGGGAGG	380
049AR	CR	C 0228		GACAGGGGTTTATTGTTTTGTCT	
052AF [5]	MS	P 052	haplotypes 052AF02-09,11 and 052AR02-11,13,14: with microsatellite, far	TGCTTATCCTTCATTATCTATACCA	316
052AR [5]	NC	C 3017	downstrean of ORF AAJ76_520005612 on contig JPQZ01000052	CTAGATGCAAGAATATTCATTGATG	
052AF [5]	NC	P 088	haplotypes 052AF01,10 and 052AR01,12: between the ORFs AAJ76_880001082	TGCTTATCCTTCATTATCTATACCA	292
052AR [5]	NC	C 1509	and AAJ76_880009907 on contig JPQZ01000088	CTAGATGCAAGAATATTCATTGATG	
105AF	MS	P 105	between the convergent ORFs AAJ76_1050007721 (pol protein) and	ACGGACGRGTGTACGAATGG	292
105AR	NC	C 0489	AAJ76_10500010291	CCCTCCGCCTTTCAAACTG	
143AF	MS	P 143	in non conding contig, upstream of region homologous to ORF	GACTGTGTACAGCCCTGCG	449
143AR	NC	C 0313	AAJ76_2460001635	CCTGCCTACTACCCACCAC	
149AF	NC	P 149	in non conding short contig	TATGGATTCACATTTATGCCCCA	353
149AR	MS	C 0460		GTCTTTGAAGTATACGGTTTTCAC	
174AF	MS	P 174	far downstream of ORF AAJ76_1740004145	TGTTTAGCTTTGCAAGTCCCTC	395
174AR	NC	C 0328		CCCTCGCCAACTTCAACGAT	
185AF	MS	P 185	in non conding short contig	GAGTTAACTTGAGCCTCCAGC	419
185AR	NC	C 0292		GTTTCGCGTCCGATTACGAC	
386AF	CR	P 386	in ORF NCER_100768 (endospore protein EnPB1, Roudel *et al*, 2013), data not	AGTATGTCCTAGTAAACCGAGAT	328
386AR [6]	CR	C 0049	exploitable for R	CTGACATCTTTAATTGGACCACA	

[1] CR: coding region, NC: non coding region, MS: microsatellite. [2] P: JPQZ01000xxx according to Pelin *et al.* (2015), C: ACOL0100xxxx according to Cornman *et al.* (2009). [3] All ORFs but the last one were name according to the new annotation in NCBI gene bank (Pelin *et al.* 2015). Missing annotated functions indicate hypothetical proteins. [4] According to Pelin *et al.* (2015) genome. [5] Primers eventually amplified two regions but data were still considered as a whole. [6] The reverse sequencing data could not be analyzed due to large *indels*

Table S3. Frequency of 636 haplotypes of 83 *V. ceranae* markers in honeybees from various archipelagoes of the SWIO and in European and Asian outgroups. For each marker (left column), the haplotypes are listed (2nd left column) and their frequency in every isolate (following columns). See Table 1 for isolate identity. The cumulative haplotypes frequency equals 1 for each marker and each isolate.

		Ⓓ	Ⓒ	①	②	③	①	②	③	④	⑤	⑥	⑦	⑧	⑨	①	②	③	④	⑤	①	②	③	④	⑤	⑥	⑦	①	②	③	①	②	③	④
Marker	haplotype	Ador	Acer1.1	GRE5.6	CAR2.1	POR	RUN0065	RUN0067	RUN0109	RUN0281	RUN1041	RUN1050	RUN1132	RUN1232	RUN1578	MU011I	MU025I	MU040I	ROD001	ROD200	COM11	COM12	COM18	COM28	COM59	COM72	MAY35	SEY059	SEY141	SEY144	ISM03	ISM04	MAD26.1	MAD30.2
001AF	001AF01	0,547	0,3675	0,5507	0,5145	0,4216	0,5028	0,5201	0,4964	0,4992	0,494	0,4986	0,4959	0,5251	0,5059	0,5039	0,5005	0,5146	0,4989	0,4736	0,4989	0,5014	0,4939	0,5079	0,4982	0,4711	0,4933	0,4981	0,4882	0,5048	0,5076	0,5047	0,5843	0,5755
	001AF02	0,4479	0,6269	0,4437	0,4794	0,5717	0,4866	0,4737	0,4993	0,4958	0,5006	0,4962	0,4991	0,4449	0,4821	0,4912	0,4934	0,4794	0,4947	0,5177	0,4962	0,4936	0,5001	0,4878	0,4962	0,5223	0,5008	0,496	0,507	0,4899	0,478	0,49	0,3496	0,4092
	001AF03	0,0009	0,0022	0,0014	0,002	0,0022	0,0074	0,0017	0,0014	0,0016	0,0021	0,0014	0,0011	0,0253	0,0081	0,0015	0,0019	0,0022	0,0016	0,0046	0,0016	0,0016	0,0016	0,0013	0,002	0,002	0,0019	0,0016	0,0016	0,0015	0,0014	0,0017	0,0008	0,0011
	001AF04	0,0016	0,0011	0,0021	0,0016	0,002	0,0014	0,002	0,0012	0,0016	0,0014	0,0019	0,0014	0,0018	0,0016	0,0012	0,0017	0,0014	0,002	0,0011	0,0013	0,0014	0,0013	0,0012	0,0015	0,002	0,0017	0,0017	0,001	0,0015	0,0042	0,0015	0,0218	0,0046
	001AF05	0,0012	0,0007	0,0013	0,0015	0,0011	0,0008	0,0013	0,0007	0,0008	0,001	0,0011	0,0013	0,0017	0,0013	0,0012	0,0015	0,0014	0,0014	0,0017	0,0009	0,0012	0,0016	0,0009	0,0011	0,0013	0,0011	0,0013	0,001	0,0012	0,0044	0,001	0,0236	0,0064
	001AF06	0,0014	0,0015	0,0008	0,0011	0,0016	0,0009	0,0012	0,001	0,001	0,001	0,0009	0,0012	0,0011	0,0011	0,001	0,001	0,001	0,0014	0,0014	0,0011	0,0009	0,0016	0,0009	0,0009	0,0014	0,0011	0,0012	0,0011	0,001	0,0043	0,0011	0,0199	0,0032
001AR	001AR01	0,939	0,939	0,9428	0,9429	0,944	0,9372	0,9383	0,937	0,9369	0,9412	0,9392	0,9413	0,9027	0,9347	0,947	0,9383	0,9385	0,9319	0,9059	0,9423	0,941	0,9435	0,9413	0,9451	0,9429	0,9427	0,9428	0,9374	0,9451	0,9409	0,9382	0,9316	0,9435
	001AR02	0,0274	0,0274	0,0274	0,0276	0,025	0,0238	0,0318	0,0301	0,0257	0,0267	0,0312	0,0262	0,0277	0,0287	0,0238	0,0296	0,0268	0,0302	0,0313	0,0241	0,028	0,0229	0,0284	0,0258	0,0262	0,03	0,0238	0,0282	0,0254	0,0272	0,028	0,028	0,0232
	001AR03	0,0259	0,0279	0,0236	0,0233	0,0245	0,0291	0,0242	0,0291	0,0306	0,0264	0,0246	0,0279	0,0259	0,0246	0,0244	0,0259	0,0273	0,0299	0,024	0,0269	0,0256	0,0259	0,0242	0,0225	0,0229	0,0227	0,027	0,0282	0,0243	0,026	0,0259	0,0268	0,0262
	001AR04	0,0065	0,005	0,0056	0,0056	0,006	0,0092	0,0053	0,0031	0,0063	0,0055	0,0046	0,0041	0,0434	0,0112	0,0043	0,0056	0,0068	0,0065	0,0061	0,0061	0,0048	0,0073	0,0054	0,006	0,0076	0,0043	0,0058	0,0056	0,0045	0,0055	0,006	0,0134	0,0065
	001AR05	0,0013	0,0007	0,0006	0,0006	0,0005	0,0006	0,0005	0,0007	0,0004	0,0002	0,0004	0,0005	0,0004	0,0009	0,0006	0,0007	0,0006	0,0016	0,0326	0,0006	0,0007	0,0003	0,0006	0,0006	0,0004	0,0003	0,0006	0,0007	0,0007	0,0004	0,002	0,0002	0,0006
001BF	001BF01	0,9143	0,7892	0,9584	0,9115	0,9154	0,9878	0,84	0,9937	0,9934	0,995	0,994	0,9938	0,9401	0,9867	0,9929	0,9942	0,9935	0,9916	0,9932	0,9938	0,9941	0,9928	0,9935	0,9921	0,9934	0,993	0,9945	0,9931	0,9947	0,9862	0,9935	0,9854	0,9652
	001BF02	0,0239	0,1692	0,0148	0,0152	0,0586	0,0014	0,0213	0,0011	0,0014	0,001	0,0012	0,0016	0,0013	0,0014	0,0018	0,0017	0,0016	0,0018	0,0017	0,0013	0,0011	0,0015	0,0012	0,0013	0,0013	0,0015	0,0009	0,0018	0,0014	0,0019	0,0013	0,0007	0,0023
	001BF03	0,0245	0,0158	0,0094	0,0312	0,0075	0,0005	0,0606	0,0006	0,001	0,0003	0,0008	0,0007	0,0019	0,0008	0,0011	0,0003	0,0007	0,0005	0,0006	0,0005	0,0006	0,0007	0,0008	0,0014	0,0008	0,0007	0,0009	0,0006	0,0005	0,0006	0,0006	0,0009	0,0007
	001BF04	0,0211	0,0126	0,0071	0,0205	0,0067	0,0018	0,0392	0,0021	0,0019	0,0015	0,0019	0,0018	0,0024	0,0026	0,0021	0,0021	0,0022	0,0022	0,0023	0,0023	0,002	0,0025	0,0019	0,002	0,0023	0,0024	0,0016	0,0022	0,0019	0,002	0,0021	0,0021	0,002
	001BF05	0,0148	0,0117	0,0064	0,0185	0,0049	0,0006	0,0371	0,0007	0,0006	0,0006	0,0006	0,0007	0,0004	0,0007	0,0005	0,0003	0,0005	0,0021	0,0007	0,0005	0,0006	0,0007	0,0006	0,001	0,0009	0,0006	0,0007	0,0004	0,0004	0,0006	0,0009	0,0007	0,0005
	001BF06	0,0005	0,0008	0,0007	0,0008	0,0008	0,001	0,0006	0,0009	0,0008	0,0009	0,0008	0,0007	0,0006	0,0006	0,0007	0,0007	0,0008	0,001	0,0007	0,0008	0,0007	0,0012	0,0006	0,0011	0,0007	0,0012	0,0009	0,001	0,0005	0,0079	0,0008	0,0093	0,0286
	001BF07	0,0009	0,0008	0,0032	0,0023	0,006	0,0069	0,0012	0,0008	0,0008	0,0006	0,0006	0,0007	0,0533	0,0072	0,001	0,0006	0,0007	0,0008	0,0007	0,0009	0,0009	0,0007	0,0014	0,0011	0,0006	0,0005	0,0005	0,0008	0,0006	0,0008	0,0008	0,001	0,0007
001BR	001BR01	0,5581	0,821	0,5792	0,5098	0,6487	0,4896	0,4884	0,4993	0,5047	0,5019	0,5055	0,5095	0,387	0,4825	0,4981	0,5023	0,488	0,5386	0,6559	0,5162	0,5072	0,5001	0,5158	0,5115	0,5221	0,5055	0,5075	0,4768	0,5092	0,5081	0,5634	0,5236	0,5059
	001BR02	0,3828	0,1697	0,3579	0,4811	0,3426	0,5088	0,503	0,4987	0,4935	0,4966	0,4929	0,4886	0,6104	0,5158	0,5003	0,4957	0,5101	0,4574	0,3416	0,4822	0,491	0,4976	0,4825	0,4852	0,4706	0,4928	0,4911	0,5217	0,4894	0,4655	0,434	0,3488	0,415
	001BR03	0,0052	0,0036	0,0054	0,0013	0,0024	0,0005	0,0014	0,0006	0,0004	0,0004	0,0004	0,0004	0,0002	0,0005	0,0005	0,0009	0,0005	0,0005	0,0008	0,0006	0,0005	0,0006	0,0006	0,0007	0,0006	0,0004	0,0004	0,0003	0,0005	0,0075	0,0007	0,0086	0,0274
	001BR04	0,0525	0,0048	0,0567	0,0064	0,005	0,0003	0,0058	0,0005	0,0005	0,0006	0,0003	0,0008	0,0018	0,0005	0,0003	0,0003	0,0005	0,0006	0,0005	0,0004	0,0004	0,0004	0,0004	0,0006	0,0008	0,0006	0,0004	0,0005	0,0003	0,0005	0,001	0,0002	0,0007
	001BR05	0,0002	0	0,0002	0,0002	0,0003	0,0003	0,0003	0,0002	0,0002	0,0002	0,0003	0,0001	0,0002	0,0002	0	0,0003	0,0002	0,002	0,0004	0,0002	0,0002	0,0008	0,0002	0,0011	0,0053	0,0004	0,0002	0,0002	0,0002	0,0062	0,0003	0,0059	0,0235
	001BR06	0,0003	0,0005	0,0004	0,0007	0,0005	0,0002	0,0007	0,0003	0,0003	0	0,0001	0,0002	0,0002	0,0002	0,0004	0,0002	0,0003	0,0004	0,0003	0,0002	0,0002	0,0002	0,0002	0,0002	0,0002	0,0002	0,0001	0,0002	0,0002	0,0074	0,0003	0,1067	0,0066
	001BR07	0,0008	0,0004	0,0002	0,0005	0,0005	0,0003	0,0004	0,0003	0,0003	0,0004	0,0004	0,0004	0,0002	0,0003	0,0003	0,0002	0,0004	0,0004	0,0005	0,0003	0,0004	0,0004	0,0003	0,0005	0,0005	0,0002	0,0003	0,0003	0,0002	0,0048	0,0004	0,0061	0,021
001CF	001CF01	0,4575	0,4697	0,4461	0,5084	0,4931	0,5069	0,5386	0,5067	0,5121	0,5012	0,5075	0,5165	0,5183	0,4934	0,4446	0,2745	0,4472	0,5211	0,5143	0,4825	0,5064	0,4987	0,5133	0,5039	0,4991	0,5108	0,5323	0,6103	0,5331	0,5324	0,5049	0,4119	0,6793
	001CF02	0,5408	0,5279	0,5516	0,4896	0,5046	0,4914	0,4594	0,4917	0,4865	0,4967	0,4909	0,4822	0,4798	0,5048	0,5535	0,7224	0,5515	0,477	0,4833	0,5157	0,4918	0,4994	0,4854	0,4942	0,4997	0,4882	0,4306	0,2054	0,4306	0,4656	0,4933	0,5528	0,3186
	001CF03	0	0	0,0006	0	0	0	0	0	0	0	0	0	0	0	0	0,0002	0	0,0001	0,0001	0,0001	0	0	0	0	0,0002	0	0,0354	0,1834	0,0351	0	0	0	0
	001CF04	0,0017	0,0023	0,0017	0,002	0,0022	0,0017	0,002	0,0014	0,0014	0,0021	0,0015	0,0012	0,0018	0,0017	0,0017	0,003	0,0013	0,0018	0,0023	0,0017	0,0018	0,0019	0,0013	0,0018	0,001	0,0009	0,0017	0,0009	0,0012	0,0019	0,0018	0,0353	0,0021
001CR	001CR01	0,9237	0,6418	0,9082	0,9485	0,8056	0,9935	0,9379	0,9939	0,9931	0,9934	0,9937	0,9941	0,9916	0,9927	0,9878	0,9868	0,9199	0,9917	0,9925	0,9631	0,8337	0,993	0,962	0,9925	0,9922	0,9931	0,993	0,9918	0,9936	0,9937	0,9928	0,9923	0,9913
	001CR02	0,0038	0,0018	0,0029	0,003	0,0021	0,0022	0,0029	0,0022	0,0024	0,0028	0,0022	0,0021	0,0029	0,0027	0,0026	0,0027	0,0028	0,003	0,0023	0,0322	0,1628	0,0023	0,0335	0,0028	0,0028	0,0027	0,0028	0,0028	0,0025	0,0026	0,0027	0,0033	0,0035
	001CR03	0,0296	0,1757	0,0354	0,024	0,0944	0,0014	0,0308	0,0009	0,0011	0,0011	0,0011	0,001	0,0019	0,0011	0,001	0,0009	0,0014	0,0015	0,0016	0,0013	0,0009	0,0011	0,0011	0,0009	0,0012	0,0012	0,0012	0,0017	0,0011	0,001	0,0014	0,0013	0,0019
	001CR04	0,0269	0,1578	0,0272	0,0174	0,084	0	0,0203	0	0	0	0	0	0	0	0	0	0	0	0	0	0	0	0	0	0	0	0	0	0	0	0	0	0
	001CR05	0,0011	0,0005	0	0,0014	0,0007	0,0008	0,0014	0,0011	0,001	0,0007	0,0009	0,0009	0,0008	0,0009	0,0067	0,0076	0,0737	0,0015	0,001	0,0009	0,0009	0,0014	0,0014	0,0014	0,0012	0,0012	0,0009	0,0009	0,001	0,0007	0,0012	0,0008	0,001
	001CR06	0,0043	0,0208	0,0033	0,0037	0,0114	0,0007	0,005	0,0006	0,0006	0,0006	0,0005	0,0005	0,0009	0,0006	0,0003	0,0006	0,0006	0,0007	0,001	0,0006	0,0004	0,0007	0,0005	0,0006	0,0009	0,0003	0,0005	0,0008	0,0003	0,0004	0,0005	0,0007	0,0006
	001CR07	0,0107	0,0015	0,0231	0,0019	0,0018	0,0015	0,0018	0,0013	0,0018	0,0013	0,0016	0,0013	0,0019	0,0018	0,0015	0,0014	0,0016	0,0015	0,0015	0,0017	0,0013	0,0016	0,0014	0,0018	0,0017	0,0014	0,0016	0,002	0,0014	0,0015	0,0015	0,0016	0,0017
001DF	001DF01	0,7322	0,3843	0,7449	0,5618	0,5251	0,5722	0,5503	0,5525	0,5415	0,5628	0,5588	0,5611	0,6866	0,5787	0,5427	0,5624	0,5887	0,5504	0,5464	0,5347	0,5506	0,5627	0,5697	0,5597	0,5773	0,5687	0,5405	0,5024	0,5472	0,5819	0,5672	0,713	0,6085
	001DF02	0,2237	0,2205	0,2262	0,4013	0,3455	0,4256	0,394	0,4445	0,4554	0,4334	0,438	0,4357	0,3109	0,4177	0,4549	0,4342	0,4081	0,4456	0,4496	0,4609	0,446	0,4324	0,4277	0,4366	0,4191	0,4282	0,4559	0,4938	0,449	0,4056	0,43	0,1837	0,3791
	001DF03	0,0201	0,1669	0,0135	0,0115	0,0543	0,0006	0,0153	0,0009	0,0012	0,0016	0,0011	0,0015	0,0012	0,0015	0,0009	0,0012	0,0013	0,0015	0,0016	0,0014	0,0013	0,0021	0,0009	0,0014	0,0013	0,0011	0,0014	0,0011	0,001	0,0012	0,0011	0,0022	0,0018
	001DF04	0,0137	0,1814	0,0089	0,0082	0,0586	0	0,0116	0	0	0	0	0	0	0	0	0	0	0	0	0	0	0	0	0	0	0	0	0	0	0	0	0	0
	001DF05	0,0072	0,0077	0,0039	0,0142	0,0031	0,0014	0,0246	0,001	0,0011	0,001	0,001	0,001	0,0008	0,0012	0,0009	0,0012	0,001	0,0011	0,0011	0,0014	0,001	0,0013	0,0008	0,001	0,0012	0,001	0,001	0,0015	0,0014	0,0013	0,0009	0,0007	0,001
	001DF06	0,0004	0,0001	0,0006	0,0009	0,0008	0,0003	0,0013	0,0007	0,0005	0,0007	0,0008	0,0006	0,0006	0,0008	0,0005	0,0008	0,0007	0,001	0,0009	0,0011	0,0007	0,001	0,0005	0,0009	0,0008	0,0006	0,0007	0,001	0,001	0,0081	0,0007	0,0762	0,0075
	001DF07	0,0026	0,0389	0,0018	0,0017	0,0123	0	0,0025	0	0	0	0	0	0	0	0	0	0	0	0	0	0	0	0	0	0	0	0	0	0	0	0	0	0
	001DF08	0,0001	0,0001	0,0002	0,0004	0,0003	0	0,0005	0,0003	0,0002	0,0004	0,0002	0,0002	0	0,0001	0	0,0002	0,0001	0,0005	0,0003	0,0005	0,0004	0,0005	0,0004	0,0003	0,0003	0,0003	0,0005	0,0002	0,0003	0,0019	0,0002	0,0241	0,002
001DR	001DR01	1	1	1	1	1	1	1	1	1	1	1	1	1	1	1	1	1	1	1	1	1	1	1	1	1	1	1	1	1	1	1	1	1
001EF	001EF01	0,9208	0,7747	0,925	0,549	0,6913	0,5182	0,541	0,5622	0,5151	0,5106	0,5305	0,5259	0,3417	0,5364	0,5946	0,5126	0,536	0,567	0,5184	0,5739	0,5408	0,5435	0,5706	0,5264	0,5419	0,5673	0,5586	0,7348	0,5634	0,5616	0,5781	0,9558	0,3642
	001EF02	0,0148	0,0177	0,0175	0,1473	0,1838	0,0434	0,1195	0,1184	0,0137	0,0535	0,1529	0,0134	0,5527	0,1847	0,3778	0,3665	0,4017	0,3618	0,4294	0,3601	0,3416	0,3508	0,3187	0,3638	0,3982	0,2947	0,3276	0,1456	0,3191	0,3471	0,3361	0,0193	0,6065
	001EF03	0,0214	0,0358	0,0155	0,2682	0,0366	0,4221	0,3033	0,2974	0,457	0,4115	0,2711	0,4497	0,0954	0,2558	0,0122	0,0567	0,03	0,0339	0,0254	0,0304	0,0542	0,0474	0,0531	0,0501	0,0286	0,0969	0,0639	0,1008	0,0639	0,0424	0,04	0,003	0,0166
	001EF04	0,0059	0,0088	0,008	0,0091	0,0105	0,0036	0,0074	0,0066	0,0021	0,0097	0,0169	0,0019	0,0058	0,0088	0,0131	0,0287	0,0215	0,0175	0,0174	0,0215	0,0293	0,0272	0,0332	0,0269	0,0216	0,0238	0,0181	0,016	0,0281	0,0289	0,0259	0,0211	0,0097
	001EF05	0,0003	0,0069	0,0004	0,0061	0,008	0	0,0054	0,0061	0	0	0,0128	0,0001	0,0013	0,0062	0	0,0272	0,0065	0,0136	0,0063	0,0092	0,0228	0,0231	0,018	0,0216	0,0061	0,01	0,0234	0,0008	0,0182	0,0152	0,013	0,0002	0,0012
	001EF06	0,0007	0,0013	0,001	0,008	0,0024	0,0125	0,0071	0,009	0,012	0,0143	0,0156	0,0088	0,002	0,0077	0,0014	0,0076	0,0037	0,0055	0,0025	0,0041	0,0105	0,0072	0,0057	0,0103	0,0027	0,0066	0,0077	0,0017	0,0068	0,0039	0,0063	0,0003	0,0009
	001EF07	0,0036	0,1527	0,0021	0,0102	0,0658	0,0002	0,0143	0,0001	0	0	0,0001	0	0,0008	0,0002	0,0006	0,0004	0,0004	0,0005	0,0005	0,0005	0,0007	0,0006	0,0005	0,0007	0,0006	0,0005	0,0005	0,0002	0,0004	0,0007	0,0003	0,0002	0,0008
	001EF08	0,0325	0,0021	0,0305	0,0021	0,0016	0	0,002	0,0001	0	0,0003	0,0001	0,0002	0,0002	0,0002	0,0004	0,0002	0,0002	0,0002	0,0002	0,0002	0,0001	0	0,0002	0,0002	0,0002	0,0002	0,0002	0,0002	0,0002	0,0002	0,0002	0,0002	0
001ER	001ER01	0,999	0,9988	0,999	0,9987	0,9987	0,9991	0,9988	0,999	0,9992	0,9544	0,8335	0,9993	0,9994	0,9994	0,9985	0,999	0,999	0,9946	0,98	0,999	0,9991	0,9992	0,9987	0,9987	0,9991	0,9572	0,9991	0,9992	0,9992	0,999	0,9959	0,9991	0,9992
	001ER02	0	0	0	0	0	0	0	0	0	0,0448	0,1659	0	0	0	0	0	0	0	0	0	0	0	0	0	0	0,0419	0	0	0	0	0	0,0001	0
	001ER03	0,001	0,0012	0,001	0,0013	0,0013	0,0009	0,0012	0,0009	0,0008	0,0008	0,0006	0,0007	0,0006	0,0006	0,0015	0,001	0,001	0,0054	0,02	0,001	0,0009	0,0008	0,0013	0,0013	0,0008	0,0008	0,0009	0,0008	0,0008	0,001	0,0041	0,0008	0,0008
002AF	002AF01	0,3251	0,6685	0,4459	0,5034	0,5919	0,4124	0,5386	0,42	0,4004	0,3976	0,4026	0,4055	0,4495	0,4066	0,2583	0,3817	0,2998	0,4147	0,3606	0,3756	0,4025	0,4251	0,411	0,427	0,5853	0,3981	0,4305	0,4957	0,4213	0,4218	0,4101	0,4121	0,5601
	002AF02	0,1451	0,0481	0,2266	0,1987	0,1318	0,2031	0,2008	0,2107	0,2039	0,2113	0,2064	0,2074	0,1655	0,2101	0,4505	0,2599	0,364	0,2512	0,3783	0,2477	0,2083	0,2203	0,2075	0,2064	0,1664	0,2312	0,224	0,2757	0,2097	0,2071	0,2265	0,2198	0,1712
	002AF03	0,1304	0,1931	0,2052	0,1826	0,1912	0,2069	0,1532	0,1995	0,1917	0,1975	0,1925	0,1873	0,319	0,2543	0,2377	0,2132	0,2349	0,2203	0,1882	0,2578	0,202	0,1938	0,1892	0,2057	0,1552	0,1995	0,1938	0,1514	0,1861	0,1989	0,1917	0,2143	0,173
	002AF04	0,033	0,0438	0,0594	0,0599	0,0439	0,0949	0,0524	0,0866	0,1055	0,1002	0,1028	0,1045	0,0331	0,0665	0,0271	0,0766	0,0534	0,0577	0,0366	0,0611	0,0974	0,0816	0,1009	0,0847	0,0472	0,0888	0,0791	0,0406	0,095	0,0892	0,0901	0,0604	0,0496
	002AF05	0,0194	0,0332	0,0323	0,0304	0,0268	0,0418	0,0272	0,0418	0,0485	0,0487	0,0482	0,047	0,0194	0,0348	0,0113	0,0345	0,0205	0,0292	0,0144	0,0305	0,046	0,04	0,0437	0,0396	0,0211	0,0397	0,0374	0,0154	0,0447	0,0416	0,0407	0,0385	0,0233
	002AF06	0,0137	0,0078	0,0306	0,025	0,0143	0,0407	0,0214	0,0412	0,0497	0,0445	0,0474	0,0483	0,0134	0,0275	0,0152	0,0339	0,0275	0,0268	0,0218	0,0272	0,0436	0,039	0,0475	0,0364	0,0245	0,0425	0,0351	0,0211	0,0432	0,0413	0,0408	0,0549	0,0228
	002AF07	0,1785	0,0034	0	0	0	0,0001	0,004	0,0001	0,0003	0,0001	0,0002	0,0001	0	0,0001	0	0,0002	0,0001	0	0,0001	0	0,0001	0,0002	0,0002	0,0002	0,0002	0,0002	0	0	0,0002	0,0002	0,0002	0	0,0001

Marker	haplotype	Ⓓ Ador	Ⓒ Acer1.1	① GRE5.6	② CAR2.1	③ POR	① RUN0065	② RUN0067	③ RUN0109	④ RUN0281	⑤ RUN1041	⑥ RUN1050	⑦ RUN1132	⑧ RUN1232	⑨ RUN1578	① MU011I	② MU025I	③ MU040I	④ ROD001	⑤ ROD200	① COM11	② COM12	③ COM18	④ COM28	⑤ COM59	⑥ COM72	⑦ MAY35	① SEY059	② SEY141	③ SEY144	① ISM03	② ISM04	③ MAD26.1	④ MAD30.2
	002AF08	0,1548	0,002	0	0	0	0	0,0023	0	0	0	0	0	0	0	0	0	0	0	0	0	0	0	0	0	0	0	0	0	0	0	0	0	0
002AR	002AR01	0,856	0,9927	0,9955	0,9967	0,9968	0,9973	0,9931	0,9977	0,9971	0,9971	0,9972	0,9971	0,9968	0,9966	0,9972	0,997	0,9968	0,9968	0,9971	0,997	0,9971	0,9971	0,9972	0,9968	0,9962	0,9971	0,9907	0,9721	0,9928	0,9971	0,9974	0,9899	0,9973
	002AR02	0,1439	0,0071	0,0041	0,0031	0,0029	0,0024	0,0065	0,0021	0,0027	0,0026	0,0026	0,0026	0,003	0,0032	0,0026	0,0027	0,003	0,0029	0,0026	0,0029	0,0027	0,0027	0,0026	0,003	0,0036	0,0027	0,0034	0,0043	0,0027	0,0027	0,0024	0,0076	0,0024
	002AR03	0,0001	0,0002	0,0003	0,0002	0,0003	0,0003	0,0004	0,0002	0,0002	0,0003	0,0002	0,0003	0,0002	0,0002	0,0002	0,0003	0,0002	0,0002	0,0003	0,0002	0,0002	0,0002	0,0002	0,0002	0,0002	0,0002	0,0059	0,0236	0,0044	0,0002	0,0002	0,0025	0,0003
003AF	003AF01	0,6496	0,7043	0,6393	0,8876	0,8083	0,9221	0,8882	0,9341	0,9321	0,926	0,9257	0,9309	0,9051	0,9132	0,9211	0,9198	0,9172	0,9083	0,9086	0,9152	0,922	0,9186	0,9252	0,9225	0,9144	0,9231	0,9217	0,9126	0,9265	0,9097	0,9237	0,8837	0,823
	003AF02	0,0505	0,0472	0,046	0,0607	0,0564	0,0589	0,063	0,0496	0,0521	0,0562	0,0564	0,0515	0,0694	0,0646	0,058	0,06	0,0641	0,0675	0,067	0,064	0,0592	0,0613	0,056	0,0574	0,0629	0,0588	0,0579	0,0622	0,0545	0,058	0,0575	0,0668	0,0622
	003AF03	0,0114	0,0136	0,009	0,0167	0,0145	0,0155	0,0162	0,0131	0,0127	0,0152	0,0146	0,0142	0,0212	0,0181	0,017	0,0164	0,0154	0,0181	0,0187	0,0175	0,0155	0,0162	0,0143	0,0154	0,0185	0,0144	0,0165	0,0211	0,0155	0,0154	0,0151	0,0201	0,0166
	003AF04	0,0319	0,2265	0,0448	0,0268	0,1126	0,0007	0,026	0,0004	0,0004	0,0003	0,0005	0,0005	0,0005	0,0008	0,0006	0,0005	0,0005	0,0029	0,0006	0,0006	0,0005	0,0007	0,0007	0,0009	0,0012	0,0004	0,0005	0,0005	0,0006	0,0004	0,0008	0,0032	0,0004
	003AF05	0,0012	0,0006	0,0008	0,0008	0,0005	0,0005	0,0006	0,0008	0,0008	0,0005	0,0007	0,0009	0,0008	0,0006	0,0007	0,0005	0,0006	0,0012	0,0027	0,0007	0,0007	0,0009	0,0008	0,0008	0,0009	0,0006	0,0009	0,0009	0,0007	0,0092	0,0008	0,0185	0,062
	003AF06	0,0012	0,0012	0,002	0,0015	0,0022	0,0015	0,0015	0,0013	0,0012	0,0011	0,0013	0,0013	0,0023	0,0013	0,0018	0,0016	0,0016	0,0015	0,0017	0,0012	0,0012	0,0015	0,0012	0,0017	0,0015	0,0014	0,0015	0,0018	0,0015	0,0062	0,0014	0,0072	0,0349
	003AF07	0,2416	0,0059	0,2369	0,0055	0,0052	0,0006	0,0041	0,0007	0,0006	0,0006	0,0009	0,0006	0,0007	0,0011	0,0008	0,0011	0,0007	0,0005	0,0006	0,0008	0,0008	0,0008	0,0016	0,0012	0,0005	0,0012	0,0009	0,0007	0,0007	0,001	0,0006	0,0005	0,0008
	003AF08	0,0126	0,0006	0,0212	0,0004	0,0004	0	0,0004	0	0	0,0002	0	0	0	0,0001	0	0	0	0	0	0	0	0	0	0	0	0	0	0	0	0	0	0	0
003AR	003AR01	0,1914	0,1993	0,1608	0,333	0,247	0,2901	0,378	0,3334	0,2788	0,2782	0,2759	0,2875	0,4531	0,3329	0,4148	0,3134	0,3432	0,3731	0,3627	0,3618	0,2973	0,28	0,2743	0,2974	0,3162	0,2956	0,3105	0,5267	0,2934	0,3922	0,2868	0,1768	0,4475
	003AR02	0,2329	0,1054	0,2617	0,2618	0,2252	0,2459	0,2399	0,1973	0,2381	0,2553	0,2358	0,2436	0,2881	0,2632	0,2844	0,2547	0,2661	0,2615	0,331	0,2236	0,2338	0,2775	0,2532	0,2504	0,3222	0,2589	0,2539	0,1512	0,2552	0,2031	0,2825	0,2139	0,2471
	003AR03	0,0795	0,1692	0,1042	0,1565	0,1844	0,1623	0,1449	0,1733	0,1559	0,1589	0,1565	0,1598	0,1471	0,1728	0,2092	0,1573	0,1645	0,1496	0,1574	0,1984	0,1635	0,1665	0,1607	0,1626	0,1802	0,1616	0,1599	0,174	0,1602	0,1362	0,1616	0,1466	0,1917
	003AR04	0,0435	0,0442	0,0397	0,099	0,0586	0,1522	0,0895	0,1515	0,1619	0,1489	0,1629	0,154	0,0543	0,1115	0,0435	0,1355	0,1118	0,1049	0,0679	0,1059	0,1497	0,135	0,1548	0,1461	0,0867	0,1397	0,132	0,0798	0,1448	0,1308	0,1304	0,0939	0,0488
	003AR05	0,0451	0,0549	0,039	0,0586	0,0473	0,0851	0,0504	0,0732	0,0945	0,0904	0,0931	0,0865	0,0298	0,0641	0,023	0,0777	0,0643	0,0616	0,0413	0,0551	0,0846	0,0792	0,0893	0,0789	0,0521	0,0821	0,0832	0,0376	0,0864	0,0769	0,0804	0,143	0,0308
	003AR06	0,0218	0,0327	0,0242	0,0431	0,0349	0,0619	0,0369	0,0692	0,0685	0,0658	0,0732	0,0662	0,0228	0,0518	0,0209	0,0581	0,0464	0,0451	0,0291	0,0515	0,068	0,0588	0,0646	0,0618	0,0393	0,0595	0,0567	0,0277	0,0576	0,0548	0,0546	0,0745	0,0255
	003AR07	0,0466	0,2538	0,0351	0,0219	0,1287	0	0,0298	0	0	0	0	0	0	0	0	0	0	0	0	0	0	0	0	0	0	0	0	0	0	0	0	0,0002	0
	003AR08	0,0101	0,0848	0,0093	0,0086	0,0428	0	0,0101	0	0	0	0	0	0	0	0	0	0	0	0	0	0	0	0	0	0	0	0	0	0	0	0	0	0
	003AR09	0,3165	0,0045	0,3154	0,005	0,0047	0,0006	0,0041	0,0004	0,0004	0,0006	0,0005	0,0006	0,0011	0,0008	0,0007	0,0006	0,0006	0,0008	0,0007	0,0005	0,0006	0,0007	0,0007	0,0006	0,0008	0,0006	0,0006	0,0004	0,0006	0,0005	0,0008	0,0005	0,0006
	003AR10	0,001	0,0243	0,0026	0,0052	0,0117	0	0,0078	0	0	0	0	0	0	0	0	0	0	0	0	0	0	0	0	0	0	0	0	0	0	0	0	0	0
	003AR11	0,0091	0,0252	0,006	0,0049	0,0123	0	0,0062	0	0	0	0	0	0	0	0	0	0	0	0	0	0	0	0	0	0	0	0	0	0	0	0	0	0
	003AR12	0	0,0002	0	0,0003	0,0002	0,0003	0,0005	0,0002	0,0003	0,0002	0,0002	0,0002	0,0008	0,0004	0,0003	0,0004	0,0004	0,0011	0,0056	0,0005	0,0003	0,0003	0,0002	0,0002	0,0003	0,0003	0,0007	0,0009	0,0004	0,0009	0,0008	0,0252	0,0014
	003AR13	0	0,0002	0,0007	0,0003	0,0005	0,0002	0,0006	0,0004	0,0003	0,0003	0,0005	0,0003	0,0007	0,0006	0,0006	0,0006	0,0009	0,0008	0,0017	0,0015	0,0005	0,0007	0,0006	0,0005	0,0006	0,0005	0,0006	0,0003	0,0003	0,0007	0,0008	0,0218	0,0014
	003AR14	0,001	0,0003	0,0003	0,0006	0,0006	0,0006	0,0005	0,0003	0,0004	0,0006	0,0003	0,0005	0,0007	0,0006	0,0009	0,0006	0,0006	0,0004	0,0013	0,0004	0,0004	0,0006	0,0005	0,0005	0,0007	0,0004	0,0008	0,0003	0,0004	0,0007	0,0004	0,018	0,0013
	003AR15	0	0,0005	0	0,0005	0,0007	0,0004	0,0005	0,0005	0,0003	0,0003	0,0004	0,0004	0,0009	0,0005	0,0007	0,0004	0,0005	0,0003	0,0006	0,0002	0,0004	0,0003	0,0005	0,0004	0,0004	0,0004	0,0004	0,001	0,0003	0,001	0,0004	0,0218	0,0011
	003AR16	0,0015	0,0004	0,001	0,0004	0,0003	0,0004	0,0003	0,0002	0,0004	0,0002	0,0003	0,0002	0,0005	0,0005	0,0008	0,0004	0,0005	0,0007	0,0006	0,0005	0,0003	0,0004	0,0004	0,0004	0,0004	0,0003	0,0004	0,0001	0,0004	0,0013	0,0004	0,0334	0,0019
	003AR17	0	0	0	0,0002	0,0002	0,0002	0,0002	0,0003	0,0001	0,0002	0,0003	0,0003	0	0,0002	0	0,0002	0,0002	0,0003	0	0,0002	0,0005	0,0002	0,0003	0,0003	0,0001	0,0002	0,0003	0	0,0002	0,001	0,0002	0,0304	0,001
003BF	003BF01	0,514	0,0277	0,508	0,5718	0,2622	0,6185	0,5721	0,6097	0,609	0,6297	0,6487	0,6878	0,4418	0,5465	0,5499	0,6133	0,6036	0,5163	0,5073	0,5497	0,6021	0,5939	0,704	0,6282	0,6411	0,6946	0,7074	0,5699	0,6896	0,6599	0,6889	0,3008	0,5934
	003BF02	0,3367	0,0137	0,3077	0,2425	0,1043	0,1182	0,2612	0,0906	0,1063	0,1266	0,0963	0,0797	0,1889	0,1671	0,1293	0,1389	0,1274	0,1854	0,2224	0,1456	0,1098	0,1495	0,0873	0,13	0,21	0,1228	0,0849	0,2432	0,0971	0,1062	0,1277	0,0639	0,2043
	003BF03	0,0547	0,0268	0,0594	0,0722	0,0811	0,0404	0,0635	0,118	0,0429	0,0502	0,1296	0,0449	0,0982	0,1384	0,2414	0,1259	0,1284	0,1274	0,1017	0,1671	0,2226	0,1284	0,1332	0,1214	0,0583	0,0916	0,0978	0,0364	0,1142	0,1276	0,0963	0,3985	0,0867
	003BF04	0,0267	0,2532	0,037	0,0362	0,1589	0,1256	0,03	0,1115	0,1375	0,1042	0,0566	0,1103	0,1466	0,0611	0,0111	0,0514	0,0828	0,081	0,0797	0,0608	0,0108	0,0573	0,0336	0,0562	0,0259	0,0431	0,0706	0,0477	0,0441	0,0522	0,0489	0,0489	0,057
	003BF05	0,0267	0,0092	0,037	0,0289	0,0295	0,0089	0,027	0,0163	0,0089	0,01	0,0186	0,0059	0,041	0,0449	0,0599	0,0306	0,029	0,0467	0,0439	0,0464	0,043	0,0323	0,0181	0,0239	0,0183	0,015	0,0067	0,0131	0,014	0,0208	0,0176	0,1692	0,0273
	003BF06	0,0119	0,0048	0,0119	0,0198	0,0076	0,0536	0,02	0,0313	0,0586	0,0457	0,0343	0,0503	0,0116	0,0169	0,0044	0,0212	0,0082	0,0024	0,0056	0,006	0,0069	0,0157	0,0158	0,0171	0,0047	0,019	0,016	0,034	0,0252	0,0193	0,0045	0	0,0073
	003BF07	0,0142	0,0902	0,0155	0,0129	0,0572	0,0225	0,0116	0,0159	0,024	0,0195	0,0085	0,0128	0,0659	0,0182	0,002	0,0117	0,017	0,0368	0,035	0,0207	0,0019	0,0145	0,0036	0,0123	0,0085	0,0078	0,0081	0,0189	0,0062	0,0085	0,0133	0,015	0,0207
	003BF08	0,01	0,003	0,0133	0,0103	0,003	0,0109	0,0113	0,0051	0,0113	0,0123	0,0055	0,0065	0,0037	0,005	0,0006	0,0052	0,0019	0,0013	0,0029	0,0007	0,0009	0,0034	0,002	0,0035	0,0016	0,0042	0,0037	0,0188	0,005	0,0036	0,0011	0	0,0019
	003BF09	0,0026	0,4229	0,005	0,0024	0,2216	0	0,001	0	0	0	0	0	0	0	0	0	0	0	0	0	0	0	0	0	0	0	0	0	0	0	0	0	0
	003BF10	0,001	0	0,0027	0,0018	0,0009	0,0013	0,0017	0,0011	0,0012	0,0012	0,0014	0,0015	0,002	0,0015	0,0011	0,0016	0,0014	0,0014	0,0013	0,0018	0,0014	0,001	0,0017	0,0019	0,0022	0,0013	0,0044	0,0178	0,0044	0,0015	0,0015	0	0,0013
	003BF11	0	0	0,0005	0,0002	0	0,0002	0,0003	0,0003	0,0004	0,0005	0,0005	0,0004	0,0002	0,0003	0,0004	0,0003	0,0003	0,0013	0,0002	0,0011	0,0005	0,0039	0,0007	0,0055	0,0292	0,0005	0,0005	0,0002	0,0003	0,0004	0,0002	0	0,0002
	003BF12	0,0016	0,1486	0,0018	0,001	0,0737	0	0,0004	0	0	0	0	0	0	0	0	0	0	0	0	0	0	0	0	0	0	0	0	0	0	0	0	0,0038	0
003BR	003BR01	0,8873	0,0612	0,862	0,8727	0,4026	0,8121	0,8906	0,7468	0,7827	0,8273	0,8197	0,8338	0,6529	0,7381	0,6939	0,7934	0,75	0,7187	0,7413	0,7362	0,7499	0,7972	0,8635	0,8031	0,9012	0,8726	0,825	0,8965	0,8562	0,8153	0,8488	0,3038	0,8232
	003BR02	0,0464	0,3465	0,072	0,063	0,2114	0,1849	0,054	0,1465	0,2136	0,1586	0,0842	0,1621	0,2115	0,0854	0,0135	0,079	0,0993	0,1176	0,1209	0,0751	0,0136	0,0826	0,0447	0,0822	0,0323	0,0573	0,0957	0,0987	0,0667	0,0732	0,0565	0,0819	0,086
	003BR03	0,0589	0,0096	0,058	0,0608	0,0942	0,003	0,0539	0,1057	0,0036	0,014	0,0955	0,0041	0,1341	0,1748	0,2898	0,1265	0,149	0,1625	0,1362	0,1874	0,2347	0,1191	0,0913	0,1136	0,0658	0,0695	0,0791	0,0048	0,0767	0,1102	0,0935	0,5939	0,0904
	003BR04	0,0072	0,5827	0,008	0,003	0,2911	0	0,0011	0	0	0	0	0	0	0	0	0	0	0	0	0	0	0	0	0	0	0	0	0	0	0	0	0,0034	0
	003BR05	0,0003	0	0	0,0006	0,0008	0	0,0005	0,0009	0	0	0,0006	0	0,0015	0,0017	0,0029	0,0011	0,0017	0,0012	0,0016	0,0013	0,0018	0,0011	0,0005	0,0011	0,0008	0,0006	0,0002	0	0,0005	0,0012	0,0011	0,0171	0,0005
004AF	004AF01	0,4274	0,0544	0,4943	0,2952	0,3064	0,3153	0,2904	0,2735	0,2358	0,1913	0,2223	0,3315	0,2965	0,3157	0,3371	0,3119	0,3557	0,2703	0,2884	0,3139	0,3098	0,3328	0,3377	0,3223	0,3822	0,0462	0,2946	0,2319	0,3412	0,3362	0,3198	0,4681	0,3811
	004AF02	0,1913	0,2759	0,1948	0,2423	0,2871	0,2586	0,2302	0,185	0,4168	0,4381	0,2602	0,2263	0,2733	0,3208	0,2921	0,2586	0,2695	0,3354	0,2474	0,2795	0,268	0,2707	0,2669	0,2555	0,2485	0,3457	0,2674	0,2625	0,2491	0,2607	0,2646	0,1278	0,2438
	004AF03	0,1418	0,1825	0,1287	0,1931	0,1478	0,1692	0,1677	0,3017	0,0968	0,1345	0,2615	0,2155	0,0446	0,1882	0,2814	0,229	0,2409	0,2922	0,4021	0,3113	0,2204	0,2402	0,2153	0,2284	0,2797	0,4247	0,2539	0,4173	0,2173	0,1983	0,2097	0,169	0,3082
	004AF04	0,0974	0,037	0,0729	0,1247	0,051	0,1236	0,1453	0,1172	0,1274	0,1331	0,136	0,1202	0,2032	0,0876	0,0418	0,0985	0,0654	0,0453	0,027	0,0432	0,1023	0,0763	0,0924	0,0964	0,0418	0,1025	0,0932	0,0422	0,1068	0,1032	0,1081	0,0933	0,0251
	004AF05	0,0746	0,023	0,0547	0,071	0,0299	0,0845	0,087	0,0533	0,0899	0,0621	0,0603	0,0638	0,155	0,0627	0,0233	0,0557	0,0385	0,0306	0,0156	0,0247	0,0575	0,0479	0,0538	0,0552	0,0245	0,0162	0,0489	0,0226	0,0511	0,0621	0,0628	0,0654	0,0203
	004AF06	0,0243	0,023	0,0156	0,03	0,0144	0,025	0,0339	0,0359	0,0202	0,0327	0,04	0,0228	0,0136	0,0122	0,015	0,0254	0,0177	0,0143	0,0087	0,0125	0,0226	0,016	0,0165	0,0217	0,0103	0,0571	0,0206	0,0104	0,0171	0,0203	0,02	0,0141	0,0056
	004AF07	0,0106	0,006	0,0129	0,0155	0,0065	0,0122	0,0187	0,0167	0,0072	0,0045	0,01	0,0104	0,0073	0,0067	0,0061	0,012	0,0073	0,0071	0,0059	0,0096	0,0114	0,0082	0,0093	0,0117	0,0077	0,0034	0,0125	0,0065	0,0091	0,0103	0,0084	0,0304	0,0082
	004AF08	0,0193	0,0105	0,0106	0,0135	0,0059	0,0112	0,0163	0,0165	0,0058	0,0033	0,0093	0,009	0,0062	0,0058	0,0027	0,0084	0,0046	0,0041	0,0042	0,0046	0,0075	0,0077	0,0076	0,0084	0,0048	0,0036	0,0082	0,0058	0,008	0,0087	0,0063	0,0314	0,0068
	004AF09	0,0134	0,3877	0,0156	0,0147	0,151	0,0004	0,0105	0,0004	0,0002	0,0004	0,0005	0,0004	0,0001	0,0002	0,0006	0,0005	0,0005	0,0007	0,0007	0,0008	0,0004	0,0002	0,0005	0,0005	0,0005	0,0006	0,0005	0,0008	0,0003	0,0003	0,0004	0,0004	0,0008
004AR	004AR01	0,5388	0,1335	0,5614	0,4151	0,3479	0,375	0,4432	0,328	0,3218	0,2931	0,3229	0,3751	0,4423	0,3724	0,3476	0,3602	0,3865	0,2945	0,3044	0,3322	0,36	0,3688	0,3693	0,3713	0,4084	0,2283	0,3448	0,2461	0,3709	0,3789	0,3612	0,529	0,3984
	004AR02	0,281	0,3297	0,2674	0,3338	0,3274	0,3387	0,3391	0,2695	0,4961	0,5105	0,3478	0,2983	0,4222	0,3707	0,3178	0,3362	0,3195	0,3619	0,2647	0,3113	0,3374	0,321	0,3338	0,3228	0,2841	0,3957	0,3161	0,2985	0,3119	0,3275	0,328	0,2143	0,2818
	004AR03	0,1802	0,5368	0,1713	0,2512	0,3247	0,2863	0,2177	0,4025	0,1821	0,1964	0,3293	0,3265	0,1355	0,2569	0,3346	0,3036	0,2941	0,3436	0,431	0,3565	0,3026	0,3101	0,2969	0,3059	0,3075	0,3759	0,3391	0,4554	0,3171	0,2935	0,3109	0,2567	0,3197
004BF	004BF01	0,4583	0,5792	0,4583	0,4571	0,479	0,4555	0,466	0,4103	0,4576	0,5129	0,5397	0,4653	0,4974	0,4529	0,4005	0,4408	0,3767	0,5366	0,4507	0,6898	0,7848	0,7165	0,8511	0,7646	0,5968	0,7468	0,6952	0,4077	0,7878	0,7987	0,7675	0,6926	0,7311
	004BF02	0,4732	0,1255	0,4786	0,4237	0,3393	0,4555	0,4003	0,4105	0,4403	0,373	0,3588	0,4585	0,4885	0,4381	0,3822	0,4433	0,3965	0,2862	0,3084	0,0155	0,0412	0,0299	0,0298	0,03	0,0111	0,0986	0,0759	0,3315	0,0784	0,0338	0,0775	0,232	0,0206
	004BF03	0,0507	0,0325	0,0457	0,0795	0,0677	0,0608	0,0887	0,1008	0,0636	0,0725	0,0697	0,0493	0,0087	0,0711	0,1708	0,0784	0,1783	0,1031	0,1805	0,1956	0,1163	0,1611	0,084	0,1251	0,1974	0,1007	0,1415	0,1623	0,0912	0,089	0,1133	0,0348	0,1116
	004BF04	0,0099	0,096	0,008	0,0248	0,0517	0,0188	0,0311	0,0417	0,0238	0,0259	0,0227	0,017	0,0041	0,0232	0,0414	0,0271	0,0398	0,0493	0,0547	0,0607	0,0401	0,059	0,024	0,0543	0,1382	0,0364	0,0622	0,0543	0,0276	0,0417	0,0305	0,0305	0,0976
	004BF05	0,0034	0,0346	0,0028	0,0096	0,0219	0,006	0,0083	0,0324	0,0132	0,0127	0,0078	0,0085	0,001	0,0127	0,004	0,0087	0,0067	0,0194	0,0045	0,0305	0,0137	0,0264	0,0088	0,0192	0,0194	0,0155	0,0214	0,0405	0,0131	0,0056	0,0091	0,0057	0,0292
	004BF06	0,0041	0,1255	0,0062	0,0044	0,0377	0,0017	0,0045	0,0013	0,0004	0,0018	0,0007	0,0004	0,0001	0,0007	0,0005	0,0008	0,0005	0,0016	0,0007	0,0014	0,0019	0,0032	0,0013	0,0016	0,0014	0,0011	0,002	0,0003	0,0009	0,0006	0,0009	0,0002	0,0003
	004BF07	0	0,0031	0	0,0004	0,0009	0,0006	0,0005	0,0011	0,0002	0,0007	0,0002	0,0006	0	0,0002	0	0,0004	0	0,0005	0,0003	0,002	0,0008	0,0009	0,0004	0,0011	0,0007	0	0,0003	0,0002	0	0,0298	0,0004	0,0041	0,0064
	004BF08	0,0004	0,0036	0,0004	0,0005	0,0017	0,0011	0,0006	0,0019	0,0009	0,0005	0,0003	0,0003	0,0001	0,0011	0,0006	0,0004	0,0014	0,0034	0,0001	0,0045	0,0013	0,0029	0,0007	0,0041	0,035	0,0009	0,0014	0,0031	0,001	0,0008	0,0008	0,0002	0,0032

		Ⓓ	Ⓒ	①	②	③	①	②	③	④	⑤	⑥	⑦	⑧	⑨	①	②	③	④	⑤	①	②	③	④	⑤	⑥	⑦	①	②	③	①	②	③	④
Marker	haplotype	Ador	Acer1.1	GRE5.6	CAR2.1	POR	RUN0065	RUN0067	RUN0109	RUN0281	RUN1041	RUN1050	RUN1132	RUN1232	RUN1578	MU011I	MU025I	MU040I	ROD001	ROD200	COM11	COM12	COM18	COM28	COM59	COM72	MAY35	SEY059	SEY141	SEY144	ISM03	ISM04	MAD26.1	MAD30.2
004BR	004BR01	0,8544	0,7015	0,8575	0,8559	0,7984	0,9059	0,8426	0,802	0,8851	0,8841	0,8811	0,9013	0,9637	0,8875	0,7917	0,8762	0,7667	0,826	0,7732	0,7017	0,8026	0,7439	0,8487	0,7846	0,6012	0,83	0,8015	0,7261	0,8414	0,8122	0,8327	0,8926	0,7422
	004BR02	0,031	0,0197	0,0259	0,0466	0,055	0,0367	0,0543	0,0682	0,0486	0,0474	0,0421	0,0315	0,0048	0,0516	0,1561	0,0542	0,1606	0,0813	0,1451	0,1785	0,0915	0,1271	0,0684	0,0924	0,1628	0,0781	0,1064	0,1418	0,0778	0,0841	0,0907	0,026	0,0924
	004BR03	0,0113	0,0808	0,0105	0,0244	0,0493	0,0166	0,0289	0,0376	0,022	0,0211	0,0209	0,0189	0,0042	0,02	0,0341	0,027	0,0393	0,0426	0,05	0,063	0,037	0,056	0,0299	0,0482	0,1451	0,0389	0,0443	0,0569	0,031	0,0403	0,031	0,0238	0,0995
	004BR04	0,0059	0,0473	0,0068	0,0136	0,0287	0,0124	0,0131	0,0437	0,0118	0,0098	0,0113	0,0113	0,0014	0,0155	0,0051	0,0101	0,016	0,0194	0,0092	0,0351	0,0239	0,0285	0,0167	0,0298	0,0749	0,0208	0,0239	0,0559	0,0211	0,0418	0,017	0,0109	0,0534
	004BR05	0,0309	0,0197	0,026	0,0284	0,0148	0,013	0,0313	0,0252	0,0152	0,0183	0,0263	0,022	0,0094	0,013	0,0084	0,02	0,0124	0,022	0,0142	0,013	0,0259	0,0265	0,0197	0,0252	0,0097	0,0195	0,0102	0,0117	0,0141	0,011	0,0153	0,02	0,0063
	004BR06	0,0253	0,0089	0,0302	0,0189	0,0101	0,0142	0,0213	0,0225	0,0165	0,0188	0,0177	0,0142	0,0161	0,0114	0,0041	0,0117	0,0046	0,0065	0,0077	0,0087	0,0185	0,0175	0,016	0,0192	0,006	0,012	0,0136	0,0074	0,0141	0,0104	0,0124	0,026	0,0055
	004BR07	0,0381	0,0049	0,0381	0,0066	0,0041	0,0012	0,0044	0,0007	0,0008	0,0005	0,0006	0,0008	0,0003	0,001	0,0005	0,0007	0,0004	0,0023	0,0006	0	0,0005	0,0005	0,0006	0,0005	0	0,0005	0	0,0002	0,0006	0,0002	0,0008	0,0008	0,0005
	004BR08	0,0031	0,1172	0,005	0,0056	0,0396	0	0,004	0	0	0	0	0,0001	0	0	0	0	0	0	0	0	0	0	0	0,0001	0,0003	0,0001	0	0	0	0	0	0	0,0001
005AF	005AF01	0,9724	0,9839	0,9843	0,9784	0,9892	0,9964	0,9663	0,9961	0,9959	0,9966	0,996	0,9962	0,9969	0,9968	0,9978	0,9964	0,9961	0,9826	0,9456	0,9957	0,9957	0,9959	0,9954	0,9951	0,9969	0,9956	0,9968	0,9964	0,9968	0,9944	0,9858	0,9962	0,995
	005AF02	0,0239	0,0118	0,0112	0,0173	0,0072	0,0013	0,0296	0,001	0,0013	0,0013	0,0014	0,0013	0,001	0,001	0,0006	0,0011	0,0011	0,0012	0,0011	0,0014	0,0014	0,001	0,0013	0,0013	0,0009	0,0014	0,0009	0,0011	0,0009	0,0013	0,0012	0,0013	0,0014
	005AF03	0,0016	0,0024	0,0009	0,0016	0,0019	0,0012	0,0014	0,0014	0,0013	0,0011	0,0013	0,0012	0,0011	0,0011	0,0009	0,0012	0,0012	0,0065	0,0176	0,0014	0,0014	0,0016	0,0014	0,0016	0,001	0,0015	0,0012	0,0013	0,0012	0,0024	0,0046	0,0018	0,0019
	005AF04	0,0016	0,0013	0,0013	0,0016	0,0009	0,0006	0,0016	0,0008	0,0006	0,0005	0,0007	0,0006	0,0005	0,0007	0,0004	0,0008	0,0009	0,0047	0,0178	0,0009	0,0007	0,0007	0,0011	0,001	0,0006	0,0005	0,0007	0,0005	0,0006	0,0011	0,0045	0,0002	0,0009
	005AF05	0,0003	0,0006	0,0022	0,0012	0,0009	0,0005	0,001	0,0006	0,0008	0,0005	0,0007	0,0007	0,0006	0,0005	0,0002	0,0006	0,0007	0,005	0,0179	0,0006	0,0008	0,0008	0,0007	0,0009	0,0006	0,0009	0,0004	0,0006	0,0006	0,0008	0,0039	0,0005	0,0007
005AR	005AR01	0,9898	0,9936	0,992	0,9898	0,9793	0,9899	0,9929	0,9948	0,9951	0,9934	0,9942	0,9943	0,9519	0,9896	0,9957	0,994	0,9937	0,9906	0,9732	0,9916	0,9939	0,9936	0,9939	0,9932	0,9946	0,9928	0,9952	0,9945	0,9947	0,9715	0,99	0,6917	0,9099
	005AR02	0,0034	0,0012	0,002	0,0019	0,0019	0,0033	0,0021	0,0015	0,0012	0,0019	0,0018	0,0019	0,0445	0,0067	0,0014	0,0017	0,0019	0,0025	0,0024	0,0022	0,0018	0,0024	0,0016	0,0019	0,0016	0,0022	0,0014	0,0018	0,0017	0,0016	0,0017	0,0018	0,0017
	005AR03	0,0018	0,0009	0,0029	0,004	0,0159	0,0012	0,0012	0,0007	0,0003	0,0012	0,0008	0,0007	0,0005	0,0006	0,0006	0,0007	0,0006	0,0007	0,001	0,0009	0,0007	0,0006	0,0009	0,0012	0,0008	0,0011	0,0006	0,0005	0,0008	0,0121	0,0008	0,2347	0,0148
	005AR04	0,0015	0,0015	0,0014	0,0017	0,0007	0,0014	0,0015	0,0006	0,0012	0,0012	0,0009	0,0008	0,0005	0,0006	0,0004	0,0009	0,0005	0,0004	0,0007	0,001	0,0008	0,0009	0,0008	0,0011	0,0005	0,0013	0,0005	0,0008	0,0007	0,0095	0,0006	0,0102	0,0681
	005AR05	0,0025	0,0021	0,0014	0,0019	0,0015	0,0032	0,0017	0,0016	0,0016	0,0014	0,0017	0,0017	0,0019	0,0019	0,0016	0,0019	0,0025	0,0012	0,0019	0,004	0,0019	0,0019	0,0018	0,0017	0,002	0,0018	0,0015	0,0015	0,0015	0,0044	0,0017	0,0608	0,0046
	005AR06	0,0009	0,0006	0,0003	0,0008	0,0007	0,0011	0,0007	0,0008	0,0007	0,0009	0,0007	0,0007	0,0008	0,0006	0,0003	0,0008	0,0008	0,0046	0,0208	0,0004	0,0008	0,0005	0,0009	0,0009	0,0006	0,0009	0,0008	0,0008	0,0006	0,0009	0,005	0,0008	0,0009
005BF	005BF01	0,7345	0,6383	0,7262	0,919	0,7667	0,9948	0,9064	0,9949	0,9946	0,9941	0,9951	0,995	0,9953	0,9951	0,9957	0,9948	0,9951	0,9944	0,9943	0,9878	0,9935	0,994	0,9926	0,9926	0,9941	0,994	0,9956	0,9943	0,995	0,9686	0,9941	0,7881	0,9042
	005BF02	0,0432	0,3345	0,0477	0,0405	0,2107	0	0,0348	0	0	0	0	0	0	0	0	0	0	0	0	0	0	0	0	0	0	0	0	0	0	0	0	0	0
	005BF03	0,0383	0,0176	0,0186	0,0278	0,011	0,0005	0,0472	0,0006	0,0008	0,0009	0,0007	0,0005	0,0004	0,0005	0,0004	0,0005	0,0005	0,0004	0,0007	0,0004	0,0005	0,0009	0,0008	0,0017	0,0008	0,0012	0,0004	0,0006	0,0005	0,0006	0,0005	0,001	0,0005
	005BF04	0	0,0003	0,0006	0,0008	0,0005	0,0004	0,0007	0,0003	0,0006	0,0005	0,0004	0,0004	0,0004	0,0004	0,0003	0,0005	0,0004	0,0004	0,0004	0,0007	0,0008	0,0003	0,0006	0,0011	0,0004	0,0004	0,0005	0,0004	0,0005	0,0135	0,0008	0,015	0,0629
	005BF05	0,003	0,0025	0,0015	0,0025	0,0021	0,0027	0,0025	0,0028	0,0027	0,0029	0,0025	0,0025	0,0026	0,0027	0,0023	0,0027	0,0025	0,0031	0,0028	0,0029	0,0027	0,003	0,003	0,0024	0,0028	0,0029	0,0022	0,0032	0,0027	0,0061	0,0028	0,0074	0,019
	005BF06	0,0016	0,0008	0,0009	0,001	0,0009	0,0009	0,001	0,0008	0,0007	0,001	0,0009	0,0009	0,0007	0,0007	0,0005	0,0007	0,0008	0,0009	0,0008	0,0074	0,0019	0,0009	0,0022	0,0011	0,001	0,001	0,0007	0,0009	0,0007	0,0106	0,001	0,1881	0,0128
	005BF07	0,1794	0,0061	0,2045	0,0084	0,0082	0,0006	0,0073	0,0006	0,0006	0,0006	0,0005	0,0006	0,0006	0,0005	0,0007	0,0008	0,0006	0,0007	0,0009	0,0008	0,0007	0,0008	0,0009	0,0012	0,0008	0,0006	0,0006	0,0006	0,0006	0,0006	0,0007	0,0004	0,0007
005BR	005BR01	0,9952	0,9947	0,9946	0,9958	0,9956	0,9935	0,9958	0,9962	0,9953	0,9964	0,996	0,9958	0,9759	0,9934	0,9952	0,996	0,9961	0,9919	0,9778	0,995	0,9956	0,9958	0,9954	0,9951	0,9956	0,9953	0,9885	0,9511	0,9886	0,9955	0,9933	0,9954	0,9959
	005BR02	0,0013	0,0017	0,0017	0,0012	0,0016	0,0016	0,0011	0,001	0,0014	0,0009	0,0011	0,0014	0,001	0,0013	0,0015	0,0012	0,0011	0,0023	0,0014	0,0014	0,0013	0,0014	0,0014	0,0014	0,0014	0,0011	0,0085	0,0461	0,0085	0,0014	0,0012	0,0009	0,0012
	005BR03	0,0025	0,0024	0,0021	0,0021	0,0021	0,004	0,0021	0,0018	0,0024	0,0017	0,0019	0,0018	0,0221	0,0044	0,0017	0,002	0,002	0,0019	0,0028	0,0021	0,002	0,002	0,0019	0,0023	0,002	0,0019	0,0021	0,002	0,002	0,0022	0,0021	0,0023	0,0019
	005BR04	0,001	0,0011	0,0017	0,0009	0,0007	0,0008	0,001	0,001	0,0009	0,0009	0,001	0,001	0,001	0,0009	0,0017	0,0008	0,0008	0,0038	0,018	0,0015	0,001	0,0008	0,0012	0,0013	0,001	0,0016	0,0009	0,0008	0,0009	0,0009	0,0035	0,0014	0,001
006AF	006AF01	0,0496	0,3246	0,6764	0,4484	0,4068	0,4435	0,4456	0,4574	0,4597	0,472	0,4776	0,4786	0,3933	0,44	0,4602	0,4675	0,5458	0,4747	0,5615	0,4731	0,4726	0,4957	0,5102	0,4523	0,2827	0,4839	0,4434	0,347	0,4795	0,4978	0,5129	0,4165	0,5196
	006AF02	0,0208	0,2578	0,0503	0,3402	0,2383	0,2466	0,3723	0,2721	0,2452	0,2354	0,2388	0,2222	0,2428	0,261	0,2753	0,2289	0,1136	0,2731	0,2248	0,2677	0,2385	0,2142	0,204	0,2581	0,2716	0,2202	0,2477	0,3047	0,2201	0,2045	0,2082	0,4543	0,2435
	006AF03	0,0183	0,3906	0,2616	0,1837	0,3319	0,2573	0,1489	0,2227	0,2411	0,2438	0,2275	0,2455	0,3422	0,2599	0,2359	0,243	0,2212	0,2142	0,1852	0,225	0,2387	0,239	0,2417	0,2348	0,2521	0,2518	0,2666	0,3275	0,2616	0,2513	0,2412	0,0523	0,1941
	006AF04	0,4896	0,0019	0	0	0	0	0,0057	0	0	0	0	0	0	0	0	0	0	0	0	0	0	0	0	0	0,0001	0	0	0	0	0	0	0	0
	006AF05	0,4196	0,0019	0,0003	0	0	0	0,005	0	0	0	0	0	0	0	0	0	0	0	0	0	0	0	0	0	0,0002	0	0	0	0	0	0	0	0
	006AF06	0,0008	0,01	0,0073	0,0121	0,011	0,0265	0,01	0,0217	0,026	0,0226	0,027	0,0251	0,0102	0,0175	0,007	0,0197	0,0154	0,0171	0,0139	0,0163	0,0239	0,0192	0,0226	0,0238	0,0077	0,0222	0,0208	0,0089	0,0201	0,0206	0,0185	0,007	0,0076
	006AF07	0,001	0,0071	0,0028	0,0099	0,0072	0,0172	0,0082	0,018	0,0198	0,0165	0,0199	0,0209	0,0074	0,0136	0,0053	0,0147	0,0056	0,0152	0,0103	0,0118	0,0179	0,025	0,0158	0,0221	0,1831	0,0158	0,0147	0,0091	0,0131	0,0141	0,0144	0,0089	0,0055
	006AF08	0,0002	0,0054	0,0006	0,005	0,0044	0,0086	0,0037	0,0072	0,0076	0,0091	0,0085	0,007	0,0032	0,0074	0,016	0,0256	0,0979	0,0053	0,0038	0,0057	0,0081	0,0063	0,005	0,0084	0,0021	0,0053	0,0062	0,0026	0,0051	0,0054	0,0042	0,003	0,0015
	006AF09	0	0,0004	0	0,0002	0,0002	0,0002	0	0,0002	0,0002	0,0003	0,0004	0,0001	0,0003	0,0003	0,0001	0,0002	0	0	0	0,0001	0	0	0,0002	0	0,0001	0,0005	0,0002	0	0,0001	0,0037	0,0001	0,0558	0,004
	006AF10	0	0,0002	0,0006	0,0006	0,0002	0	0,0006	0,0007	0,0003	0,0003	0,0004	0,0005	0,0005	0,0004	0,0003	0,0003	0,0005	0,0004	0,0005	0,0003	0,0003	0,0005	0,0005	0,0006	0,0002	0,0003	0,0003	0	0,0005	0,0025	0,0004	0,0022	0,0242
006AR	006AR01	0,0088	0,0787	0,0563	0,3216	0,1576	0,25	0,3553	0,2718	0,2483	0,2388	0,2369	0,2312	0,2566	0,2639	0,2873	0,2441	0,2109	0,2685	0,2397	0,2714	0,2424	0,0223	0,2152	0,2531	0,2421	0,227	0,2484	0,2964	0,2255	0,2145	0,2094	0,4399	0,2306
	006AR02	0,027	0,2438	0,4131	0,2392	0,2309	0,2189	0,2451	0,2259	0,2197	0,2331	0,2286	0,2082	0,2025	0,2135	0,24	0,2307	0,2674	0,2435	0,2762	0,2279	0,2248	0,2379	0,2518	0,2199	0,244	0,2443	0,2371	0,2092	0,2545	0,2383	0,2723	0,1974	0,336
	006AR03	0,0178	0,384	0,2626	0,1818	0,3314	0,2485	0,1493	0,208	0,2275	0,225	0,224	0,2401	0,3362	0,2561	0,2409	0,2368	0,2127	0,2115	0,1802	0,219	0,2298	0,2379	0,2293	0,2257	0,2558	0,2386	0,2496	0,3257	0,2443	0,2483	0,2263	0,1113	0,2118
	006AR04	0,0142	0,0693	0,2008	0,1917	0,1499	0,2021	0,1854	0,2078	0,2065	0,2093	0,218	0,224	0,1645	0,2021	0,2005	0,2161	0,2505	0,2118	0,2475	0,2288	0,215	0,4356	0,2149	0,2201	0,2166	0,2046	0,1938	0,1225	0,188	0,2099	0,2095	0,2226	0,1889
	006AR05	0,0013	0,024	0,0226	0,0244	0,029	0,0458	0,0172	0,0482	0,0559	0,0507	0,0523	0,0518	0,0193	0,033	0,0159	0,039	0,0331	0,0352	0,0324	0,0274	0,05	0,0409	0,0508	0,0436	0,0233	0,0469	0,037	0,0252	0,0481	0,0503	0,0481	0,0142	0,018
	006AR06	0,5023	0,0044	0	0	0	0	0,0062	0	0	0	0	0	0	0	0	0	0	0	0	0	0	0	0,0001	0	0,0001	0	0	0	0	0	0	0	0
	006AR07	0,424	0,0035	0	0	0	0	0,0058	0	0	0	0	0	0	0	0	0	0	0	0	0	0	0	0	0	0	0	0	0	0	0	0	0	0
	006AR08	0,0018	0,1694	0,0095	0,0278	0,0806	0,0139	0,026	0,0175	0,0181	0,0174	0,0167	0,0173	0,009	0,0126	0,009	0,014	0,0093	0,0132	0,0099	0,0135	0,014	0,0046	0,0126	0,0139	0,0064	0,0137	0,013	0,0081	0,0124	0,0119	0,011	0,0067	0,0055
	006AR09	0,0011	0,0222	0,0095	0,0125	0,0194	0,0207	0,009	0,0207	0,024	0,0255	0,0234	0,027	0,0117	0,0185	0,0062	0,0191	0,0156	0,0163	0,0138	0,0118	0,0238	0,0196	0,0252	0,0236	0,0113	0,0247	0,0212	0,0129	0,0271	0,0267	0,0232	0,0077	0,0091
	006AR10	0,0017	0,0009	0,0255	0,001	0,001	0,0001	0,0008	0,0002	0,0001	0,0002	0	0,0003	0,0002	0,0003	0,0002	0,0003	0,0003	0	0,0001	0,0002	0,0001	0,0001	0	0,0001	0,0004	0,0001	0	0	0,0001	0,0002	0	0	0
009AF	009AF01	0,0335	0,5184	0,6768	0,4968	0,5509	0,4746	0,492	0,4728	0,475	0,4812	0,4753	0,4951	0,4892	0,4872	0,4927	0,4732	0,4933	0,5191	0,5038	0,5167	0,6593	0,4597	0,5187	0,2264	0,4303	0,4983	0,4686	0,5102	0,4811	0,4895	0,4971	0,2958	0,6187
	009AF02	0,0099	0,4296	0,2034	0,4354	0,3303	0,1962	0,4217	0,2053	0,1984	0,2117	0,1973	0,193	0,1452	0,2133	0,4801	0,4527	0,4662	0,4329	0,4534	0,2047	0,2038	0,2305	0,2026	0,2034	0,212	0,2087	0,2166	0,2029	0,2067	0,2143	0,229	0,3931	0,1801
	009AF03	0,002	0,0021	0,0959	0,0223	0,0983	0,2012	0,0061	0,1993	0,2047	0,2057	0,1981	0,1916	0,148	0,2229	0	0	0,0002	0,0162	0,0194	0,2434	0,0595	0,2471	0,1749	0,4935	0,3325	0,1967	0,2304	0,2404	0,1979	0,1907	0,181	0,1493	0,1547
	009AF04	0,4739	0,0084	0	0	0	0	0,0167	0	0	0	0	0	0	0	0	0	0	0	0	0	0	0	0	0	0	0	0	0	0	0	0	0	0
	009AF05	0,0008	0,0162	0,0107	0,0225	0,0098	0,0315	0,0234	0,0294	0,0296	0,0241	0,0305	0,0291	0,054	0,0192	0,0133	0,0364	0,0179	0,0114	0,007	0,0114	0,0307	0,0135	0,0275	0,0117	0,0042	0,023	0,0203	0,0113	0,0287	0,0263	0,0239	0,0414	0,0144
	009AF06	0,0006	0,0166	0,0115	0,0217	0,0099	0,031	0,0224	0,0275	0,0273	0,026	0,0297	0,0291	0,0548	0,0182	0,0138	0,0376	0,0222	0,0123	0,008	0,01	0,0312	0,0145	0,0294	0,0099	0,0052	0,0231	0,0208	0,01	0,028	0,0266	0,0223	0,0393	0,0114
	009AF07	0,2464	0,003	0	0	0	0	0,007	0	0	0	0	0	0	0	0	0	0	0	0	0	0	0	0	0	0	0	0	0	0	0	0	0	0
	009AF08	0,2329	0,005	0,0002	0	0	0	0,0095	0	0	0	0	0	0	0	0	0	0	0	0	0	0	0	0	0	0	0	0	0	0	0	0	0	0
	009AF09	0	0	0,0004	0,0003	0,0002	0,0217	0,0002	0,0208	0,0207	0,0158	0,0216	0,0186	0,0323	0,011	0	0	0	0,0026	0,0023	0,0044	0,0046	0,0087	0,0144	0,014	0,0031	0,0153	0,0131	0,0073	0,0178	0,015	0,0144	0,0155	0,0054
	009AF10	0	0,0002	0,0002	0,0003	0,0003	0,0199	0,0004	0,0187	0,0192	0,0159	0,0207	0,0198	0,0382	0,0129	0	0	0,0001	0,0025	0,0027	0,0045	0,0052	0,0094	0,0138	0,0106	0,0043	0,0157	0,0132	0,0071	0,0176	0,0145	0,0137	0,0151	0,0053
	009AF11	0	0,0002	0,0009	0,0004	0,0002	0,0131	0,0004	0,0134	0,0138	0,0104	0,0143	0,0121	0,0212	0,0085	0	0	0	0,0014	0,0016	0,0028	0,0033	0,0089	0,0095	0,0142	0,0054	0,0106	0,0096	0,006	0,0112	0,0124	0,0095	0,0257	0,0058
	009AF12	0	0,0001	0	0,0003	0,0001	0,0107	0,0003	0,0128	0,0112	0,0092	0,0126	0,0116	0,0171	0,0068	0	0	0	0,0016	0,0017	0,0021	0,0024	0,0077	0,0091	0,0163	0,0029	0,0085	0,0075	0,0047	0,0109	0,0107	0,0089	0,0249	0,0041
009AR	009AR01	0,0299	0,5364	0,4653	0,5347	0,6553	0,7624	0,5164	0,7506	0,757	0,7537	0,759	0,7624	0,7811	0,759	0,5113	0,5102	0,5043	0,5505	0,5207	0,7804	0,7609	0,7467	0,7623	0,7638	0,7805	0,7567	0,7502	0,7803	0,7547	0,7468	0,7349	0,5414	0,8148
	009AR02	0,0109	0,4408	0,1959	0,4577	0,3387	0,237	0,4427	0,2485	0,2424	0,2452	0,2403	0,2366	0,2182	0,2402	0,4883	0,4891	0,4949	0,4488	0,4785	0,2186	0,2382	0,2524	0,2369	0,2353	0,2188	0,2423	0,249	0,2188	0,2445	0,2521	0,264	0,4574	0,184
	009AR03	0,9516	0,0179	0	0	0	0	0,0356	0	0	0	0	0	0	0	0	0	0,0002	0	0	0	0	0	0	0	0	0	0	0	0,0001	0	0	0	0
	009AR04	0,0075	0,0049	0,3388	0,0076	0,006	0,0006	0,0053	0,0008	0,0006	0,0012	0,0007	0,0009	0,0006	0,0008	0,0004	0,0007	0,0006	0,0007	0,0007	0,0009	0,0009	0,0009	0,0008	0,0009	0,0008	0,0009	0,0008	0,0008	0,0007	0,001	0,001	0,0011	0,0011

Marker	haplotype	Ⓓ Ador	Ⓒ Acer1.1	① GRE5.6	② CAR2.1	③ POR	① RUN0065	② RUN0067	③ RUN0109	④ RUN0281	⑤ RUN1041	⑥ RUN1050	⑦ RUN1132	⑧ RUN1232	⑨ RUN1578	① MU011I	② MU025I	③ MU040I	④ ROD001	⑤ ROD200	① COM11	② COM12	③ COM18	④ COM28	⑤ COM59	⑥ COM72	⑦ MAY35	① SEY059	② SEY141	③ SEY144	① ISM03	② ISM04	③ MAD26.1	④ MAD30.2
011AF	011AF01	0,672	0,6614	0,6937	0,5821	0,6447	0,6222	0,5953	0,5847	0,5804	0,6136	0,6022	0,5125	0,6815	0,6228	0,586	0,5934	0,5676	0,5864	0,6364	0,5753	0,5991	0,5858	0,5939	0,5905	0,442	0,6051	0,548	0,4855	0,439	0,5581	0,5787	0,7237	0,4677
	011AF02	0,1382	0,1687	0,1217	0,2097	0,1916	0,2153	0,1904	0,2656	0,2627	0,2329	0,2462	0,3281	0,1307	0,2151	0,2641	0,2497	0,2695	0,2553	0,221	0,2871	0,2691	0,2562	0,262	0,2534	0,3985	0,262	0,2889	0,3511	0,3949	0,2685	0,2645	0,0931	0,3118
	011AF03	0,0801	0,07	0,0745	0,1074	0,0713	0,072	0,1116	0,0748	0,077	0,0733	0,0721	0,0919	0,0769	0,0712	0,0674	0,0719	0,0783	0,0723	0,0603	0,0658	0,064	0,0741	0,0709	0,075	0,1023	0,0585	0,0833	0,1009	0,1045	0,0839	0,076	0,0482	0,1122
	011AF04	0,0926	0,0842	0,0952	0,0811	0,0796	0,0714	0,0808	0,0569	0,0625	0,0662	0,0614	0,0527	0,0869	0,0757	0,0709	0,071	0,074	0,0758	0,0697	0,059	0,0557	0,0698	0,0592	0,0676	0,0477	0,063	0,0646	0,0494	0,0484	0,069	0,0679	0,1027	0,0721
	011AF05	0,0162	0,0146	0,0137	0,0191	0,0105	0,018	0,0209	0,0171	0,0165	0,0131	0,0173	0,0141	0,0231	0,0144	0,0105	0,013	0,0098	0,0097	0,0115	0,0111	0,011	0,013	0,0128	0,0123	0,0073	0,0103	0,0143	0,0128	0,0125	0,0136	0,0121	0,0183	0,0101
	011AF06	0,001	0,0011	0,0012	0,0006	0,0023	0,0012	0,001	0,0008	0,0009	0,0008	0,0009	0,0007	0,0008	0,0009	0,0011	0,0009	0,0009	0,0005	0,0011	0,0017	0,001	0,0012	0,0012	0,0012	0,0024	0,0011	0,0008	0,0003	0,0006	0,0069	0,0009	0,014	0,0262
011AR	011AR01	0,2432	0,7273	0,2316	0,3672	0,6995	0,3567	0,33	0,3689	0,3664	0,3575	0,3582	0,3787	0,2243	0,3406	0,3536	0,3657	0,4114	0,3815	0,3408	0,3813	0,3834	0,3776	0,4025	0,3713	0,5891	0,38	0,407	0,5125	0,5896	0,4316	0,4233	0,1313	0,4821
	011AR02	0,5536	0,2301	0,5898	0,3564	0,2104	0,3286	0,3797	0,3086	0,3274	0,3292	0,3166	0,3171	0,4773	0,3357	0,3461	0,3219	0,3308	0,3034	0,2591	0,357	0,316	0,3251	0,3039	0,3121	0,1411	0,3162	0,1612	0,2756	0,1279	0,2883	0,2792	0,6943	0,219
	011AR03	0,1487	0,0145	0,1413	0,218	0,0684	0,2795	0,2204	0,2909	0,2852	0,298	0,3023	0,2815	0,1988	0,2948	0,2966	0,3036	0,2534	0,3087	0,395	0,252	0,2918	0,2917	0,2713	0,3084	0,2683	0,2934	0,2965	0,1782	0,2538	0,264	0,2827	0,1319	0,2733
	011AR04	0,0209	0,0045	0,011	0,0194	0,0046	0,0086	0,0228	0,0099	0,0062	0,0031	0,0074	0,0073	0,0301	0,0074	0,0012	0,0021	0,001	0,0017	0,0014	0,0029	0,0024	0,0006	0,0064	0,0017	0	0,0024	0,0028	0,0028	0,0067	0,0038	0,0046	0,0136	0,0014
	011AR05	0,0124	0,0186	0,0073	0,016	0,0124	0,008	0,0182	0,0084	0,0053	0,0047	0,0057	0,0059	0,026	0,0061	0,0008	0,0015	0,0008	0,0014	0,0011	0,0032	0,0028	0,0007	0,0066	0,0017	0,0004	0,0031	0,0019	0,0016	0,005	0,0036	0,0044	0,0093	0,0017
	011AR06	0,0085	0,003	0,0113	0,0127	0,0025	0,0075	0,0158	0,0073	0,0056	0,0042	0,0059	0,0053	0,0229	0,0072	0,0008	0,0024	0,0011	0,001	0,0014	0,0018	0,0016	0,0019	0,0048	0,0023	0,0004	0,0021	0,0034	0,0018	0,0038	0,0024	0,003	0,0083	0,0012
	011AR07	0,012	0,0021	0,0077	0,0101	0,0021	0,011	0,0129	0,0058	0,0037	0,0029	0,0039	0,0041	0,0204	0,0079	0,0008	0,0023	0,0014	0,0019	0,0011	0,0018	0,0018	0,0022	0,0042	0,0022	0,0003	0,0025	0,0013	0,0016	0,0021	0,0025	0,0026	0,008	0,0016
	011AR08	0,0007	0	0	0,0002	0	0,0002	0,0002	0,0003	0,0002	0,0003	0	0,0002	0,0002	0,0002	0	0,0004	0	0,0003	0,0002	0	0,0002	0,0002	0,0003	0,0003	0,0003	0,0003	0,0004	0	0,0003	0,0039	0,0002	0,0033	0,0198
	011AR09	0	0	0	0	0	0	0	0	0	0	0	0	0	0	0	0	0	0	0	0	0	0	0	0	0	0	0,1255	0,0259	0,0108	0	0	0	0
011BF	011BF01	0,6182	0,6214	0,6513	0,5918	0,5073	0,5698	0,6218	0,6075	0,5511	0,5537	0,5683	0,5782	0,6969	0,5892	0,5585	0,5652	0,5891	0,561	0,6097	0,537	0,5826	0,5713	0,5848	0,5477	0,602	0,5753	0,5008	0,4817	0,2568	0,5837	0,5579	0,3691	0,6616
	011BF02	0,3031	0,0966	0,2596	0,3139	0,2063	0,3801	0,3056	0,2076	0,3549	0,393	0,3759	0,3327	0,2764	0,3726	0,416	0,39	0,3827	0,3897	0,323	0,4198	0,3632	0,3854	0,359	0,407	0,3735	0,3777	0,4504	0,4889	0,6914	0,3679	0,3931	0,59	0,2969
	011BF03	0,0561	0,262	0,0374	0,0415	0,1338	0,0048	0,0455	0,1479	0,0436	0,0084	0,006	0,041	0,0047	0,0069	0,0052	0,0062	0,0051	0,0101	0,0064	0,0079	0,0064	0,0062	0,006	0,0068	0,0067	0,0067	0,0073	0,0072	0,0115	0,0067	0,0079	0,0148	0,006
	011BF04	0,012	0,0058	0,0066	0,0147	0,0086	0,0219	0,0133	0,0149	0,0237	0,0254	0,0254	0,0227	0,01	0,0163	0,0106	0,0187	0,0108	0,0165	0,0121	0,0183	0,0244	0,0173	0,025	0,018	0,0089	0,0218	0,0198	0,0099	0,024	0,0215	0,0191	0,0153	0,0087
	011BF05	0,0067	0,0134	0,0088	0,0132	0,0113	0,0225	0,0125	0,0212	0,0257	0,0193	0,0238	0,0247	0,011	0,0143	0,0091	0,019	0,0117	0,0139	0,0122	0,016	0,0228	0,019	0,0242	0,0195	0,0084	0,0177	0,0213	0,0123	0,016	0,0187	0,0195	0,0081	0,0074
	011BF06	0	0	0,0352	0,0223	0,127	0	0,0001	0	0,0001	0	0	0,0001	0	0,0001	0,0001	0	0,0001	0	0	0,0002	0	0	0	0,0001	0	0	0	0	0	0	0	0	0,0001
	011BF07	0	0,0003	0,0011	0,0012	0,0018	0,0006	0,0006	0,0004	0,0005	0	0,0003	0,0002	0,0007	0,0003	0,0001	0,0004	0	0,0084	0,0363	0,0005	0,0003	0,0004	0,0004	0,0004	0,0003	0,0003	0,0003	0	0	0,0004	0,0022	0	0,0004
	011BF08	0,004	0,0003	0	0,0014	0,0038	0,0004	0,0007	0,0004	0,0004	0,0003	0,0003	0,0005	0,0002	0,0003	0,0003	0,0004	0,0003	0,0004	0,0003	0,0003	0,0003	0,0003	0,0005	0,0005	0,0002	0,0005	0	0	0,0002	0,0011	0,0003	0,0026	0,0188
011BR	011BR01	0,3398	0,3637	0,3246	0,3446	0,3396	0,3823	0,3376	0,3421	0,4039	0,408	0,3798	0,3808	0,2991	0,3752	0,4126	0,2054	0,3693	0,397	0,3438	0,4486	0,3754	0,3843	0,3729	0,4067	0,4031	0,3908	0,4486	0,5002	0,6666	0,3815	0,396	0,6446	0,3239
	011BR02	0,2416	0,2935	0,2432	0,1851	0,4608	0,2261	0,225	0,0325	0,4424	0,3205	0,5022	0,2178	0,3016	0,1633	0,3119	0,4555	0,3301	0,2949	0,3704	0,2663	0,2821	0,2506	0,4713	0,237	0,0369	0,5091	0,2618	0,2711	0,2245	0,4937	0,4982	0,0648	0,0785
	011BR03	0,1576	0,2314	0,1944	0,2004	0,1112	0,222	0,1818	0,4806	0,021	0,1364	0,0089	0,2244	0,3241	0,3389	0,2346	0,2065	0,2405	0,2186	0,2211	0,1967	0,1903	0,2176	0,0266	0,1964	0,2227	0,0087	0,1612	0,1722	0,0101	0,026	0,0081	0,2468	0,533
	011BR04	0,0194	0,0227	0,0217	0,0245	0,0239	0,0374	0,0227	0,0248	0,0589	0,0483	0,0562	0,0495	0,0169	0,0254	0,0101	0,0268	0,0139	0,0266	0,0205	0,0284	0,0426	0,0316	0,0616	0,0385	0,0118	0,0465	0,0393	0,0216	0,0493	0,0495	0,0486	0,0153	0,0142
	011BR05	0,0207	0,0273	0,0163	0,0226	0,0141	0,0589	0,0228	0,0597	0,02	0,0353	0,003	0,0587	0,0272	0,0437	0,0138	0,0464	0,0202	0,0258	0,0165	0,0224	0,0463	0,0442	0,0104	0,0448	0,0099	0,0034	0,0351	0,0066	0,0041	0,004	0,0049	0,0072	0,0086
	011BR06	0,0078	0,0094	0,0054	0,0063	0,0133	0,0186	0,0057	0,0045	0,0419	0,0265	0,0468	0,0239	0,0083	0,0109	0,0049	0,0183	0,0095	0,0156	0,0132	0,0172	0,0235	0,0173	0,0487	0,0189	0,0047	0,0382	0,0247	0,0195	0,0421	0,0407	0,041	0,0129	0,008
	011BR07	0,1822	0,0237	0,1629	0,1634	0,022	0,0034	0,1548	0,0001	0,0006	0,0012	0,0005	0,0012	0,0004	0,0011	0,0008	0,0034	0,0008	0,0008	0,0006	0,0004	0,002	0,0021	0,0007	0,002	0,0008	0,0004	0,001	0,0002	0,0002	0,0004	0,0004	0	0,0003
	011BR08	0,0065	0,0095	0,0043	0,0136	0,0054	0,0163	0,0124	0,0033	0,0023	0,0077	0,001	0,013	0,0104	0,0127	0,0067	0,0257	0,008	0,0088	0,0066	0,0076	0,014	0,0298	0,0036	0,0293	0,2941	0,0013	0,0104	0,0035	0,0009	0,0012	0,0014	0,0011	0,0026
	011BR09	0,0116	0,0139	0,0087	0,0166	0,0067	0,0307	0,0162	0,0481	0,0081	0,0141	0,0014	0,0284	0,0116	0,0255	0,0039	0,0103	0,0071	0,0105	0,0068	0,0107	0,0207	0,0199	0,0038	0,0229	0,0148	0,0013	0,0152	0,0042	0,0018	0,0019	0,0011	0,0057	0,0057
	011BR10	0,0129	0,0049	0,0174	0,0225	0,003	0,0038	0,0207	0,0039	0,0009	0,0022	0,0003	0,0022	0,0004	0,0031	0,0007	0,0015	0,0004	0,0011	0,0006	0,0015	0,0031	0,0025	0,0004	0,0032	0,0011	0,0004	0,0026	0,0005	0,0005	0,0006	0,0003	0,0002	0,0008
	011BR11	0	0	0,0011	0,0003	0	0,0004	0,0004	0,0004	0	0	0	0,0001	0	0,0003	0,0002	0,0001	0,0002	0,0002	0,0001	0,0002	0,0001	0	0	0,0001	0,0001	0	0,0002	0,0002	0	0,0005	0	0,0013	0,0244
013AF	013AF01	0,8475	0,9904	0,8735	0,9894	0,9876	0,9971	0,9901	0,9969	0,9972	0,9948	0,9967	0,9964	0,9982	0,997	0,7132	0,7466	0,7103	0,7831	0,7963	0,9975	0,9968	0,9947	0,9958	0,9929	0,9843	0,9965	0,9971	0,9968	0,9973	0,9931	0,9597	0,923	0,9933
	013AF02	0,0017	0,0006	0,0012	0,0014	0,0017	0,0004	0,0012	0,0005	0,0005	0,0008	0,0004	0,0007	0,0003	0,0007	0,2841	0,2514	0,2878	0,2075	0,1756	0,0004	0,0005	0,0025	0,0008	0,003	0,0113	0,0006	0,0007	0,0006	0,0004	0,0007	0,033	0,0011	0,0006
	013AF03	0,0004	0,0029	0,001	0,0016	0,0024	0,0008	0,0012	0,0007	0,0009	0,0014	0,0008	0,0007	0,0004	0,0006	0,0008	0,0006	0,0003	0,008	0,0268	0,0005	0,0006	0,001	0,0007	0,0016	0,0026	0,001	0,0006	0,0006	0,0007	0,0008	0,0053	0,0006	0,0007
	013AF04	0,0026	0,004	0,0024	0,0026	0,0043	0,0007	0,0027	0,0012	0,0009	0,0023	0,0014	0,0014	0,0008	0,001	0,0016	0,0011	0,0011	0,0007	0,001	0,0012	0,0015	0,0014	0,0018	0,0016	0,0011	0,0015	0,0011	0,0014	0,001	0,0047	0,0012	0,0745	0,0048
	013AF05	0,1478	0,0021	0,1219	0,005	0,004	0,001	0,0047	0,0007	0,0005	0,0006	0,0008	0,0008	0,0004	0,0006	0,0003	0,0003	0,0005	0,0007	0,0004	0,0004	0,0006	0,0004	0,0009	0,001	0,0006	0,0004	0,0005	0,0006	0,0006	0,0007	0,0007	0,0008	0,0006
013AR	013AR01	0,3036	0,9753	0,3302	0,7603	0,8611	0,7317	0,7236	0,5413	0,7192	0,614	0,5433	0,7825	0,8248	0,5129	0,5665	0,5026	0,7952	0,7268	0,7443	0,7987	0,8298	0,5151	0,8228	0,7937	0,8096	0,7905	0,7664	0,7432	0,8166	0,4985	0,5725	0,542	0,2657
	013AR02	0,6944	0,0208	0,6686	0,2367	0,1363	0,2655	0,2737	0,457	0,2775	0,2241	0,4411	0,2155	0,1733	0,4844	0,4322	0,4945	0,2028	0,2719	0,2543	0,1994	0,1683	0,4822	0,175	0,2038	0,1891	0,1945	0,2307	0,2543	0,1816	0,447	0,4251	0,3885	0,3889
	013AR03	0,0004	0,0003	0	0,0003	0,0002	0,0004	0,0002	0,0002	0,0002	0	0,0002	0,0002	0,0003	0,0004	0	0,0002	0,0002	0,0002	0,0002	0,0002	0,0005	0	0,0002	0,0004	0,0001	0,0002	0,0004	0,0002	0,0002	0,0433	0	0,0571	0,2895
	013AR04	0,0004	0,0009	0,0003	0,0011	0,0005	0,0016	0,0012	0,0008	0,002	0,1611	0,0143	0,0009	0,0008	0,0013	0,0004	0,0013	0,0007	0,0004	0,0006	0,0007	0,0006	0,0015	0,0006	0,001	0,0004	0,0138	0,0016	0,0014	0,0008	0,0005	0,0013	0,0004	0,0003
	013AR05	0,0004	0	0,0008	0,0004	0	0,0004	0,0003	0,0005	0,0002	0,0002	0,0005	0,0003	0,0002	0,0007	0,0005	0,0007	0,0001	0,0001	0,0003	0,0005	0,0003	0,0006	0,0003	0,0001	0,0002	0,0004	0,0004	0,0002	0,0001	0,0062	0,0005	0,0069	0,0298
	013AR06	0,0008	0,0027	0,0003	0,0012	0,0019	0,0003	0,0009	0,0003	0,0009	0,0006	0,0005	0,0006	0,0006	0,0003	0,0003	0,0007	0,0008	0,0006	0,0004	0,0005	0,0006	0,0005	0,0011	0,0009	0,0005	0,0007	0,0006	0,0006	0,0007	0,0045	0,0006	0,0051	0,0259
013BF	013BF01	0,1907	0,8971	0,9896	0,9762	0,9572	0,9883	0,9637	0,9936	0,9713	0,9649	0,9862	0,9795	0,9642	0,9895	0,9921	0,9924	0,9403	0,9456	0,8655	0,8941	0,9059	0,8902	0,9256	0,8918	0,7153	0,9373	0,907	0,9433	0,9288	0,9053	0,9275	0,3442	0,7935
	013BF02	0,5243	0,004	0	0	0	0	0,0101	0	0	0	0	0	0	0	0	0	0	0	0	0	0	0	0,0001	0	0,0001	0	0	0	0	0	0	0	0
	013BF03	0,2827	0,0027	0	0	0	0	0,0049	0	0	0	0	0	0	0	0	0	0,0002	0	0	0	0	0	0	0	0	0	0	0	0	0,0001	0	0	0
	013BF04	0,0012	0,0786	0,0059	0,0156	0,0315	0,0016	0,0114	0,0018	0,0117	0,0178	0,0092	0,0094	0,0084	0,0013	0,0056	0,0047	0,0564	0,0426	0,1277	0,089	0,0751	0,0748	0,0506	0,0957	0,2779	0,0538	0,0583	0,039	0,0464	0,0609	0,0616	0,0412	0,1737
	013BF05	0,0009	0,0172	0,0036	0,0072	0,0105	0,0093	0,0091	0,0041	0,0166	0,0163	0,0042	0,0103	0,0268	0,009	0,0018	0,002	0,0025	0,0111	0,0064	0,0164	0,0184	0,0342	0,022	0,0118	0,0064	0,0084	0,0345	0,0163	0,0241	0,0155	0,0105	0,0043	0,0075
	013BF06	0,0001	0,0004	0,0008	0,0011	0,0008	0,0008	0,0008	0,0005	0,0004	0,0009	0,0005	0,0006	0,0006	0,0002	0,0005	0,0009	0,0006	0,0007	0,0004	0,0005	0,0006	0,0009	0,0017	0,0006	0,0003	0,0005	0,0002	0,0014	0,0007	0,0181	0,0003	0,6103	0,0254
013BR	013BR01	0,0818	0,9328	0,4584	0,4479	0,6908	0,5219	0,3723	0,5066	0,6377	0,6589	0,5607	0,6385	0,7271	0,5687	0,5112	0,5243	0,6492	0,6294	0,565	0,89	0,9017	0,8935	0,9291	0,8959	0,7253	0,8316	0,8983	0,7588	0,9264	0,9207	0,8962	0,9411	0,8143
	013BR02	0,0981	0,0603	0,5416	0,5521	0,3092	0,4781	0,6083	0,4934	0,3623	0,3411	0,4393	0,3614	0,2728	0,4313	0,4888	0,4757	0,3507	0,3706	0,435	0,11	0,0982	0,1065	0,0708	0,1041	0,2745	0,1683	0,1017	0,2412	0,0734	0,0791	0,1038	0,0589	0,1857
	013BR03	0,5343	0,0034	0	0	0	0	0,0125	0	0	0	0	0	0	0	0	0	0	0	0	0	0	0	0	0	0,0001	0	0	0	0,0001	0	0	0	0
	013BR04	0,2858	0,0034	0	0	0	0	0,0069	0	0	0	0	0	0	0	0	0	0	0	0	0	0	0	0	0	0	0,0001	0	0	0	0,0001	0	0	0
014AF	014AF01	0,9986	0,9995	0,999	0,9993	0,9994	0,6195	0,9993	0,9995	0,9993	0,9996	0,9995	0,9995	0,9734	0,9734	0,9997	0,9996	0,9994	0,9991	0,9993	0,9997	0,9993	0,9993	0,9994	0,9996	0,9995	0,9994	0,9994	0,9997	0,9995	0,9994	0,9993	0,9993	0,9994
	014AF02	0,0014	0,0005	0,001	0,0007	0,0006	0,3805	0,0007	0,0005	0,0007	0,0004	0,0005	0,0005	0,0266	0,0266	0,0003	0,0004	0,0006	0,0009	0,0007	0,0003	0,0007	0,0007	0,0006	0,0004	0,0005	0,0006	0,0006	0,0003	0,0005	0,0006	0,0007	0,0007	0,0006
014AR	014AR01	0,5572	0,7308	0,5458	0,5395	0,6304	0,5094	0,5597	0,5021	0,4857	0,4889	0,4969	0,488	0,6168	0,4971	0,3039	0,4831	0,46	0,5257	0,6061	0,4843	0,5044	0,4952	0,4965	0,4992	0,513	0,4918	0,4914	0,4932	0,4989	0,4845	0,499	0,3525	0,51
	014AR02	0,4089	0,1025	0,4094	0,4268	0,2355	0,4673	0,4116	0,4802	0,4925	0,4878	0,4798	0,4899	0,3538	0,4766	0,6748	0,4924	0,5132	0,4476	0,3647	0,4897	0,4731	0,4795	0,481	0,4766	0,4586	0,484	0,4833	0,4808	0,4799	0,491	0,4773	0,6195	0,4676
	014AR03	0,0108	0,0215	0,0148	0,0135	0,0191	0,012	0,0135	0,009	0,0105	0,0119	0,0115	0,0108	0,0169	0,0139	0,0074	0,0119	0,012	0,0148	0,0197	0,0121	0,0112	0,0128	0,0111	0,0124	0,0141	0,0124	0,0117	0,0116	0,0107	0,012	0,0122	0,0104	0,0115
	014AR04	0,0089	0,0032	0,01	0,0113	0,006	0,0108	0,0102	0,0084	0,0109	0,0112	0,0114	0,011	0,0119	0,0122	0,0136	0,0122	0,0145	0,0116	0,0092	0,0134	0,011	0,0121	0,0108	0,0113	0,0133	0,0111	0,013	0,0133	0,01	0,012	0,0112	0,0176	0,0106
	014AR05	0,0141	0,142	0,02	0,0089	0,1089	0,0005	0,005	0,0004	0,0003	0,0003	0,0004	0,0004	0,0005	0,0003	0,0002	0,0004	0,0003	0,0003	0,0004	0,0004	0,0003	0,0005	0,0005	0,0006	0,001	0,0006	0,0006	0,001	0,0005	0,0005	0,0003	0	0,0003
016AF	016AF01	0,6378	0,3914	0,6745	0,4414	0,5235	0,4383	0,4203	0,4192	0,4061	0,4877	0,4663	0,4	0,5221	0,4317	0,7323	0,7171	0,7491	0,7369	0,7268	0,7818	0,9211	0,7385	0,7671	0,7255	0,7115	0,6534	0,6048	0,2279	0,6372	0,7345	0,9364	0,8828	0,935
	016AF02	0,3339	0,5877	0,3057	0,5323	0,4554	0,5142	0,5558	0,5357	0,5513	0,4656	0,4933	0,5622	0,4408	0,538	0,2448	0,2531	0,2309	0,236	0,2534	0,1911	0,0639	0,2343	0,207	0,2454	0,2685	0,3123	0,3478	0,7421	0,3282	0,233	0,0511	0,019	0,0141
	016AF03	0,0079	0,0097	0,0108	0,0127	0,0089	0,0215	0,0113	0,0235	0,0224	0,024	0,0219	0,0187	0,0086	0,0137	0,0101	0,0134	0,0077	0,0115	0,0069	0,0111	0,0063	0,0124	0,0114	0,0135	0,0083	0,0158	0,0195	0,0056	0,0129	0,0108	0,0043	0,0029	0,0028
	016AF04	0,0142	0,0086	0,0036	0,011	0,0084	0,021	0,0102	0,0195	0,0179	0,0202	0,0161	0,0171	0,0081	0,0125	0,0088	0,0127	0,0086	0,0113	0,0091	0,0114	0,0056	0,0115	0,0097	0,0108	0,0086	0,0147	0,0187	0,005	0,0134	0,0089	0,0046	0,0039	0,0041

Marker	haplotype	Ⓓ Ador	Ⓒ Acer1.1	① GRE5.6	② CAR2.1	③ POR	① RUN0065	② RUN0067	③ RUN0109	④ RUN0281	⑤ RUN1041	⑥ RUN1050	⑦ RUN1132	⑧ RUN1232	⑨ RUN1578	① MU011I	② MU025I	③ MU040I	④ ROD001	⑤ ROD200	① COM11	② COM12	③ COM18	④ COM28	⑤ COM59	⑥ COM72	⑦ MAY35	① SEY059	② SEY141	③ SEY144	① ISM03	② ISM04	③ MAD26.1	④ MAD30.2
	016AF05	0,0031	0,0003	0,0018	0,0004	0,0003	0,0006	0,0004	0,0002	0,0003	0,0002	0,0004	0,0002	0,0003	0,0002	0,0009	0,0005	0,0004	0,0012	0,0005	0,0002	0,0003	0,0007	0,0008	0,001	0,0002	0,0003	0,0072	0,0185	0,0064	0,0015	0,0003	0,0025	0,0059
	016AF06	0	0,0001	0	0,0002	0,0004	0,0004	0,0002	0,0005	0,0005	0,0006	0,0004	0	0,0002	0,0002	0,0006	0,0005	0,0008	0,0007	0,0004	0,0006	0,0005	0,0004	0,0015	0,0009	0,0003	0,0009	0,0008	0,0002	0,0005	0,0043	0,0005	0,0052	0,0224
	016AF07	0	0,0007	0,0018	0,0011	0,0011	0,0032	0,0011	0,0008	0,001	0,0015	0,0012	0,0008	0,0197	0,0033	0,0019	0,0018	0,0018	0,0012	0,0016	0,003	0,0015	0,0012	0,0019	0,0014	0,0014	0,0018	0,0005	0,0005	0,0008	0,0019	0,0017	0,0022	0,002
	016AF08	0	0,0014	0,0009	0,0007	0,0015	0,0003	0,0005	0,0003	0,0005	0,0002	0,0002	0,0005	0,0003	0,0002	0,0003	0,0004	0,0005	0,0002	0,0005	0,0005	0,0006	0,0008	0,0002	0,0008	0,0007	0,0004	0,0003	0,0002	0,0005	0,0033	0,0006	0,0524	0,0107
	016AF09	0,0031	0	0,0009	0,0003	0,0005	0,0006	0,0003	0,0002	0,0001	0	0,0002	0,0003	0,0002	0,0002	0,0003	0,0005	0,0002	0,0009	0,0008	0,0002	0,0002	0,0002	0,0004	0,0007	0,0005	0,0003	0,0003	0,0001	0	0,0018	0,0005	0,029	0,003
016AR	016AR01	0,5313	0,3403	0,5738	0,3905	0,4688	0,3866	0,3715	0,3539	0,3331	0,3759	0,3485	0,3284	0,5179	0,3793	0,7339	0,698	0,7244	0,7255	0,7081	0,7801	0,9177	0,7235	0,7533	0,6916	0,7001	0,3582	0,5934	0,2248	0,6069	0,719	0,9285	0,9424	0,9739
	016AR02	0,4063	0,5963	0,352	0,5373	0,4674	0,5136	0,5617	0,5507	0,5718	0,5197	0,5601	0,5914	0,4415	0,55	0,0718	0,2233	0,2208	0,2215	0,255	0,1832	0,061	0,2202	0,2005	0,2413	0,265	0,5745	0,3378	0,7457	0,3404	0,2448	0,0539	0,0176	0,0165
	016AR03	0,0163	0,0263	0,0183	0,0306	0,0251	0,0415	0,0258	0,0423	0,0417	0,0495	0,0388	0,0341	0,0131	0,0286	0,1815	0,0455	0,0314	0,0227	0,0144	0,0165	0,0071	0,0239	0,0194	0,0301	0,0143	0,0281	0,028	0,0108	0,0198	0,0116	0,0053	0,0009	0,001
	016AR04	0,0238	0,0311	0,0292	0,0357	0,0333	0,0521	0,0346	0,0492	0,05	0,0499	0,0474	0,0405	0,0222	0,0361	0,0117	0,0309	0,0214	0,028	0,0198	0,0174	0,0133	0,0304	0,0247	0,0353	0,0183	0,034	0,0366	0,0124	0,0299	0,0207	0,0114	0,0067	0,0054
	016AR05	0,0213	0,0059	0,0259	0,0057	0,0051	0,0062	0,0062	0,0038	0,0032	0,005	0,0048	0,0055	0,005	0,0058	0,0008	0,0019	0,0017	0,0019	0,0023	0,0022	0,0005	0,0012	0,0016	0,0015	0,0018	0,0045	0,0042	0,0061	0,0027	0,0014	0,0005	0,0001	0,0001
	016AR06	0,0013	0,0001	0,0008	0,0002	0,0003	0,0001	0,0003	0,0002	0,0002	0	0,0003	0	0,0003	0,0001	0,0003	0,0003	0,0003	0,0004	0,0004	0,0005	0,0004	0,0008	0,0005	0,0003	0,0006	0,0007	0	0,0001	0,0003	0,0024	0,0004	0,0323	0,003
016BF	016BF01	0,7522	0,5633	0,761	0,5976	0,5745	0,4939	0,6126	0,4893	0,4936	0,4898	0,5018	0,4925	0,4702	0,4878	0,4778	0,492	0,4754	0,4935	0,4502	0,5378	0,6384	0,4998	0,5449	0,6861	0,479	0,4965	0,5026	0,5218	0,5052	0,502	0,4964	0,6288	0,4593
	016BF02	0,2473	0,4363	0,2381	0,4018	0,4247	0,5061	0,3868	0,5103	0,5059	0,5095	0,4977	0,5071	0,5296	0,5119	0,5219	0,5077	0,5244	0,5062	0,5494	0,4617	0,361	0,4999	0,4546	0,3126	0,5206	0,503	0,4972	0,4767	0,4944	0,4875	0,5031	0,3139	0,5034
	016BF03	0,0004	0,0005	0,0009	0,0007	0,0008	0	0,0007	0,0004	0,0005	0,0006	0,0005	0,0004	0,0002	0,0004	0,0003	0,0003	0,0002	0,0003	0,0003	0,0005	0,0006	0,0004	0,0005	0,0013	0,0004	0,0005	0,0002	0,0014	0,0004	0,0105	0,0005	0,0573	0,0373
016BR	016BR01	0,7611	0,5471	0,8029	0,5885	0,6484	0,4906	0,5985	0,4899	0,4828	0,5311	0,5238	0,488	0,4772	0,4897	0,7329	0,7399	0,7312	0,8043	0,7207	0,7761	0,9323	0,7387	0,7738	0,7493	0,7512	0,7114	0,7513	0,6565	0,7486	0,7395	0,94	0,7466	0,6618
	016BR02	0,2127	0,4492	0,1941	0,4093	0,3492	0,5076	0,3971	0,5083	0,5154	0,4666	0,4742	0,5101	0,5209	0,5084	0,2639	0,2572	0,2645	0,1903	0,2559	0,2202	0,0639	0,259	0,2228	0,2469	0,2458	0,2858	0,2457	0,3407	0,2488	0,2553	0,0548	0,2225	0,3334
	016BR03	0,0019	0,0009	0,002	0,0014	0,0014	0,0009	0,0015	0,001	0,0013	0,0014	0,0013	0,0012	0,0013	0,0013	0,0017	0,0018	0,0035	0,0035	0,022	0,0018	0,0022	0,0015	0,0016	0,0017	0,002	0,0017	0,0019	0,0012	0,0016	0,0021	0,0033	0,0021	0,002
	016BR04	0,0237	0,0022	0,0006	0,0004	0,0006	0,0006	0,0025	0,0005	0,0003	0,0003	0,0003	0,0004	0,0002	0,0004	0,0005	0,0005	0,0004	0,0009	0,0009	0,001	0,0009	0,0003	0,0009	0,0012	0,0005	0,0008	0,0009	0,0012	0,0006	0,0006	0,001	0,0006	0,0006
	016BR05	0,0006	0,0005	0,0004	0,0004	0,0004	0,0003	0,0004	0,0003	0,0003	0,0005	0,0004	0,0003	0,0005	0,0003	0,0011	0,0005	0,0004	0,0011	0,0005	0,0008	0,0008	0,0005	0,0009	0,0008	0,0004	0,0003	0,0003	0,0003	0,0005	0,0025	0,0009	0,0281	0,0021
017AF	017AF01	0,261	0,2699	0,5091	0,4721	0,3687	0,4495	0,4474	0,4737	0,4693	0,4764	0,4593	0,4837	0,4554	0,3314	0,5249	0,4897	0,5391	0,5045	0,5207	0,512	0,467	0,4727	0,4659	0,1946	0,4953	0,4784	0,2053	0,4007	0,1763	0,3621	0,4777	0,3651	0,5903
	017AF02	0,1003	0,2492	0,184	0,2219	0,2712	0,2248	0,212	0,2201	0,2146	0,22	0,2165	0,207	0,2126	0,2707	0,253	0,2174	0,2191	0,2107	0,2077	0,1907	0,2119	0,2508	0,2168	0,5136	0,2293	0,2191	0,5342	0,4387	0,5339	0,3767	0,2132	0,3612	0,2024
	017AF03	0,1041	0,2759	0,1745	0,2349	0,2505	0,2256	0,2491	0,2212	0,2331	0,232	0,2364	0,2262	0,2085	0,2246	0,1959	0,2296	0,2031	0,2523	0,2483	0,2601	0,24	0,2306	0,2383	0,2324	0,2492	0,2339	0,2134	0,1258	0,2223	0,1771	0,2366	0,0975	0,1601
	017AF04	0,0327	0,0095	0,0653	0,0241	0,0101	0,0368	0,0249	0,0386	0,0366	0,0264	0,0348	0,0408	0,0671	0,021	0,0074	0,0247	0,0114	0,009	0,0046	0,0142	0,0322	0,013	0,0334	0,0146	0,0053	0,0274	0,0135	0,0078	0,0209	0,0351	0,0291	0,0744	0,0199
	017AF05	0,015	0,0066	0,0232	0,0145	0,0064	0,0301	0,0161	0,0178	0,0163	0,0163	0,0199	0,0149	0,0278	0,1226	0,0058	0,0135	0,009	0,0058	0,0035	0,0075	0,0166	0,011	0,0165	0,0165	0,0051	0,0138	0,0113	0,006	0,0182	0,0237	0,0145	0,0651	0,012
	017AF06	0,0154	0,0069	0,0302	0,0147	0,007	0,025	0,0181	0,0211	0,0232	0,0205	0,025	0,0201	0,0212	0,0191	0,0052	0,0168	0,0102	0,0081	0,0035	0,0074	0,0237	0,0121	0,0213	0,0097	0,0062	0,0198	0,0063	0,0054	0,0113	0,0119	0,02	0,0215	0,0064
	017AF07	0,0037	0,0109	0,0055	0,008	0,0115	0,0073	0,0075	0,0067	0,0062	0,0078	0,0075	0,0067	0,0067	0,01	0,0075	0,0077	0,0074	0,0086	0,0112	0,0074	0,0079	0,0093	0,0073	0,0174	0,0085	0,007	0,0155	0,0145	0,0159	0,0128	0,0084	0,0144	0,0083
	017AF08	0,0091	0,143	0,0063	0,0076	0,0655	0	0,011	0	0	0	0	0	0	0	0	0	0	0	0	0	0	0	0	0	0	0	0	0	0	0	0	0	0
	017AF09	0,2192	0,0017	0	0	0	0	0,0047	0	0	0	0	0	0	0	0	0	0	0	0	0	0	0	0	0	0	0	0	0	0	0	0	0	0
	017AF10	0,15	0,004	0	0	0	0	0,0036	0	0	0	0	0	0	0	0	0	0	0	0	0	0	0	0	0	0	0	0	0	0	0	0	0	0
	017AF11	0,0878	0,0019	0	0	0	0	0,0026	0	0	0	0	0	0	0	0	0	0	0	0	0	0	0	0	0	0	0	0	0	0	0	0	0	0
	017AF12	0,0017	0,0205	0,002	0,0022	0,0092	0,0008	0,0029	0,0007	0,0008	0,0006	0,0006	0,0006	0,0007	0,0006	0,0002	0,0007	0,0007	0,001	0,0005	0,0008	0,0008	0,0005	0,0005	0,0011	0,001	0,0006	0,0005	0,001	0,0012	0,0006	0,0005	0,0007	0,0006
017AR	017AR01	0,2735	0,2392	0,5376	0,3061	0,3569	0,5098	0,2888	0,4957	0,5087	0,5059	0,5095	0,5099	0,4891	0,4795	0,522	0,5188	0,5503	0,5176	0,5233	0,5261	0,5117	0,489	0,4982	0,2333	0,4826	0,5037	0,24	0,4044	0,2202	0,3943	0,5112	0,4604	0,5972
	017AR02	0,1604	0,2705	0,2705	0,4339	0,299	0,2536	0,426	0,2709	0,2428	0,248	0,2386	0,2472	0,2975	0,273	0,2704	0,2366	0,2405	0,2235	0,2185	0,2031	0,2301	0,2687	0,2494	0,5272	0,2418	0,2441	0,5272	0,4659	0,5508	0,4128	0,2386	0,3829	0,2326
	017AR03	0,1108	0,2893	0,1822	0,2483	0,2582	0,2359	0,2541	0,2329	0,248	0,2459	0,2513	0,2424	0,2131	0,2466	0,2069	0,244	0,2086	0,2581	0,2578	0,2705	0,2579	0,2412	0,2518	0,238	0,275	0,2518	0,2314	0,1292	0,228	0,1836	0,2497	0,0835	0,1601
	017AR04	0,0108	0,1878	0,0091	0,0112	0,0851	0,0002	0,015	0,0001	0,0002	0,0001	0,0001	0,0001	0	0	0,0001	0	0	0,0002	0	0	0	0	0	0	0	0	0	0	0,0001	0	0	0	0
	017AR05	0	0	0,0001	0,0003	0,0003	0	0,0003	0,0002	0,0002	0	0	0,0002	0,0002	0,0002	0,0001	0,0002	0,0003	0,0002	0,0002	0,0003	0	0,0002	0,0001	0,0006	0,0003	0,0001	0,0004	0,0003	0,0004	0,0069	0,0002	0,0523	0,0078
	017AR06	0,2047	0,0049	0	0	0	0	0,0066	0	0	0	0	0	0	0	0	0	0	0	0	0	0	0	0	0	0	0	0	0	0	0	0	0	0
	017AR07	0,1524	0,0042	0	0	0	0	0,0057	0	0	0	0	0	0	0	0	0	0	0	0	0	0	0	0	0	0	0	0	0	0	0	0	0	0
	017AR08	0,0004	0,0003	0,0005	0,0003	0,0006	0,0005	0,0003	0,0002	0,0001	0,0001	0,0004	0,0002	0	0,0007	0,0004	0,0003	0,0002	0,0004	0	0	0,0001	0,0007	0,0004	0,0009	0,0003	0,0002	0,001	0,0001	0,0005	0,0023	0,0002	0,0208	0,0022
	017AR09	0,0869	0,0039	0	0	0	0	0,0033	0	0	0	0	0	0	0	0	0	0	0	0	0	0	0	0	0	0	0	0	0	0	0	0	0	0
019AF	019AF01	0,1634	0,6895	0,1507	0,3178	0,5629	0,4047	0,3071	0,4674	0,4313	0,4076	0,2172	0,4216	0,1851	0,3331	0,5142	0,4679	0,5677	0,4941	0,4315	0,5153	0,4547	0,4488	0,4474	0,4668	0,5502	0,4206	0,4387	0,2424	0,4222	0,4822	0,4783	0,5775	0,5802
	019AF02	0,2266	0,1998	0,1895	0,4559	0,2667	0,3552	0,4885	0,3644	0,3556	0,3511	0,4477	0,3693	0,4413	0,4143	0,4297	0,376	0,3906	0,3688	0,466	0,3433	0,3346	0,3822	0,3366	0,3444	0,3481	0,3723	0,3951	0,6232	0,3752	0,3579	0,3361	0,267	0,3542
	019AF03	0,561	0,048	0,6117	0,1516	0,1135	0,1023	0,1285	0,0608	0,0766	0,1028	0,2378	0,0705	0,345	0,1608	0,02	0,0535	0,014	0,0488	0,0346	0,0473	0,0711	0,0603	0,0711	0,0649	0,0313	0,0877	0,0527	0,0185	0,0624	0,053	0,06	0,0544	0,0218
	019AF04	0,0093	0,0271	0,0055	0,0228	0,026	0,0851	0,02	0,0671	0,0826	0,0815	0,0453	0,0804	0,0114	0,0517	0,0197	0,0631	0,0147	0,0506	0,031	0,054	0,0855	0,0685	0,0834	0,0775	0,0397	0,0694	0,0574	0,0193	0,0746	0,063	0,0741	0,067	0,0251
	019AF05	0,0049	0,0106	0,0059	0,0108	0,0108	0,0297	0,0098	0,022	0,0263	0,031	0,0284	0,0292	0,0067	0,0213	0,0061	0,0206	0,0045	0,0164	0,0096	0,0178	0,0276	0,0209	0,0295	0,0238	0,0149	0,0249	0,0211	0,0065	0,0278	0,021	0,0246	0,0187	0,0086
	019AF06	0,0076	0,0075	0,0039	0,0117	0,0084	0,0217	0,0115	0,0176	0,0267	0,0248	0,0223	0,0278	0,0091	0,0176	0,0098	0,018	0,0075	0,0163	0,0122	0,0181	0,0248	0,0181	0,0303	0,0213	0,0153	0,0235	0,0197	0,0127	0,0255	0,022	0,0235	0,0146	0,0094
	019AF07	0	0,0003	0	0,0004	0,0005	0,0003	0,0004	0,0004	0,0004	0,0008	0,0004	0,0006	0,0003	0,0006	0,0003	0,0003	0,0005	0	0,0004	0,0002	0,0006	0,0003	0,0005	0,0005	0,0001	0,001	0,0152	0,0773	0,0118	0,0003	0,0002	0,0001	0,0002
	019AF08	0,0142	0,0167	0,0117	0,0283	0,0104	0,0006	0,0336	0,0003	0,0001	0,0002	0,0002	0,0003	0,0001	0,0002	0,0003	0,0005	0,0004	0,0048	0,0147	0,004	0,0011	0,0008	0,001	0,0006	0,0004	0,0003	0	0,0002	0,0003	0,0005	0,003	0,0004	0,0004
	019AF09	0,0129	0,0004	0,0212	0,0007	0,0008	0,0004	0,0006	0,0002	0,0003	0,0003	0,0007	0,0002	0,001	0,0004	0	0,0002	0	0	0	0	0,0002	0,0001	0,0002	0,0002	0	0,0003	0	0	0,0002	0,0002	0,0001	0,0002	0
019AR	019AR01	0,9804	0,7981	0,9794	0,9749	0,8636	0,9956	0,9743	0,9955	0,9953	0,9956	0,9955	0,9956	0,9938	0,9947	0,9961	0,995	0,995	0,9894	0,9752	0,9948	0,9958	0,9938	0,9954	0,9947	0,9954	0,9954	0,9901	0,9758	0,9915	0,992	0,9925	0,9868	0,9775
	019AR02	0,0149	0,1974	0,0156	0,0194	0,1312	0,0004	0,0201	0,0002	0,0002	0,0002	0,0002	0,0002	0,0002	0,0002	0,0003	0,0001	0,0002	0,0001	0,0002	0,0004	0,0001	0,0002	0,0002	0,0001	0,0002	0,0002	0,0003	0,0001	0,0002	0,0001	0,0001	0,0002	0,0001
	019AR03	0	0,002	0,0017	0,0025	0,0024	0,0016	0,0023	0,0016	0,0025	0,0014	0,0019	0,0019	0,0021	0,002	0,001	0,0018	0,0021	0,0024	0,0021	0,0019	0,0016	0,0024	0,0014	0,002	0,0023	0,0019	0,0022	0,002	0,0014	0,0049	0,0018	0,0064	0,019
	019AR04	0,0034	0,0014	0,0023	0,0019	0,002	0,0017	0,002	0,0017	0,0011	0,0021	0,0013	0,0017	0,0022	0,002	0,0021	0,0023	0,0019	0,0018	0,002	0,0018	0,0014	0,0021	0,0017	0,0018	0,0014	0,0017	0,0061	0,0211	0,0059	0,0012	0,0017	0,0019	0,0017
	019AR05	0,0013	0,001	0,0011	0,0013	0,0008	0,0007	0,0013	0,0009	0,001	0,0007	0,0011	0,0007	0,0018	0,0012	0,0004	0,0007	0,0009	0,0062	0,0204	0,0011	0,0011	0,0015	0,0013	0,0014	0,0007	0,0008	0,0013	0,0009	0,001	0,0018	0,0039	0,0047	0,0017
019BF	019BF01	0,4158	0,7395	0,3481	0,4269	0,5791	0,5603	0,4755	0,6619	0,6475	0,6138	0,4583	0,6489	0,3438	0,4756	0,6734	0,6473	0,6223	0,6414	0,6279	0,6137	0,6408	0,6437	0,6529	0,6511	0,7176	0,6243	0,6504	0,6444	0,6648	0,6754	0,6768	0,7632	0,6113
	019BF02	0,3348	0,209	0,3383	0,3744	0,2314	0,3611	0,4084	0,3372	0,3504	0,3506	0,359	0,3488	0,4305	0,3769	0,3255	0,3517	0,3754	0,3551	0,3707	0,3846	0,3567	0,3545	0,3453	0,3476	0,2811	0,3422	0,3479	0,3538	0,3334	0,3183	0,3211	0,2316	0,3659
	019BF03	0,2495	0,0515	0,3123	0,1984	0,1895	0,0786	0,1161	0,0009	0,0013	0,0356	0,1827	0,0023	0,2255	0,1472	0,0008	0,0007	0,0022	0,0017	0,0007	0,0013	0,0021	0,0018	0,0011	0,0012	0,0011	0,033	0,0016	0,0011	0,0015	0,0008	0,0013	0,0019	0,0012
	019BF04	0	0	0,0012	0,0003	0	0	0	0	0,0008	0	0	0	0,0002	0,0002	0,0003	0,0002	0,0001	0,0017	0,0007	0,0003	0,0003	0	0,0006	0,0002	0,0002	0,0005	0	0,0008	0,0004	0,0055	0,0007	0,0033	0,0216
019BR	019BR01	0,2723	0,6821	0,2373	0,3782	0,5207	0,5018	0,399	0,5878	0,58	0,544	0,3532	0,5786	0,2574	0,4035	0,62	0,5683	0,5421	0,5712	0,5462	0,5156	0,5604	0,5452	0,5818	0,5718	0,644	0,545	0,5651	0,5568	0,5741	0,5823	0,5917	0,6893	0,5172
	019BR02	0,4383	0,254	0,4144	0,4207	0,2651	0,4202	0,4659	0,3765	0,3812	0,3905	0,4328	0,3815	0,5153	0,4469	0,3597	0,4055	0,4335	0,4045	0,4299	0,4611	0,4002	0,4306	0,3707	0,3978	0,3354	0,3952	0,4031	0,421	0,3789	0,3799	0,3735	0,2956	0,4602
	019BR03	0,2426	0,0531	0,3105	0,1898	0,2022	0,0613	0,1242	0,0126	0,0212	0,0437	0,1983	0,0169	0,2199	0,1407	0,009	0,0131	0,0108	0,0088	0,0103	0,0114	0,0173	0,0103	0,0198	0,0137	0,01	0,0395	0,0174	0,0096	0,0181	0,0156	0,0155	0,0034	0,0101
	019BR04	0,0064	0,0088	0,0047	0,007	0,0059	0,016	0,0075	0,0227	0,0165	0,0218	0,0155	0,0225	0,0069	0,0084	0,0113	0,0119	0,013	0,0156	0,013	0,0111	0,0213	0,0135	0,0266	0,0156	0,0095	0,0201	0,0128	0,0124	0,0282	0,022	0,0189	0,0107	0,0122
	019BR05	0,0404	0,002	0,0331	0,0043	0,0061	0,0006	0,0034	0,0005	0,0012	0	0,0002	0,0005	0,0005	0,0005	0	0,0013	0,0006	0	0,0007	0,0008	0,0009	0,0004	0,001	0,0012	0,001	0,0001	0,0015	0,0003	0,0008	0,0002	0,0004	0,001	0,0003
019CF	019CF01	0,0696	0,1082	0,6176	0,4943	0,3313	0,4288	0,4713	0,4525	0,4381	0,4393	0,4544	0,4296	0,3969	0,4566	0,462	0,4497	0,4441	0,4678	0,46	0,4903	0,4369	0,4463	0,4276	0,45	0,4516	0,43	0,4355	0,4409	0,4234	0,4357	0,44	0,8403	0,4453
	019CF02	0,0363	0,2076	0,1199	0,2536	0,2161	0,4311	0,2552	0,4746	0,4725	0,4462	0,3024	0,4633	0,1867	0,3517	0,5022	0,4794	0,4965	0,4799	0,5014	0,4513	0,4704	0,4839	0,4554	0,4747	0,5012	0,4521	0,4921	0,5228	0,4734	0,4597	0,4611	0,1168	0,4391
	019CF03	0,0131	0,0355	0,0854	0,1767	0,145	0,0947	0,1211	0,0353	0,0466	0,0721	0,2125	0,0533	0,34	0,1557	0,0169	0,0352	0,0292	0,0275	0,0193	0,0293	0,0472	0,0351	0,0589	0,0377	0,0242	0,0746	0,0343	0,0179	0,0536	0,0446	0,0512	0,0122	0,0164

Marker	haplotype	Ⓓ Ador	Ⓒ Acer1.1	① GRE5.6	② CAR2.1	③ POR	① RUN0065	② RUN0067	③ RUN0109	④ RUN0281	⑤ RUN1041	⑥ RUN1050	⑦ RUN1132	⑧ RUN1232	⑨ RUN1578	① MU011I	② MU025I	③ MU040I	④ ROD001	⑤ ROD200	① COM11	② COM12	③ COM18	④ COM28	⑤ COM59	⑥ COM72	⑦ MAY35	① SEY059	② SEY141	③ SEY144	① ISM03	② ISM04	③ MAD26.1	④ MAD30.2
	019CF04	0,8462	0,0907	0,0004	0	0	0	0,0843	0	0	0	0	0,0001	0	0	0	0	0	0	0	0	0	0	0,0002	0	0	0	0	0	0	0	0	0	0
	019CF05	0,0008	0,0031	0,0068	0,0096	0,0055	0,0363	0,0071	0,0356	0,0404	0,0398	0,0289	0,0513	0,0075	0,0247	0,0167	0,034	0,0281	0,0226	0,0173	0,0277	0,0429	0,0326	0,055	0,034	0,0215	0,0408	0,0356	0,0159	0,0481	0,0414	0,0456	0,0122	0,0159
	019CF06	0,0293	0,5533	0,0829	0,0626	0,2991	0,0007	0,0588	0,0007	0,0007	0,0011	0,0005	0,0008	0,0006	0,0007	0,0005	0,0005	0,0006	0,001	0,0005	0,0003	0,0007	0,0007	0,0007	0,0011	0,0004	0,0008	0,001	0,0005	0,0004	0,0005	0,0004	0,0017	0,0005
	019CF07	0	0,0001	0,0004	0,0003	0,0002	0,0004	0,0002	0,0005	0,0007	0,0003	0,0003	0,0005	0,0001	0,0003	0,0009	0,0004	0,0005	0,0005	0,0007	0,0002	0,0005	0,0005	0,0007	0,001	0,0006	0,0004	0,0008	0,001	0,0004	0,0073	0,0007	0,007	0,0319
	019CF08	0	0,0001	0	0,0003	0,0002	0,0003	0,0002	0,0005	0,0005	0,0005	0,0004	0,0006	0,0001	0,0005	0,0004	0,0006	0,0006	0,0004	0,0003	0,0003	0,0007	0,0005	0,0009	0,0009	0,0002	0,0004	0,0004	0,0003	0,0003	0,007	0,0004	0,0063	0,0329
	019CF09	0	0	0	0,0001	0,0001	0,0075	0	0	0	0	0	0	0,0681	0,0097	0	0	0	0	0	0	0	0	0	0	0	0	0	0	0	0	0	0	0
	019CF10	0	0	0,0004	0	0,0002	0,0002	0	0,0001	0,0003	0,0006	0,0002	0,0002	0	0	0,0003	0,0003	0,0002	0,0003	0,0002	0,0003	0,0004	0	0,0004	0,0003	0,0002	0,0008	0,0002	0,0005	0,0003	0,0036	0,0003	0,0035	0,0179
	019CF11	0,0046	0,0013	0,0864	0,0025	0,0023	0	0,0016	0	0	0,0001	0,0003	0,0001	0	0	0,0001	0	0,0001	0	0,0002	0,0002	0,0002	0,0003	0,0003	0,0003	0,0001	0,0002	0	0,0001	0,0002	0,0002	0,0003	0	0,0001
019CR	019CR01	0,1281	0,708	0,7649	0,7308	0,7628	0,5366	0,6541	0,487	0,4871	0,5228	0,665	0,4803	0,7931	0,623	0,4837	0,4841	0,481	0,5019	0,477	0,5236	0,4961	0,4821	0,4876	0,4874	0,4886	0,5134	0,4757	0,45	0,4746	0,4841	0,4796	0,8229	0,4756
	019CR02	0,0523	0,205	0,1433	0,2627	0,2311	0,46	0,2515	0,5106	0,5105	0,4741	0,3328	0,5171	0,204	0,3744	0,514	0,5135	0,5161	0,4937	0,5198	0,4738	0,5012	0,5122	0,5096	0,5065	0,4903	0,4843	0,5162	0,5299	0,5193	0,5057	0,5172	0,1663	0,4891
	019CR03	0,8141	0,0829	0,0008	0,0003	0,0001	0,0008	0,0901	0,0005	0,0005	0,0006	0,0003	0,0008	0,0002	0,0003	0,0004	0,0005	0,0006	0,0004	0,0006	0,0003	0,0005	0,0003	0,0005	0,0005	0,0005	0,0004	0,0005	0,0009	0,0006	0,0006	0,0006	0,0003	0,0005
	019CR04	0	0	0,0003	0,0005	0,0002	0,0005	0,0003	0,0005	0,0003	0,0005	0,0003	0,0005	0,0003	0,0005	0,0002	0,0003	0,0004	0,0005	0,0004	0,0005	0,0004	0,0008	0,0006	0,0009	0,0012	0,0003	0,0003	0,0004	0,0003	0,008	0,0005	0,0091	0,0329
	019CR05	0,0003	0,0017	0,0033	0,002	0,0018	0,0012	0,0017	0,0007	0,001	0,0011	0,0012	0,0007	0,0017	0,0012	0,0013	0,0012	0,0012	0,0009	0,0011	0,001	0,001	0,0009	0,001	0,001	0,001	0,0009	0,0067	0,0183	0,0046	0,0008	0,0009	0,0003	0,0012
	019CR06	0,0051	0,0023	0,0874	0,0034	0,0036	0,0005	0,0022	0,0005	0,0005	0,0005	0,0002	0,0004	0,0006	0,0005	0,0002	0,0002	0,0004	0,0004	0,0004	0,0004	0,0005	0,0007	0,0003	0,0004	0,0005	0,0004	0,0002	0,0002	0,0004	0,0005	0,0006	0	0,0004
	019CR07	0	0	0	0,0003	0,0004	0,0003	0,0002	0,0002	0,0001	0,0005	0,0001	0,0003	0,0001	0	0,0003	0,0002	0,0003	0,0023	0,0007	0,0003	0,0004	0,0029	0,0004	0,0033	0,018	0,0003	0,0003	0,0002	0,0002	0,0003	0,0006	0,001	0,0003
020AF	020AF01	0,3735	0,4305	0,326	0,5213	0,507	0,3093	0,5954	0,3204	0,3648	0,392	0,3699	0,3133	0,0809	0,3371	0,3877	0,4475	0,3951	0,4186	0,5859	0,5247	0,3779	0,4391	0,3789	0,4391	0,4026	0,3722	0,4694	0,7616	0,4334	0,3662	0,3652	0,2973	0,4397
	020AF02	0,0426	0,259	0,033	0,2232	0,226	0,3261	0,2156	0,3595	0,298	0,2817	0,2771	0,3524	0,6124	0,3142	0,3317	0,2409	0,3505	0,3148	0,186	0,2058	0,2456	0,2571	0,2897	0,2516	0,3522	0,3293	0,2383	0,1301	0,2603	0,2989	0,3423	0,5452	0,24
	020AF03	0,1149	0,226	0,1144	0,159	0,1871	0,2294	0,1169	0,2099	0,2115	0,206	0,224	0,2022	0,1271	0,2445	0,2579	0,215	0,1881	0,2186	0,2074	0,2066	0,2175	0,2169	0,2013	0,2086	0,1971	0,2117	0,1923	0,0517	0,1878	0,2278	0,2208	0,0366	0,2221
	020AF04	0,007	0,0241	0,0056	0,0293	0,0234	0,0715	0,023	0,0607	0,0655	0,0604	0,0683	0,0773	0,1011	0,0557	0,0105	0,0497	0,0375	0,0243	0,0109	0,029	0,0584	0,047	0,0708	0,0514	0,0282	0,0482	0,0503	0,0167	0,0629	0,0553	0,0421	0,0197	0,0331
	020AF05	0,0043	0,0182	0,0046	0,0194	0,0136	0,041	0,0155	0,0346	0,0446	0,045	0,0437	0,0352	0,0383	0,0317	0,0087	0,0364	0,0204	0,0172	0,006	0,0195	0,038	0,0303	0,0369	0,038	0,0132	0,025	0,0344	0,0097	0,0355	0,026	0,0178	0,005	0,007
	020AF06	0,4555	0,0359	0,5144	0,039	0,0381	0,0003	0,0271	0,0001	0,0002	0,0005	0,0002	0,0001	0,0002	0,0002	0,0003	0	0,0002	0,0001	0	0,0002	0,0002	0,0004	0,0002	0,0002	0,0001	0,0002	0,0003	0	0,0003	0,0002	0,0001	0	0,0002
	020AF07	0,0014	0,0054	0,0012	0,0067	0,0034	0,0214	0,0045	0,0136	0,0146	0,0135	0,0155	0,0185	0,0388	0,0154	0,0026	0,0095	0,0071	0,0055	0,0021	0,0048	0,0113	0,0071	0,0141	0,009	0,0053	0,0121	0,0101	0,0029	0,0149	0,0129	0,0106	0,0028	0,0031
	020AF08	0	0,0002	0	0,0003	0	0,0003	0,0002	0,0003	0,0002	0,0002	0,0004	0,0002	0,0004	0,0002	0,0002	0,0003	0,0001	0,0001	0,0003	0	0,0001	0,0006	0,0004	0,0004	0,0003	0,0001	0,0001	0	0,0002	0,0107	0,0002	0,0283	0,0522
	020AF09	0,0002	0	0,0004	0,0008	0,0005	0,0003	0,0008	0,0003	0,0001	0	0,0003	0,0003	0	0,0004	0,0002	0,0004	0,0004	0,0002	0,0007	0,0083	0,0504	0,0005	0,0076	0,0007	0,0003	0,0005	0,0009	0,0006	0,0006	0,0004	0,0003	0,0011	0,0004
	020AF10	0,0005	0,0005	0,0003	0,0008	0,0006	0,0005	0,0008	0,0004	0,0002	0,0005	0,0005	0,0003	0,0001	0,0005	0,0003	0,0002	0,0005	0,0005	0,0006	0,0007	0,0003	0,0007	0,0003	0,0008	0,0005	0,0003	0,0037	0,0265	0,0039	0,0003	0,0001	0	0,0004
	020AF11	0	0,0002	0,0002	0,0001	0,0001	0	0,0002	0,0002	0	0,0002	0	0,0003	0,0004	0,0002	0	0	0,0002	0,0001	0,0001	0,0002	0,0002	0,0001	0,0001	0,0001	0,0003	0,0003	0,0001	0	0,0002	0,0014	0,0003	0,0641	0,0017
020AR	020AR01	0,3691	0,3313	0,3517	0,3584	0,3933	0,2569	0,4076	0,2674	0,2915	0,3331	0,3062	0,2821	0,1116	0,2731	0,3288	0,4174	0,3722	0,3859	0,5075	0,4712	0,4029	0,4044	0,3937	0,4204	0,371	0,3591	0,4346	0,5915	0,4131	0,3805	0,3592	0,3475	0,4475
	020AR02	0,0355	0,2135	0,0293	0,1953	0,1998	0,2951	0,1811	0,308	0,2735	0,2419	0,2549	0,2846	0,4609	0,2864	0,2917	0,2301	0,2952	0,2782	0,1619	0,1977	0,2302	0,2389	0,2387	0,236	0,303	0,2673	0,2112	0,1243	0,2166	0,2453	0,2781	0,5217	0,2108
	020AR03	0,5139	0,2823	0,5586	0,1955	0,2439	0,2366	0,14	0,2306	0,2336	0,2354	0,2373	0,2271	0,191	0,2599	0,2902	0,2254	0,1993	0,2271	0,2078	0,2151	0,2282	0,234	0,2186	0,2177	0,1972	0,2219	0,191	0,0501	0,2037	0,2409	0,2303	0,0366	0,2333
	020AR04	0,0606	0,1374	0,0449	0,1813	0,1256	0,1189	0,2133	0,1334	0,1348	0,1247	0,1351	0,1341	0,0487	0,1109	0,0585	0,084	0,088	0,0664	0,098	0,0841	0,0884	0,0798	0,0876	0,0817	0,0852	0,0954	0,1191	0,2138	0,1132	0,0698	0,0816	0,0199	0,046
	020AR05	0,0073	0,0207	0,0052	0,0257	0,0225	0,0538	0,0218	0,034	0,0365	0,0371	0,0382	0,0405	0,1129	0,045	0,0233	0,0283	0,0321	0,0315	0,0195	0,0235	0,0303	0,0296	0,0375	0,0292	0,0308	0,0355	0,0306	0,0134	0,0313	0,0339	0,0328	0,0432	0,022
	020AR06	0,0029	0,0038	0,003	0,0052	0,0032	0,0169	0,0041	0,0107	0,0106	0,0102	0,0124	0,0121	0,0477	0,012	0,0024	0,0063	0,0047	0,0039	0,0024	0,0036	0,0094	0,0057	0,0118	0,0069	0,0042	0,01	0,0053	0,0023	0,01	0,0108	0,0085	0,0039	0,0033
	020AR07	0,001	0,005	0,001	0,0077	0,0038	0,0211	0,0054	0,0151	0,0189	0,0166	0,015	0,0189	0,0258	0,0119	0,0038	0,0079	0,0076	0,0063	0,0023	0,0043	0,0098	0,0067	0,0112	0,0074	0,0076	0,0099	0,0075	0,0041	0,0115	0,01	0,0088	0,0066	0,002
	020AR08	0,0002	0,0007	0,0001	0,0007	0,0005	0,0007	0,0009	0,0006	0,0007	0,0006	0,0007	0,0006	0,0013	0,0008	0,0013	0,0005	0,0007	0,0006	0,0004	0,0004	0,0006	0,0008	0,0007	0,0007	0,0008	0,0004	0,0003	0,0004	0,0005	0,0086	0,0005	0,0202	0,0351
	020AR09	0,0094	0,0053	0,0061	0,0303	0,0074	0	0,0256	0,0001	0	0,0004	0,0001	0	0	0,0001	0	0,0002	0,0001	0,0002	0,0001	0	0,0002	0,0002	0,0002	0	0,0002	0,0004	0,0003	0,0002	0,0002	0,0001	0,0002	0,0003	0,0001
021AF	021AF01	0,096	0,4477	0,4232	0,4153	0,4269	0,3489	0,432	0,3536	0,355	0,3593	0,3406	0,3413	0,4129	0,4193	0,4566	0,3576	0,3643	0,3965	0,4929	0,4057	0,3544	0,3701	0,3161	0,3397	0,3912	0,3635	0,4105	0,6273	0,632	0,3682	0,3605	0,3861	0,4585
	021AF02	0,0787	0,0147	0,0394	0,2056	0,1093	0,1845	0,2079	0,167	0,1676	0,1535	0,1585	0,1498	0,1402	0,2335	0,2198	0,1811	0,2257	0,1899	0,152	0,2163	0,1419	0,1823	0,0348	0,3639	0,2225	0,1563	0,3266	0,1033	0,1246	0,329	0,3368	0,497	0,2024
	021AF03	0,0482	0,3731	0,2434	0,1683	0,3261	0,221	0,1269	0,1933	0,1796	0,1846	0,181	0,1775	0,3832	0,2726	0,2513	0,1886	0,1874	0,1878	0,1993	0,1927	0,2139	0,1773	0,3642	0,0086	0,1928	0,1864	0,0318	0,1572	0,0268	0,0371	0,0179	0,0214	0,1908
	021AF04	0,0168	0,0478	0,0948	0,08	0,0449	0,0918	0,0645	0,105	0,1118	0,1188	0,1208	0,1313	0,0181	0,0247	0,0272	0,1079	0,0874	0,0947	0,0708	0,0641	0,1043	0,1216	0,0922	0,1293	0,0809	0,1169	0,0939	0,0505	0,1033	0,115	0,1249	0,0346	0,0529
	021AF05	0,0188	0,0037	0,0132	0,0455	0,0149	0,0506	0,0387	0,0673	0,0677	0,0751	0,0745	0,0726	0,0106	0,0179	0,0169	0,0662	0,0616	0,0596	0,0372	0,0405	0,0542	0,0723	0,0142	0,1341	0,0564	0,0676	0,1107	0,0324	0,095	0,1204	0,1394	0,0438	0,0352
	021AF06	0,0122	0,0459	0,0796	0,0322	0,0327	0,0334	0,0266	0,0408	0,0429	0,0461	0,0449	0,0499	0,0073	0,0079	0,0104	0,039	0,0293	0,0213	0,0158	0,0317	0,0554	0,0256	0,1023	0,003	0,023	0,0461	0,0025	0,008	0,0021	0,0042	0,0027	0,0027	0,0094
	021AF07	0,3187	0,0054	0	0	0	0	0,0264	0	0	0	0	0	0	0	0	0	0	0	0	0	0	0	0	0	0	0	0	0	0	0	0	0	0
	021AF08	0,0057	0,0264	0,0369	0,0229	0,0222	0,0251	0,0183	0,0295	0,0317	0,0274	0,0333	0,0322	0,0115	0,0106	0,0069	0,0237	0,0176	0,0226	0,0151	0,0181	0,0305	0,0215	0,0344	0,0087	0,0138	0,0262	0,01	0,012	0,0102	0,0091	0,0075	0,0049	0,012
	021AF09	0,003	0,009	0,0179	0,0158	0,0078	0,0239	0,012	0,0248	0,0259	0,018	0,027	0,026	0,0066	0,0071	0,0058	0,0207	0,0163	0,0162	0,0082	0,0183	0,0258	0,0171	0,019	0,0111	0,0115	0,0225	0,0128	0,0039	0,0044	0,01	0,0077	0,0043	0,0076
	021AF10	0,1974	0,0053	0,0008	0,0014	0,0004	0,0067	0,0246	0,0029	0,0041	0,002	0,0042	0,0037	0,0001	0,0003	0,0006	0,0024	0,0015	0,0003	0,0002	0,0009	0,0026	0,0014	0,0006	0,0004	0,0007	0,0026	0	0	0	0,0003	0,0003	0,0001	0,0002
	021AF11	0,1992	0,0028	0,0001	0	0	0	0,0116	0	0	0	0	0	0	0	0	0	0	0	0	0	0	0	0	0	0	0	0	0	0	0	0	0	0
	021AF12	0,0031	0,0163	0,0212	0,0112	0,0128	0,0126	0,0094	0,0154	0,0135	0,0151	0,0148	0,0156	0,006	0,0051	0,0037	0,0124	0,0086	0,0104	0,0082	0,0111	0,0169	0,0105	0,0214	0,0012	0,0068	0,0117	0,0012	0,0053	0,0016	0,0026	0,0023	0,0017	0,005
	021AF13	0	0	0,0003	0,0001	0,0001	0,0004	0	0	0	0	0,0001	0,0002	0,0028	0,0005	0,0003	0,0001	0,0002	0	0,0001	0,0001	0	0	0,0002	0	0,0001	0	0	0	0	0,0041	0	0,0032	0,0257
	021AF14	0,0023	0,0018	0,0291	0,0017	0,0018	0,0012	0,001	0,0003	0	0	0,0003	0,0002	0,0005	0,0003	0,0005	0,0003	0	0,0008	0,0002	0,0004	0,0002	0,0003	0,0005	0	0,0002	0,0002	0	0,0001	0	0	0	0	0,0002
021AR	021AR01	0,1046	0,5124	0,522	0,4818	0,4809	0,4268	0,4651	0,4509	0,4517	0,4509	0,4477	0,4505	0,333	0,4358	0,4714	0,4537	0,4404	0,4872	0,5617	0,4638	0,4413	0,4663	0,4299	0,4431	0,4718	0,4622	0,5172	0,6681	0,7147	0,465	0,4726	0,4298	0,5038
	021AR02	0,0737	0,0396	0,0716	0,26	0,1381	0,2271	0,2756	0,2374	0,2339	0,2241	0,2328	0,2258	0,1509	0,2512	0,2387	0,2447	0,2896	0,2515	0,1941	0,2415	0,2077	0,2687	0,0447	0,4752	0,2734	0,2236	0,3908	0,1272	0,1987	0,4335	0,4332	0,4878	0,2317
	021AR03	0,0456	0,3768	0,3037	0,1972	0,332	0,2597	0,1367	0,2381	0,2239	0,2297	0,222	0,2273	0,3909	0,2808	0,2553	0,225	0,2154	0,2065	0,2069	0,236	0,2619	0,1947	0,4186	0,0087	0,2056	0,2283	0,0347	0,1767	0,0272	0,0311	0,0177	0,0353	0,2352
	021AR04	0,7585	0,0215	0,0001	0,0005	0,0003	0,0005	0,0704	0,0002	0,0002	0,0005	0,0003	0,0004	0	0,0001	0,0002	0,0006	0,0004	0	0,0002	0,0003	0,0003	0,0003	0,0001	0,0007	0,0005	0,0002	0,0006	0,0001	0,0003	0,0007	0,0003	0,0002	0,0002
	021AR05	0,0093	0,026	0,0472	0,0299	0,0233	0,0302	0,027	0,0356	0,0421	0,0464	0,0477	0,0481	0,0052	0,0065	0,0159	0,037	0,0251	0,0269	0,0174	0,0293	0,0445	0,0358	0,0511	0,0344	0,0244	0,0422	0,0286	0,0124	0,0295	0,0343	0,0365	0,0129	0,0132
	021AR06	0,0041	0,0034	0,0049	0,0126	0,006	0,0141	0,0114	0,0165	0,0226	0,0218	0,0227	0,0207	0,0015	0,0028	0,0086	0,0186	0,0146	0,0163	0,012	0,0085	0,0156	0,0186	0,0047	0,0355	0,0127	0,0196	0,0261	0,009	0,0269	0,0317	0,0374	0,0117	0,0087
	021AR07	0,0041	0,02	0,0497	0,0172	0,0187	0,0201	0,0128	0,0207	0,025	0,0262	0,0265	0,0269	0,0066	0,0052	0,0092	0,02	0,0142	0,0109	0,0071	0,0203	0,0284	0,0145	0,0507	0,0017	0,0111	0,0233	0,0016	0,0059	0,0023	0,0017	0,0016	0,0012	0,0059
	021AR08	0	0,0003	0,0007	0,0008	0,0007	0,0215	0,0007	0,0003	0,0003	0,0004	0,0002	0	0,1117	0,0174	0,0005	0,0003	0,0002	0,0005	0,0004	0,0001	0,0002	0,0008	0,0002	0,0004	0,0004	0,0005	0,0003	0,0006	0,0003	0,0005	0,0004	0,0005	0,0005
	021AR09	0,0001	0	0,0001	0,0001	0	0	0,0002	0,0002	0,0002	0,0001	0,0001	0,0002	0,0001	0,0002	0,0002	0,0002	0,0002	0,0002	0,0002	0,0002	0,0001	0,0005	0	0,0003	0	0,0002	0,0001	0	0,0001	0,0015	0,0003	0,0207	0,0009
021BF	021BF01	0,7011	0,4875	0,7331	0,4795	0,5233	0,5297	0,4208	0,5394	0,5417	0,5391	0,5443	0,5291	0,4064	0,5351	0,5167	0,5363	0,4783	0,5525	0,5983	0,5264	0,5355	0,544	0,5271	0,5396	0,5348	0,5276	0,5677	0,5478	0,7252	0,5167	0,5436	0,1841	0,4702
	021BF02	0,2636	0,3713	0,2302	0,4952	0,3795	0,4634	0,5531	0,4579	0,4556	0,4583	0,4532	0,4685	0,5686	0,4583	0,4745	0,4533	0,4531	0,4443	0,3954	0,4702	0,4616	0,4532	0,4702	0,4574	0,4613	0,4696	0,4297	0,4492	0,2717	0,4807	0,4522	0,8144	0,5276
	021BF03	0,0099	0,1091	0,0105	0,0151	0,0723	0,0004	0,0166	0,0002	0,0002	0,0005	0,0004	0,0004	0,0009	0,0004	0,0002	0,0003	0,0006	0,0003	0,0003	0,0006	0,0004	0,0006	0,0004	0,0003	0,0008	0,0003	0,0004	0,0003	0,0002	0,0003	0,0004	0,0007	0,0005
	021BF04	0,0177	0,004	0,0184	0,0039	0,0042	0,001	0,004	0,0009	0,0011	0,0009	0,001	0,0007	0,0005	0,0012	0,001	0,0009	0,0015	0,0015	0,0047	0,0017	0,0012	0,001	0,0007	0,0011	0,0012	0,001	0,0013	0,0016	0,0014	0,001	0,0025	0	0,0008
	021BF05	0,0063	0,027	0,006	0,0047	0,0183	0,0002	0,0045	0,0003	0,0003	0,0003	0,0003	0,0005	0,0004	0,0005	0,0003	0,0003	0,0003	0,0003	0,0004	0,0002	0,0004	0,0002	0,0002	0,0005	0,0006	0,0003	0,0006	0,0004	0,0006	0,0005	0,0006	0	0,0002
	021BF06	0,0007	0,0007	0,0009	0,0007	0,0016	0,0004	0,0002	0,0007	0,0005	0,0006	0,0005	0,0005	0,0005	0,0003	0,007	0,0085	0,0661	0,0006	0,0004	0,0007	0,0004	0,0005	0,0006	0,0006	0,0002	0,0006	0,0002	0,0004	0,0003	0,0003	0,0006	0,0004	0,0005
	021BF07	0,0007	0,0003	0,001	0,0009	0,0007	0,0049	0,0008	0,0005	0,0005	0,0002	0,0002	0,0003	0,0227	0,0042	0,0004	0,0003	0,0001	0,0005	0,0004	0,0001	0,0005	0,0005	0,0008	0,0005	0,0011	0,0005	0	0,0002	0,0005	0,0005	0,0002	0,0004	0,0002
021BR	021BR01	0,7138	0,5289	0,757	0,5033	0,5501	0,5666	0,454	0,5568	0,5738	0,57	0,5724	0,5562	0,4462	0,5649	0,5356	0,5576	0,5524	0,5604	0,5888	0,5475	0,5602	0,5704	0,5492	0,5597	0,5353	0,5489	0,5797	0,5544	0,7376	0,5291	0,5549	0,1474	0,4606

Marker	haplotype	Ⓓ Ador	Ⓒ Acer1.1	① GRE5.6	② CAR2.1	③ POR	① RUN0065	② RUN0067	③ RUN0109	④ RUN0281	⑤ RUN1041	⑥ RUN1050	⑦ RUN1132	⑧ RUN1232	⑨ RUN1578	① MU011I	② MU025I	③ MU040I	④ ROD001	⑤ ROD200	① COM11	② COM12	③ COM18	④ COM28	⑤ COM59	⑥ COM72	⑦ MAY35	① SEY059	② SEY141	③ SEY144	① ISM03	② ISM04	③ MAD26.1	④ MAD30.2
	021BR02	0,2837	0,4693	0,2405	0,4933	0,4476	0,4312	0,5436	0,4414	0,4241	0,4276	0,4259	0,4417	0,5517	0,4326	0,4631	0,4403	0,4458	0,4297	0,3733	0,4509	0,4378	0,4279	0,4486	0,4379	0,4622	0,4487	0,4189	0,4439	0,2605	0,4643	0,4367	0,8442	0,5165
	021BR03	0,0006	0,0007	0,001	0,0012	0,0006	0,0007	0,0006	0,0008	0,0011	0,0011	0,0008	0,0007	0,0007	0,0007	0,0004	0,0007	0,0006	0,005	0,0203	0,0008	0,0009	0,0008	0,0008	0,0007	0,0008	0,0009	0,0004	0,0005	0,0008	0,0008	0,0041	0	0,0005
	021BR04	0,0004	0,0008	0,0005	0,0009	0,0009	0,0009	0,001	0,0004	0,0005	0,0006	0,0005	0,0007	0,0008	0,0005	0,0006	0,0006	0,0006	0,001	0,0007	0	0,0004	0,0004	0,0007	0,0008	0,0007	0,0009	0,0002	0,0006	0,0002	0,0051	0,0006	0,0085	0,022
	021BR05	0,0014	0,0003	0,0009	0,0012	0,0008	0,0006	0,0008	0,0005	0,0006	0,0007	0,0004	0,0007	0,0006	0,0012	0,0003	0,0008	0,0006	0,0039	0,017	0,0006	0,0007	0,0006	0,0007	0,0009	0,0008	0,0007	0,0008	0,0006	0,0008	0,0006	0,0037	0	0,0003
025AF	025AF01	0,4167	0,3377	0,3743	0,5588	0,4432	0,48	0,5968	0,5246	0,5179	0,5245	0,5191	0,5258	0,5139	0,5014	0,5356	0,512	0,5202	0,5206	0,5327	0,5311	0,5163	0,5183	0,5318	0,5138	0,5613	0,5184	0,5041	0,4829	0,5261	0,6021	0,5423	0,3899	0,454
	025AF02	0,4477	0,2377	0,4852	0,2105	0,2394	0,2874	0,1867	0,2403	0,2418	0,2861	0,2834	0,233	0,2395	0,2558	0,2221	0,2387	0,2301	0,2449	0,2131	0,237	0,2423	0,2403	0,238	0,2412	0,234	0,4355	0,2346	0,241	0,2389	0,1946	0,2299	0,316	0,2015
	025AF03	0,1181	0,26	0,1249	0,2152	0,2346	0,2293	0,2032	0,2299	0,2338	0,1853	0,192	0,2359	0,2431	0,2382	0,2397	0,244	0,2453	0,231	0,2522	0,2284	0,2355	0,237	0,2244	0,2397	0,2021	0,0443	0,2564	0,2736	0,2304	0,1959	0,2237	0,2857	0,3041
	025AF04	0,0051	0,0221	0,004	0,0057	0,0121	0,0033	0,0051	0,0052	0,0065	0,0041	0,0055	0,0053	0,0036	0,0046	0,0026	0,0052	0,0044	0,0034	0,0021	0,0035	0,0059	0,0044	0,0058	0,0053	0,0025	0,0017	0,0049	0,0025	0,0046	0,0036	0,004	0,0025	0,0028
	025AF05	0,0125	0,1425	0,0116	0,0098	0,0707	0	0,0081	0	0	0	0	0	0	0	0	0	0	0	0	0	0	0	0	0	0	0	0	0	0	0	0	0,0001	0
	025AF06	0	0	0	0	0	0	0	0	0	0	0	0	0	0	0	0	0	0	0	0	0	0	0	0	0	0	0	0	0	0,0039	0	0,0058	0,0376
025AR	025AR01	0,6876	0,8152	0,6537	0,9734	0,9061	0,9951	0,9744	0,9963	0,9966	0,9961	0,9956	0,9964	0,9947	0,9957	0,9965	0,9961	0,996	0,9944	0,9933	0,9957	0,9962	0,9943	0,9957	0,9933	0,9865	0,9958	0,9956	0,9956	0,9968	0,9889	0,9954	0,7919	0,9837
	025AR02	0,0189	0,1774	0,0193	0,0157	0,0848	0,0027	0,0172	0,0016	0,0014	0,0019	0,0021	0,0016	0,0033	0,0021	0,0018	0,0019	0,0019	0,0026	0,0046	0,002	0,0019	0,0018	0,0016	0,0021	0,002	0,0022	0,0023	0,0024	0,0014	0,002	0,0024	0,001	0,0034
	025AR03	0,2907	0,0055	0,3257	0,0087	0,0075	0,0008	0,0064	0,0005	0,0007	0,0005	0,0006	0,0005	0	0,0007	0,0004	0,0006	0,0009	0,0015	0,0007	0,0007	0,0006	0,0023	0,0013	0,0023	0,0101	0,0005	0,0004	0,0007	0,0004	0,0008	0,0008	0,0006	0,0007
	025AR04	0,0028	0,0019	0,0013	0,0022	0,0016	0,0013	0,002	0,0016	0,0012	0,0016	0,0016	0,0015	0,002	0,0015	0,0013	0,0013	0,0013	0,0015	0,0014	0,0016	0,0013	0,0016	0,0015	0,0022	0,0013	0,0015	0,0018	0,0013	0,0014	0,0082	0,0014	0,2065	0,0123
034AF	034AF01	0,0573	0,6228	0,7908	0,5699	0,6201	0,5741	0,5113	0,5735	0,562	0,5571	0,5589	0,5962	0,7431	0,5759	0,5624	0,5369	0,541	0,5286	0,4842	0,539	0,5545	0,538	0,5773	0,5397	0,5244	0,566	0,5473	0,52	0,5851	0,6019	0,5819	0,4717	0,5482
	034AF02	0,0258	0,3001	0,2086	0,4296	0,3795	0,4252	0,3812	0,4261	0,4375	0,4425	0,4407	0,4031	0,2564	0,4236	0,4372	0,4627	0,4584	0,4708	0,5148	0,4607	0,445	0,4617	0,4218	0,4597	0,4749	0,4332	0,452	0,4794	0,414	0,397	0,4174	0,5278	0,4512
	034AF03	0,9169	0,0771	0,0005	0,0006	0,0005	0,0008	0,1075	0,0005	0,0004	0,0004	0,0004	0,0007	0,0004	0,0005	0,0003	0,0004	0,0006	0,0005	0,0011	0,0003	0,0005	0,0003	0,0009	0,0006	0,0007	0,0008	0,0006	0,0005	0,0009	0,0011	0,0007	0,0005	0,0006
034AR	034AR01	0,0547	0,0483	0,4418	0,4823	0,2774	0,5378	0,4607	0,5318	0,5383	0,5128	0,5155	0,5663	0,6889	0,5383	0,5535	0,5196	0,5166	0,5133	0,4728	0,53	0,5138	0,5164	0,5439	0,5009	0,5172	0,5367	0,5193	0,4967	0,5423	0,5575	0,5366	0,3746	0,5247
	034AR02	0,0326	0,493	0,2287	0,4282	0,4834	0,41	0,3952	0,4175	0,417	0,4398	0,4288	0,3958	0,2519	0,4241	0,4378	0,4499	0,4635	0,4632	0,5098	0,4462	0,4311	0,4506	0,4138	0,4448	0,4644	0,4161	0,4377	0,4812	0,4046	0,3805	0,4107	0,3474	0,4064
	034AR03	0,4621	0,0103	0,0001	0	0	0	0,0233	0	0	0	0	0	0	0	0	0	0	0	0	0	0	0	0,0003	0	0	0,0003	0	0	0	0,0001	0	0	0
	034AR04	0,4346	0,0209	0,0002	0	0	0	0,0449	0	0	0	0	0,0001	0,0001	0	0	0	0	0	0	0	0	0	0	0	0	0,0001	0	0,0002	0,0002	0,0001	0,0001	0	0,0002
	034AR05	0,0091	0,4194	0,1805	0,0621	0,2275	0,0289	0,0516	0,0268	0,0249	0,0232	0,0292	0,0203	0,0327	0,0203	0,0037	0,0148	0,0096	0,0103	0,0065	0,0103	0,0283	0,0164	0,0238	0,0295	0,0075	0,024	0,0208	0,0118	0,0295	0,0219	0,0278	0,0522	0,0165
	034AR06	0,0031	0,0042	0,0087	0,0201	0,0058	0,0213	0,0201	0,0223	0,0186	0,0223	0,0249	0,0163	0,0252	0,0155	0,0035	0,0137	0,0087	0,0117	0,0089	0,0116	0,0253	0,0144	0,0163	0,023	0,0087	0,0209	0,0195	0,0086	0,0218	0,0249	0,0234	0,0617	0,0287
	034AR07	0,0001	0,0022	0,0008	0,0016	0,0016	0,0015	0,0014	0,0014	0,001	0,0019	0,0015	0,001	0,0011	0,0018	0,0015	0,0019	0,0015	0,0014	0,002	0,0017	0,0013	0,0019	0,0017	0,0017	0,0019	0,0019	0,0026	0,0016	0,0013	0,0122	0,0013	0,1408	0,0197
	034AR08	0,0038	0,0008	0,1385	0,0054	0,0033	0	0,0025	0	0	0	0	0	0	0	0	0	0	0	0	0	0	0	0	0	0	0	0	0	0	0	0	0	0
	034AR09	0	0,0008	0,0007	0,0003	0,001	0,0005	0,0002	0	0,0002	0	0	0	0,0001	0	0	0	0	0	0	0,0002	0,0002	0,0003	0,0002	0,0001	0	0	0	0	0,0002	0,0029	0,0001	0,0233	0,0038
036AF	036AF01	0,7554	0,5722	0,7526	0,7286	0,6546	0,7687	0,7341	0,7384	0,743	0,7563	0,7496	0,7418	0,8518	0,7593	0,7389	0,7398	0,7585	0,669	0,5082	0,7511	0,7394	0,7444	0,7502	0,7356	0,7414	0,7493	0,7433	0,7739	0,7578	0,5517	0,6788	0,8324	0,6442
	036AF02	0,2203	0,4267	0,2147	0,2703	0,3439	0,2311	0,2648	0,261	0,2566	0,2428	0,2501	0,2577	0,1481	0,2403	0,2607	0,2597	0,2413	0,3304	0,4906	0,2482	0,2602	0,255	0,2493	0,2638	0,2581	0,2503	0,2562	0,2255	0,2419	0,4477	0,3207	0,1676	0,3548
	036AF03	0,0243	0,0012	0,0327	0,0011	0,0015	0,0002	0,0011	0,0006	0,0004	0,0009	0,0003	0,0004	0,0002	0,0004	0,0004	0,0004	0,0002	0,0006	0,0013	0,0008	0,0004	0,0006	0,0005	0,0006	0,0004	0,0004	0,0005	0,0006	0,0003	0,0006	0,0005	0	0,001
036AR	036AR01	0,4522	0,5023	0,4628	0,4682	0,5125	0,5209	0,4203	0,5168	0,514	0,498	0,5056	0,5153	0,5811	0,5257	0,5117	0,507	0,4857	0,5017	0,5463	0,5002	0,5132	0,5196	0,5104	0,5134	0,5302	0,5038	0,5154	0,4988	0,5084	0,4876	0,5115	0,1593	0,4126
	036AR02	0,5471	0,4967	0,5354	0,5304	0,4865	0,4785	0,5784	0,4827	0,4854	0,5016	0,4936	0,484	0,418	0,4735	0,4874	0,4923	0,5131	0,4974	0,4529	0,4993	0,4862	0,4791	0,4888	0,4859	0,4693	0,4953	0,4805	0,4764	0,4868	0,5076	0,4877	0,8335	0,561
	036AR03	0,0007	0,0006	0,0013	0,0009	0,0005	0,0003	0,0009	0,0002	0,0003	0,0002	0,0004	0,0004	0,0004	0,0005	0,0006	0,0005	0,0006	0,0006	0,0003	0,0003	0,0002	0,0005	0,0004	0,0003	0,0002	0,0003	0,004	0,0246	0,0044	0,0008	0,0004	0,004	0,0017
	036AR04	0	0,0005	0,0004	0,0004	0,0004	0,0002	0,0004	0,0002	0,0004	0,0002	0,0004	0,0004	0,0006	0,0003	0,0003	0,0003	0,0007	0,0004	0,0005	0,0002	0,0004	0,0007	0,0004	0,0004	0,0003	0,0005	0,0002	0,0002	0,0004	0,004	0,0004	0,0032	0,0247
041AF	041AF01	0,7431	0,5212	0,7732	0,5087	0,522	0,5116	0,5048	0,506	0,5113	0,5109	0,5106	0,5086	0,4875	0,4954	0,5247	0,5075	0,4855	0,5022	0,5301	0,4626	0,507	0,4991	0,5028	0,4997	0,4748	0,5214	0,5215	0,6489	0,5232	0,525	0,4971	0,3372	0,5529
	041AF02	0,2338	0,4655	0,2063	0,4584	0,4569	0,4318	0,4676	0,4487	0,4258	0,4291	0,4212	0,4313	0,494	0,4683	0,4552	0,4453	0,4819	0,46	0,4457	0,4977	0,4325	0,4504	0,4277	0,4474	0,487	0,4278	0,4266	0,3211	0,4113	0,4254	0,4513	0,6379	0,4113
	041AF03	0,0156	0,0064	0,012	0,0174	0,0107	0,0292	0,0144	0,0246	0,0325	0,0323	0,0374	0,0307	0,0089	0,0183	0,0107	0,0254	0,0163	0,0191	0,0128	0,02	0,0324	0,0271	0,0383	0,0276	0,0211	0,0271	0,0273	0,0154	0,0346	0,0238	0,0273	0,0077	0,0088
	041AF04	0,0071	0,0063	0,008	0,0145	0,0092	0,0258	0,012	0,02	0,0295	0,0268	0,0302	0,0286	0,0084	0,0169	0,0085	0,0209	0,015	0,0173	0,0102	0,0185	0,0272	0,0224	0,0302	0,0244	0,016	0,0227	0,0237	0,0138	0,03	0,0196	0,0231	0,008	0,0081
	041AF05	0,0004	0,0006	0,0005	0,001	0,0011	0,0015	0,0013	0,0007	0,0009	0,0009	0,0007	0,0008	0,0013	0,001	0,0009	0,0009	0,0014	0,0013	0,0012	0,0013	0,0009	0,0011	0,001	0,0009	0,001	0,001	0,001	0,0007	0,0009	0,0062	0,0011	0,0092	0,0189
041AR	041AR01	0,7848	0,5145	0,8146	0,5055	0,5244	0,4959	0,5045	0,4992	0,5007	0,507	0,4983	0,4976	0,4836	0,4908	0,5101	0,5124	0,4828	0,4894	0,5052	0,4549	0,4923	0,5017	0,4888	0,4953	0,4633	0,5041	0,5144	0,6473	0,509	0,5131	0,4844	0,3341	0,5466
	041AR02	0,1968	0,4717	0,167	0,4614	0,4511	0,447	0,4681	0,4527	0,4311	0,4286	0,429	0,4397	0,4925	0,4681	0,4411	0,2754	0,4448	0,4651	0,4442	0,5005	0,441	0,4496	0,4389	0,4494	0,5008	0,4404	0,4311	0,3162	0,4234	0,4264	0,459	0,6273	0,3739
	041AR03	0,0051	0,0059	0,0062	0,0123	0,0091	0,0223	0,0101	0,0181	0,0266	0,0247	0,0287	0,0252	0,0098	0,0159	0,0062	0,01	0,0115	0,016	0,0107	0,0176	0,0264	0,0199	0,03	0,021	0,0154	0,023	0,0208	0,014	0,0285	0,0189	0,0208	0,006	0,007
	041AR04	0,0081	0,005	0,0073	0,0121	0,0088	0,0219	0,0103	0,0169	0,0256	0,0224	0,0283	0,0234	0,0086	0,0147	0,0072	0,0183	0,012	0,0137	0,0093	0,0164	0,0257	0,0179	0,0266	0,021	0,0126	0,0208	0,0204	0,0141	0,0254	0,0176	0,0194	0,0047	0,0063
	041AR05	0,0047	0,0023	0,0043	0,0078	0,0059	0,0124	0,0063	0,0125	0,0152	0,0171	0,0152	0,0133	0,0049	0,01	0,0042	0,0109	0,0064	0,0099	0,0065	0,0099	0,014	0,0104	0,0148	0,0124	0,0075	0,0112	0,0128	0,0079	0,0132	0,0109	0,0115	0,0032	0,0042
	041AR06	0,0002	0	0	0	0	0	0	0	0	0	0	0	0	0	0,0307	0,1723	0,0421	0	0	0	0	0	0	0	0	0	0	0	0	0	0	0	0
	041AR07	0	0,0002	0,0001	0,0005	0,0003	0	0,0005	0,0002	0,0002	0	0,0003	0,0003	0,0002	0,0002	0,0001	0,0001	0,0002	0,0003	0,0003	0,0003	0,0004	0,0002	0,0005	0,0004	0,0002	0,0002	0	0,0001	0,0002	0,0126	0,0002	0,0239	0,0618
	041AR08	0,0002	0,0004	0,0004	0,0004	0,0005	0,0004	0,0004	0,0005	0,0004	0,0002	0,0003	0,0005	0,0003	0,0003	0,0004	0,0006	0,0002	0,0056	0,0238	0,0005	0,0003	0,0003	0,0005	0,0004	0,0003	0,0004	0,0004	0,0005	0,0003	0,0004	0,0047	0,0007	0,0004
043AF	043AF01	0,0794	0,1827	0,2126	0,2541	0,2287	0,2568	0,2588	0,2434	0,2549	0,2475	0,2572	0,2623	0,4075	0,2587	0,279	0,2615	0,3179	0,2356	0,2587	0,2431	0,2465	0,2464	0,2625	0,2519	0,1972	0,2798	0,2651	0,3287	0,2801	0,2562	0,2582	0,5475	0,245
	043AF02	0,053	0,276	0,1276	0,3501	0,2604	0,2432	0,4019	0,2921	0,2518	0,2429	0,228	0,2623	0,1832	0,2549	0,3041	0,2283	0,1086	0,283	0,3623	0,2845	0,2682	0,2633	0,2364	0,2655	0,3479	0,2325	0,261	0,3331	0,2443	0,2877	0,22	0,0079	0,2733
	043AF03	0,0982	0,1422	0,382	0,1442	0,1767	0,1815	0,1044	0,1768	0,1779	0,1942	0,1834	0,1792	0,1356	0,1881	0,1995	0,1913	0,1915	0,1947	0,1671	0,2073	0,1829	0,1903	0,1818	0,177	0,1559	0,2001	0,1936	0,1744	0,1772	0,1784	0,1873	0,2866	0,2387
	043AF04	0,0645	0,3197	0,228	0,1702	0,2631	0,1626	0,159	0,1674	0,1726	0,1549	0,1573	0,1644	0,2263	0,1925	0,1864	0,1774	0,193	0,1944	0,1484	0,1845	0,161	0,1729	0,1613	0,177	0,2241	0,1583	0,1576	0,1032	0,1577	0,1611	0,2071	0,1161	0,1429
	043AF05	0,0045	0,0147	0,0118	0,0167	0,0144	0,0357	0,014	0,0254	0,0318	0,0393	0,038	0,0327	0,0116	0,0215	0,0036	0,0281	0,0214	0,0217	0,013	0,016	0,0315	0,0272	0,0394	0,0302	0,014	0,0298	0,0247	0,0124	0,0353	0,0242	0,0308	0,0115	0,0076
	043AF06	0,0033	0,0199	0,011	0,0153	0,0178	0,029	0,0095	0,0237	0,0287	0,0295	0,033	0,0212	0,0096	0,0223	0,0055	0,0232	0,0153	0,0238	0,0098	0,0151	0,0236	0,0236	0,0269	0,0229	0,0125	0,0214	0,0302	0,0083	0,0236	0,0175	0,028	0,0044	0,0078
	043AF07	0,003	0,0093	0,0044	0,0146	0,0121	0,0217	0,0117	0,0223	0,0146	0,0243	0,0282	0,0232	0,0071	0,0165	0,004	0,0179	0,006	0,0125	0,0117	0,0148	0,0237	0,0177	0,024	0,0223	0,0158	0,0219	0,0139	0,0116	0,0211	0,0184	0,0188	0,0014	0,0095
	043AF08	0,0053	0,0133	0,008	0,0121	0,0115	0,0261	0,0096	0,0174	0,0263	0,0262	0,0265	0,0193	0,0066	0,0169	0,0027	0,0193	0,0125	0,0122	0,0088	0,0124	0,0228	0,0215	0,0265	0,0209	0,0114	0,019	0,0203	0,0078	0,0214	0,0148	0,0198	0,0033	0,0073
	043AF09	0,0034	0,0049	0,0104	0,0095	0,007	0,0174	0,0085	0,0133	0,017	0,0211	0,0235	0,0174	0,0067	0,0131	0,0036	0,0146	0,0126	0,011	0,0086	0,0104	0,0196	0,0164	0,0205	0,0154	0,0104	0,0191	0,0149	0,0096	0,0189	0,0152	0,0149	0,0038	0,0082
	043AF10	0,0008	0,0074	0,003	0,012	0,0075	0,0241	0,0102	0,0168	0,0232	0,0192	0,0237	0,017	0,0048	0,0141	0,0027	0,0169	0,0049	0,0101	0,0106	0,0111	0,019	0,0187	0,0195	0,0156	0,0096	0,0169	0,0176	0,0102	0,019	0,0146	0,0136	0,0008	0,007
	043AF11	0	0,0005	0,0006	0,0006	0,0006	0,0019	0,0005	0,0011	0,0012	0,0009	0,0009	0,0008	0,0004	0,001	0,0086	0,0211	0,1158	0,0003	0,0007	0,0005	0,0012	0,001	0,0009	0,001	0,0006	0,0008	0,001	0,0005	0,0012	0,0006	0,0011	0	0,0003
	043AF12	0,3917	0,0037	0	0	0	0	0,0045	0	0	0	0	0	0	0	0	0	0	0	0	0	0	0	0	0	0	0	0	0	0	0	0	0	0
	043AF13	0,2928	0,0056	0	0	0	0	0,0072	0	0	0	0	0	0	0	0	0	0	0	0	0	0	0	0	0	0	0	0	0	0	0	0	0	0
	043AF14	0	0,0002	0,0003	0,0003	0,0001	0	0,0001	0,0001	0	0	0,0002	0,0002	0,0003	0,0002	0,0001	0,0002	0,0003	0,0003	0,0003	0,0001	0,0001	0,0008	0,0002	0,0001	0,0004	0,0002	0	0,0002	0,0001	0,0063	0,0003	0,0068	0,0285
	043AF15	0	0	0,0003	0,0002	0,0001	0	0,0001	0,0002	0	0	0,0001	0	0,0002	0,0002	0	0,0002	0,0003	0,0003	0,0002	0,0001	0	0,0003	0,0001	0,0001	0	0,0001	0	0,0001	0,0001	0,0048	0,0001	0,0099	0,0241
043AR	043AR01	0,1161	0,4366	0,6247	0,3413	0,4487	0,3879	0,2844	0,3776	0,4021	0,4118	0,4028	0,3888	0,3901	0,4176	0,4012	0,4102	0,4295	0,4216	0,3342	0,418	0,3877	0,4021	0,4051	0,3985	0,3999	0,4063	0,5938	0,3454	0,602	0,382	0,4385	0,4385	0,4292
	043AR02	0,0437	0,2835	0,1281	0,337	0,2538	0,2325	0,3834	0,2327	0,2222	0,2189	0,2098	0,239	0,1736	0,237	0,2918	0,2197	0,219	0,2664	0,3293	0,2678	0,237	0,2472	0,2251	0,2397	0,3409	0,2267	0,2418	0,3257	0,2351	0,2657	0,2142	0,0197	0,2502
	043AR03	0,0594	0,1828	0,211	0,2431	0,221	0,2439	0,2501	0,2277	0,226	0,2311	0,2324	0,2329	0,4037	0,2501	0,261	0,2504	0,2831	0,2286	0,2486	0,224	0,2235	0,2361	0,2294	0,2271	0,1862	0,2555	0,0497	0,2551	0,0303	0,2354	0,2347	0,526	0,2534
	043AR04	0,0046	0,0368	0,0216	0,0357	0,0365	0,0534	0,0286	0,0712	0,0673	0,0597	0,0697	0,0595	0,0148	0,0417	0,0226	0,0507	0,0312	0,0371	0,0397	0,0385	0,0683	0,0507	0,0615	0,0587	0,0302	0,0478	0,0517	0,0348	0,059	0,0462	0,05	0,0101	0,0195
	043AR05	0,0024	0,0299	0,0063	0,0274	0,0277	0,0592	0,0204	0,0582	0,0524	0,0528	0,056	0,0518	0,0091	0,0367	0,0155	0,0439	0,023	0,0303	0,0283	0,0339	0,0546	0,0403	0,0533	0,0479	0,0259	0,0427	0,0612	0,0262	0,0723	0,0429	0,0435	0,0031	0,0151

		Ⓓ	Ⓒ	①	②	③	①	②	③	④	⑤	⑥	⑦	⑧	⑨	①	②	③	④	⑤	①	②	③	④	⑤	⑥	⑦	①	②	③	①	②	③	④
Marker	haplotype	Ador	Acer1.1	GRE5.6	CAR2.1	POR	RUN0065	RUN0067	RUN0109	RUN0281	RUN1041	RUN1050	RUN1132	RUN1232	RUN1578	MU011I	MU025I	MU040I	ROD001	ROD200	COM11	COM12	COM18	COM28	COM59	COM72	MAY35	SEY059	SEY141	SEY144	ISM03	ISM04	MAD26.1	MAD30.2
	043AR06	0,0029	0,0121	0,0084	0,0152	0,0122	0,0231	0,0142	0,0324	0,0298	0,0256	0,0291	0,028	0,0087	0,0169	0,0079	0,025	0,014	0,0161	0,0197	0,0177	0,0288	0,0223	0,0253	0,0273	0,0122	0,0208	0,0016	0,0125	0,0012	0,022	0,019	0,0017	0,0082
	043AR07	0,5833	0,015	0	0	0	0	0,0149	0	0	0	0	0	0	0	0	0	0	0	0	0	0	0	0	0	0	0	0	0	0	0	0	0	0
	043AR08	0	0	0	0,0003	0,0001	0	0,0002	0,0001	0,0002	0,0001	0,0001	0,0001	0	0	0	0,0002	0,0002	0	0,0002	0,0001	0,0001	0,0012	0,0002	0,0007	0,0046	0,0001	0,0001	0,0002	0,0001	0,0059	0	0,001	0,0245
	043AR09	0,1876	0,0031	0	0	0	0	0,0037	0	0	0	0	0	0	0	0	0	0	0	0	0	0	0	0	0	0	0	0	0	0	0	0	0	0
049AF	049AF01	0,3941	0,398	0,387	0,4104	0,3872	0,3889	0,3933	0,3873	0,3855	0,3916	0,402	0,4219	0,3851	0,435	0,4758	0,4365	0,5308	0,4289	0,5197	0,3906	0,4134	0,4119	0,3957	0,4173	0,4541	0,4235	0,4354	0,4482	0,4128	0,452	0,4437	0,338	0,4102
	049AF02	0,2585	0,4128	0,2557	0,3927	0,4321	0,3778	0,3866	0,4035	0,385	0,4052	0,3969	0,4097	0,2757	0,4469	0,4388	0,4371	0,4112	0,4244	0,4278	0,4661	0,4539	0,4132	0,4262	0,4527	0,4963	0,4173	0,4466	0,4698	0,4053	0,4302	0,4209	0,5548	0,544
	049AF03	0,1826	0,0938	0,1938	0,0999	0,0917	0,1165	0,1125	0,1076	0,1146	0,1065	0,103	0,0862	0,158	0,0575	0,0404	0,0657	0,0276	0,0717	0,026	0,0717	0,0699	0,0887	0,0919	0,0661	0,0246	0,0813	0,0583	0,0425	0,0918	0,0587	0,069	0,0397	0,0204
	049AF04	0,1145	0,0899	0,1079	0,0881	0,0804	0,1145	0,1031	0,1013	0,1144	0,0961	0,0976	0,0818	0,1721	0,0586	0,0451	0,0601	0,03	0,0738	0,0259	0,0705	0,0624	0,0856	0,0855	0,0633	0,0244	0,0775	0,0588	0,0391	0,0897	0,0575	0,066	0,0398	0,0229
	049AF05	0,0331	0,0026	0,0369	0,0054	0,0053	0,0009	0,0028	0,0001	0	0,0001	0,0002	0,0001	0,0052	0,0008	0	0,0002	0,0002	0,0003	0,0001	0	0	0,0003	0,0002	0,0004	0,0004	0,0001	0,0001	0	0,0002	0,0002	0	0,0003	0,0003
	049AF06	0,0172	0,0028	0,0184	0,0032	0,003	0,0011	0,0013	0	0	0	0	0	0,0037	0,0006	0	0	0	0	0	0	0	0	0	0	0	0	0,0003	0,0001	0	0	0	0,0001	0
	049AF07	0	0,0001	0,0002	0,0003	0,0002	0,0003	0,0003	0,0002	0,0004	0,0005	0,0002	0,0003	0,0002	0,0005	0	0,0002	0,0003	0,0008	0,0005	0,001	0,0003	0,0003	0,0004	0,0002	0,0001	0,0003	0,0005	0,0004	0,0003	0,0013	0,0003	0,0273	0,0022
049AR	049AR01	0,564	0,4542	0,56	0,5074	0,4488	0,4759	0,5	0,4844	0,4774	0,4726	0,4952	0,5051	0,5349	0,5114	0,5237	0,5053	0,5796	0,4737	0,5507	0,4503	0,4845	0,4643	0,4743	0,4878	0,4965	0,5046	0,5	0,4999	0,4996	0,5164	0,5073	0,3723	0,4537
	049AR02	0,3376	0,4344	0,3311	0,4406	0,4301	0,4374	0,4501	0,4821	0,4449	0,4508	0,4693	0,4677	0,3836	0,4661	0,4421	0,4747	0,411	0,4284	0,4276	0,4908	0,4939	0,4471	0,4943	0,4873	0,4887	0,4699	0,4668	0,4827	0,4713	0,4577	0,4678	0,5294	0,4826
	049AR03	0,0332	0,0208	0,0357	0,0177	0,0181	0,0246	0,0216	0,0152	0,0217	0,0185	0,0152	0,012	0,0411	0,0116	0,0093	0,0099	0,0049	0,0134	0,0049	0,0118	0,0097	0,0148	0,0133	0,0095	0,0044	0,0109	0,0088	0,0076	0,0138	0,0088	0,01	0,0067	0,005
	049AR04	0,0313	0,0176	0,0317	0,0167	0,0146	0,0233	0,0188	0,0132	0,0185	0,0139	0,0152	0,0115	0,0382	0,0079	0,0073	0,0074	0,0021	0,0124	0,0071	0,0096	0,0074	0,0125	0,0133	0,0083	0,0033	0,0102	0,0098	0,0065	0,0133	0,0076	0,0091	0,0072	0,0032
	049AR05	0,0033	0,0379	0,0067	0,0061	0,0398	0,0198	0,0031	0,0022	0,0187	0,0216	0,0025	0,0017	0,001	0,0013	0,0091	0,001	0,001	0,0377	0,0051	0,0187	0,0015	0,0317	0,0019	0,0033	0,003	0,0019	0,0084	0,0016	0,0007	0,0017	0,0026	0,0298	0,0025
	049AR06	0,0036	0,0325	0,0051	0,0066	0,0434	0,0182	0,0031	0,0019	0,0182	0,0219	0,0017	0,0013	0,0008	0,0013	0,0081	0,001	0,0007	0,0338	0,0041	0,0183	0,0024	0,0288	0,002	0,003	0,0033	0,002	0,0056	0,0015	0,0007	0,0023	0,0023	0,046	0,0043
	049AR07	0,0267	0,0025	0,0294	0,0047	0,005	0,0007	0,0027	0,0005	0,0004	0,0004	0,0005	0,0004	0,0003	0,0003	0,0003	0,0004	0,0005	0,0003	0,0004	0,0005	0,0005	0,0003	0,0006	0,0006	0,0004	0,0003	0,0003	0,0002	0,0003	0,0005	0,0005	0,0002	0,0004
	049AR08	0,0002	0	0,0002	0,0003	0,0003	0,0002	0,0006	0,0004	0,0001	0,0004	0,0003	0,0003	0,0001	0,0001	0	0,0002	0,0002	0,0002	0,0001	0	0,0002	0,0004	0,0004	0,0003	0,0002	0,0002	0,0002	0,0001	0,0002	0,005	0,0004	0,0085	0,0483
052AF	052AF01	0,4311	0,3985	0,4193	0,3763	0,4289	0,451	0,3969	0,5059	0,4738	0,5177	0,5064	0,613	0,3484	0,3357	0,4309	0,3324	0,5178	0,3711	0,4275	0,4114	0,4472	0,4526	0,7319	0,4008	0,367	0,6675	0,6075	0,2658	0,632	0,5406	0,6364	0,529	0,4649
	052AF02	0,3202	0,2895	0,326	0,3685	0,3303	0,2987	0,369	0,2885	0,3117	0,285	0,2645	0,2512	0,3499	0,3566	0,2791	0,2819	0,2739	0,3278	0,2636	0,3478	0,3687	0,3451	0,1751	0,3376	0,3661	0,2062	0,2271	0,448	0,2249	0,2967	0,2351	0,2896	0,3329
	052AF03	0,0453	0,0171	0,045	0,0551	0,0186	0,056	0,0539	0,0458	0,04	0,0448	0,0512	0,0269	0,089	0,0861	0,0939	0,1287	0,0527	0,0899	0,1253	0,0741	0,0363	0,0284	0,0305	0,0567	0,0659	0,0305	0,0401	0,0936	0,0333	0,0424	0,0352	0,081	0,0636
	052AF04	0,0328	0,0988	0,0323	0,0581	0,0701	0,0519	0,0588	0,0218	0,0301	0,0379	0,0382	0,021	0,0547	0,0586	0,0754	0,0939	0,0446	0,058	0,0598	0,0501	0,0296	0,051	0,017	0,0476	0,0448	0,0222	0,0282	0,0348	0,0184	0,0238	0,0239	0,016	0,0187
	052AF05	0,0217	0,0392	0,0218	0,0471	0,0341	0,0547	0,0374	0,0492	0,0561	0,0556	0,0631	0,0315	0,0346	0,0652	0,0373	0,0948	0,0322	0,0632	0,05	0,0211	0,0512	0,0462	0,0182	0,0688	0,0419	0,0297	0,0193	0,0279	0,0255	0,0364	0,0254	0,0284	0,0354
	052AF06	0,0846	0,0446	0,0876	0,0339	0,0397	0,0335	0,0249	0,0378	0,0356	0,0138	0,0248	0,0206	0,061	0,0336	0,0506	0,0035	0,0441	0,0364	0,0289	0,0624	0,0197	0,0367	0,0143	0,0307	0,067	0,012	0,0529	0,0754	0,0333	0,0158	0,0136	0,0165	0,0361
	052AF07	0,0296	0,0249	0,0224	0,0338	0,0269	0,0238	0,0375	0,0222	0,0242	0,0172	0,0205	0,0156	0,0287	0,0292	0,0147	0,0176	0,017	0,0224	0,0204	0,0218	0,0222	0,0211	0,01	0,0246	0,0255	0,0137	0,016	0,0358	0,0163	0,0196	0,0135	0,0205	0,0249
	052AF08	0,0287	0,0088	0,0347	0,0227	0,0112	0,0272	0,0194	0,0289	0,0286	0,0278	0,0311	0,0199	0,0162	0,0307	0,0178	0,0467	0,0158	0,0307	0,024	0,0112	0,025	0,0183	0,0116	0,0327	0,0216	0,0179	0,0089	0,0187	0,0159	0,0242	0,0164	0,0188	0,0232
	052AF09	0,0042	0,0577	0,0082	0,0033	0,0294	0	0,0012	0	0	0	0	0	0	0	0	0	0	0	0	0	0	0	0	0	0	0	0	0	0	0	0	0	0
	052AF10	0,0005	0,0005	0,0006	0,0003	0,0014	0,0031	0,0004	0	0	0,0003	0,0004	0,0003	0,0175	0,0044	0,0004	0,0006	0,0018	0,0004	0,0004	0	0,0002	0,0005	0,0001	0,0004	0,0001	0,0004	0	0	0,0004	0,0005	0,0005	0	0,0004
	052AF11	0,0014	0,0204	0,0021	0,0008	0,0094	0	0,0006	0	0	0	0	0	0	0	0	0	0	0	0	0	0	0	0	0	0	0	0	0	0	0	0	0	0
052AR	052AR01	0,362	0,3286	0,3748	0,3469	0,3712	0,4061	0,3566	0,471	0,4254	0,461	0,4633	0,589	0,3298	0,2982	0,3817	0,2954	0,5004	0,3341	0,3666	0,3648	0,4158	0,3263	0,6944	0,3473	0,3201	0,6427	0,5455	0,2472	0,6009	0,5118	0,6043	0,4972	0,4162
	052AR02	0,1525	0,2566	0,137	0,2762	0,2368	0,2391	0,2509	0,1829	0,2462	0,2253	0,1947	0,1589	0,2959	0,2576	0,3364	0,4573	0,2294	0,3389	0,3341	0,2865	0,2097	0,2603	0,1057	0,3099	0,2677	0,1524	0,1613	0,3019	0,1627	0,1931	0,1539	0,1752	0,1836
	052AR03	0,218	0,1255	0,2342	0,1368	0,1374	0,1606	0,1397	0,1307	0,119	0,1568	0,1713	0,1269	0,1661	0,1451	0,147	0,1134	0,1554	0,1652	0,0883	0,1782	0,2048	0,2512	0,1249	0,1849	0,1761	0,1279	0,1586	0,1603	0,1259	0,1757	0,1393	0,1937	0,2328
	052AR04	0,0588	0,0367	0,0369	0,1104	0,0587	0,0587	0,1388	0,0721	0,0481	0,0527	0,0913	0,0384	0,0555	0,123	0,0006	0,0005	0,001	0,0062	0,0075	0,0567	0,0934	0,0105	0,0294	0,0498	0,0531	0,0331	0,0218	0,0103	0,0205	0,0673	0,0344	0,0832	0,0804
	052AR05	0,1076	0,0582	0,1205	0,0412	0,0682	0,0475	0,0358	0,051	0,0387	0,0095	0,0407	0,0274	0,0573	0,0465	0	0	0	0,0025	0,0029	0,0558	0,0331	0,0426	0,0211	0,0268	0,0648	0,0223	0,0511	0,1014	0,0415	0,0274	0,013	0,0256	0,0507
	052AR06	0,041	0,0075	0,0417	0,0392	0,0099	0,0374	0,0365	0,0497	0,0699	0,0383	0,0031	0,031	0,0489	0,0722	0,0536	0,0761	0,0493	0,066	0,1156	0	0,0002	0,001	0,0001	0,0047	0,0497	0,0029	0,0235	0,1502	0,023	0	0,0248	0,0002	0,0001
	052AR07	0,0058	0,0022	0,0061	0,0093	0,0021	0,0176	0,0086	0,0167	0,014	0,027	0,0159	0,005	0,0132	0,0391	0,015	0,0377	0,0089	0,035	0,0355	0,0243	0,0219	0,0108	0,008	0,0378	0,0241	0,0079	0,0155	0,0185	0,0076	0,0122	0,0128	0,0189	0,0287
	052AR08	0,0043	0,004	0,0046	0,0093	0,002	0,0144	0,0074	0,0041	0,0179	0,0108	0,002	0,0111	0,0278	0,0046	0,0611	0,0087	0,0496	0,0448	0,0436	0,0267	0,0049	0,0028	0,0089	0,0211	0,0266	0,0009	0,0187	0,0049	0,0118	0,0001	0,0095	0	0
	052AR09	0,0058	0,0028	0,0031	0,0083	0,0061	0,0107	0,0064	0,0199	0,0185	0,0153	0,0152	0,01	0,0041	0,0117	0,0032	0,0086	0,0045	0,0056	0,0041	0,0048	0,0132	0,0014	0,0052	0,0072	0,0055	0,007	0,0027	0,0043	0,0037	0,0096	0,0064	0,0047	0,0055
	052AR10	0,0112	0,0744	0,0099	0,0104	0,0423	0,0069	0,0088	0,0013	0,0016	0,0018	0,0021	0,0012	0,0006	0,0014	0,0008	0,0018	0,001	0,0008	0,0012	0,0009	0,0021	0,0014	0,0012	0,0017	0,0009	0,0017	0,0009	0,0009	0,0015	0,002	0,0009	0,0008	0,0011
	052AR11	0,0081	0,067	0,0102	0,0065	0,0421	0	0,006	0	0	0	0	0	0	0	0	0	0	0	0	0	0	0,0003	0	0	0	0	0	0	0	0	0	0	0
	052AR12	0,0004	0,0003	0,0003	0,0003	0,0004	0,0011	0,0003	0,0005	0,0007	0,0009	0,0005	0,0008	0,0005	0,0003	0,0004	0,0004	0,0005	0,0006	0,0005	0,0012	0,0008	0,0908	0,0012	0,0083	0,0105	0,001	0,0004	0	0,0008	0,0006	0,0004	0,0004	0,0007
	052AR13	0,0035	0,0349	0,0046	0,0029	0,0216	0	0,0032	0	0	0	0	0	0	0	0	0	0	0	0	0	0	0	0	0	0	0	0	0	0	0	0	0	0
	052AR14	0,0209	0,0012	0,0163	0,002	0,0012	0	0,0009	0	0	0,0005	0	0,0002	0,0002	0,0003	0	0,0001	0	0,0003	0,0001	0	0,0002	0,0003	0	0,0005	0,0009	0,0002	0	0,0002	0	0	0	0,0002	0,0001
105AF	105AF01	0,3456	0,3885	0,2932	0,3991	0,3773	0,3819	0,4248	0,3879	0,3914	0,4021	0,3866	0,4345	0,2445	0,3852	0,7488	0,4181	0,5203	0,3492	0,122	0,3636	0,4388	0,3881	0,4351	0,3918	0,3344	0,4173	0,4292	0,5255	0,4645	0,7984	0,7125	0,9236	0,692
	105AF02	0,3243	0,5385	0,3382	0,3256	0,4811	0,3259	0,3153	0,3236	0,3225	0,3123	0,3134	0,3146	0,3828	0,338	0,0929	0,3076	0,2906	0,3266	0,3563	0,3136	0,2741	0,3001	0,2893	0,3077	0,256	0,3114	0,2937	0,2738	0,3127	0,018	0,0539	0,022	0,1125
	105AF03	0,1092	0,0132	0,1059	0,1358	0,0501	0,1318	0,1475	0,1226	0,1166	0,1173	0,1169	0,1044	0,2054	0,1288	0,0325	0,1062	0,0642	0,1377	0,2331	0,1356	0,111	0,1378	0,103	0,1274	0,2162	0,1109	0,1168	0,063	0,0831	0,0777	0,0995	0,0188	0,1056
	105AF04	0,0452	0,0096	0,0449	0,0551	0,0306	0,057	0,0441	0,071	0,0591	0,0523	0,0564	0,0544	0,0891	0,0628	0,0806	0,0654	0,0632	0,1049	0,1911	0,0912	0,0585	0,0777	0,0498	0,0699	0,1073	0,0548	0,0666	0,0798	0,0471	0,0363	0,0642	0,0073	0,0351
	105AF05	0,0074	0,0017	0,0061	0,012	0,0061	0,013	0,0103	0,0121	0,0116	0,0116	0,0126	0,0112	0,0191	0,0136	0,0137	0,0135	0,0159	0,0195	0,0346	0,017	0,0115	0,0141	0,0106	0,0131	0,0208	0,011	0,0129	0,0137	0,0101	0,0061	0,0108	0,001	0,0074
	105AF06	0,0099	0,0054	0,0099	0,0123	0,0089	0,0183	0,0094	0,0166	0,019	0,0236	0,0251	0,0174	0,0113	0,0128	0,0055	0,0158	0,0084	0,0081	0,0035	0,0136	0,0185	0,0174	0,0229	0,018	0,012	0,0219	0,0153	0,0066	0,0176	0,0011	0,0017	0,0021	0,002
	105AF07	0,0107	0,0015	0,0122	0,0136	0,0054	0,011	0,0145	0,009	0,0088	0,0097	0,0091	0,0086	0,0211	0,0129	0,0025	0,0099	0,0052	0,0198	0,0355	0,0136	0,011	0,0135	0,0091	0,0102	0,0206	0,0091	0,0113	0,0122	0,0094	0,0078	0,0115	0,001	0,0138
	105AF08	0,0066	0,0035	0,0046	0,0085	0,0048	0,0117	0,0065	0,0113	0,0159	0,0201	0,0184	0,0148	0,0065	0,0104	0,0076	0,0151	0,0087	0,0083	0,0057	0,0137	0,0211	0,0118	0,0247	0,0139	0,0081	0,0183	0,0141	0,0067	0,0174	0,0239	0,0183	0,0136	0,0149
	105AF09	0,0057	0,0037	0,003	0,0098	0,0051	0,0138	0,0079	0,0144	0,0164	0,0142	0,0213	0,0156	0,0044	0,0105	0,0056	0,0146	0,0084	0,0073	0,0025	0,0114	0,0207	0,0131	0,0185	0,0148	0,0067	0,014	0,0141	0,0062	0,0129	0,0187	0,0172	0,0052	0,0076
	105AF10	0,0041	0,0026	0,0076	0,0104	0,0052	0,016	0,0076	0,0146	0,0192	0,0158	0,0182	0,0114	0,0067	0,0116	0,0039	0,0156	0,006	0,0061	0,0025	0,0132	0,0154	0,0096	0,0181	0,0148	0,0092	0,0148	0,0117	0,0036	0,012	0,0083	0,0057	0,0052	0,0056
	105AF11	0,0049	0,0048	0,0076	0,0106	0,007	0,0177	0,0074	0,015	0,0169	0,0186	0,0194	0,0105	0,0064	0,0107	0,0033	0,0157	0,0071	0,0079	0,0053	0,0089	0,0166	0,0136	0,0162	0,0153	0,0058	0,0138	0,0114	0,0046	0,0107	0,0004	0,001	0	0,0008
	105AF12	0,0025	0,0237	0,0023	0,0022	0,0144	0,0007	0,0017	0,0011	0,0008	0,0007	0,0007	0,0006	0,0022	0,0015	0,0024	0,0009	0,0013	0,0038	0,0077	0,0037	0,0009	0,002	0,0009	0,0014	0,0024	0,0012	0,0012	0,0029	0,001	0,0011	0,0013	0	0,0011
	105AF13	0,124	0,0033	0,1645	0,0051	0,0041	0,0013	0,003	0,0009	0,0018	0,0016	0,002	0,0019	0,0005	0,0011	0,0007	0,0015	0,0008	0,0009	0,0002	0,0009	0,0019	0,0011	0,0016	0,0017	0,0006	0,0017	0,0015	0,0014	0,0015	0,0024	0,0025	0	0,0017
105AR	105AR01	0,4188	0,307	0,392	0,3392	0,2863	0,2981	0,3705	0,3161	0,3159	0,3263	0,3092	0,3435	0,1965	0,3229	0,7339	0,3507	0,4436	0,2948	0,0996	0,308	0,3542	0,3146	0,336	0,3154	0,2906	0,3296	0,3621	0,4562	0,3788	0,7168	0,6235	0,5714	0,6322
	105AR02	0,3373	0,5629	0,341	0,3291	0,5122	0,3017	0,3243	0,3105	0,2857	0,2797	0,2732	0,2975	0,3578	0,3215	0,0635	0,2791	0,2663	0,3361	0,3731	0,3196	0,2517	0,2886	0,2682	0,2922	0,2361	0,2779	0,2653	0,2873	0,2795	0,0172	0,067	0,0729	0,112
	105AR03	0,1831	0,0661	0,179	0,2299	0,1205	0,235	0,2209	0,232	0,2281	0,2245	0,2334	0,2337	0,3574	0,2359	0,1592	0,2197	0,1902	0,2993	0,5001	0,1648	0,198	0,2671	0,1574	0,2474	0,3848	0,2338	0,237	0,1875	0,2009	0,1992	0,2437	0,341	0,2028
	105AR04	0,0158	0,0216	0,0231	0,0294	0,0246	0,0525	0,0259	0,046	0,0517	0,049	0,0544	0,0385	0,0288	0,038	0,0137	0,042	0,0309	0,0159	0,0053	0,0277	0,041	0,0341	0,0461	0,0423	0,0233	0,0465	0,0385	0,0228	0,0414	0,0025	0,0041	0,0011	0,007
	105AR05	0,0152	0,0073	0,0226	0,022	0,0146	0,0406	0,0188	0,0295	0,0387	0,0336	0,0443	0,0325	0,0224	0,0268	0,0141	0,0346	0,0268	0,0222	0,0089	0,0196	0,0455	0,0344	0,0359	0,0351	0,0245	0,042	0,0369	0,0157	0,0379	0,0395	0,0358	0,0065	0,0229
	105AR06	0,0109	0,0232	0,0131	0,019	0,0228	0,0299	0,0161	0,0267	0,0306	0,0297	0,0299	0,0205	0,0104	0,022	0,006	0,0284	0,0177	0,0072	0,0013	0,0161	0,0266	0,0194	0,026	0,0227	0,0107	0,024	0,0189	0,0134	0,023	0,0011	0,0017	0,0011	0,0059
	105AR07	0,0071	0,0026	0,0131	0,0096	0,0048	0,0164	0,0076	0,0139	0,0176	0,019	0,0191	0,013	0,0073	0,011	0,0048	0,016	0,0105	0,0119	0,0046	0,0108	0,0226	0,0182	0,0213	0,0154	0,0109	0,0176	0,015	0,0063	0,0152	0,0232	0,0227	0,0053	0,0136
	105AR08	0,006	0,0044	0,0078	0,012	0,0058	0,0138	0,009	0,0155	0,0186	0,0229	0,0182	0,0117	0,0103	0,013	0,0031	0,0161	0,0069	0,0056	0,0038	0,0098	0,0113	0,0098	0,0119	0,0156	0,011	0,0161	0,0138	0,0055	0,0116	0,0002	0,0007	0,0001	0,0018
	105AR09	0,0054	0,0038	0,007	0,0083	0,0055	0,0119	0,0065	0,0095	0,0127	0,0154	0,0179	0,0089	0,0087	0,0088	0,0015	0,0132	0,0073	0,0058	0,0026	0,008	0,0145	0,0136	0,0138	0,0133	0,0077	0,0121	0,0124	0,0053	0,0117	0,0004	0,0005	0,0004	0,0018

Marker	haplotype	Ⓓ Ador	Ⓒ Acer1.1	① GRE5.6	② CAR2.1	③ POR	① RUN0065	② RUN0067	③ RUN0109	④ RUN0281	⑤ RUN1041	⑥ RUN1050	⑦ RUN1132	⑧ RUN1232	⑨ RUN1578	① MU011I	② MU025I	③ MU040I	④ ROD001	⑤ ROD200	① COM11	② COM12	③ COM18	④ COM28	⑤ COM59	⑥ COM72	⑦ MAY35	① SEY059	② SEY141	③ SEY144	① ISM03	② ISM04	③ MAD26.1	④ MAD30.2
	105AR10	0,0005	0,001	0,0013	0,0015	0,0029	0	0,0003	0,0002	0,0003	0	0,0003	0,0002	0,0005	0	0,0002	0	0	0,0012	0,0006	0,1156	0,0346	0,0002	0,0834	0,0006	0,0003	0,0003	0,0002	0	0,0001	0	0,0002	0,0001	0,0002
143AF	143AF01	0,8	0,8157	0,8315	0,7141	0,7672	0,7057	0,7513	0,6471	0,7001	0,6805	0,6785	0,6764	0,6915	0,6483	0,7384	0,3309	0,7202	0,7025	0,63	0,6976	0,6806	0,7232	0,6983	0,7071	0,7302	0,6674	0,7062	0,6029	0,6736	0,7333	0,6793	0,8258	0,7768
	143AF02	0,0542	0,0482	0,0353	0,1057	0,068	0,1076	0,0938	0,1481	0,124	0,1236	0,1251	0,1343	0,0951	0,123	0,0215	0,062	0,0226	0,0596	0,0537	0,1169	0,1243	0,1094	0,1223	0,1094	0,0971	0,1303	0,0904	0,1023	0,1141	0,0935	0,0917	0,0497	0,0604
	143AF03	0,0583	0,0596	0,0679	0,0525	0,0559	0,0363	0,0614	0,0356	0,0344	0,0475	0,0428	0,0374	0,0596	0,0469	0,0488	0,0277	0,0557	0,0507	0,058	0,0539	0,0446	0,0468	0,0376	0,0482	0,066	0,0447	0,0433	0,0463	0,0359	0,0535	0,0416	0,06	0,0637
	143AF04	0,025	0,0241	0,019	0,0414	0,0334	0,0326	0,03	0,0366	0,0348	0,0397	0,0399	0,0311	0,0395	0,0468	0,0131	0,0264	0,0197	0,0286	0,0273	0,0397	0,0368	0,0374	0,0334	0,039	0,0364	0,039	0,0372	0,0412	0,0396	0,0352	0,0314	0,0254	0,0297
	143AF05	0,0042	0,0076	0,0245	0,02	0,0178	0,0284	0,0114	0,0403	0,0274	0,0403	0,0377	0,0334	0,0345	0,0384	0,0225	0,0603	0,0232	0,036	0,0525	0,0302	0,0391	0,0246	0,0328	0,0339	0,0255	0,0351	0,0391	0,0415	0,0425	0,0238	0,0356	0,0104	0,0142
	143AF06	0,025	0,0171	0,0163	0,0296	0,021	0,0363	0,0229	0,0593	0,0552	0,0265	0,0362	0,0534	0,0322	0,038	0,0374	0,0984	0,0426	0,0512	0,0753	0,0119	0,0147	0,0111	0,0144	0,0124	0,0107	0,024	0,0212	0,0297	0,0196	0,0106	0,0353	0,0031	0,0055
	143AF07	0,0167	0,0087	0	0,0163	0,0141	0,0218	0,0129	0,015	0,0106	0,0177	0,014	0,0156	0,0182	0,0237	0,0889	0,3102	0,0865	0,0379	0,0525	0,0125	0,0154	0,0099	0,0107	0,0108	0,0092	0,0138	0,0089	0,0136	0,011	0,0072	0,0332	0,0031	0,0059
	143AF08	0,0167	0,0163	0,0054	0,016	0,0195	0,029	0,0141	0,0157	0,011	0,0088	0,0103	0,0153	0,0263	0,0324	0,0249	0,083	0,0265	0,0236	0,0372	0,0047	0,0053	0,0029	0,0053	0,0047	0,0042	0,008	0,0094	0,0124	0,0061	0,0028	0,0156	0,0014	0,0023
	143AF09	0	0,0003	0	0,0003	0,0002	0,0006	0,0002	0	0,0004	0,011	0,0128	0	0,0004	0,0003	0	0	0,0001	0,0059	0,0102	0,0281	0,0361	0,0275	0,0422	0,0314	0,0179	0,0338	0,024	0,013	0,037	0,0294	0,0338	0,0089	0,0089
	143AF10	0	0,0011	0	0,0027	0,0019	0	0,0015	0,001	0,0016	0,0022	0,0019	0,0015	0,0023	0,0014	0,0024	0,0002	0,0013	0,0025	0,0027	0,0034	0,0018	0,0035	0,0013	0,0022	0,0017	0,0024	0,0009	0,0022	0,0011	0,0097	0,0015	0,0104	0,031
	143AF11	0	0,0014	0	0,0014	0,001	0,0018	0,0005	0,0012	0,0004	0,0022	0,0008	0,0015	0,0005	0,0006	0,0021	0,0007	0,0016	0,0015	0,0007	0,001	0,0014	0,0035	0,0017	0,001	0,0012	0,0015	0,0193	0,095	0,0196	0,0009	0,001	0,0019	0,0015
143AR	143AR01	0,3613	0,2801	0,3922	0,4375	0,38	0,5304	0,3973	0,562	0,5377	0,531	0,5537	0,5531	0,4531	0,5583	0,4213	0,411	0,3757	0,5083	0,5829	0,5356	0,5377	0,5012	0,5358	0,5122	0,4453	0,5488	0,5054	0,4884	0,5353	0,4658	0,5462	0,2262	0,3206
	143AR02	0,4922	0,6096	0,4865	0,4474	0,5088	0,371	0,4826	0,351	0,3605	0,3628	0,3422	0,3404	0,4477	0,3501	0,4622	0,1472	0,4644	0,3942	0,3294	0,3659	0,3577	0,3814	0,3569	0,3859	0,45	0,348	0,377	0,4193	0,3627	0,4178	0,3514	0,6282	0,5694
	143AR03	0,084	0,0798	0,0711	0,069	0,0724	0,0531	0,0732	0,0499	0,0555	0,0619	0,0608	0,063	0,0538	0,0488	0,0447	0,0229	0,0648	0,0525	0,0466	0,0578	0,0604	0,0682	0,0637	0,0593	0,0629	0,0593	0,0649	0,0569	0,0591	0,0709	0,0573	0,078	0,0723
	143AR04	0,002	0,0003	0	0,0004	0,0004	0,001	0,0007	0,0005	0,0006	0,0005	0,0004	0,0007	0,0002	0,0008	0,0519	0,3958	0,0619	0,0007	0,0008	0,0004	0,0007	0,0012	0,0004	0,0005	0,0007	0,001	0,0007	0,0005	0,0006	0,0006	0,0007	0,0006	0,0004
	143AR05	0,0156	0,0102	0,0135	0,0171	0,0152	0,0179	0,014	0,0124	0,017	0,0189	0,0165	0,0173	0,0194	0,0204	0,0098	0,0167	0,0146	0,0227	0,0234	0,0207	0,0196	0,0205	0,0187	0,0183	0,0179	0,02	0,0246	0,0178	0,0182	0,0166	0,0211	0,0082	0,0132
	143AR06	0,0254	0,0086	0,0086	0,0148	0,0082	0,0184	0,0164	0,0165	0,0199	0,0157	0,0186	0,0168	0,0091	0,0111	0,0017	0,0029	0,0059	0,0106	0,0068	0,0105	0,0155	0,0126	0,0158	0,0127	0,0071	0,0145	0,0168	0,0063	0,0152	0,0128	0,0144	0,0064	0,0055
	143AR07	0,0176	0,0102	0,027	0,0125	0,0123	0,0072	0,0145	0,0072	0,0077	0,0079	0,0069	0,0072	0,0152	0,009	0,0078	0,0029	0,0116	0,0099	0,0094	0,0085	0,0073	0,013	0,0076	0,0103	0,0152	0,0079	0,0105	0,0092	0,0083	0,0109	0,0081	0,0174	0,0144
	143AR08	0,002	0,0013	0,0012	0,0013	0,0026	0,001	0,0013	0,0005	0,001	0,0014	0,0007	0,0015	0,0014	0,0015	0,0006	0,0006	0,0011	0,0011	0,0008	0,0006	0,0012	0,002	0,0012	0,0008	0,0011	0,0005	0	0,0015	0,0007	0,0047	0,0007	0,0351	0,0043
149AF	149AF01	0,1837	0,7457	0,4851	0,4854	0,5654	0,5989	0,4677	0,6038	0,5087	0,534	0,5156	0,5546	0,5452	0,5646	0,5211	0,5978	0,5261	0,467	0,4463	0,4984	0,5737	0,502	0,5932	0,4695	0,471	0,4918	0,5934	0,6103	0,5684	0,3894	0,4438	0,3418	0,4574
	149AF02	0,0351	0,0607	0,1014	0,1629	0,1299	0,154	0,1598	0,144	0,1564	0,1773	0,174	0,1591	0,2114	0,1505	0,1616	0,1235	0,1301	0,1545	0,1991	0,1692	0,1453	0,2275	0,1577	0,2193	0,2933	0,1798	0,1973	0,1566	0,2332	0,2518	0,1961	0,1877	0,204
	149AF03	0,0768	0,0339	0,2084	0,1562	0,1545	0,1287	0,157	0,0974	0,1298	0,1521	0,1396	0,1193	0,0938	0,1202	0,1858	0,2513	0,2156	0,268	0,2927	0,1755	0,1194	0,1701	0,1678	0,1551	0,1213	0,1359	0,0829	0,1021	0,087	0,1842	0,158	0,3601	0,2704
	149AF04	0,0391	0,0205	0,0913	0,1172	0,0626	0,0976	0,1258	0,0978	0,1098	0,083	0,116	0,1076	0,0772	0,0989	0,0597	0,0127	0,0827	0,0529	0,0136	0,0888	0,0947	0,055	0,0227	0,1024	0,0666	0,1436	0,0919	0,1219	0,0671	0,1077	0,104	0,0202	0,0217
	149AF05	0,015	0,1309	0,0321	0,0747	0,0845	0,0192	0,0822	0,0558	0,0942	0,0524	0,0537	0,058	0,0712	0,0645	0,0702	0,013	0,0442	0,0564	0,0469	0,0669	0,0656	0,0441	0,0573	0,0525	0,0464	0,0477	0,0331	0,0077	0,0432	0,0645	0,097	0,0623	0,0432
	149AF06	0,2394	0,0018	0	0	0	0	0,0016	0	0	0	0	0	0	0	0	0	0	0	0	0	0	0	0	0	0	0	0	0	0	0	0	0	0
	149AF07	0,2106	0,001	0	0	0	0	0,0014	0	0	0	0	0	0	0	0	0	0	0	0	0	0	0	0	0	0	0	0	0	0	0	0	0	0
	149AF08	0,0006	0,0019	0,0012	0,0012	0,0013	0,0014	0,0012	0,0011	0,0009	0,001	0,0009	0,0011	0,0012	0,0012	0,0012	0,0011	0,001	0,0009	0,0009	0,001	0,0011	0,0012	0,0011	0,0009	0,0012	0,0011	0,001	0,0013	0,001	0,002	0,0008	0,0273	0,0031
	149AF09	0,1057	0,0014	0	0	0	0	0,0012	0	0	0	0	0	0	0	0	0	0	0	0	0	0	0	0	0	0	0	0	0	0	0	0	0	0
	149AF10	0,0171	0,0008	0,0488	0,0017	0,0012	0,0002	0,0012	0,0001	0	0,0002	0,0001	0,0003	0	0,0002	0,0004	0,0005	0,0003	0,0003	0,0005	0,0002	0,0001	0,0002	0,0002	0,0003	0,0001	0,0001	0,0004	0,0001	0,0001	0,0003	0,0003	0,0006	0,0002
	149AF11	0,0663	0,0006	0	0	0	0	0,0004	0	0	0	0	0	0	0	0	0	0	0	0	0	0	0	0	0	0	0	0	0	0	0	0	0	0
	149AF12	0,0108	0,0009	0,0318	0,0008	0,0007	0	0,0006	0	0	0	0	0	0	0	0	0	0	0	0	0	0	0	0	0	0	0	0	0	0	0	0	0	0
149AR	149AR01	0,0897	0,6514	0,2012	0,2316	0,3891	0,229	0,2246	0,2321	0,2292	0,2336	0,237	0,2413	0,2408	0,2389	0,1734	0,164	0,1535	0,1579	0,1666	0,1967	0,2243	0,2559	0,198	0,2514	0,3177	0,2387	0,2372	0,1846	0,2883	0,2903	0,1943	0,1879	0,2142
	149AR02	0,0999	0,0351	0,2586	0,2062	0,179	0,2477	0,2011	0,2051	0,155	0,2173	0,2006	0,2009	0,2276	0,2635	0,2313	0,315	0,2289	0,1981	0,2592	0,1962	0,1794	0,1303	0,2069	0,1585	0,0766	0,1619	0,2058	0,3837	0,2153	0,146	0,2327	0,1681	0,1767
	149AR03	0,0702	0,0167	0,1727	0,1003	0,0621	0,0744	0,1077	0,0478	0,1044	0,0969	0,0685	0,0652	0,082	0,0619	0,1662	0,1542	0,1776	0,2131	0,2098	0,1276	0,062	0,1005	0,0911	0,119	0,0936	0,0712	0,0814	0,0607	0,0495	0,1088	0,1088	0,2756	0,1979
	149AR04	0,0464	0,0242	0,1163	0,1327	0,0722	0,1283	0,1378	0,1073	0,14	0,1149	0,1434	0,1335	0,0892	0,1123	0,0727	0,0512	0,1104	0,0832	0,0409	0,1161	0,1164	0,0759	0,0573	0,137	0,082	0,1692	0,1085	0,1313	0,0875	0,1433	0,1127	0,0432	0,0351
	149AR05	0,0218	0,1588	0,0381	0,0827	0,1029	0,0157	0,0911	0,0673	0,117	0,0679	0,0598	0,071	0,071	0,0683	0,0851	0,0145	0,0513	0,064	0,0523	0,0828	0,0738	0,0541	0,069	0,0572	0,0564	0,0571	0,04	0,0108	0,0502	0,0776	0,1052	0,0688	0,0497
	149AR06	0,0099	0,0136	0,0145	0,0327	0,0086	0,0947	0,0411	0,128	0,0573	0,054	0,0476	0,08	0,0974	0,0739	0,0021	0,0057	0,0023	0,002	0,0028	0,0073	0,0339	0,0624	0,0083	0,0371	0,0596	0,0619	0,0668	0,0537	0,0679	0,003	0,0009	0,0007	0,001
	149AR07	0,0045	0,0016	0,003	0,0136	0,0012	0,0185	0,0143	0,0012	0,0013	0,0104	0,0278	0,0157	0,0429	0,0021	0,0416	0,0032	0,0554	0,0688	0,0402	0,0535	0,0632	0,0885	0,0994	0,0252	0,0871	0,0411	0,0718	0,0061	0,0417	0,032	0,0867	0,0043	0,0915
	149AR08	0,0081	0,0098	0,0118	0,0314	0,0208	0,0262	0,0333	0,0501	0,0324	0,0432	0,0257	0,0284	0,0312	0,0307	0,0409	0,0587	0,0423	0,0259	0,024	0,0243	0,0269	0,0164	0,0262	0,0236	0,0386	0,0233	0,0261	0,0298	0,0237	0,0313	0,0149	0,0588	0,0889
	149AR09	0,0063	0,0123	0,0258	0,035	0,0534	0,023	0,0228	0,0331	0,0428	0,0359	0,0323	0,0288	0,0176	0,0335	0,0215	0,0723	0,0315	0,0278	0,0338	0,0223	0,0261	0,0219	0,0282	0,0318	0,0252	0,0225	0,0161	0,0146	0,0192	0,0283	0,0155	0,0182	0,0218
	149AR10	0,0175	0,0063	0,0407	0,0295	0,0192	0,0174	0,0328	0,0102	0,0188	0,0185	0,0154	0,0138	0,0197	0,0164	0,0266	0,0286	0,0353	0,0451	0,0488	0,0305	0,0137	0,0228	0,0173	0,0264	0,0214	0,0176	0,0187	0,0191	0,0129	0,0235	0,0225	0,0611	0,0328
	149AR11	0,025	0,0078	0,0627	0,0361	0,0297	0,0049	0,0288	0,0332	0,0393	0,0163	0,0207	0,0175	0,0075	0,0169	0,0498	0,0617	0,0379	0,0165	0,0366	0,0151	0,0197	0,0119	0,0133	0,0036	0,0194	0,0057	0,0125	0,0166	0,0118	0,0009	0,0071	0,0003	0,0005
	149AR12	0,0022	0,0023	0,003	0,0086	0,002	0,0191	0,0074	0,0023	0,0021	0,0156	0,0318	0,0188	0,007	0,0026	0,0073	0,0042	0,0182	0,033	0,0182	0,0359	0,055	0,0485	0,0741	0,0308	0,0367	0,0362	0,0328	0,0088	0,0396	0,0277	0,0332	0,0039	0,0074
	149AR13	0,0065	0,051	0,0165	0,0172	0,0327	0,014	0,0159	0,0102	0,011	0,0123	0,0129	0,0124	0,0149	0,0155	0,0091	0,0092	0,0115	0,0122	0,0153	0,017	0,0126	0,0171	0,0105	0,0165	0,026	0,0149	0,0128	0,0139	0,0163	0,018	0,011	0,0168	0,0126
	149AR14	0,008	0,0031	0,0186	0,016	0,0147	0,0141	0,0161	0,0104	0,0087	0,0141	0,0113	0,0113	0,0185	0,0189	0,014	0,0196	0,0165	0,0159	0,0231	0,0151	0,0114	0,0098	0,0127	0,0095	0,0057	0,0098	0,0129	0,0322	0,014	0,0097	0,0136	0,0161	0,0151
	149AR15	0,0015	0,0007	0,0064	0,0066	0,005	0,0087	0,0045	0,0057	0,0083	0,0115	0,011	0,0058	0,0043	0,0074	0,0059	0,021	0,0067	0,0095	0,0096	0,0238	0,0178	0,0189	0,0129	0,0361	0,0066	0,0138	0,0037	0,0042	0,0034	0,0345	0,014	0,0112	0,038
	149AR16	0,0012	0,0008	0,0009	0,0042	0,0007	0,0107	0,0035	0,0018	0,0018	0,0078	0,0163	0,0099	0,0058	0,002	0,0046	0,0015	0,0076	0,0125	0,0071	0,0178	0,0298	0,0351	0,0388	0,0147	0,031	0,0193	0,0257	0,0064	0,0254	0,0141	0,0132	0,0027	0,0062
	149AR17	0,0026	0,0013	0,0056	0,0074	0,0043	0,0282	0,007	0,0326	0,0153	0,0144	0,0154	0,0228	0,0128	0,022	0,0029	0,0087	0,0027	0,0013	0,0029	0,0033	0,0081	0,0102	0,0033	0,0073	0,0043	0,0116	0,0105	0,0132	0,0142	0,0005	0,0011	0,0002	0,0002
	149AR18	0,0011	0,0005	0,0006	0,0026	0,0005	0,0072	0,0026	0,0011	0,0008	0,0051	0,0105	0,0053	0,0028	0,0011	0,044	0,0046	0,0087	0,0123	0,0081	0,0126	0,0188	0,0117	0,0303	0,0065	0,0059	0,009	0,0096	0,0033	0,0112	0,0061	0,0123	0,0016	0,0028
	149AR19	0,0028	0,0007	0,0029	0,0053	0,0017	0,0181	0,0053	0,0204	0,0145	0,0096	0,0119	0,0174	0,0067	0,012	0,0007	0,0018	0,0013	0,0007	0,0004	0,002	0,0069	0,008	0,0019	0,0078	0,006	0,0151	0,0068	0,0064	0,0076	0,0007	0,0002	0,0003	0,0003
	149AR20	0,2523	0,0009	0	0	0	0	0,0009	0	0	0	0	0	0	0	0	0	0	0	0	0	0	0	0	0	0	0	0	0	0	0	0	0	0
	149AR21	0,178	0,0004	0	0	0	0	0,0005	0	0	0	0	0	0	0	0	0	0	0	0	0	0	0	0	0	0	0	0	0	0	0	0	0	0
	149AR22	0	0	0	0	0	0	0	0	0	0	0	0	0	0	0	0	0	0	0	0	0	0	0	0	0	0	0	0	0	0,0022	0	0,0361	0,0047
	149AR23	0	0	0,0002	0,0002	0,0002	0,0003	0,0002	0,0001	0	0,0004	0,0001	0,0001	0,0004	0,0002	0,0002	0,0002	0,0002	0,0001	0,0003	0,0002	0,0003	0	0,0002	0,0001	0	0,0002	0,0003	0,0004	0,0001	0,0015	0,0002	0,024	0,0028
	149AR24	0,0588	0,0004	0	0	0	0	0,0003	0	0	0	0	0	0	0	0	0	0	0	0	0	0	0	0	0	0	0	0	0	0	0	0	0	0
	149AR25	0,0567	0	0	0	0	0	0,0002	0	0	0	0	0	0	0	0	0	0	0	0	0	0	0	0	0	0	0	0	0	0	0	0	0	0
	149AR26	0,0288	0,0001	0	0	0	0	0,0002	0	0	0	0	0	0	0	0	0	0	0	0	0	0	0	0	0	0	0	0	0	0	0	0	0	0
174AF	174AF01	0,2874	0,4154	0,2761	0,4014	0,5454	0,5791	0,3083	0,5357	0,5114	0,5227	0,524	0,5163	0,7244	0,5534	0,6551	0,5428	0,6968	0,5571	0,4674	0,5149	0,4971	0,4799	0,5169	0,5091	0,1613	0,5204	0,5157	0,6107	0,5394	0,5683	0,5385	0,3236	0,7249
	174AF02	0,1211	0,0491	0,1116	0,1241	0,0734	0,1008	0,1128	0,1199	0,1253	0,1066	0,1207	0,1172	0,0605	0,1121	0,0475	0,098	0,0834	0,1007	0,1183	0,1217	0,0843	0,1314	0,1185	0,1299	0,1864	0,1195	0,0793	0,1199	0,1339	0,0948	0,1224	0,0583	0,0406
	174AF03	0,0702	0,069	0,0535	0,0786	0,0429	0,0441	0,1179	0,0856	0,0861	0,0833	0,0921	0,0834	0,0394	0,0791	0,018	0,016	0,02	0,0293	0,0347	0,089	0,1515	0,0938	0,1057	0,0915	0,1548	0,0912	0,067	0,0394	0,079	0,076	0,0639	0,0248	0,0491
	174AF04	0,0459	0,0151	0,0664	0,047	0,0308	0,0285	0,0425	0,0322	0,0356	0,0375	0,0409	0,0357	0,0181	0,0348	0,0948	0,1042	0,0443	0,1148	0,165	0,0526	0,0508	0,0617	0,0576	0,0579	0,0806	0,0556	0,1139	0,048	0,0636	0,0441	0,0859	0,0455	0,0142
	174AF05	0,0518	0,0155	0,0652	0,0625	0,0464	0,0094	0,0512	0,0117	0,0123	0,0264	0,0164	0,0133	0,0072	0,0117	0,0024	0,0124	0,0063	0,0125	0,0114	0,106	0,0809	0,0841	0,088	0,0719	0,1439	0,0624	0,0743	0,0227	0,0737	0,1014	0,0471	0,2616	0,0684
	174AF06	0,1863	0,0355	0,1857	0,1024	0,0284	0,0773	0,1509	0,0887	0,082	0,0552	0,0088	0,083	0,0461	0,0738	0,0157	0,0157	0,0238	0,0122	0,0126	0,0256	0,0176	0,0193	0,0156	0,0167	0,0387	0,0197	0,0209	0,0406	0,0239	0,0279	0,0161	0,076	0,0259
	174AF07	0,0476	0,0186	0,0429	0,0279	0,0113	0,0124	0,0423	0,0133	0,0125	0,0086	0,0018	0,014	0,0094	0,0142	0,0948	0,0711	0,0377	0,0633	0,0567	0,0056	0,0019	0,0027	0,0024	0,002	0,0057	0,0028	0,0045	0,0067	0,0029	0,0048	0,0232	0,0116	0,005
	174AF08	0,0309	0,0033	0,0317	0,0108	0,0059	0,0272	0,0091	0,0274	0,032	0,0295	0,0317	0,0328	0,0105	0,0218	0,0201	0,0261	0,0124	0,0302	0,0553	0,0108	0,0089	0,0104	0,0079	0,0099	0,0144	0,015	0,0272	0,0091	0,011	0,0074	0,022	0,0109	0,0039

		Ⓓ	Ⓒ	①	②	③	①	②	③	④	⑤	⑥	⑦	⑧	⑨	①	②	③	④	⑤	①	②	③	④	⑤	⑥	⑦	①	②	③	①	②	③	④
Marker	haplotype	Ador	Acer1.1	GRE5.6	CAR2.1	POR	RUN0065	RUN0067	RUN0109	RUN0281	RUN1041	RUN1050	RUN1132	RUN1232	RUN1578	MU011I	MU025I	MU040I	ROD001	ROD200	COM11	COM12	COM18	COM28	COM59	COM72	MAY35	SEY059	SEY141	SEY144	ISM03	ISM04	MAD26.1	MAD30.2
	174AF09	0,0251	0,0053	0,0288	0,0166	0,0124	0,0104	0,0154	0,0127	0,0141	0,0172	0,0052	0,0151	0,0056	0,0086	0,0013	0,0067	0,0055	0,0042	0,0031	0,0272	0,0188	0,0257	0,0208	0,021	0,0383	0,0216	0,0137	0,0064	0,0156	0,0232	0,0144	0,0715	0,016
	174AF10	0,0058	0,0142	0,0129	0,0158	0,0226	0,0122	0,0132	0,0103	0,0107	0,0134	0,0131	0,0103	0,0127	0,0113	0,018	0,0156	0,0221	0,0272	0,031	0,015	0,0119	0,0124	0,0094	0,0123	0,0065	0,0119	0,012	0,0227	0,0136	0,0161	0,0193	0,0102	0,0291
	174AF11	0,0067	0,0164	0,01	0,0117	0,0092	0,0252	0,0124	0,0154	0,0159	0,0187	0,0205	0,0231	0,0236	0,0247	0,0032	0,0208	0,0091	0,0043	0,0046	0,0057	0,0195	0,018	0,0153	0,0228	0,0091	0,0186	0,0171	0,0081	0,0083	0,0079	0,0137	0,0074	0,0033
	174AF12	0,01	0,0147	0,0082	0,011	0,0112	0,024	0,0095	0,0146	0,0162	0,014	0,0029	0,0172	0,0172	0,0175	0,0034	0,0132	0,0089	0,0065	0,0052	0,0081	0,0169	0,0135	0,0137	0,0136	0,0041	0,0109	0,0107	0,0053	0,0079	0,0091	0,0096	0,035	0,006
	174AF13	0,0075	0,0007	0,0082	0,003	0,0021	0,0237	0,0025	0,0141	0,0204	0,0358	0,0767	0,0151	0,0097	0,0154	0,001	0,0046	0,0033	0,0014	0,0009	0,0015	0,0049	0,0057	0,0025	0,0038	0,0302	0,0186	0,0053	0,0012	0,0026	0,0029	0,0011	0,0109	0,0018
	174AF14	0,0443	0,2062	0,0417	0,0298	0,0948	0	0,0355	0,0001	0	0	0	0	0,0001	0	0	0	0	0	0	0	0	0	0	0	0	0	0	0	0	0	0	0	0
	174AF15	0,0042	0,0008	0,0053	0,0092	0,0037	0,0073	0,0062	0,0031	0,0062	0,0084	0,0161	0,0038	0,0039	0,0053	0,0019	0,0081	0,0031	0,0032	0,0031	0,0072	0,0192	0,0236	0,013	0,0202	0,0932	0,0138	0,0196	0,0026	0,0068	0,0087	0,0073	0,0329	0,005
	174AF16	0,0042	0,0009	0,0041	0,008	0,0023	0,011	0,0068	0,0081	0,0104	0,0144	0,0225	0,0103	0,006	0,0084	0,0008	0,0057	0,0053	0,0025	0,0016	0,0036	0,0089	0,0129	0,0083	0,0148	0,0292	0,012	0,0055	0,0029	0,0042	0,005	0,0054	0,0141	0,0022
	174AF17	0,0276	0,09	0,0317	0,0198	0,0415	0,0015	0,0272	0,0014	0,0016	0,0012	0,0002	0,0013	0,0016	0,0018	0,0149	0,0126	0,0075	0,0145	0,014	0,001	0,0005	0,0004	0,0004	0,0004	0,0012	0,0007	0,0004	0,0014	0,0007	0,0002	0,0046	0,0008	0,001
	174AF18	0,0067	0,0013	0,0029	0,0004	0,0017	0,0037	0,0014	0,0038	0,0059	0,0054	0,005	0,0056	0,002	0,0042	0,0044	0,0232	0,007	0,0114	0,0118	0,0022	0,001	0,0026	0,0013	0,0008	0,0014	0,003	0,0035	0,0026	0,0015	0,001	0,0031	0,0014	0,0004
	174AF19	0,0033	0,0214	0,0053	0,0044	0,0097	0,0009	0,0057	0,0012	0,0007	0,0012	0,0002	0,0011	0,0006	0,0013	0,0013	0,0022	0,0021	0,0035	0,0023	0,0015	0,0039	0,0008	0,0009	0,0007	0,0002	0,0009	0,0004	0	0,0002	0,0004	0,0015	0,0014	0,001
	174AF20	0,0125	0,0062	0,0071	0,0154	0,004	0,0008	0,0288	0,0003	0,0002	0,0003	0,0002	0,0005	0,0009	0,0001	0,0005	0,0005	0,0006	0,0001	0,0004	0,0005	0,0006	0,0005	0,0009	0,0002	0,0002	0,0006	0,0004	0	0,0004	0,0006	0,0005	0,001	0,001
	174AF21	0,0008	0,0005	0,0006	0,0001	0,0004	0,0006	0,0005	0,0004	0,0004	0,0005	0,0009	0,0007	0,0004	0,0004	0,001	0,0004	0,0009	0,0011	0,0005	0,0004	0,0005	0,0005	0,0006	0,0008	0,0006	0,0007	0,0086	0,0497	0,0108	0,0004	0,0003	0,0011	0,0012
174AR	174AR01	0,4067	0,5308	0,4181	0,4458	0,4334	0,4067	0,4473	0,4392	0,4506	0,4348	0,4524	0,4537	0,3464	0,4516	0,3446	0,4205	0,3198	0,437	0,533	0,4249	0,4495	0,4521	0,4556	0,46	0,5122	0,4595	0,4318	0,3925	0,4604	0,3897	0,4551	0,2319	0,242
	174AR02	0,1483	0,2481	0,1902	0,2749	0,3528	0,3827	0,2325	0,3725	0,3609	0,3589	0,349	0,3678	0,5359	0,3788	0,4603	0,3917	0,5264	0,4045	0,3426	0,3317	0,36	0,3552	0,3604	0,3629	0,0886	0,3629	0,3839	0,4416	0,3688	0,3489	0,3922	0,238	0,4084
	174AR03	0,3598	0,0482	0,331	0,2278	0,1188	0,1965	0,2427	0,1806	0,1802	0,1977	0,188	0,1692	0,1104	0,1604	0,1843	0,1758	0,1408	0,149	0,1184	0,226	0,1808	0,1819	0,175	0,1682	0,3785	0,1675	0,1726	0,1558	0,1608	0,2104	0,1453	0,4913	0,1939
	174AR04	0,0852	0,1721	0,0607	0,0509	0,0945	0,0141	0,0768	0,007	0,0071	0,008	0,0095	0,0084	0,0064	0,0087	0,0097	0,0096	0,0082	0,0088	0,0054	0,0169	0,0089	0,0103	0,0084	0,0079	0,0203	0,0092	0,0105	0,0097	0,0093	0,0116	0,0068	0,0251	0,01
	174AR05	0	0,0006	0	0,0006	0,0002	0	0,0004	0,0005	0,001	0,0003	0,0007	0,0004	0,0006	0,0005	0	0,0005	0,0006	0,0007	0,0005	0,0005	0,0003	0,0004	0,0005	0,0007	0,0002	0,0004	0,0006	0,0002	0,0005	0,0223	0,0004	0,008	0,0881
	174AR06	0	0,0002	0	0	0,0002	0	0,0003	0,0002	0,0002	0,0003	0,0004	0,0006	0,0004	0	0,001	0,0019	0,0042	0	0,0002	0	0,0005	0	0,0002	0,0002	0,0002	0,0005	0,0006	0,0002	0,0002	0,0172	0,0002	0,0056	0,0576
185AF	185AF01	0,4476	0,1471	0,4501	0,4723	0,3295	0,5689	0,4216	0,6066	0,6843	0,5612	0,5763	0,6035	0,5107	0,5396	0,2021	0,4405	0,2564	0,3298	0,3494	0,56	0,5623	0,5263	0,5854	0,2521	0,4934	0,561	0,5571	0,545	0,568	0,5523	0,528	0,5063	0,5114
	185AF02	0,1937	0,0661	0,192	0,2449	0,1318	0,2289	0,2635	0,229	0,1152	0,2459	0,2408	0,22	0,2335	0,1969	0,3368	0,2567	0,2503	0,1733	0,1607	0,2155	0,2411	0,2755	0,2291	0,5967	0,2741	0,2255	0,2326	0,2197	0,2288	0,2265	0,2165	0,2289	0,1998
	185AF03	0,1571	0,1483	0,1767	0,138	0,1642	0,1395	0,1386	0,1228	0,1606	0,1364	0,1308	0,1312	0,168	0,1535	0,3296	0,2131	0,3439	0,365	0,3491	0,1577	0,1372	0,1302	0,1334	0,0562	0,1527	0,1504	0,147	0,1601	0,145	0,1574	0,1812	0,169	0,2048
	185AF04	0,1126	0,5152	0,1167	0,0837	0,288	0,0211	0,1065	0,0157	0,0232	0,0234	0,0196	0,0185	0,0334	0,0282	0,0697	0,0462	0,0875	0,0878	0,0943	0,0291	0,0229	0,0244	0,0204	0,0106	0,0296	0,0277	0,025	0,0329	0,0251	0,0284	0,0394	0,0404	0,0427
	185AF05	0,0497	0,0158	0,0338	0,0434	0,0229	0,0379	0,0478	0,0241	0,0139	0,0306	0,0301	0,0253	0,0501	0,0771	0,0507	0,0374	0,0477	0,0307	0,0317	0,0331	0,0341	0,0412	0,0293	0,0828	0,0461	0,0322	0,0362	0,0374	0,0295	0,031	0,0297	0,05	0,0344
	185AF06	0,0393	0,1074	0,0307	0,0177	0,0637	0,0036	0,022	0,0018	0,0027	0,0026	0,0024	0,0015	0,0042	0,0047	0,0111	0,0061	0,0142	0,0135	0,0148	0,0046	0,0024	0,0024	0,0024	0,0016	0,0042	0,0032	0,0021	0,0048	0,0036	0,0044	0,0052	0,0054	0,0069
185AR	185AR01	0,8193	0,7714	0,8076	0,8612	0,8252	0,8981	0,8513	0,9201	0,9052	0,8909	0,9007	0,9129	0,882	0,8859	0,8995	0,8899	0,8813	0,8754	0,8756	0,8857	0,8962	0,8879	0,9025	0,8944	0,8929	0,8955	0,8881	0,8704	0,8976	0,8637	0,9017	0,7115	0,7969
	185AR02	0,0777	0,0668	0,0672	0,0733	0,068	0,0573	0,0774	0,0469	0,0552	0,0634	0,0613	0,052	0,0655	0,0636	0,0571	0,0653	0,0692	0,0688	0,0692	0,0667	0,0606	0,0648	0,0572	0,0581	0,0662	0,0624	0,0637	0,0681	0,0617	0,0647	0,0601	0,0504	0,0695
	185AR03	0,0287	0,026	0,0394	0,0298	0,0268	0,0286	0,0316	0,0204	0,0243	0,029	0,023	0,0239	0,0317	0,0282	0,0263	0,0265	0,0293	0,0297	0,0292	0,0264	0,0251	0,0257	0,0222	0,0259	0,0274	0,0272	0,028	0,0261	0,0242	0,0249	0,0232	0,0261	0,0265
	185AR04	0,0186	0,0156	0,0209	0,0164	0,0177	0,0118	0,0195	0,0084	0,0095	0,0116	0,0103	0,0065	0,0166	0,0166	0,0116	0,0122	0,0128	0,0145	0,02	0,0158	0,0131	0,0157	0,0101	0,0152	0,0097	0,0099	0,0147	0,0281	0,0116	0,012	0,0103	0,0217	0,0221
	185AR05	0,0372	0,114	0,0185	0,0124	0,0567	0	0,0128	0	0	0	0	0	0	0	0	0	0	0	0	0	0	0	0	0	0	0	0	0	0	0	0	0	0
	185AR06	0	0,0003	0,0023	0,0009	0,0008	0,0007	0,0012	0,0011	0,0016	0,0003	0,0008	0,0013	0,0008	0,0019	0,0007	0,0011	0,001	0,0036	0,0013	0,0014	0,0011	0,0007	0,0011	0,0017	0,001	0,0008	0,0014	0,0014	0,0011	0,0122	0,0007	0,0109	0,034
	185AR07	0	0,0011	0	0,001	0,0008	0,0007	0,0005	0,0006	0,0008	0,0012	0,0009	0,0004	0,0006	0,0004	0,0015	0,0006	0,0008	0,0022	0,0008	0,0014	0,0007	0,0005	0,0019	0,0005	0,0005	0,0009	0,0012	0,0031	0,0014	0,0063	0,0013	0,0066	0,0218
	185AR08	0,0034	0,001	0,0023	0,0014	0,0009	0,0008	0,0022	0,0007	0,0012	0,002	0,0009	0,0007	0,0006	0,0009	0,0006	0,0009	0,0005	0,001	0,0007	0,0004	0,001	0,0007	0,0012	0,0016	0,0005	0,001	0,0007	0,0012	0,0003	0,0047	0,0008	0,0059	0,0188
	185AR09	0	0,0018	0,0012	0,0019	0,0009	0,0004	0,0015	0,0006	0,0006	0,0003	0,0004	0,0008	0,0005	0,0005	0,0019	0,0021	0,0039	0,0028	0,0016	0,0007	0,0008	0,002	0,0009	0,001	0,0004	0,0004	0,001	0,0005	0,0005	0,0058	0,0009	0,0788	0,0046
	185AR10	0	0,0004	0	0,0004	0,0006	0,0009	0,0005	0,0004	0,0004	0,0005	0,0006	0,0005	0,0006	0,0012	0,0001	0,0004	0,0004	0,0009	0,0007	0,0003	0,0005	0,0007	0,0012	0,0006	0,0007	0,0007	0,0004	0,0005	0,0005	0,0051	0,0005	0,0871	0,0054
	185AR11	0,0152	0,0017	0,0406	0,0013	0,0015	0,0007	0,0012	0,0009	0,0013	0,0008	0,0012	0,001	0,0011	0,0007	0,0007	0,0011	0,0008	0,0012	0,0008	0,0011	0,0009	0,0013	0,0017	0,001	0,0007	0,0012	0,0008	0,0007	0,0011	0,0007	0,0005	0,0011	0,0006
386AF	386AF01	0,1581	0,0446	0,1382	0,2824	0,2563	0,2267	0,2843	0,3862	0,3742	0,2649	0,261	0,3134	0,102	0,1337	0,2725	0,2614	0,1946	0,3042	0,3412	0,318	0,2338	0,2853	0,2779	0,2935	0,3576	0,2916	0,2765	0,3121	0,2829	0,3296	0,2988	0,1641	0,2427
	386AF02	0,3611	0,3443	0,4842	0,2535	0,3571	0,3012	0,1875	0,022	0,0175	0,2811	0,301	0,168	0,1808	0,4086	0,2722	0,3009	0,3297	0,2505	0,1734	0,2572	0,4337	0,4201	0,2794	0,2829	0,2682	0,2629	0,2931	0,2006	0,2465	0,0366	0,2857	0,3065	0,1701
	386AF03	0,2352	0,1357	0,2266	0,1696	0,1466	0,1729	0,1757	0,2314	0,4081	0,1857	0,1724	0,2269	0,284	0,0763	0,2208	0,1957	0,2663	0,2019	0,2403	0,1631	0,0301	0,0039	0,1684	0,1733	0,1895	0,1949	0,1686	0,191	0,1808	0,4256	0,1841	0,3313	0,2292
	386AF04	0,1282	0,0465	0,0708	0,1517	0,0724	0,2167	0,1871	0,1707	0,1698	0,1671	0,1644	0,182	0,3448	0,277	0,1436	0,1376	0,0837	0,1505	0,1404	0,1655	0,1466	0,151	0,1641	0,159	0,1165	0,1587	0,165	0,1814	0,1836	0,1819	0,1467	0,177	0,2236
	386AF05	0,1109	0,3168	0,0713	0,1377	0,1364	0,0813	0,1585	0,1086	0,0161	0,0995	0,0993	0,0894	0,0877	0,1033	0,0902	0,1026	0,1241	0,0911	0,1039	0,0949	0,1541	0,1387	0,1086	0,0892	0,0672	0,0908	0,0952	0,1135	0,1044	0,0168	0,0835	0,0054	0,0801
	386AF06	0,0008	0,0005	0,0011	0,0004	0,0005	0,0004	0,0005	0,0803	0,0139	0,0008	0,0009	0,0195	0,0004	0,0006	0,0005	0,0009	0,001	0,0005	0,0002	0,0009	0,0005	0,0002	0,0007	0,0009	0,0003	0,0007	0,0009	0,0005	0,0006	0,0002	0,0004	0	0,0002
	386AF07	0,0039	0,1112	0,0039	0,0032	0,0299	0	0,0053	0	0	0	0	0	0	0	0	0	0	0	0	0	0	0,0002	0	0	0	0	0	0	0	0	0	0	0
	386AF08	0,0016	0,0005	0,0017	0,0008	0,0004	0	0,0006	0	0	0,0003	0,0004	0,0001	0,0002	0,0003	0	0,0002	0,0002	0,0005	0	0,0002	0,0003	0,0005	0	0,0006	0,0001	0,0003	0	0	0,0002	0,0039	0,0002	0,011	0,0328
	386AF09	0	0	0,0022	0,0007	0,0003	0,0008	0,0006	0,0007	0,0004	0,0006	0,0007	0,0006	0,0003	0,0003	0,0002	0,0007	0,0003	0,0009	0,0006	0,0002	0,0009	0,0002	0,0009	0,0006	0,0006	0,0002	0,0007	0,0009	0,0011	0,0054	0,0007	0,0047	0,0211

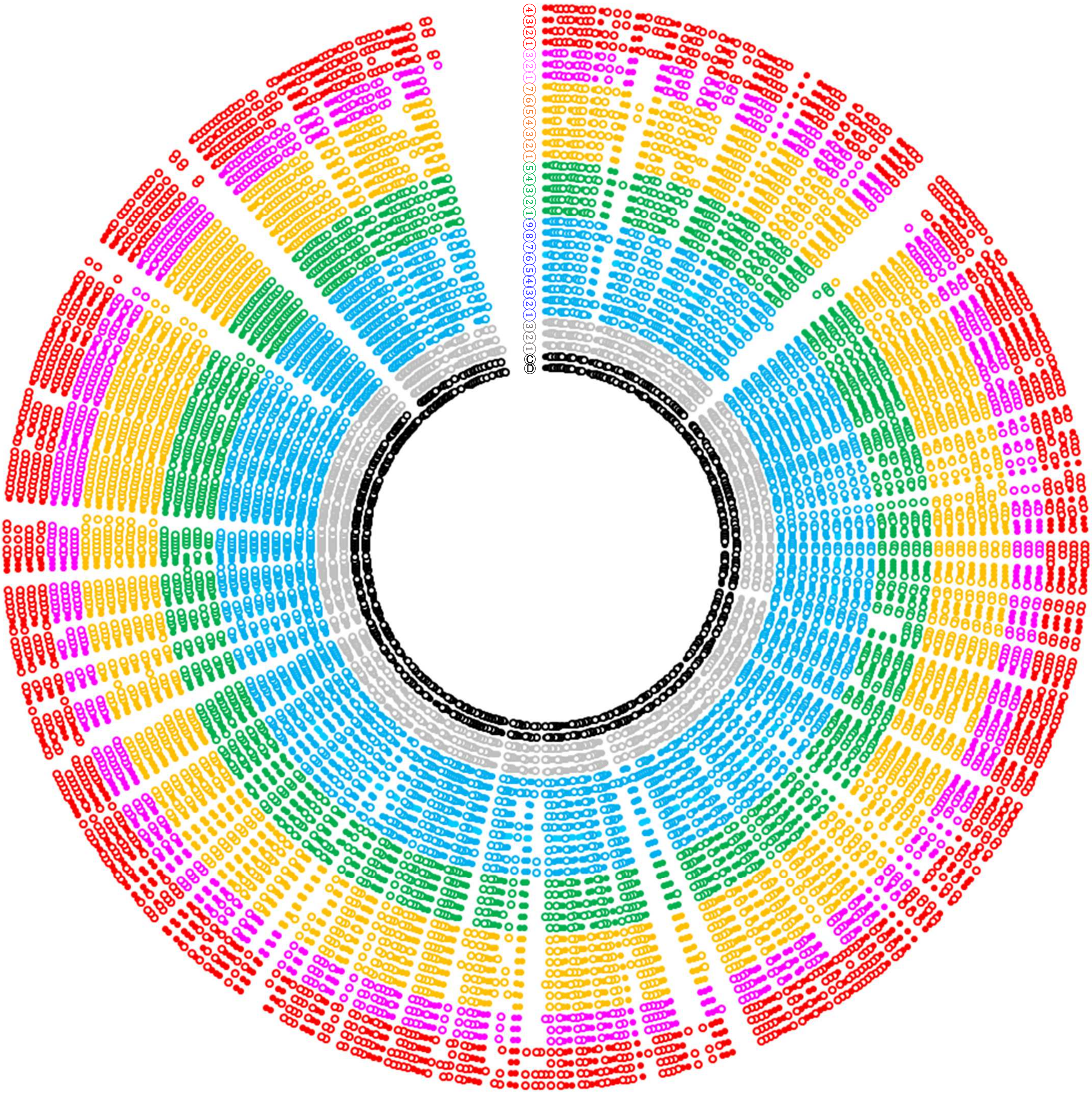

Figure S1. Presence of haplotype in isolates. From inside to outside are Asian host outgroup (ⒹⒸ), European outgroup (①②③), and isolates from La Reunion (①②③④⑤⑥⑦⑧⑨), Mauritius (①②③④⑤), Comoros (①②③④⑤⑥⑦), Seychelles (①②③), Madagascar (①②③④). Filled and empty dots indicate high (>0,12) or low (between 0,001 and 0,12) frequency in each isolate respectively. See Table 1 for isolate identity.

Following pages

Figure S2. Haplotypes distribution in the isolates. Each marker, with forward (F) and reverse (R) side of the *locus*, was considered separately. The type of marker is indicated in parenthesis (CR: coding region, NC: non-coding region, MS: microsatellite). The top histograms represent the haplotype composition of the indicated marker in every isolate (indicated on the left). The bottom tree indicate the evolutionary relationships of haplotypes inferred by the Neighbor-Joining method computed using *p*-distances. "del" indicates the presence of a deletion. The numbers of putative motive repeats are indicated for microsatellites and other markers. The scale bars represent a *p*-distance of 0.001. Trees were poorly supported by bootstrap analyses. Each haplotype was represented by the same color in both figures.

001AF (CR, translation elongation factor EF-2)

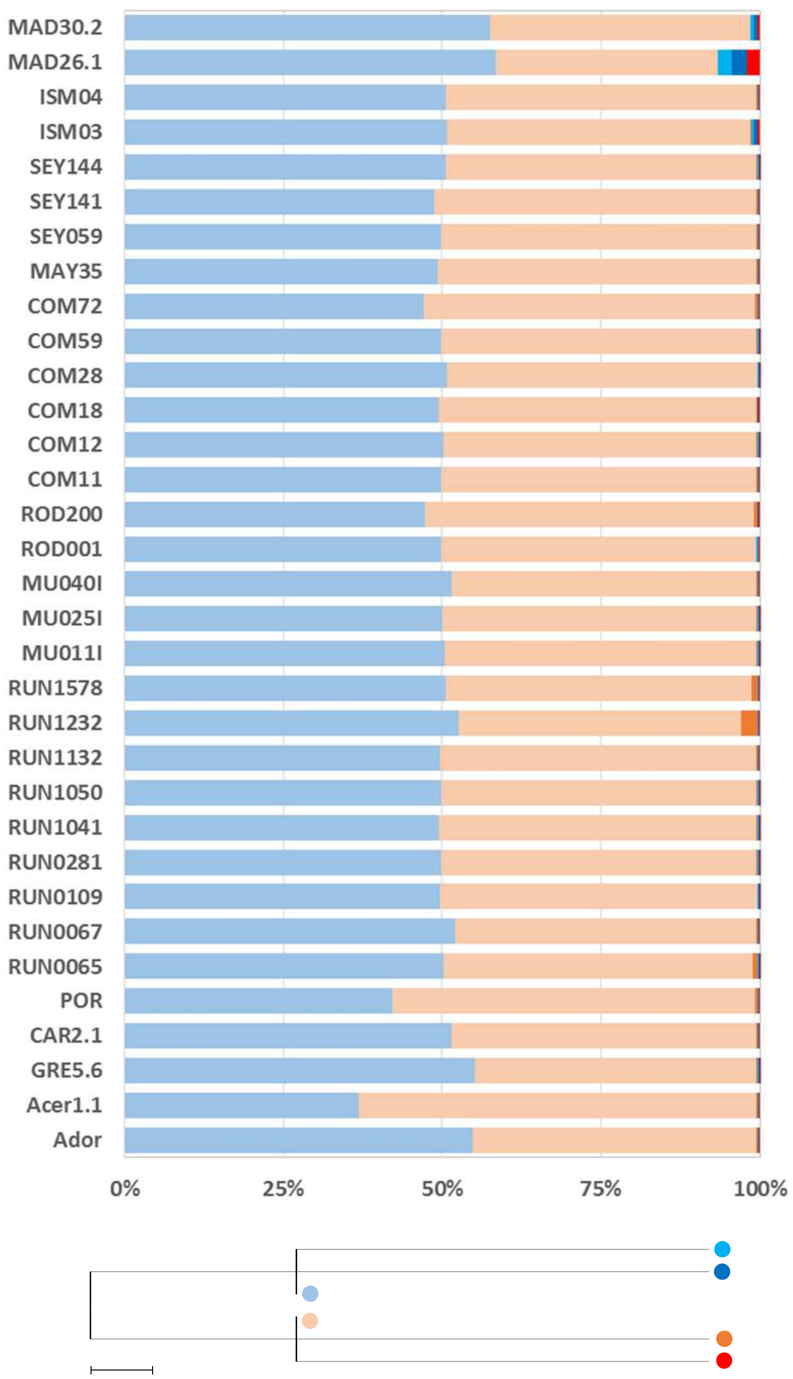

001AR (CR, translation elongation factor EF-2)

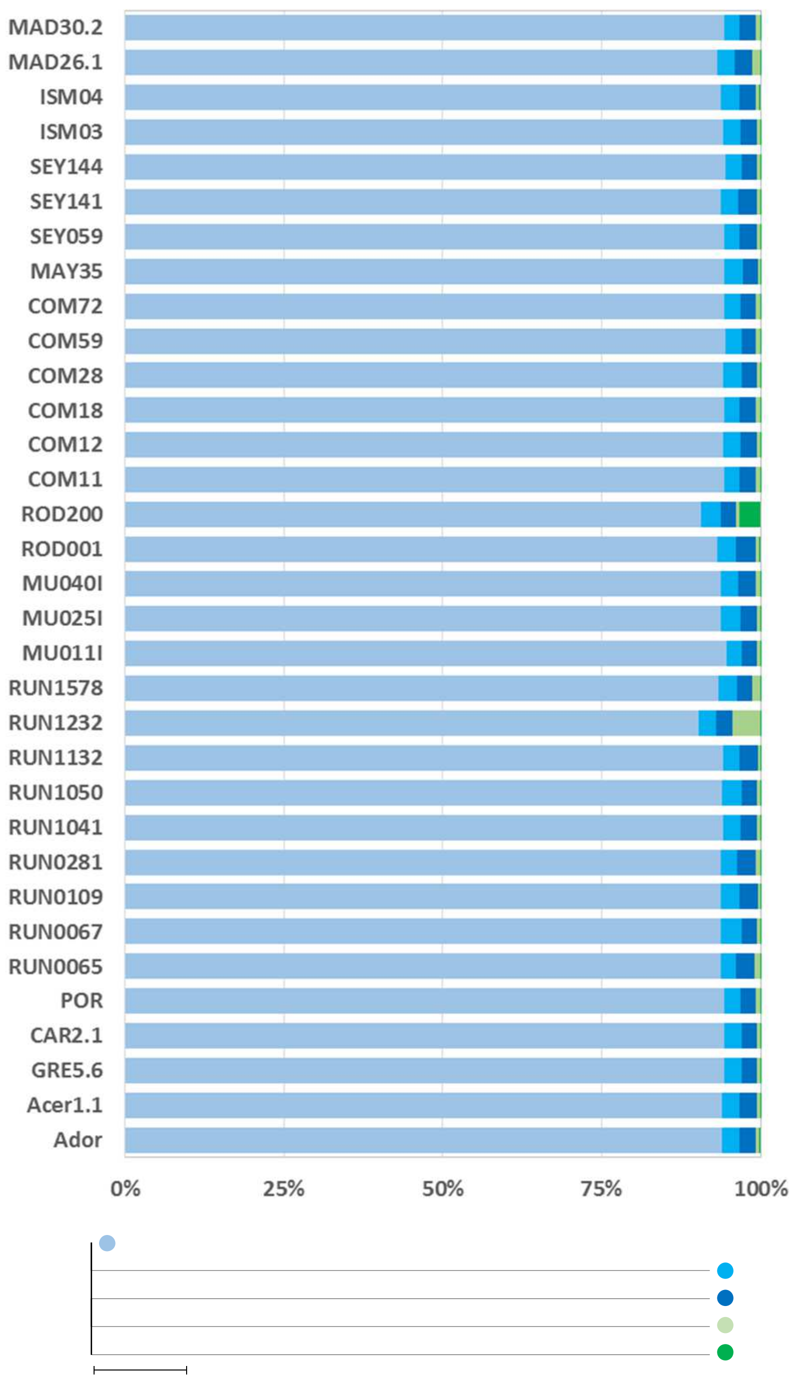

001BF (CR, polar tube protein PTP3)

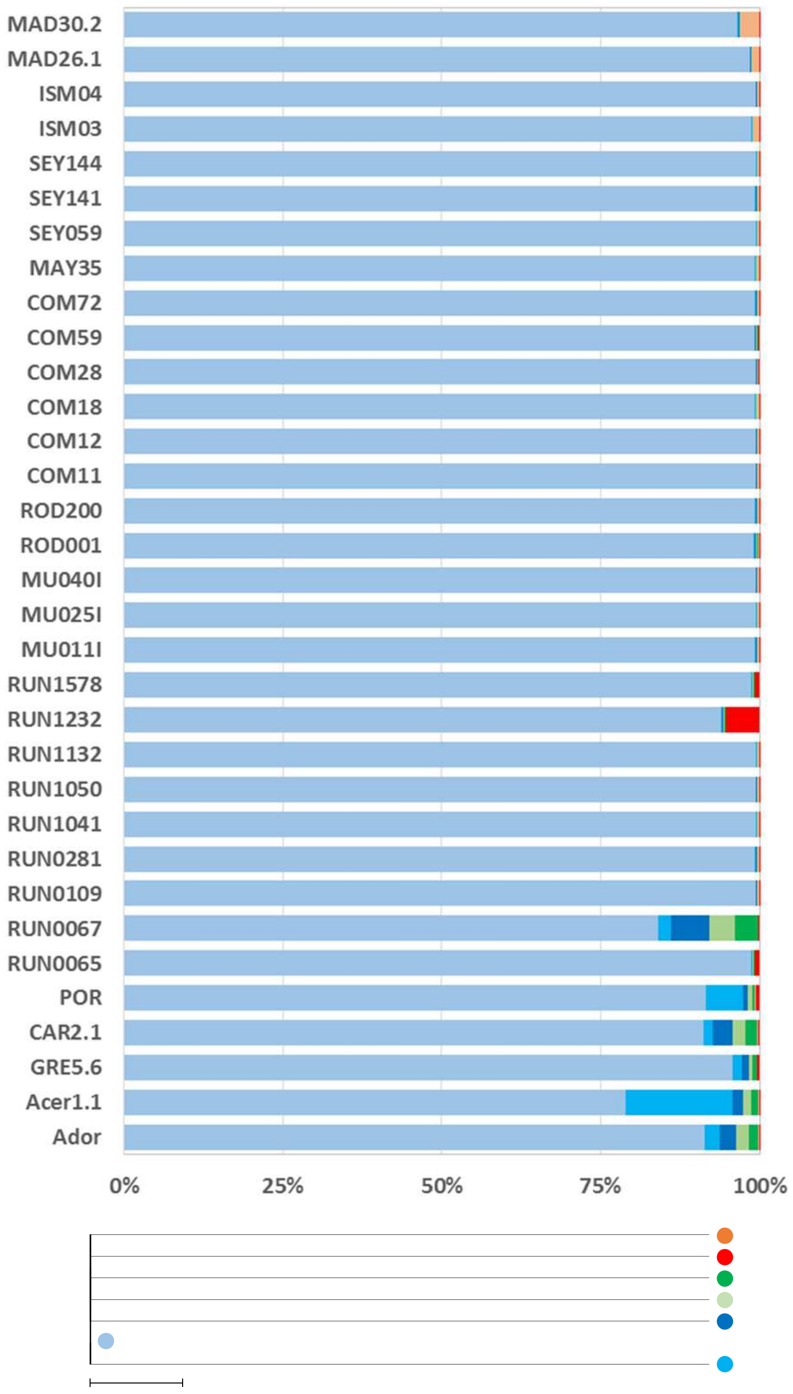

001BR (CR, polar tube protein PTP3)

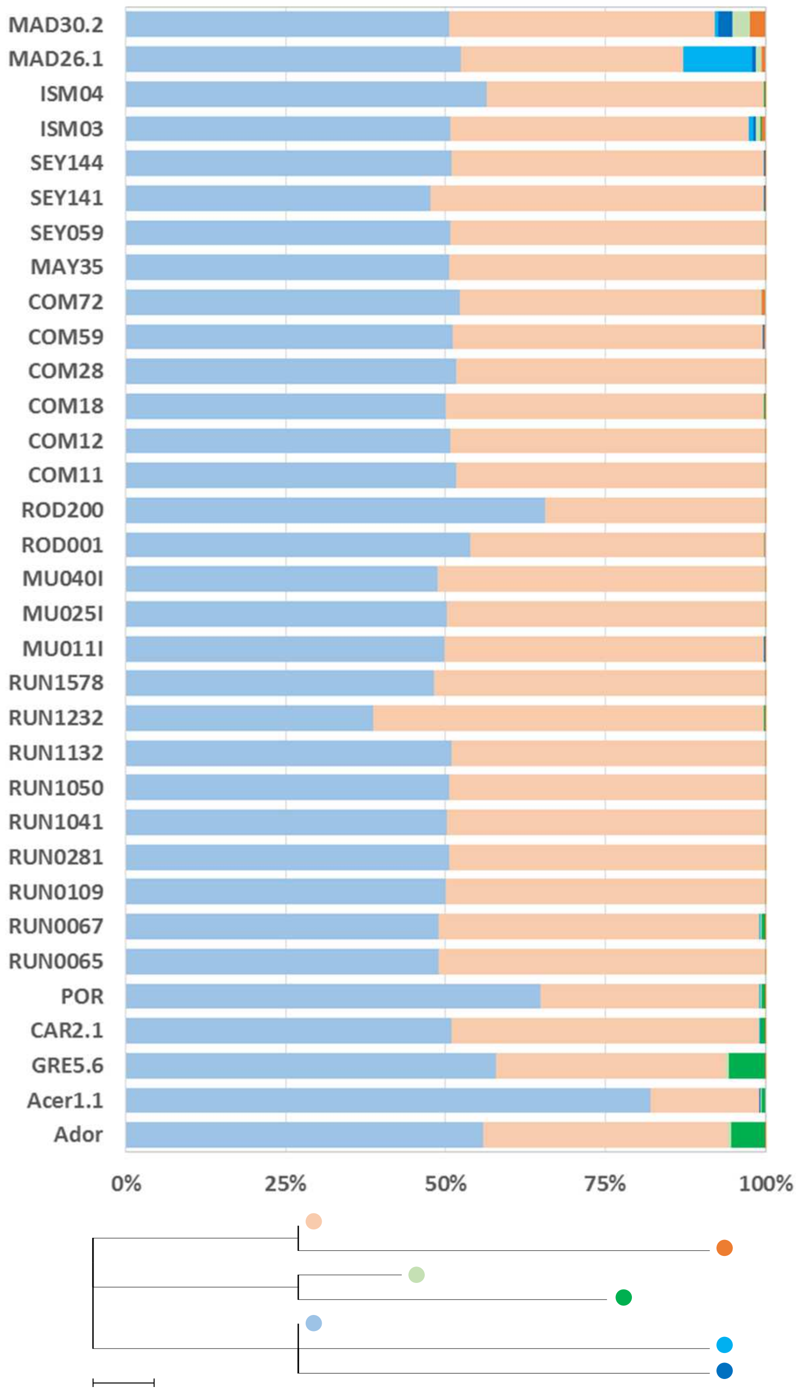

001CF (CR, translation initiation factor EIF2a)

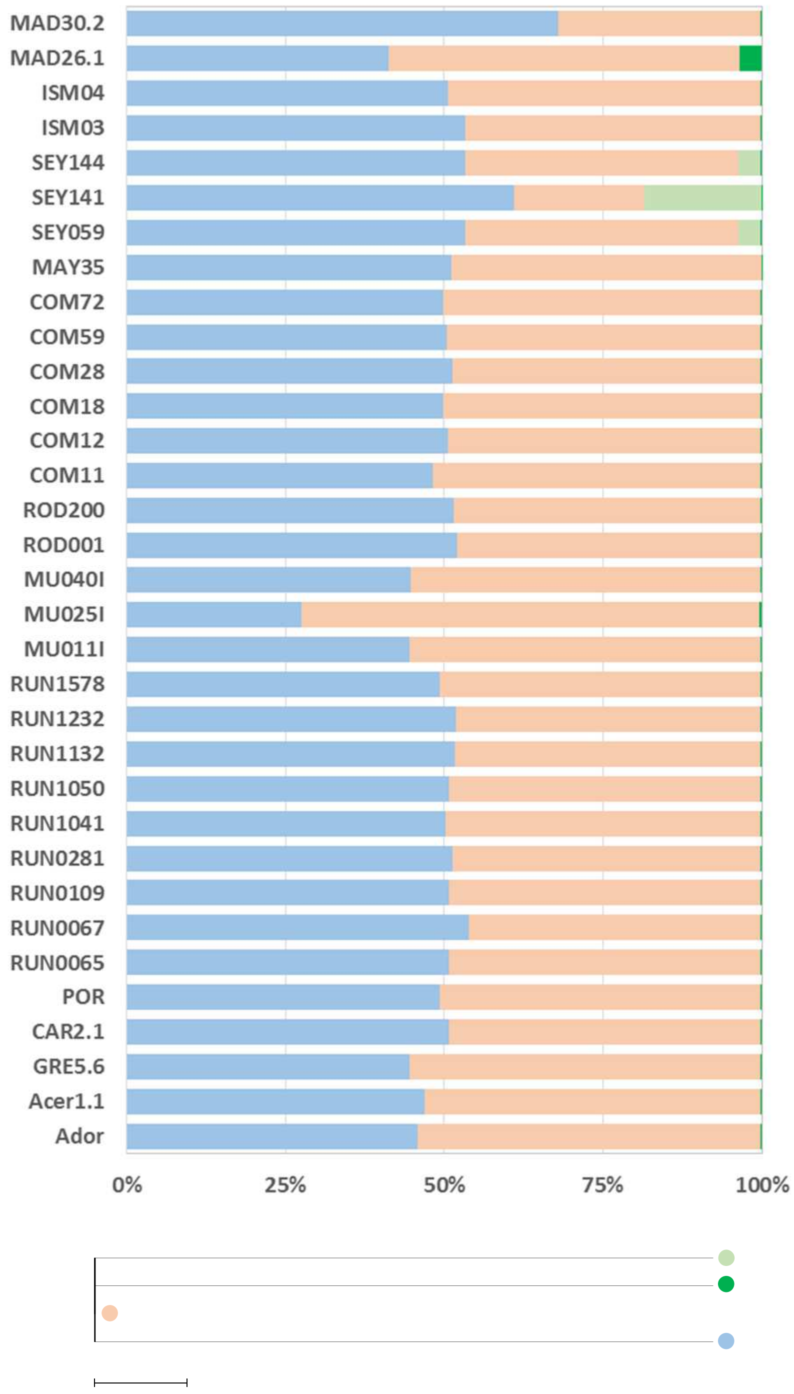

001CR (CR, translation initiation factor EIF2a)

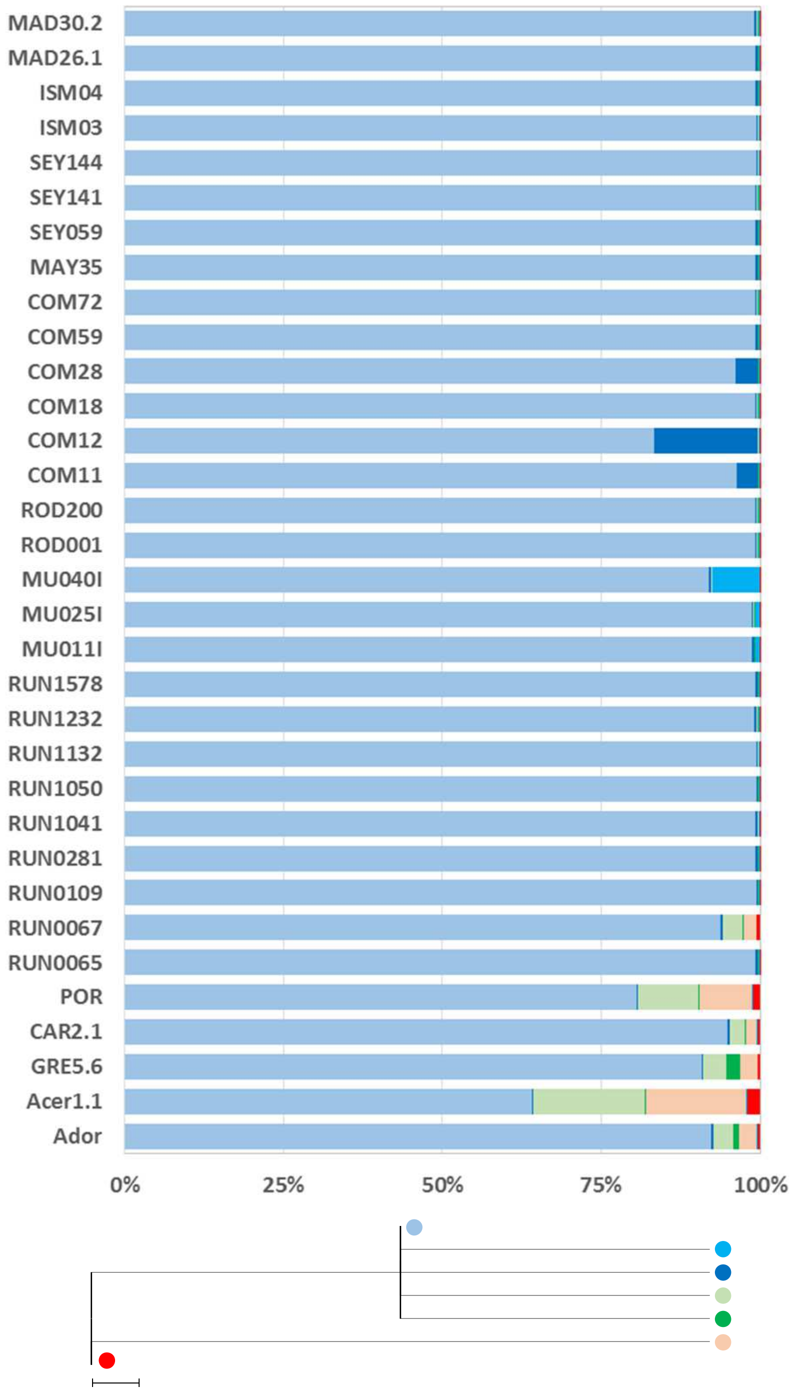

001DF (MS)

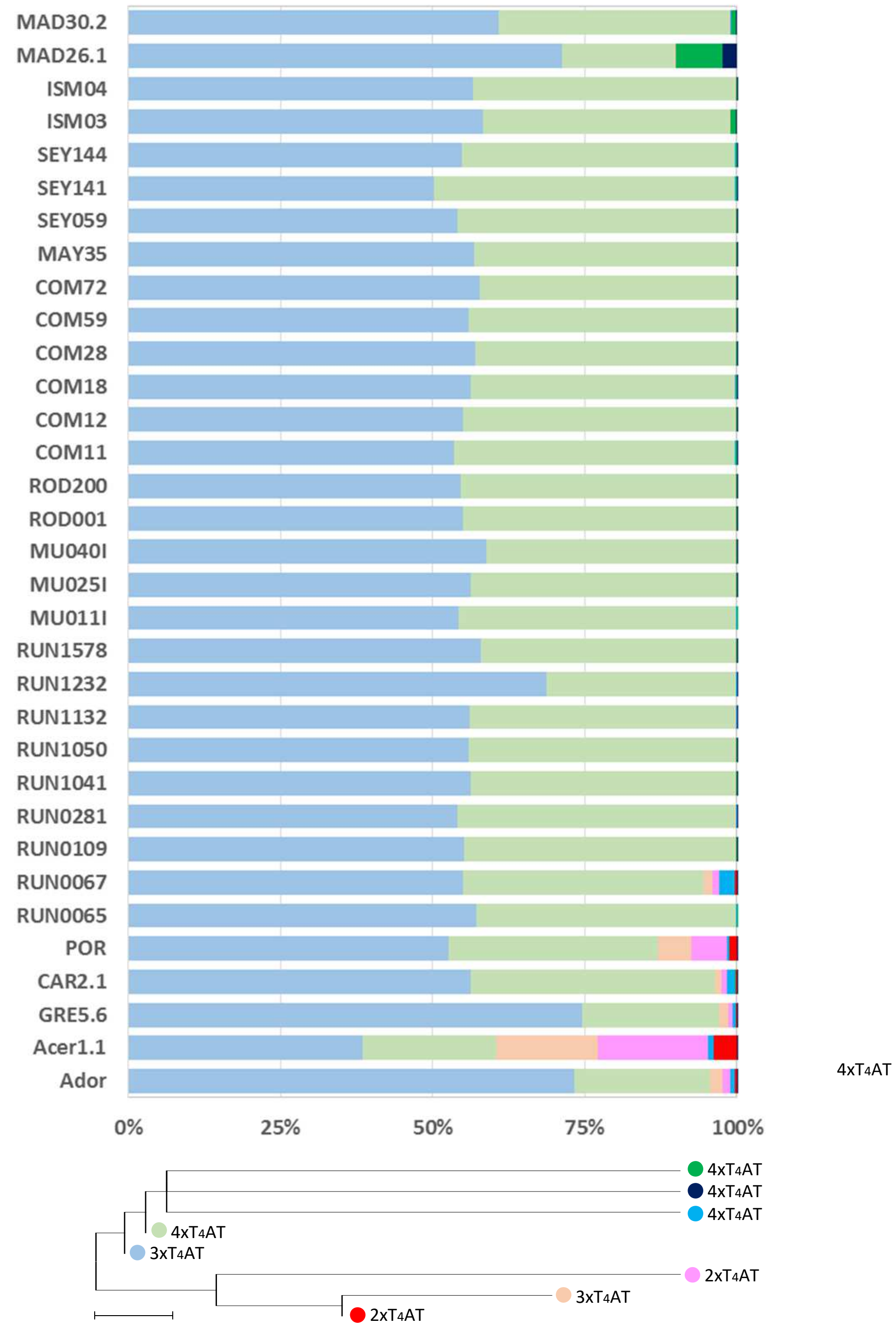

001DR (CR, ORF AAJ76_100020621)

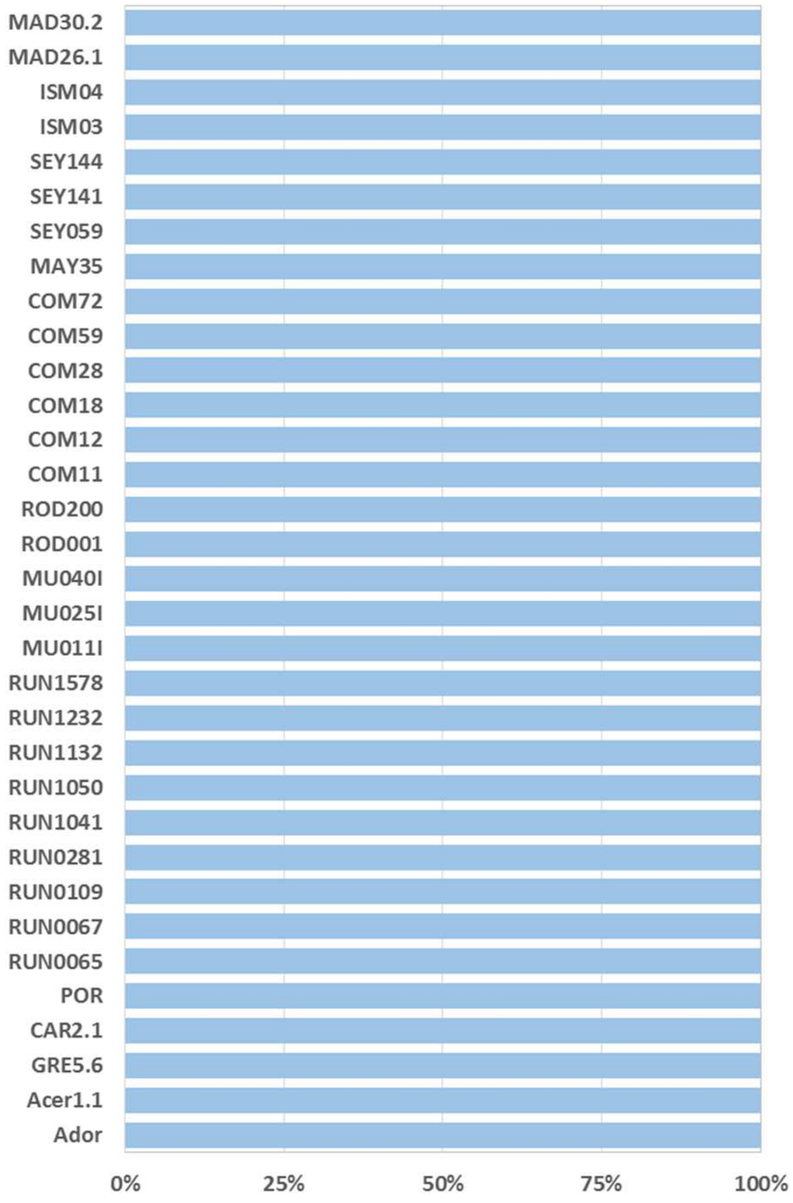

001EF (MS)

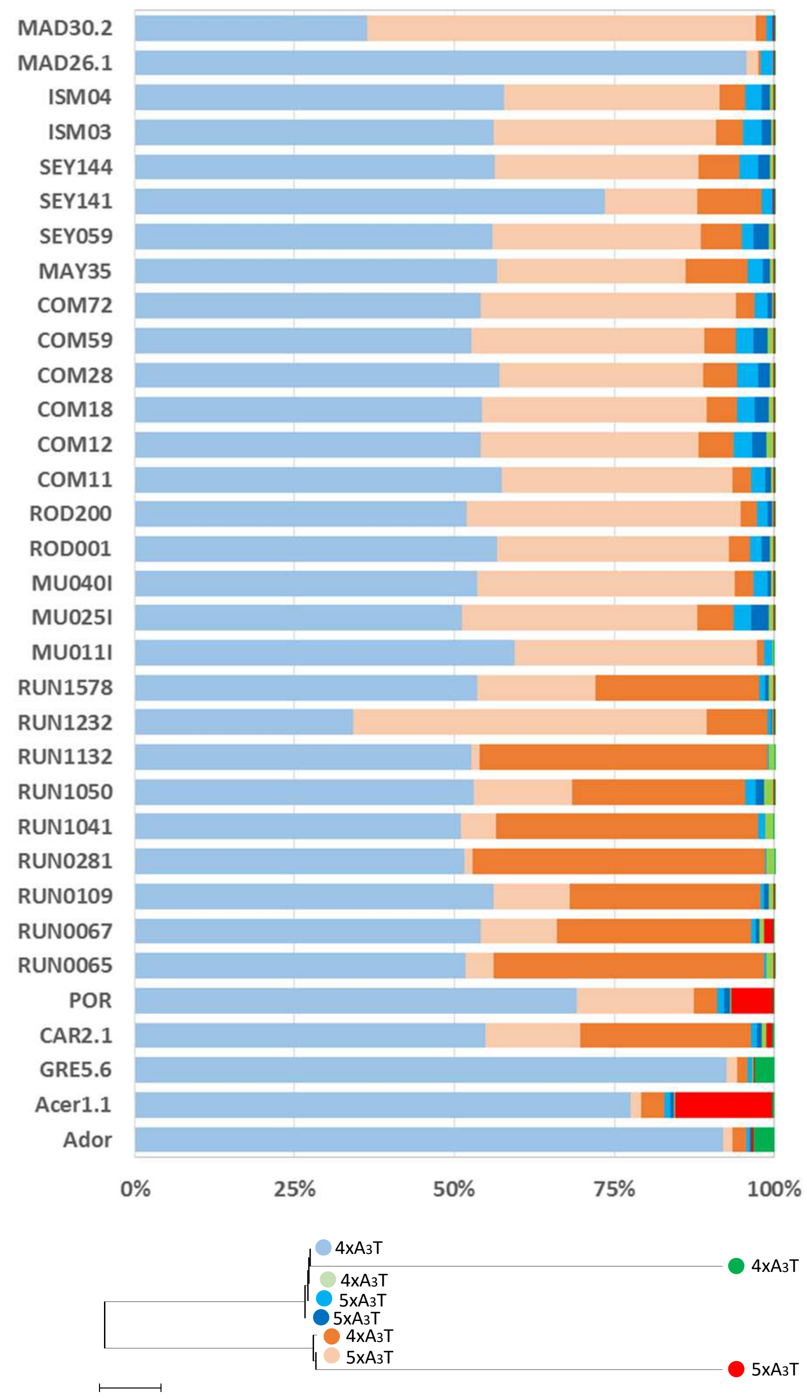

001ER (NC)

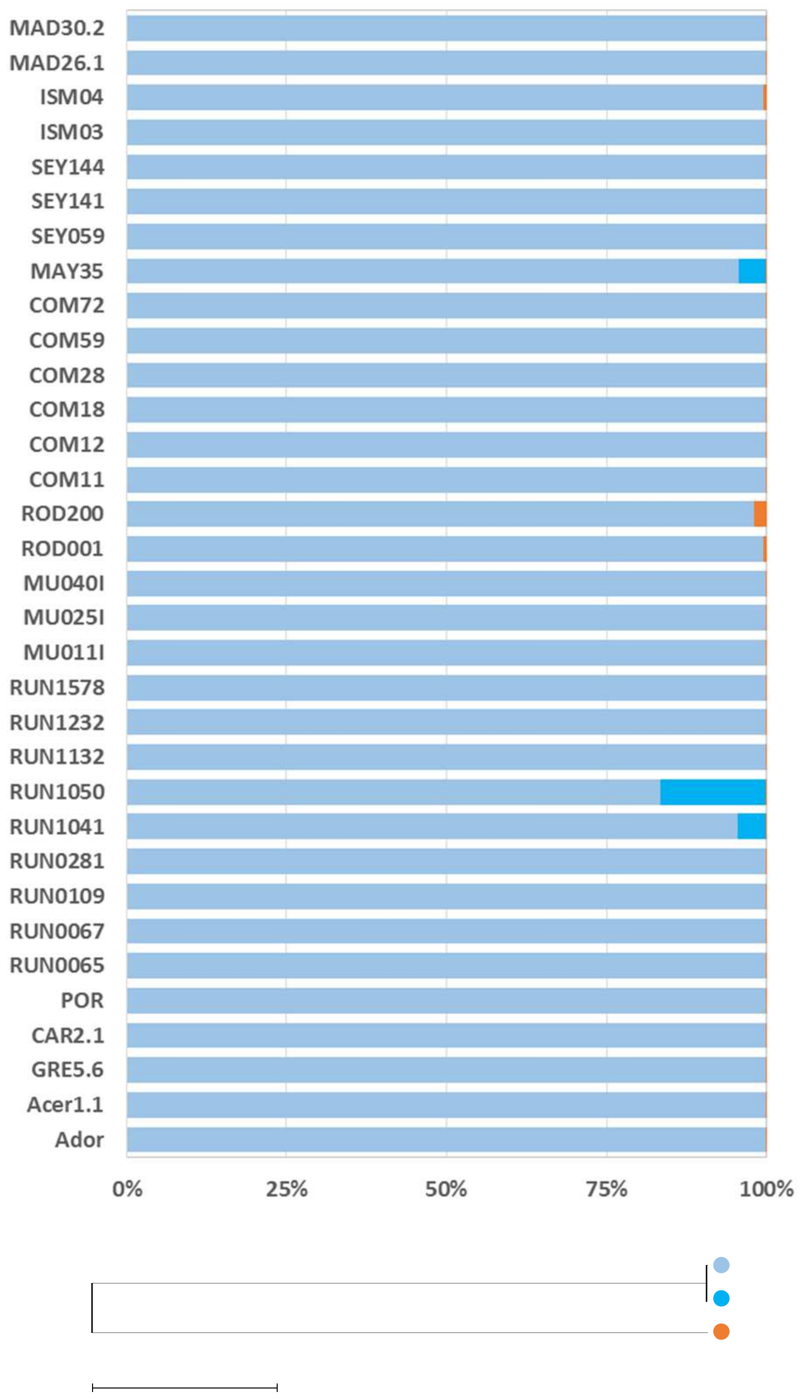

002AF (CR, spore wall protein SWP30)

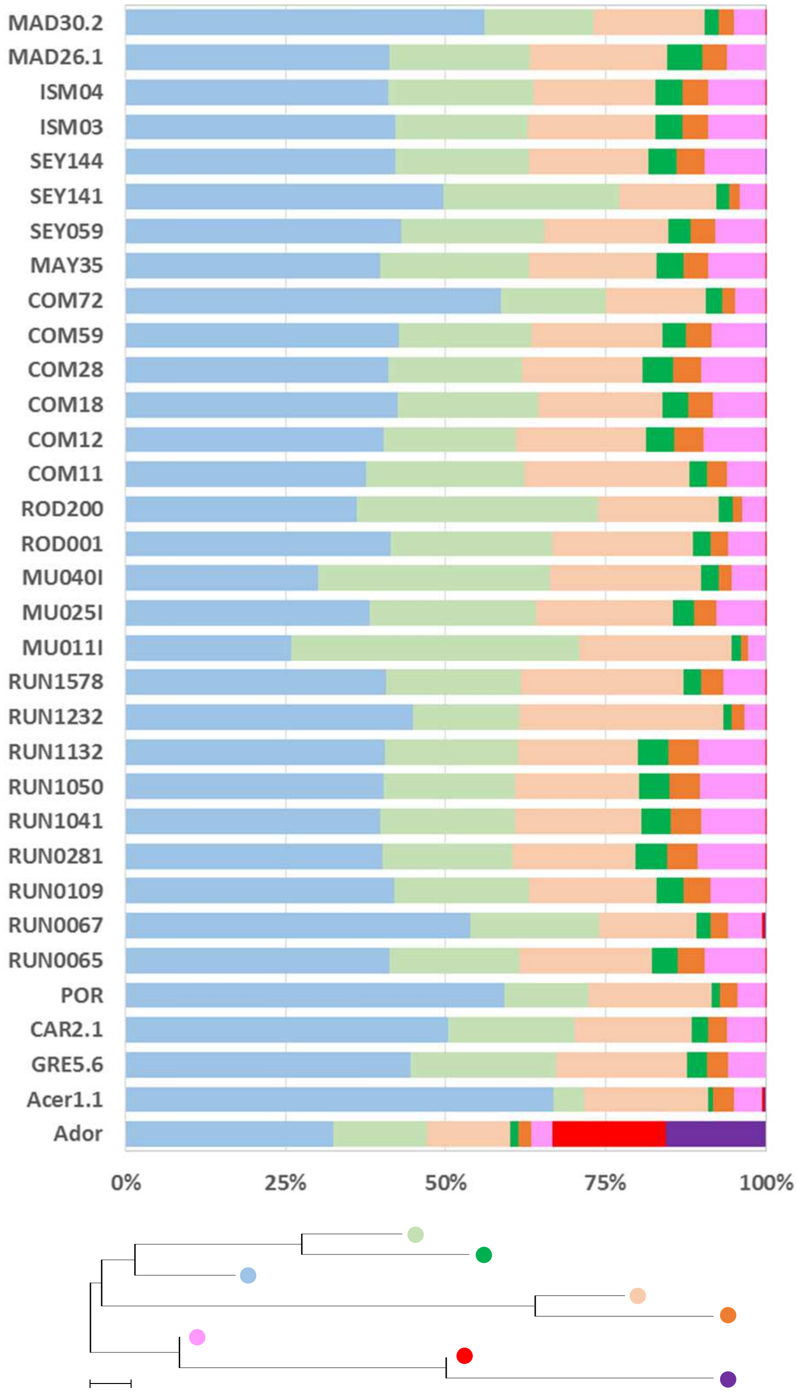

002AR (CR, spore wall protein SWP30)

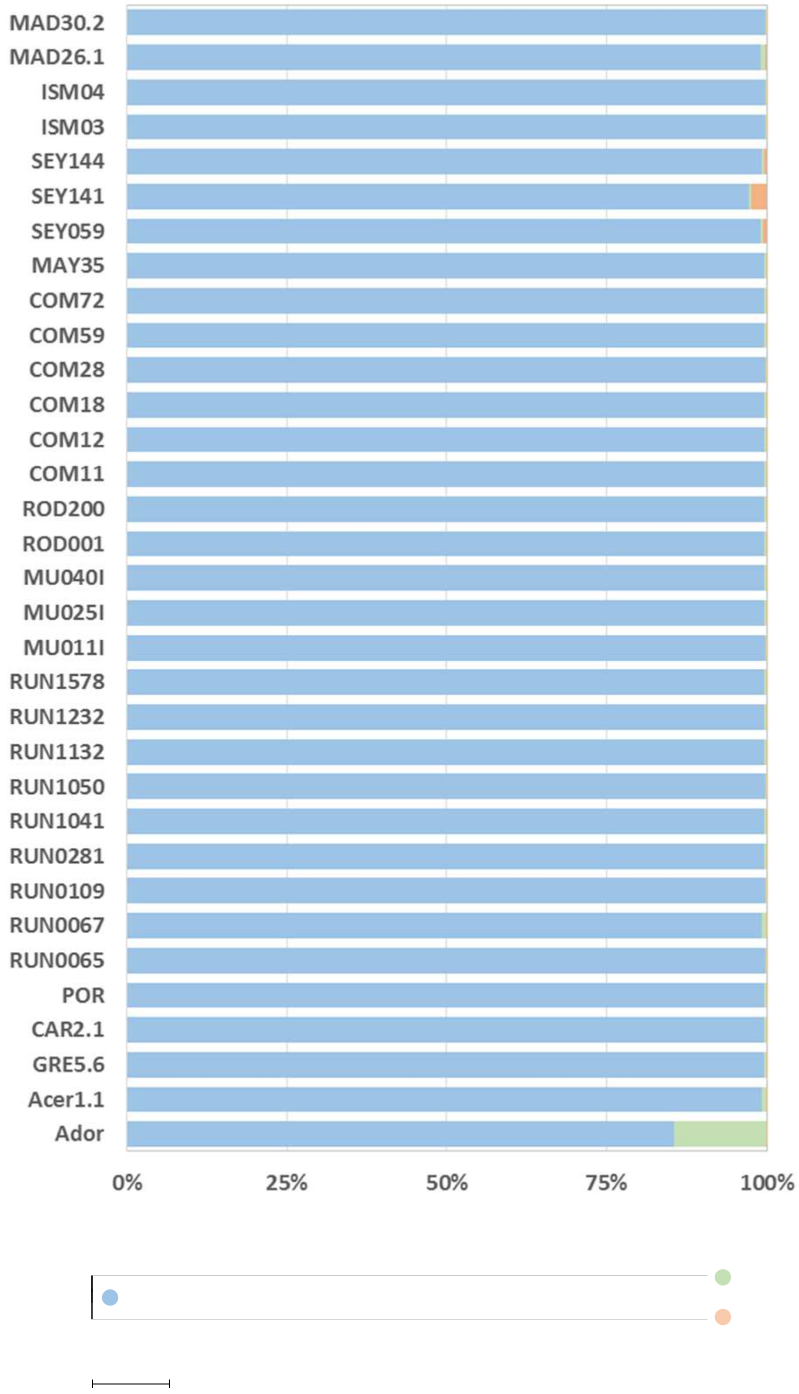

003AF (CR, in ORF AAJ76_3000124483)

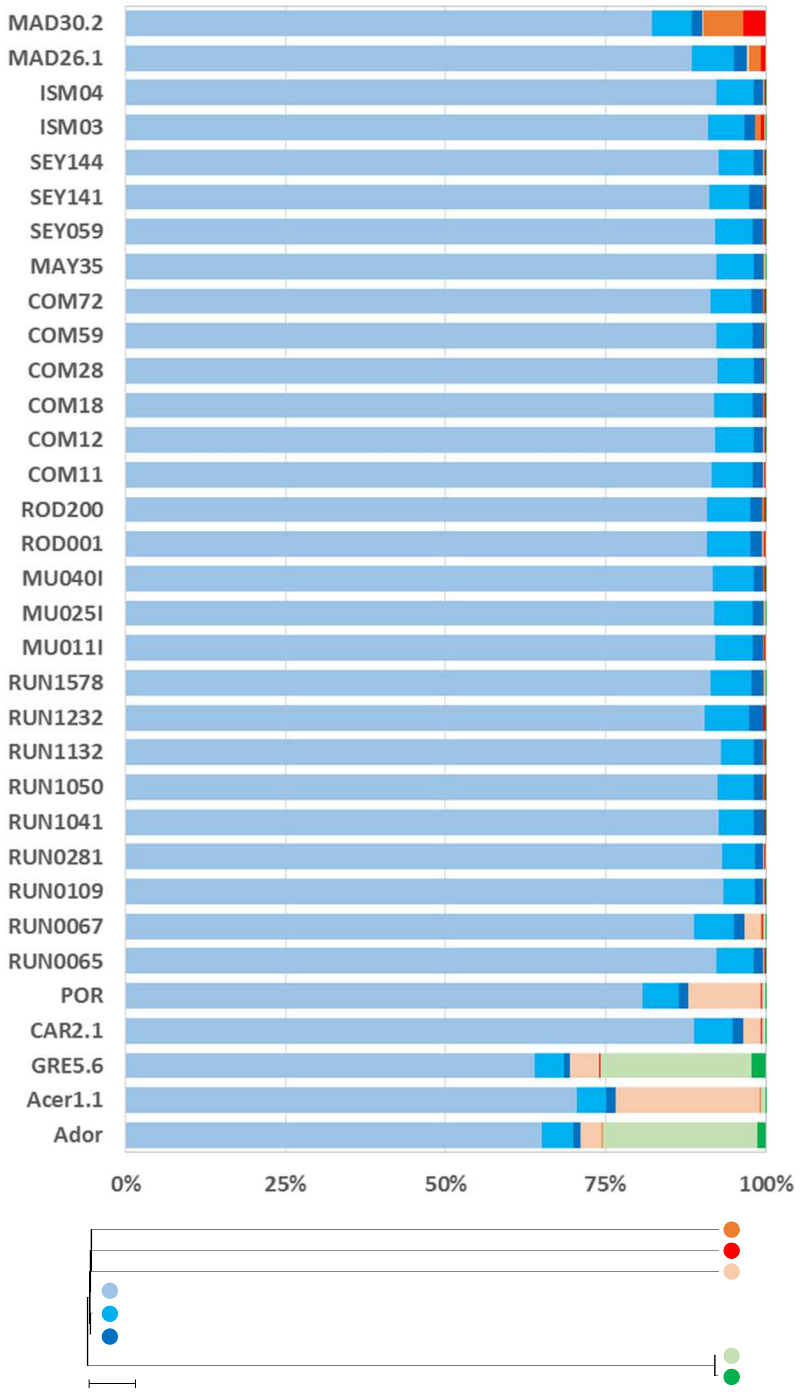

003AR (MS)

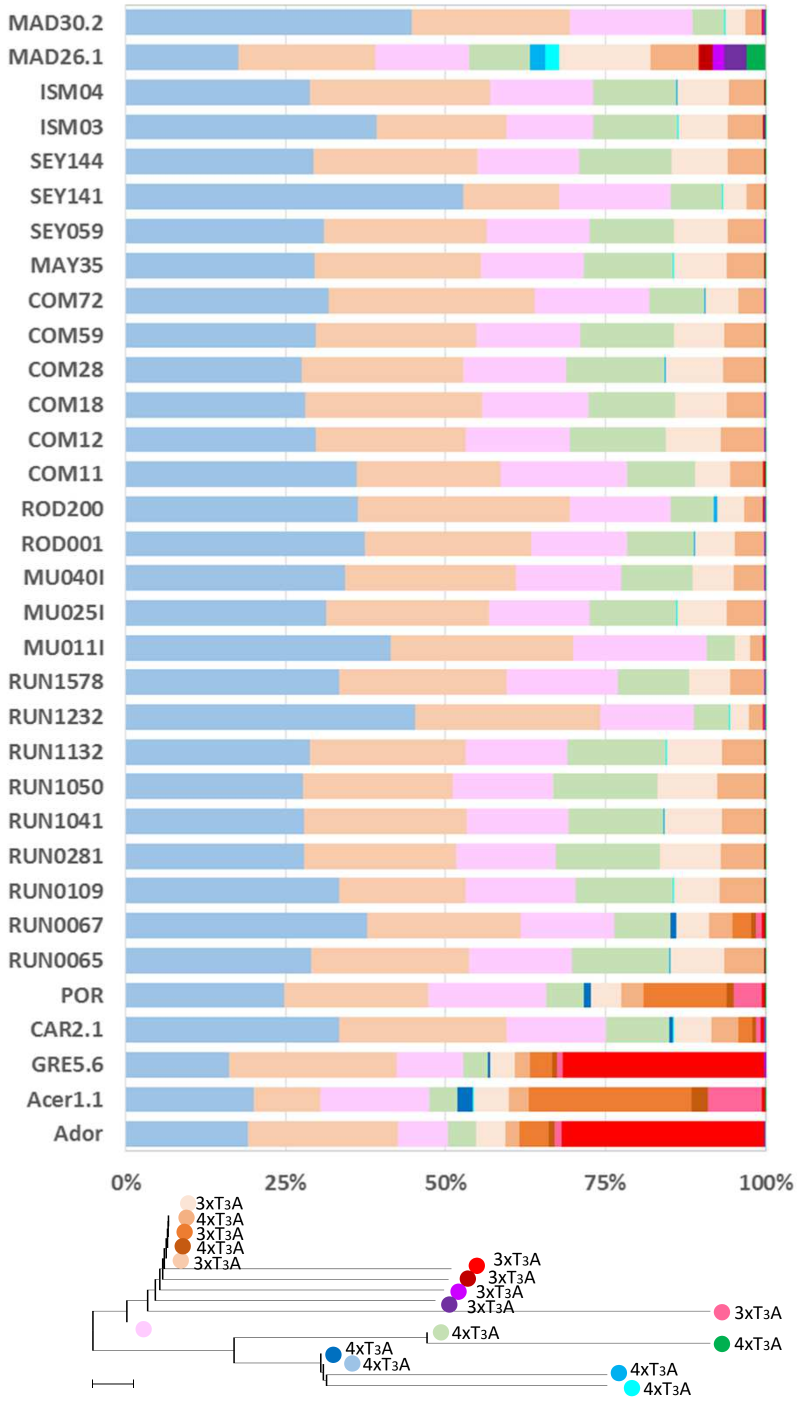

003BF (NC)

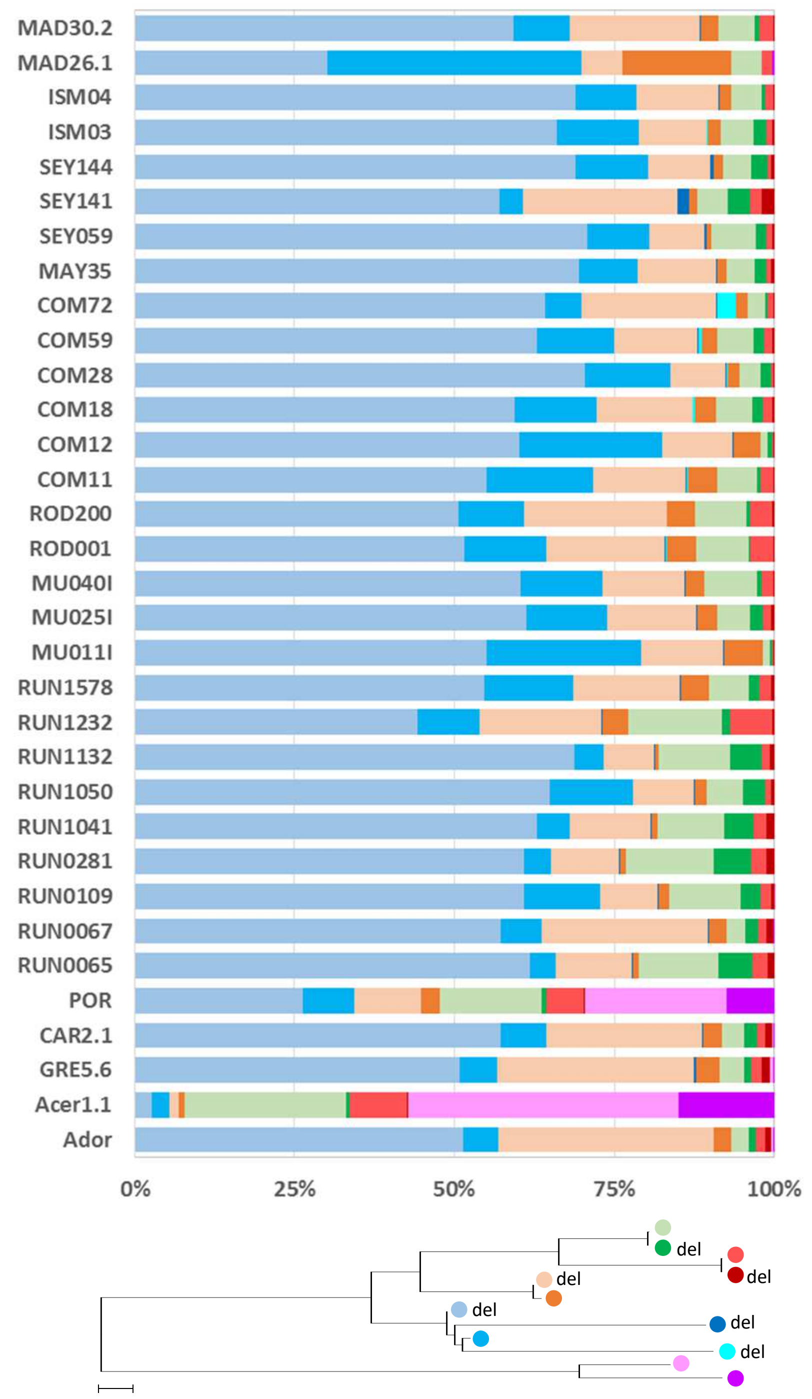

003BR (NC)

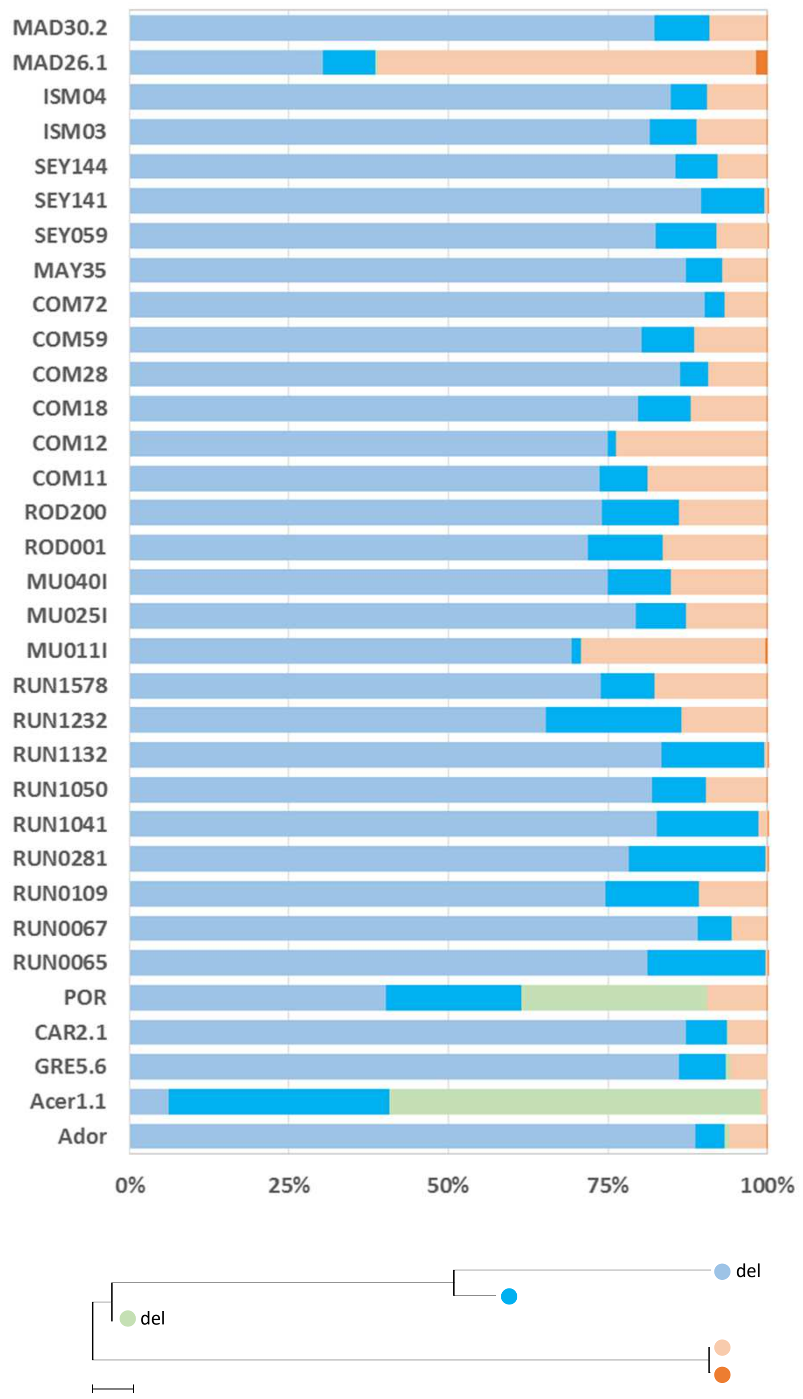

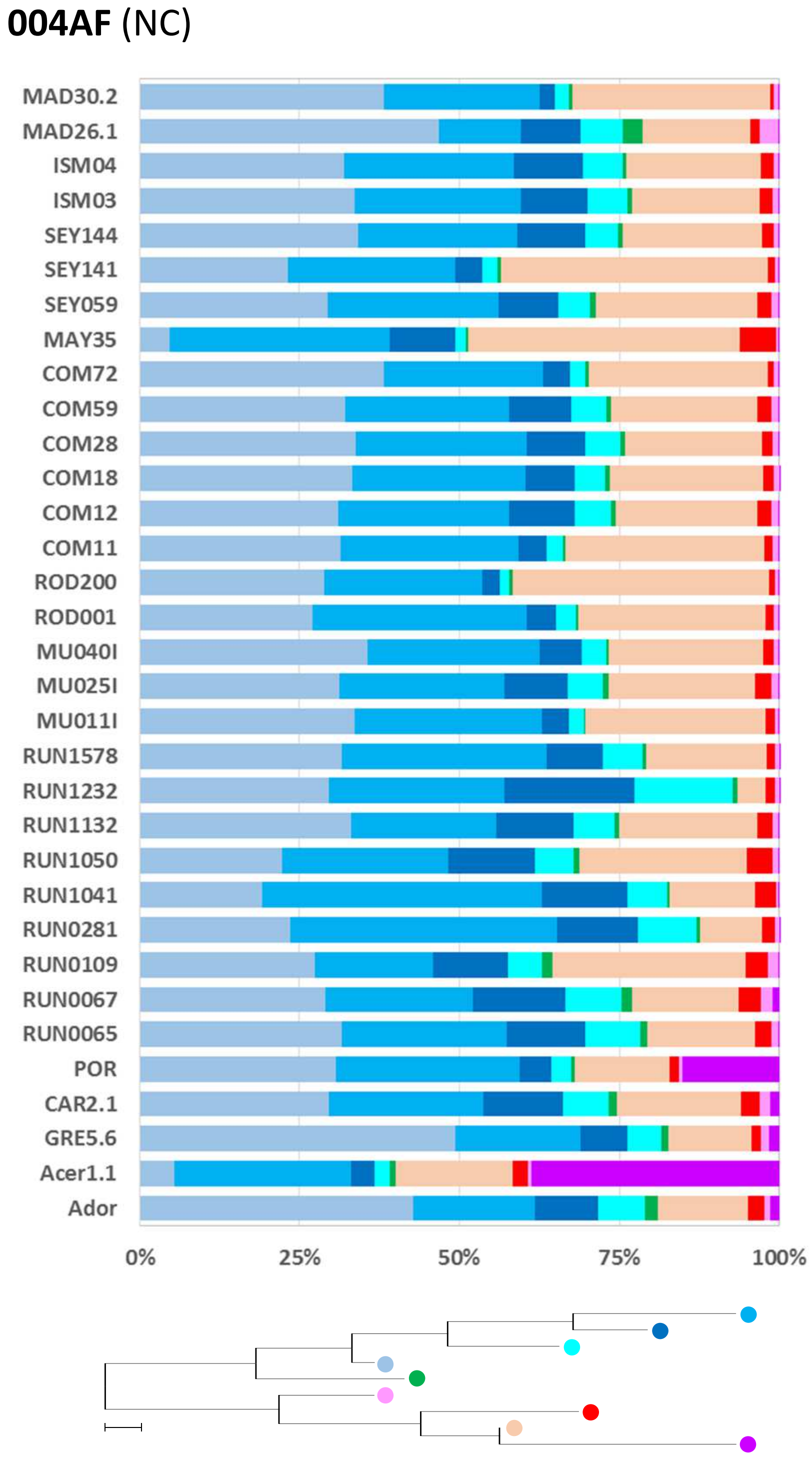
004AF (NC)
MAD30.2
MAD26.1
ISM04
ISM03
SEY144
SEY141
SEY059
MAY35
COM72
COM59
COM28
COM18
COM12
COM11
ROD200
ROD001
MU040I
MU025I
MU011I
RUN1578
RUN1232
RUN1132
RUN1050
RUN1041
RUN0281
RUN0109
RUN0067
RUN0065
POR
CAR2.1
GRE5.6
Acer1.1
Ador
0%
25%
50%
75%
100%

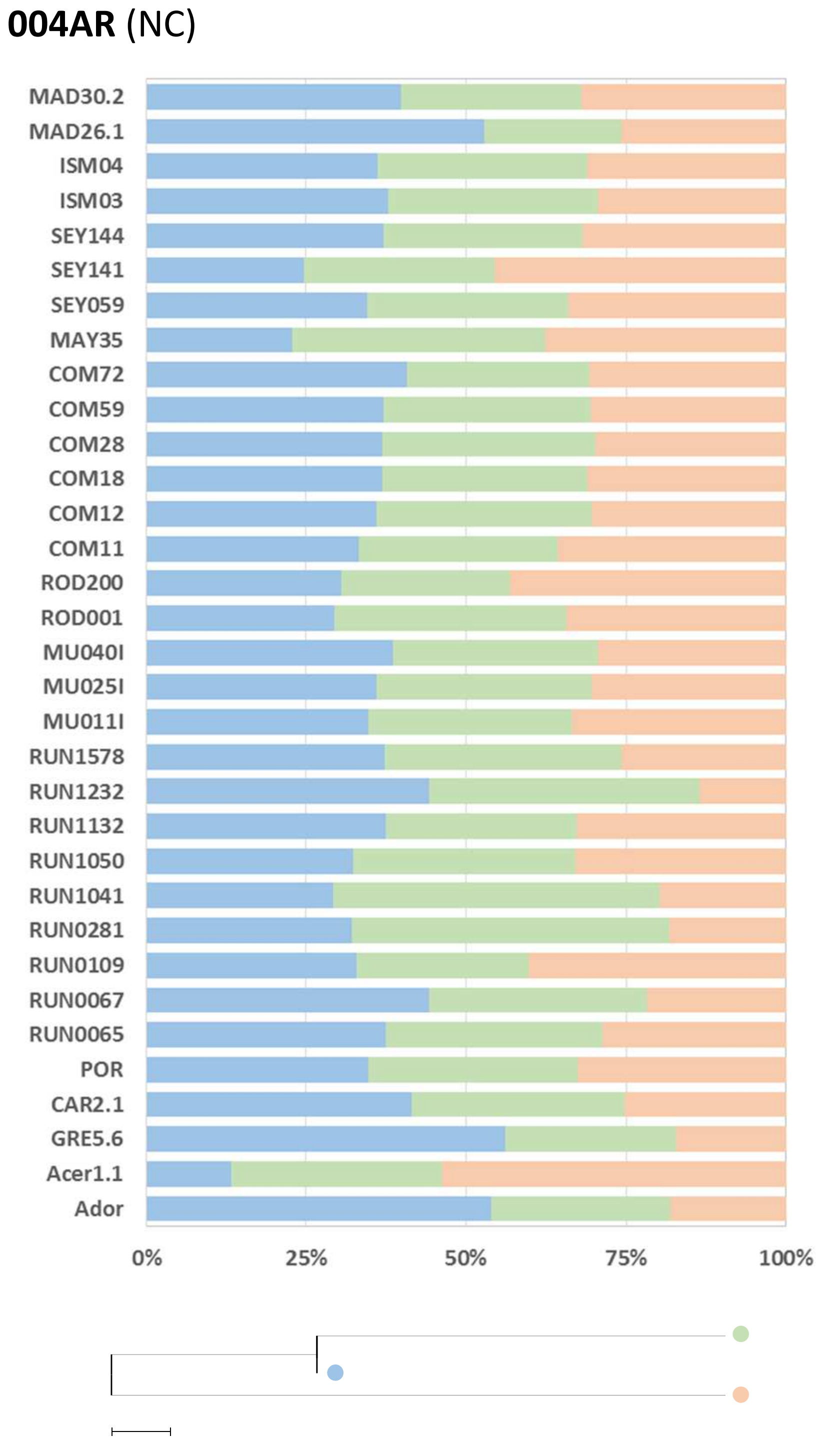
004AR (NC)
MAD30.2
MAD26.1
ISM04
ISM03
SEY144
SEY141
SEY059
MAY35
COM72
COM59
COM28
COM18
COM12
COM11
ROD200
ROD001
MU040I
MU025I
MU011I
RUN1578
RUN1232
RUN1132
RUN1050
RUN1041
RUN0281
RUN0109
RUN0067
RUN0065
POR
CAR2.1
GRE5.6
Acer1.1
Ador
0%
25%
50%
75%
100%

004BF (MS)

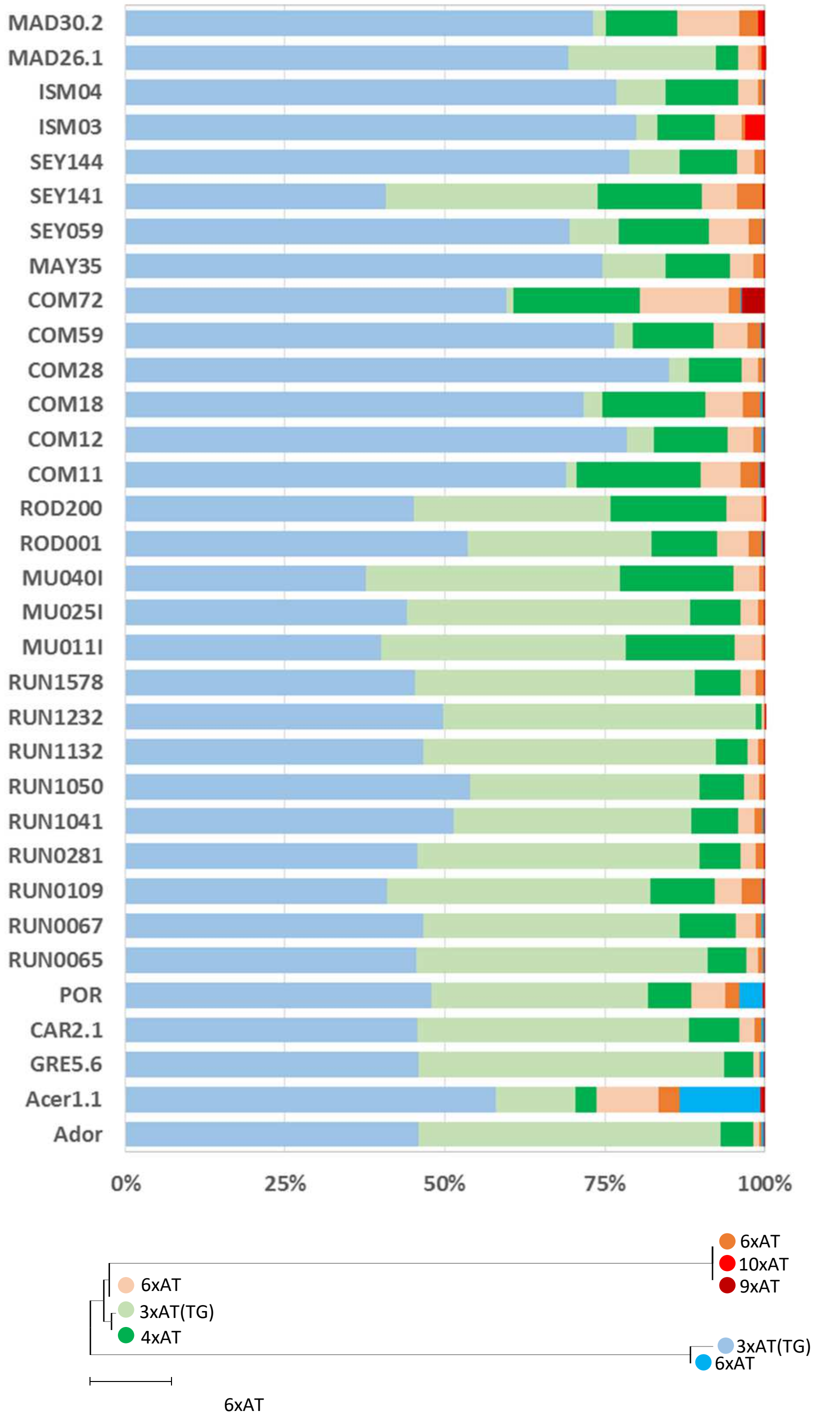

004BR (NC)

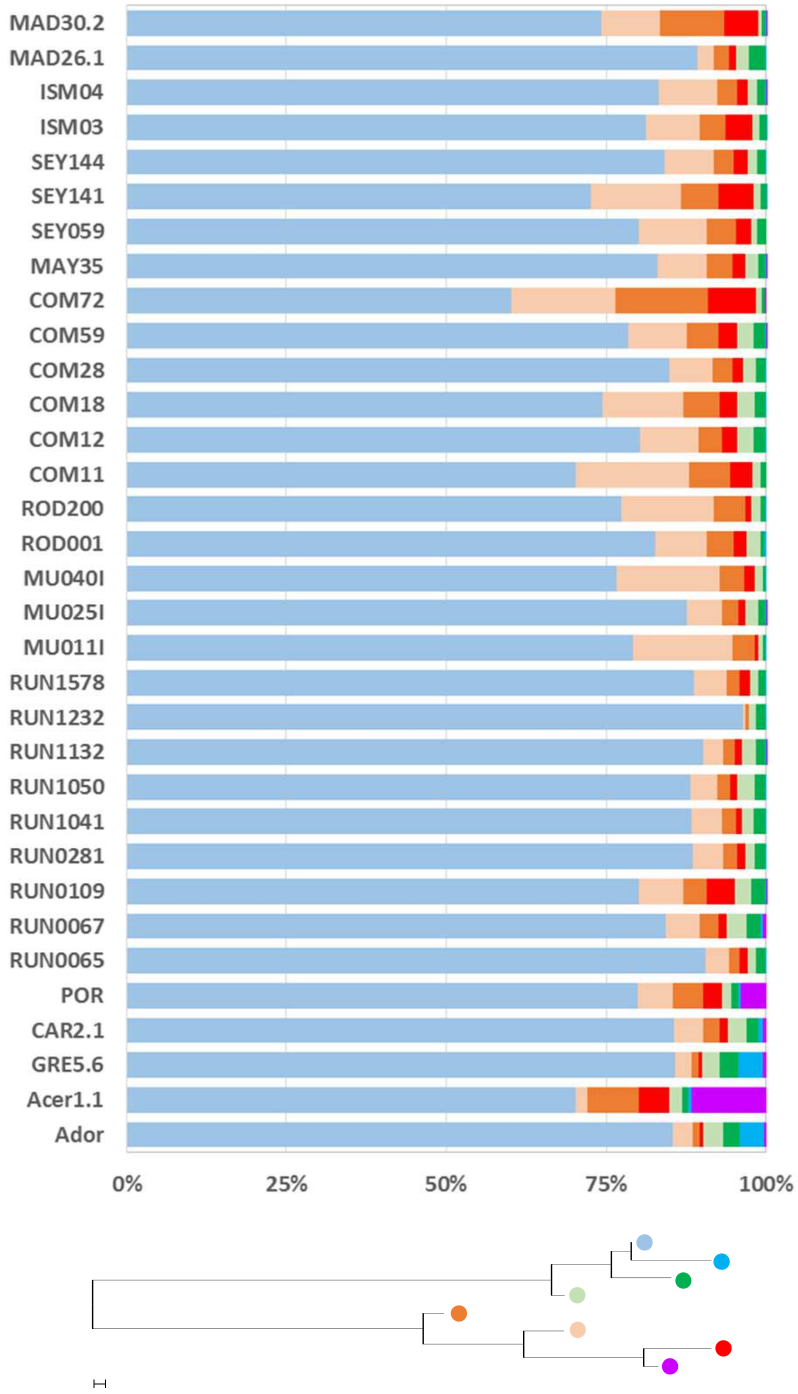

005AF (CR, phosphoacetyl-glucosamine mutase)

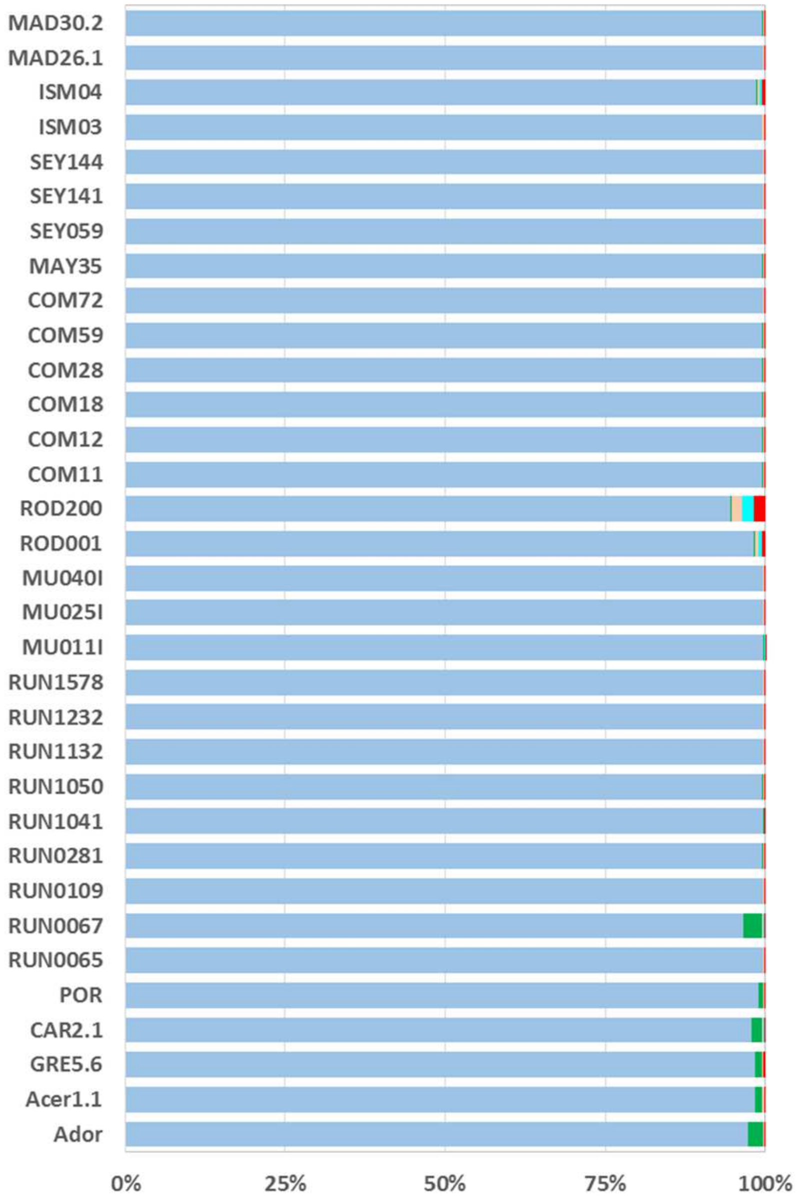

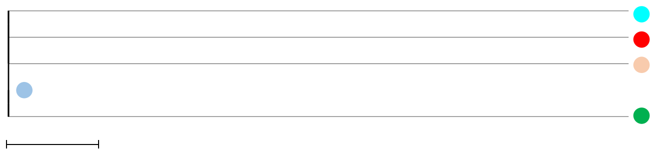

005AR (CR, phosphoacetyl-glucosamine mutase)

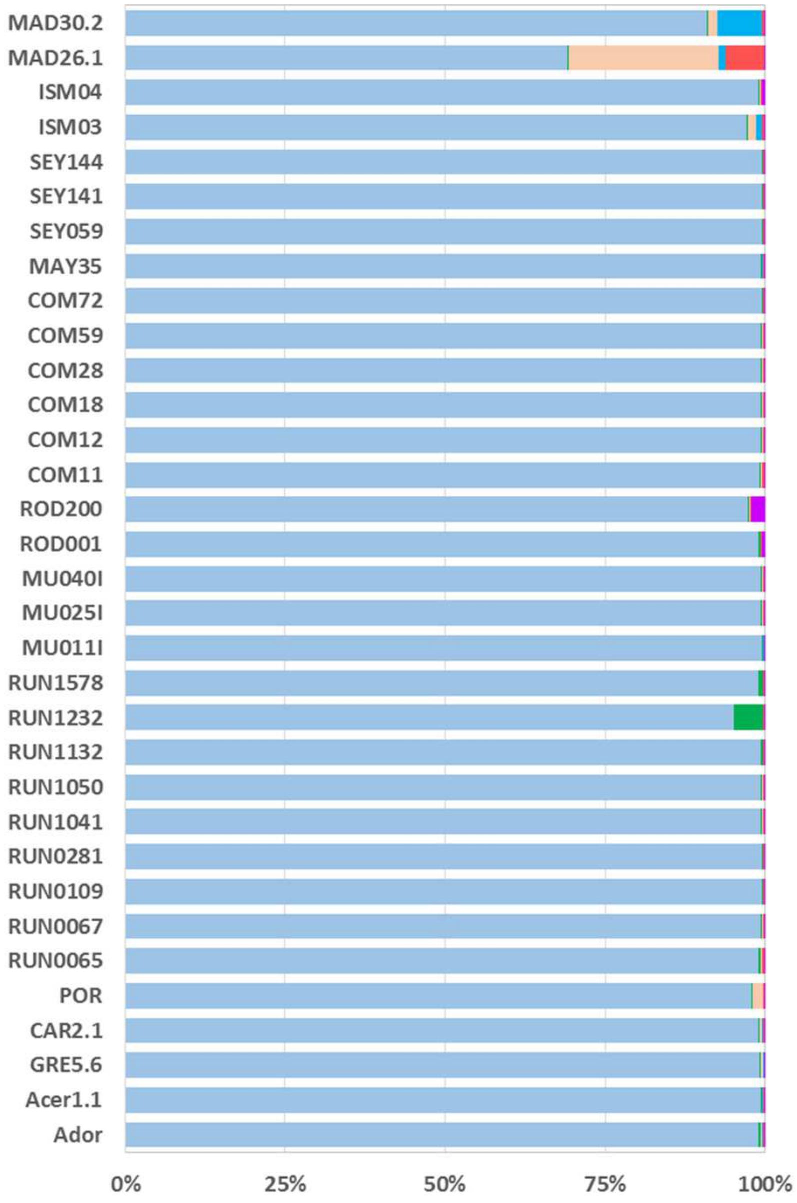

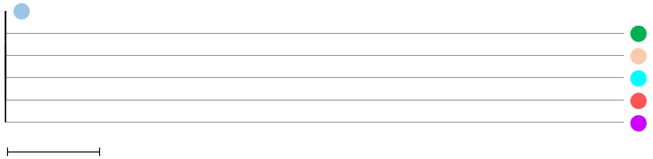

005BF (CR, hypothetical actin)

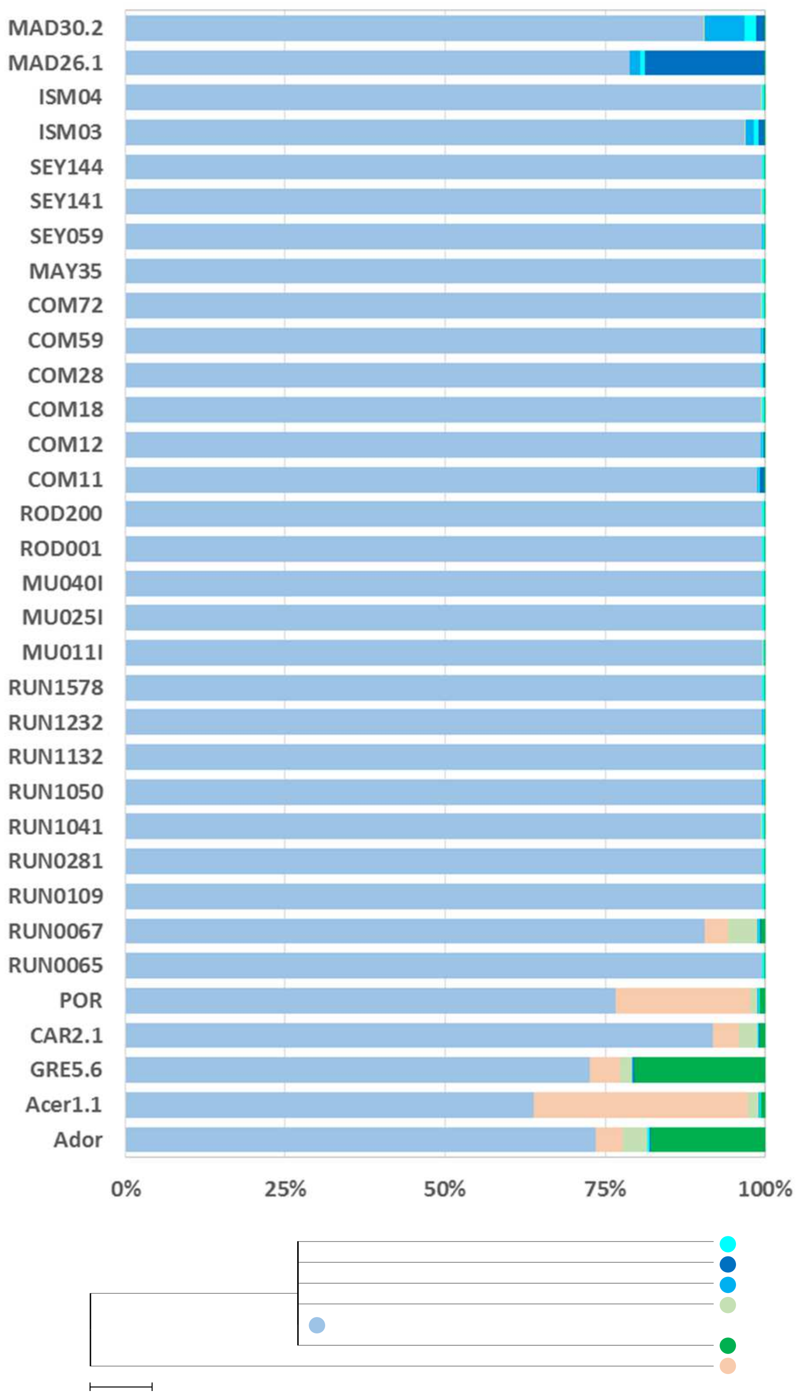

005BR (CR, hypothetical actin)

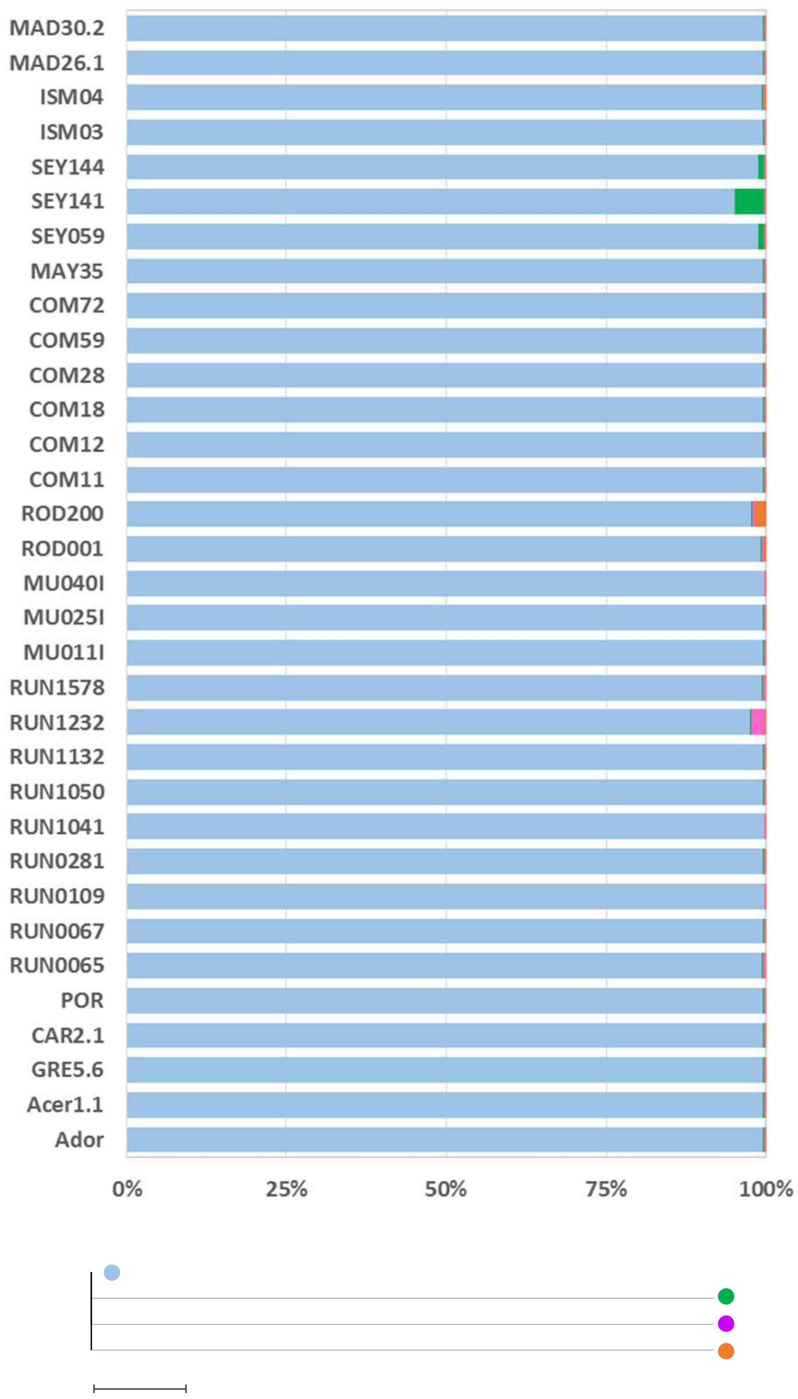

006AF (NC)

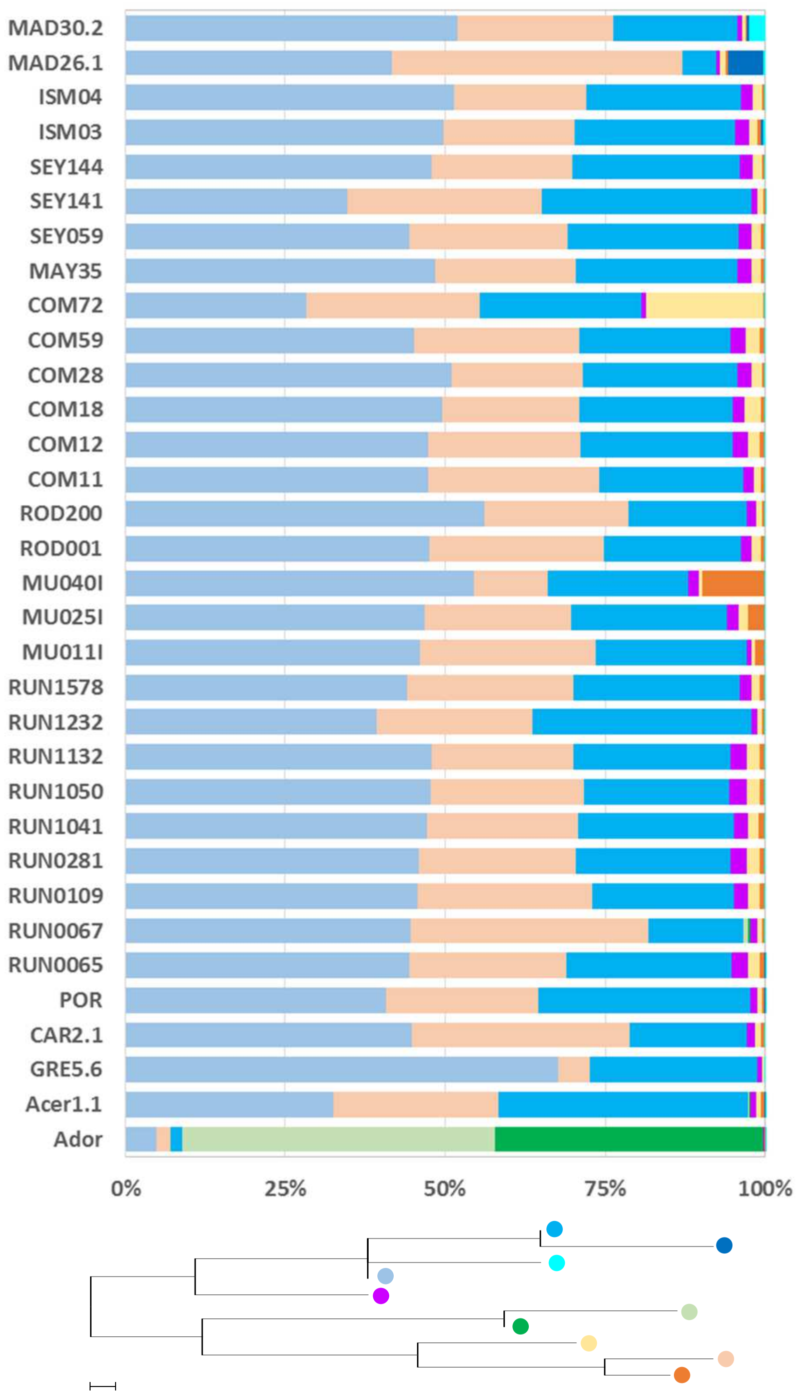

006AR (NC)

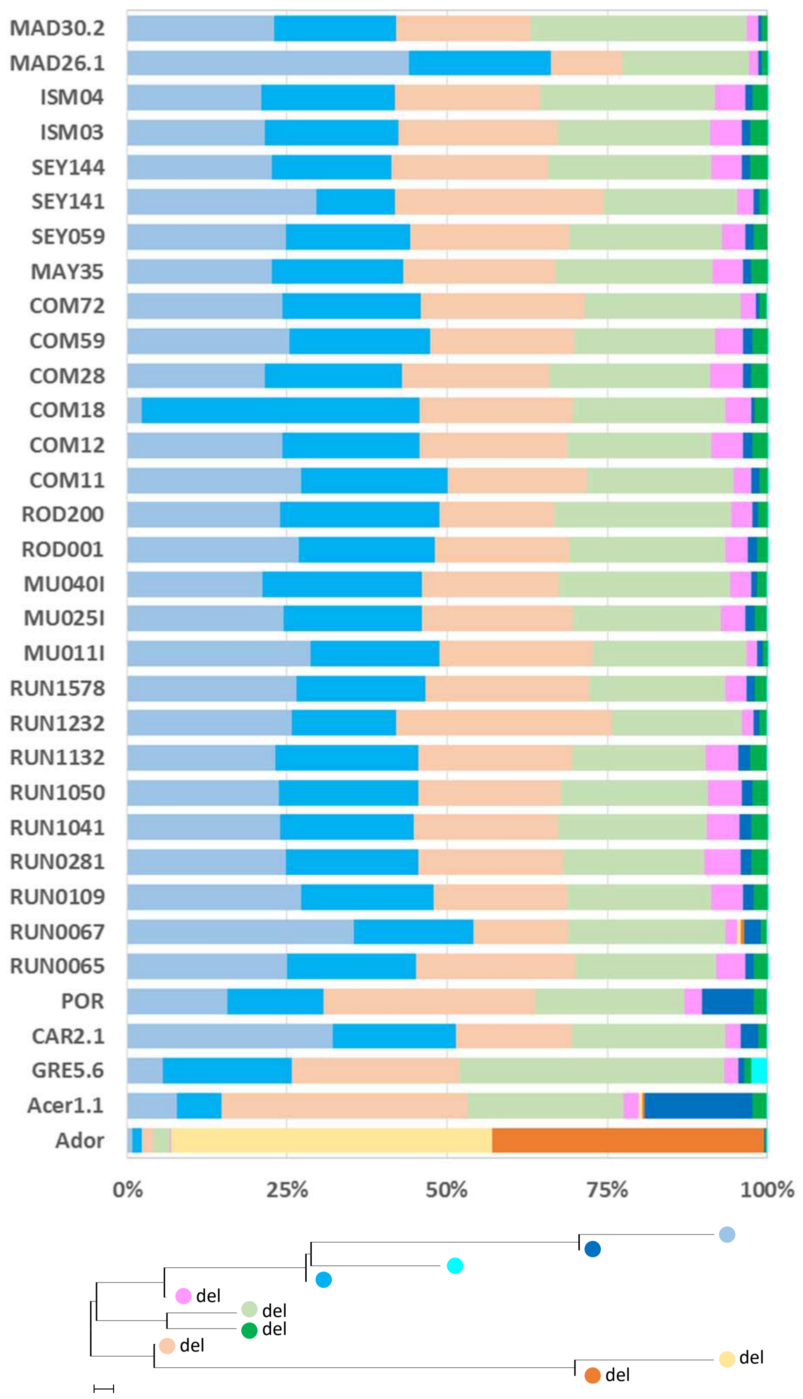

009AF (CR, in ORF AAJ76_900012560)

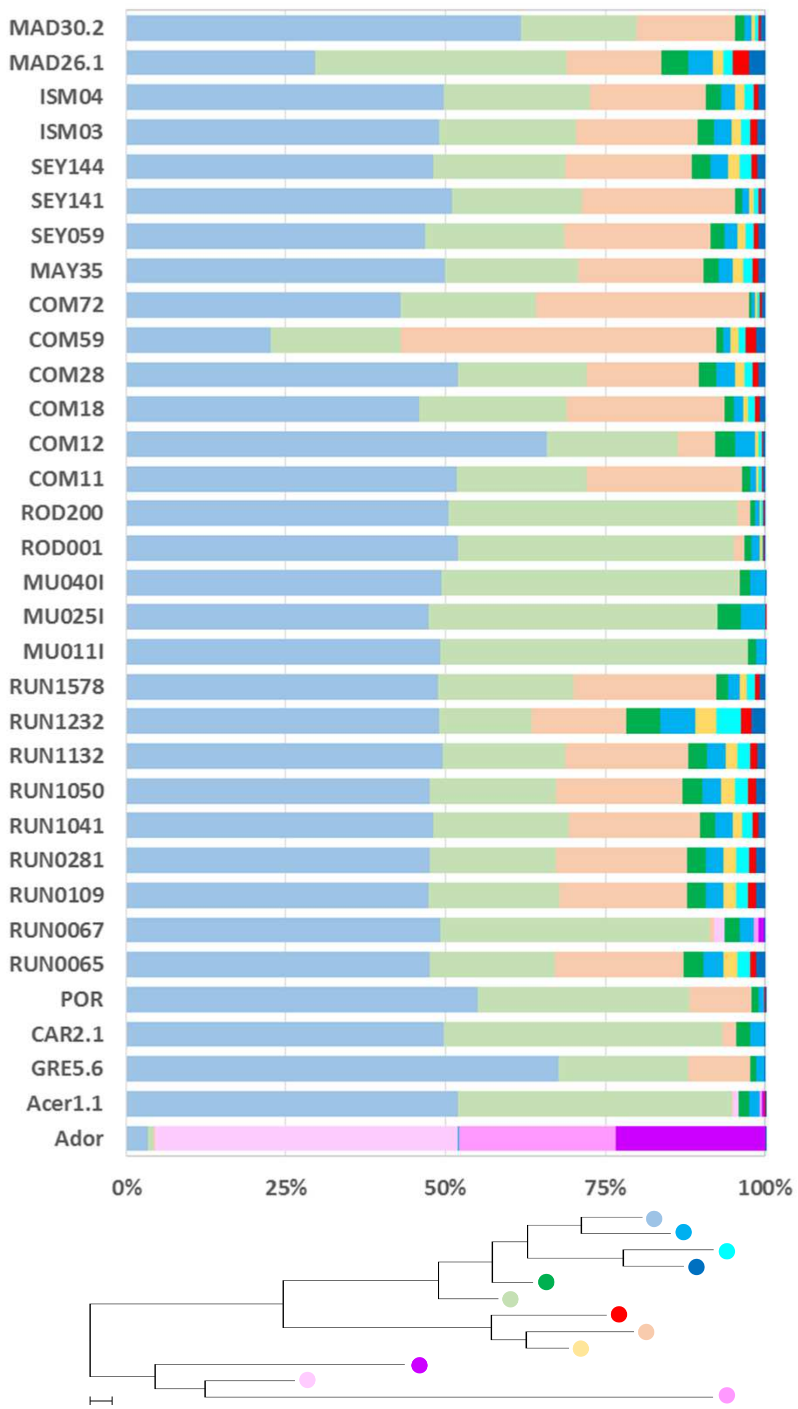

009AR (CR, in ORF AAJ76_900012560)

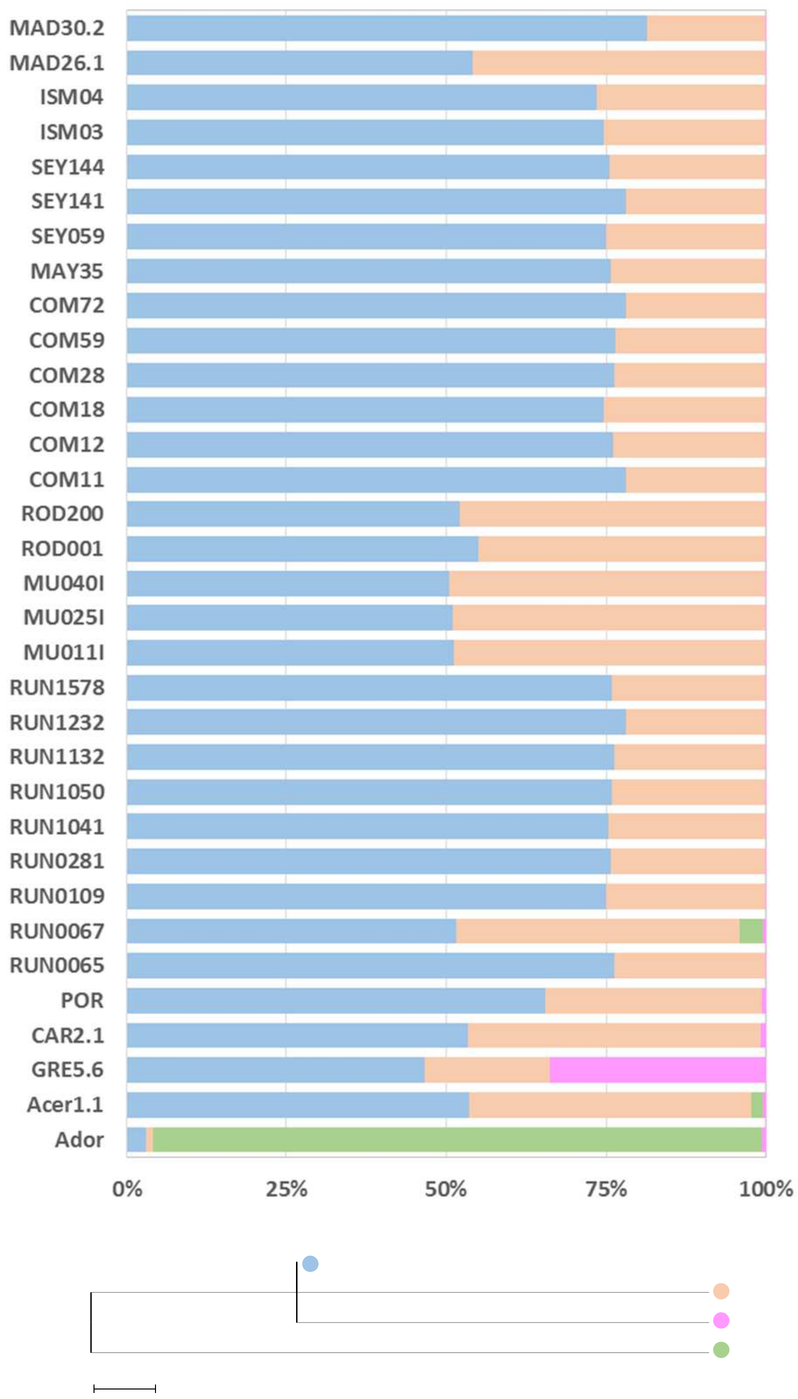

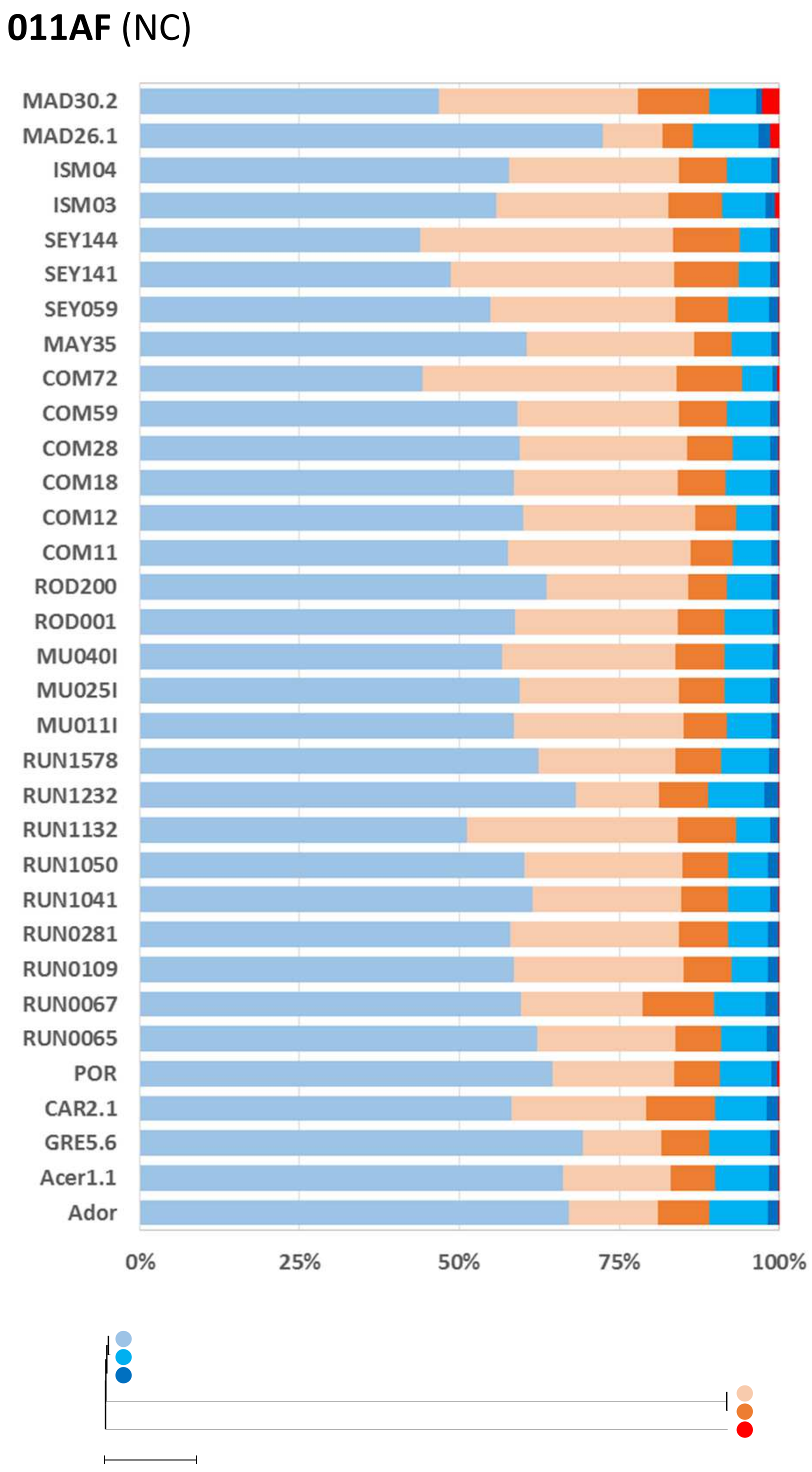

011AF (NC)
MAD30.2
MAD26.1
ISM04
ISM03
SEY144
SEY141
SEY059
MAY35
COM72
COM59
COM28
COM18
COM12
COM11
ROD200
ROD001
MU040I
MU025I
MU011I
RUN1578
RUN1232
RUN1132
RUN1050
RUN1041
RUN0281
RUN0109
RUN0067
RUN0065
POR
CAR2.1
GRE5.6
Acer1.1
Ador
0%
25%
50%
75%
100%

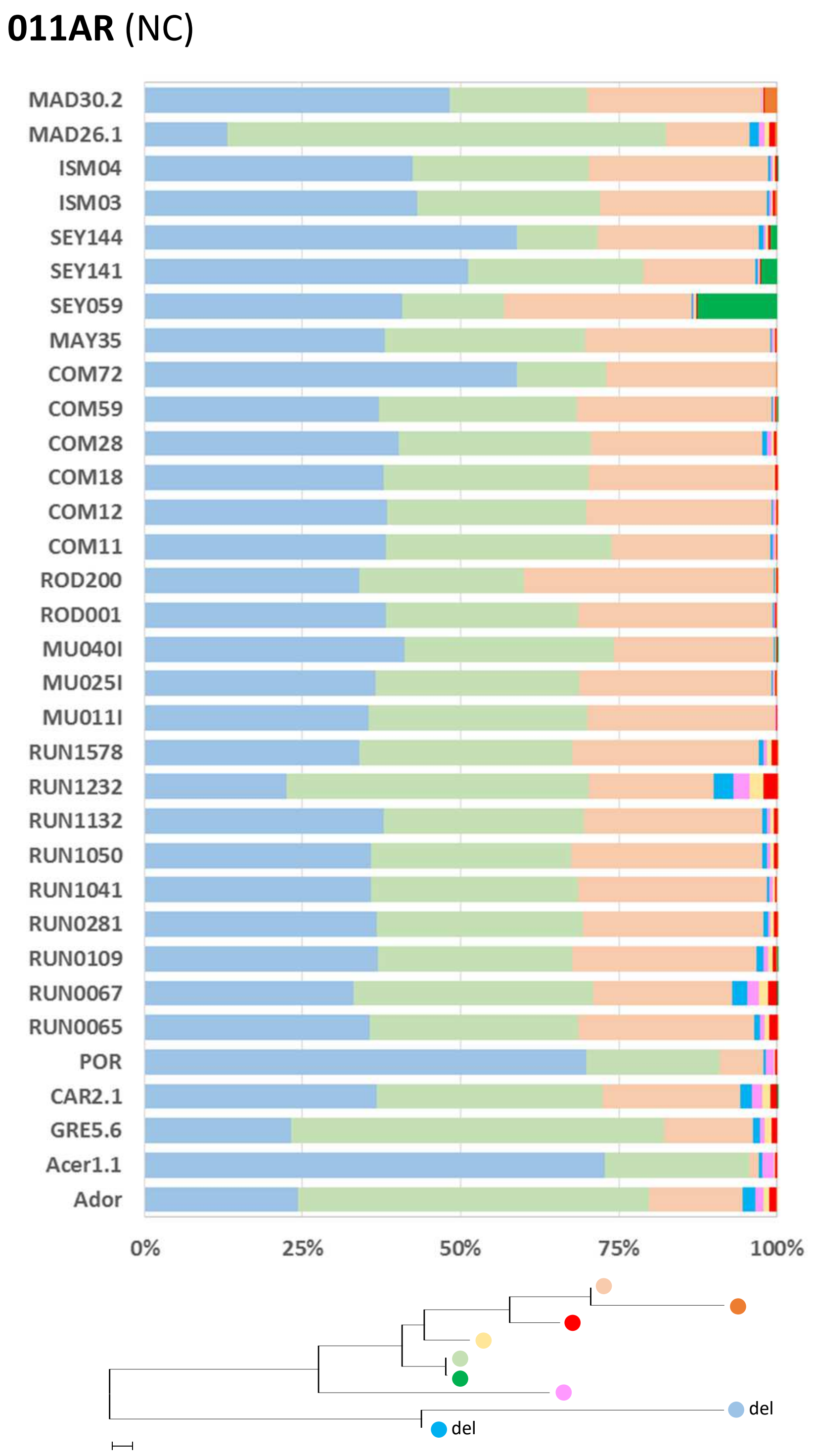

011AR (NC)
MAD30.2
MAD26.1
ISM04
ISM03
SEY144
SEY141
SEY059
MAY35
COM72
COM59
COM28
COM18
COM12
COM11
ROD200
ROD001
MU040I
MU025I
MU011I
RUN1578
RUN1232
RUN1132
RUN1050
RUN1041
RUN0281
RUN0109
RUN0067
RUN0065
POR
CAR2.1
GRE5.6
Acer1.1
Ador
0%
25%
50%
75%
100%
del
del

011BF (NC)

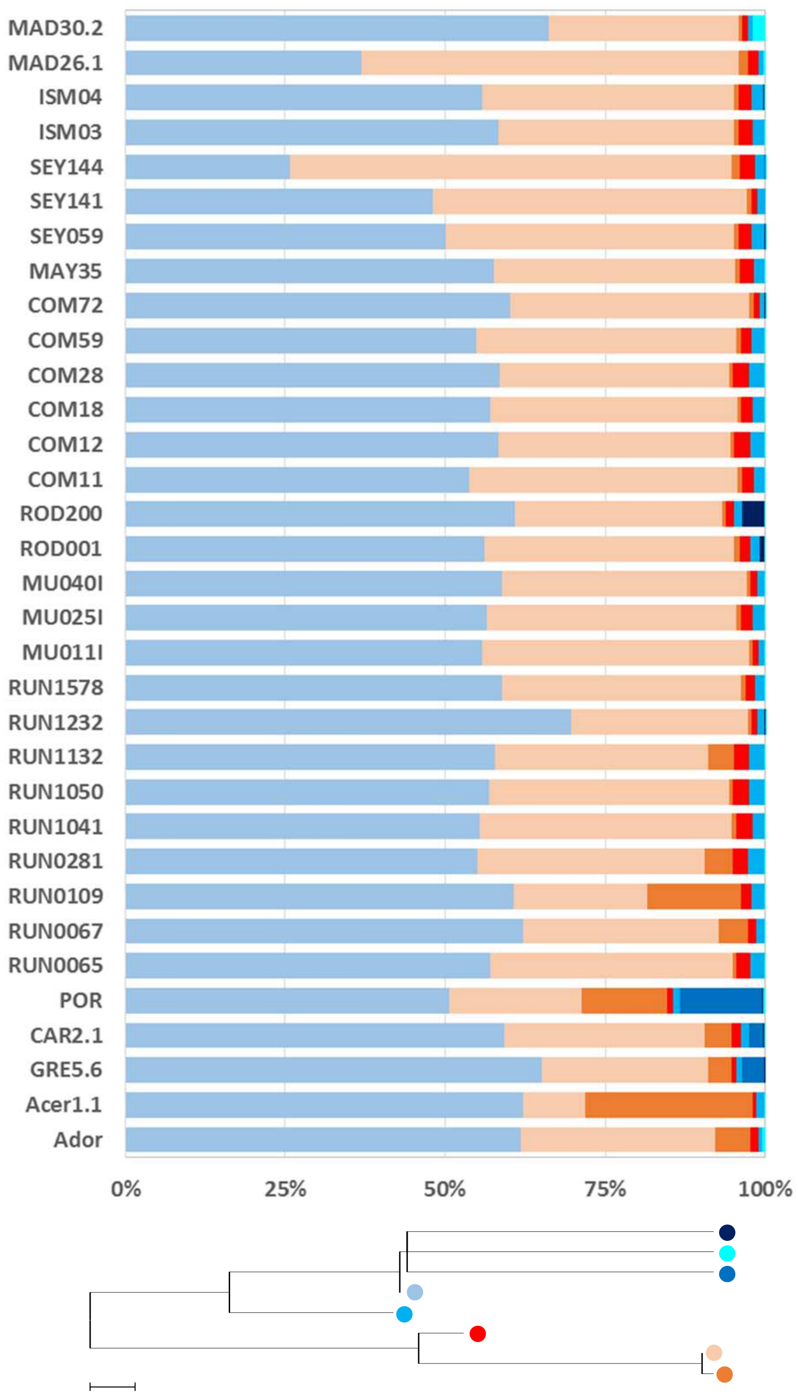

011BR (NC)

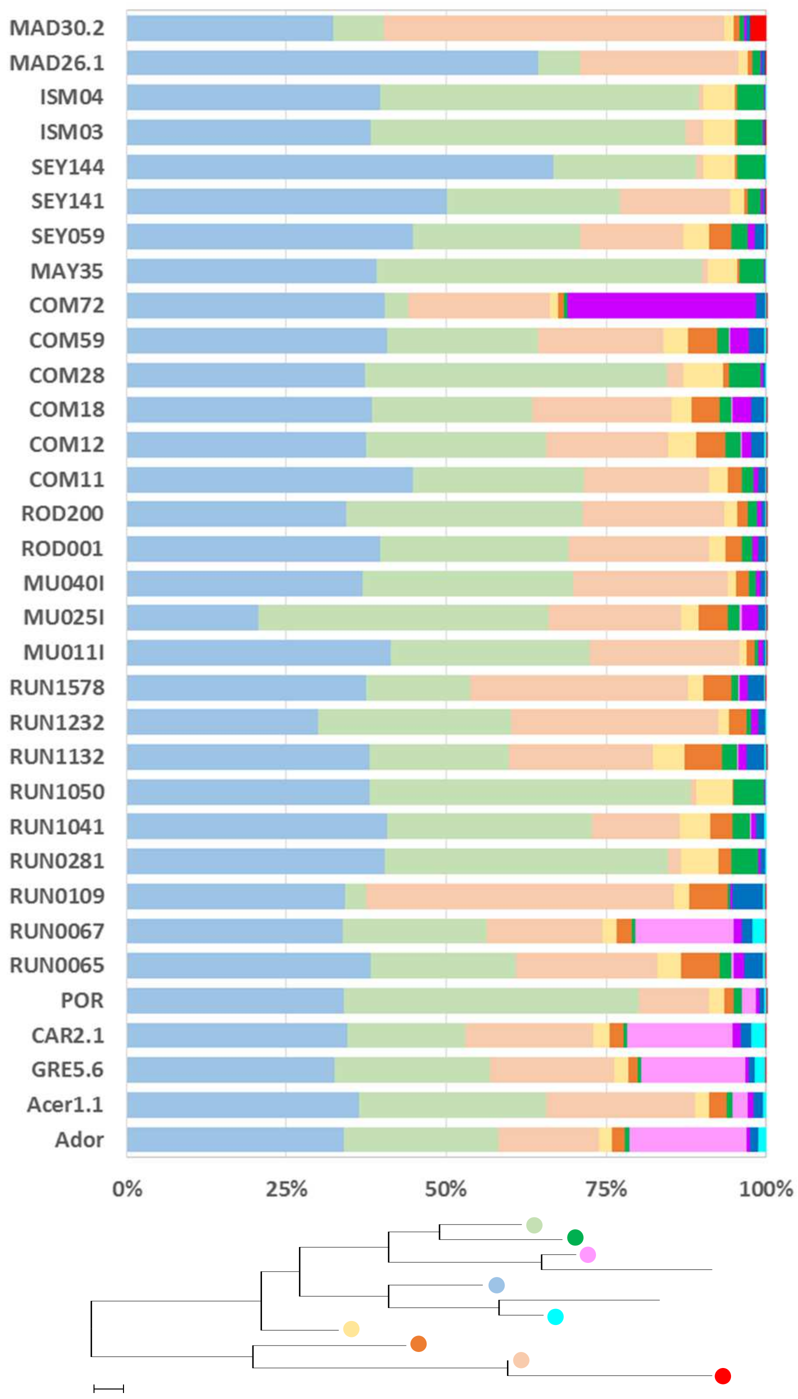

013AF (CR, in ORF AAJ76_1300036493)

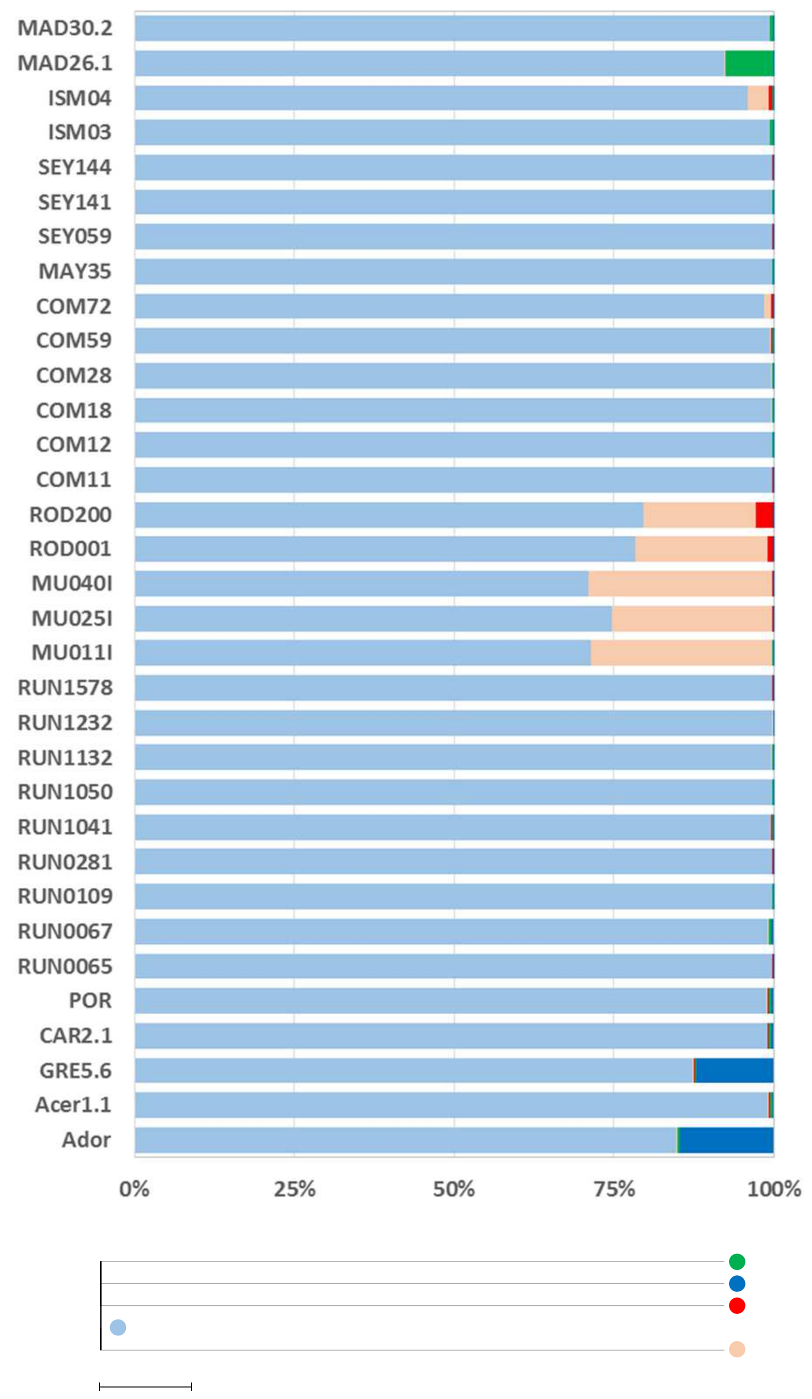

013AR (CR, in ORF AAJ76_1300036493)

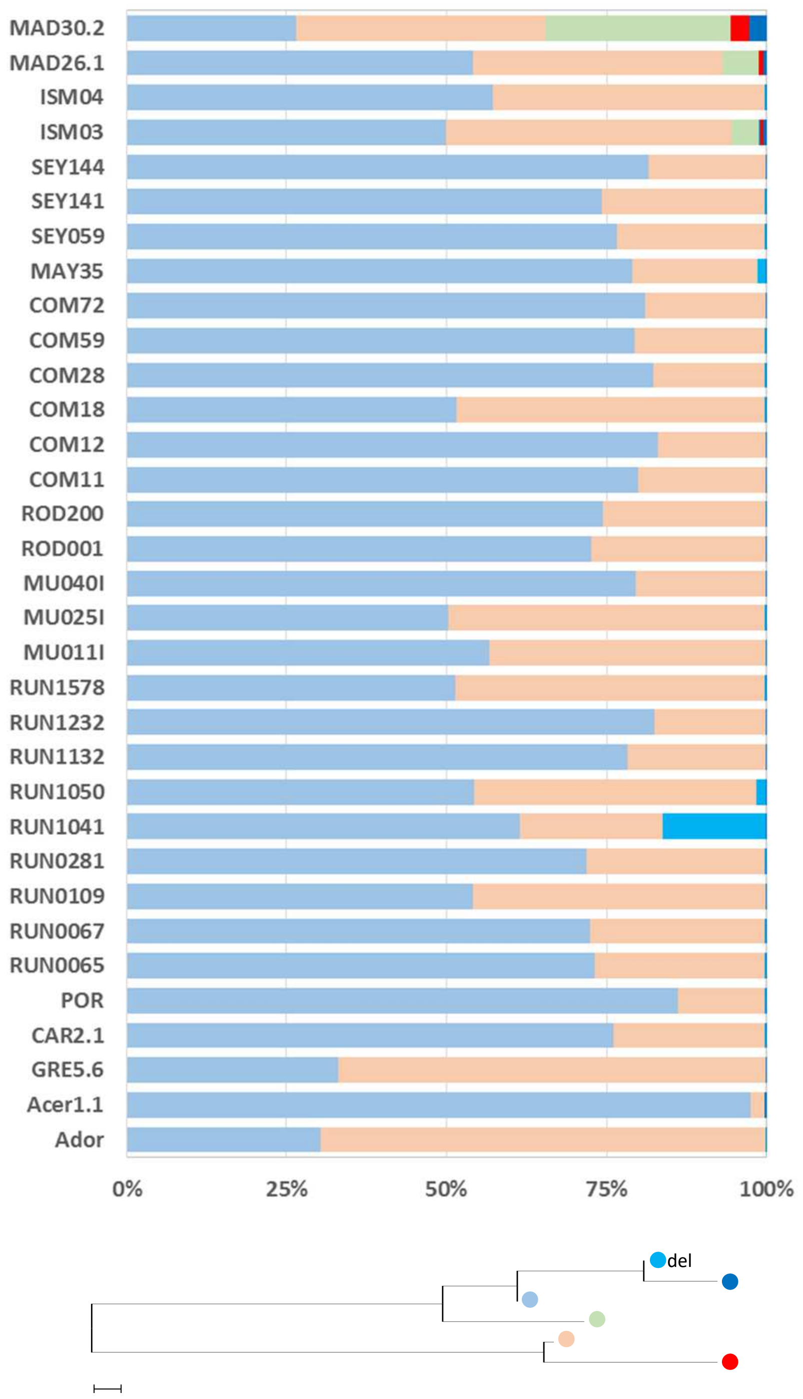

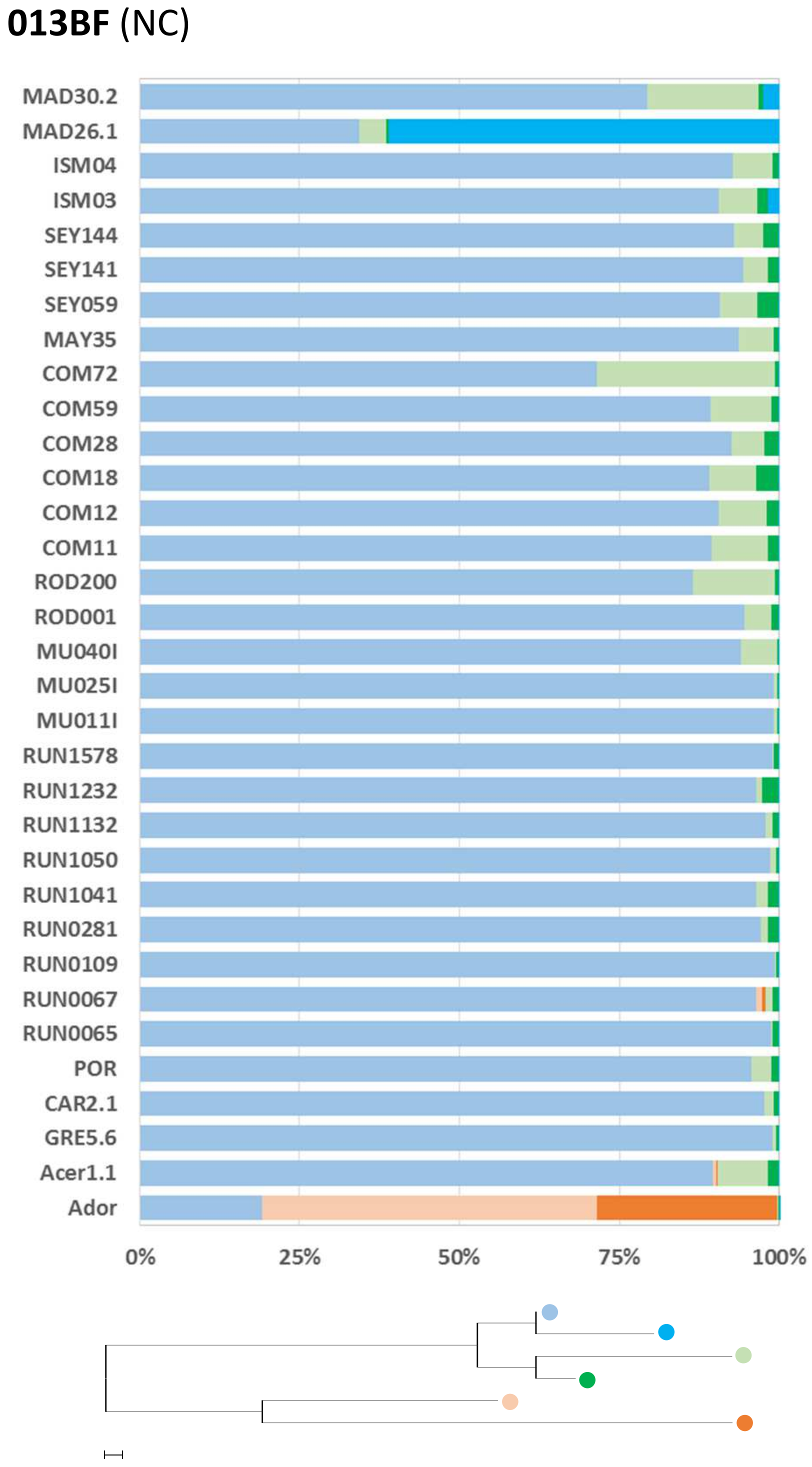
013BF (NC)
MAD30.2
MAD26.1
ISM04
ISM03
SEY144
SEY141
SEY059
MAY35
COM72
COM59
COM28
COM18
COM12
COM11
ROD200
ROD001
MU040I
MU025I
MU011I
RUN1578
RUN1232
RUN1132
RUN1050
RUN1041
RUN0281
RUN0109
RUN0067
RUN0065
POR
CAR2.1
GRE5.6
Acer1.1
Ador
0%
25%
50%
75%
100%

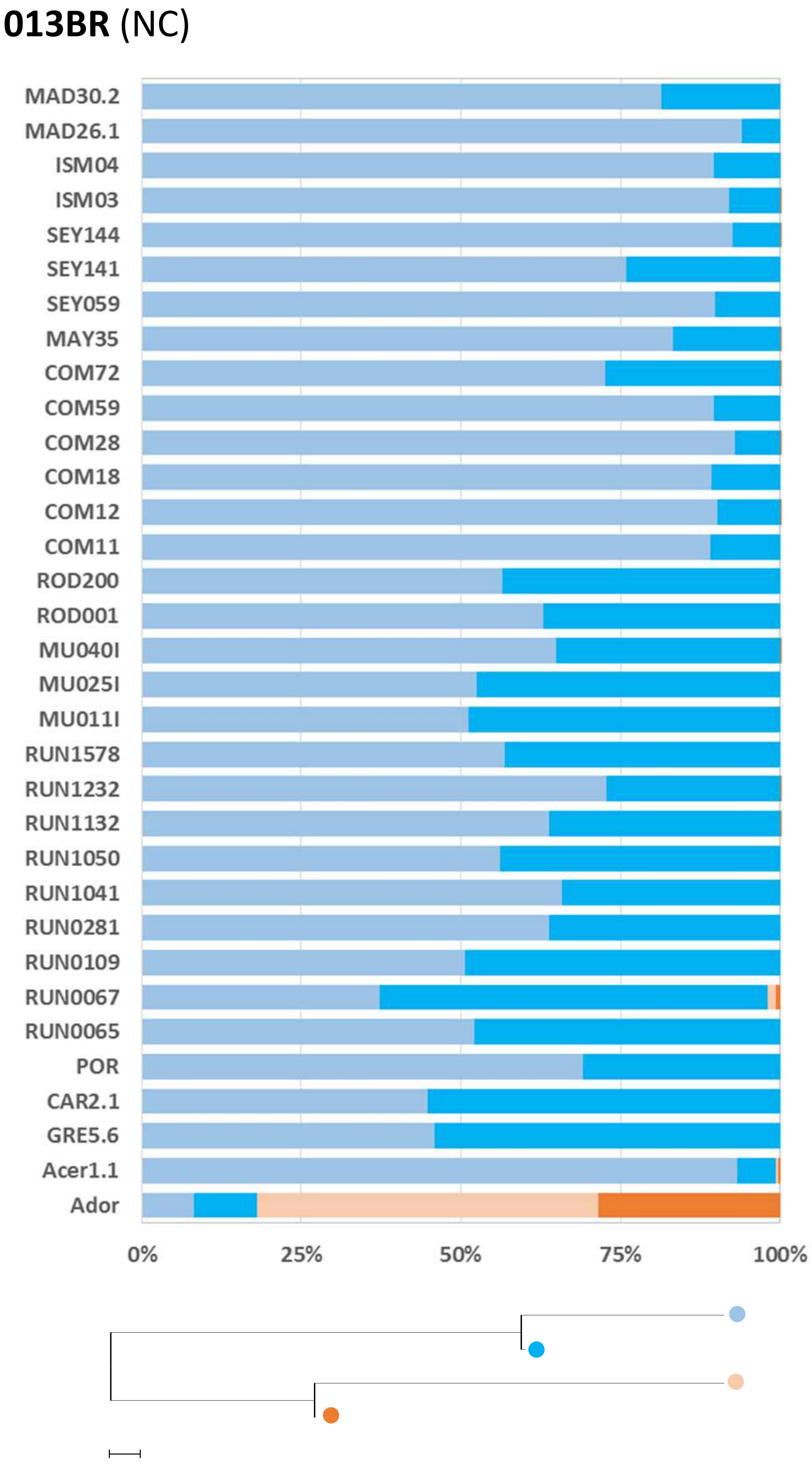
013BR (NC)
MAD30.2
MAD26.1
ISM04
ISM03
SEY144
SEY141
SEY059
MAY35
COM72
COM59
COM28
COM18
COM12
COM11
ROD200
ROD001
MU040I
MU025I
MU011I
RUN1578
RUN1232
RUN1132
RUN1050
RUN1041
RUN0281
RUN0109
RUN0067
RUN0065
POR
CAR2.1
GRE5.6
Acer1.1
Ador
0%
25%
50%
75%
100%

014AF (CR, zinc finger protein)

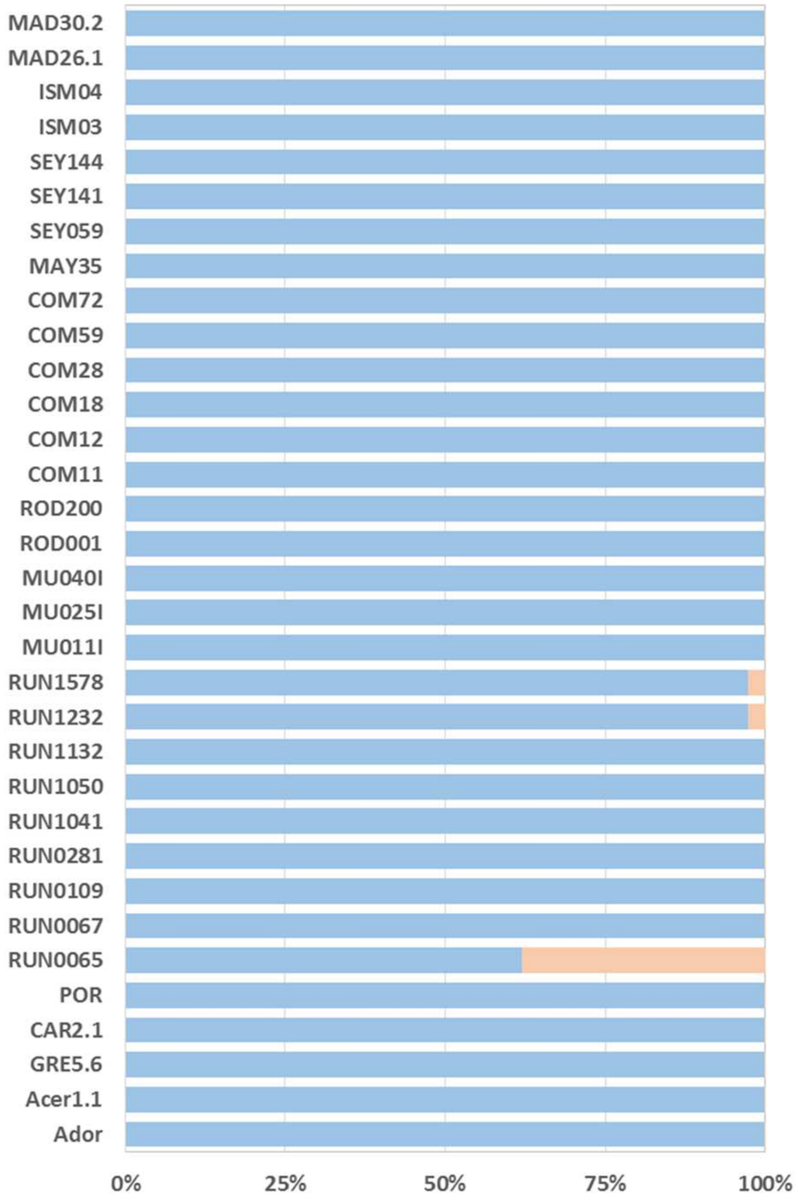

014AR (NC)

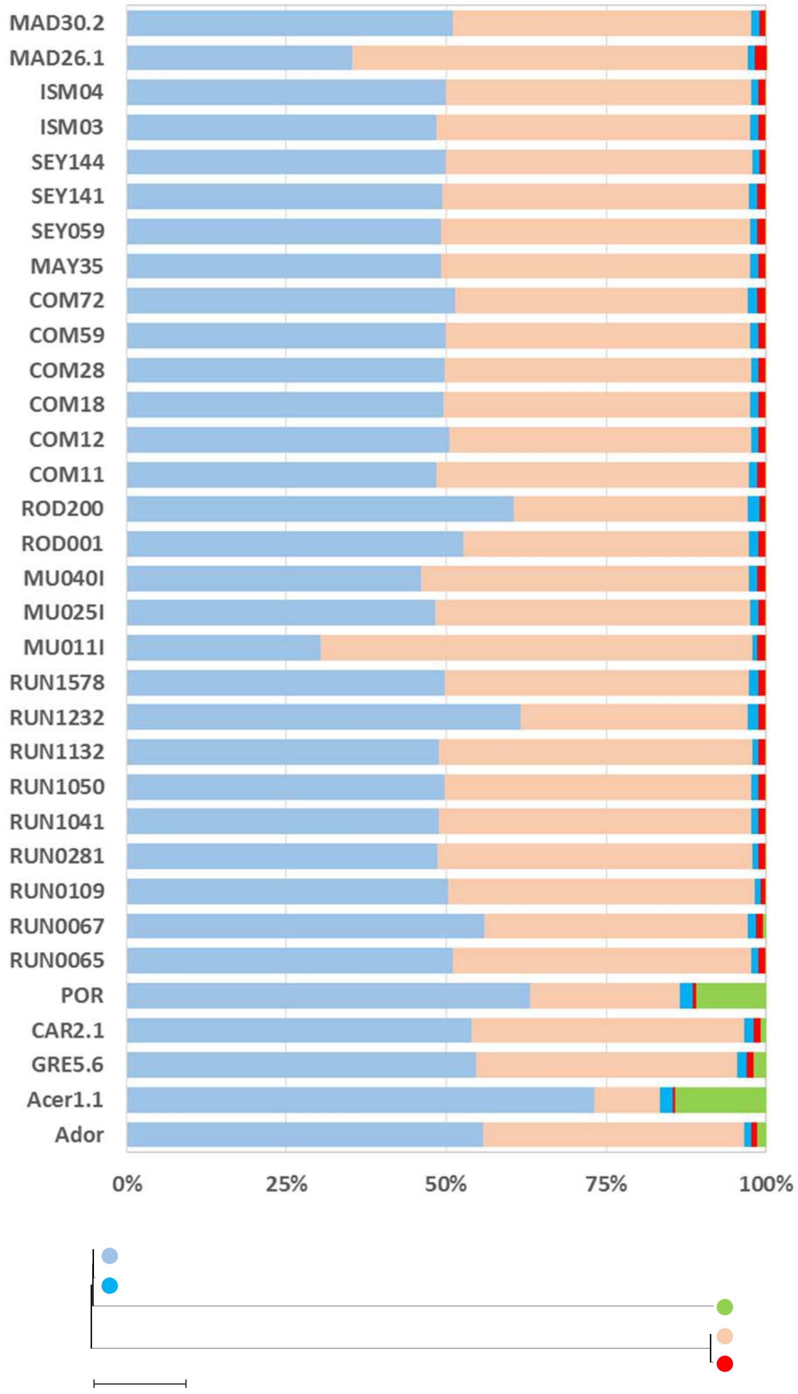

016AF (CR, in ORF AAJ76_1600025098)

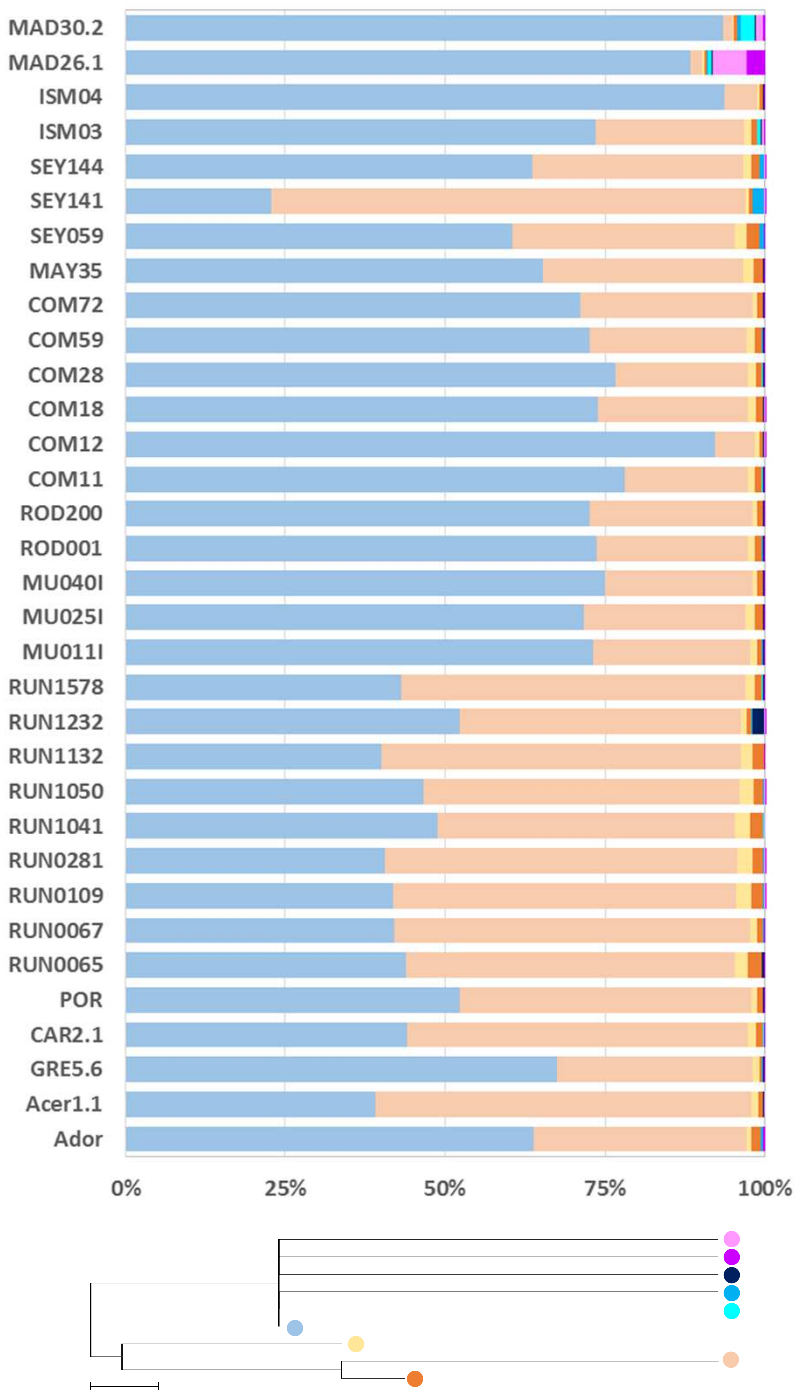

016AR (NC)

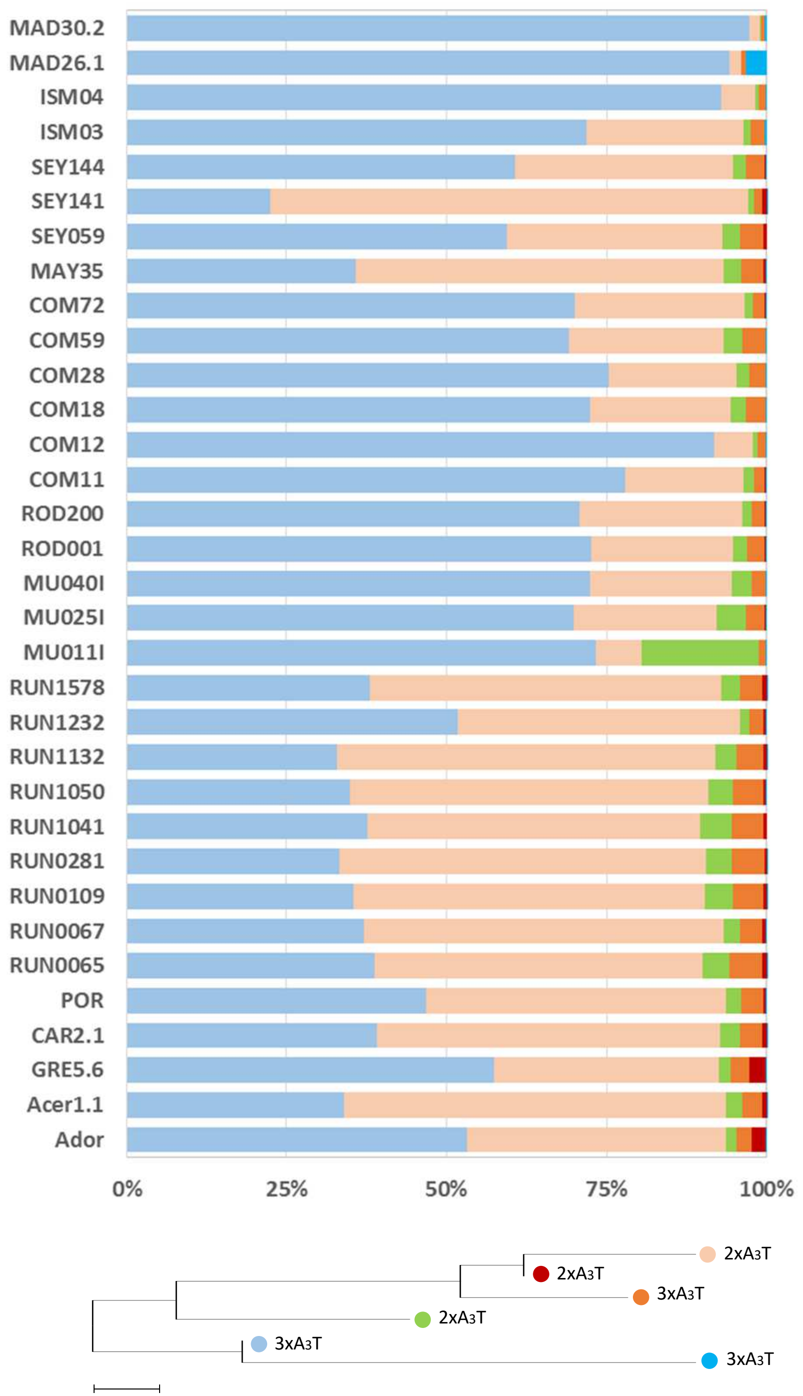

016BF (CR, AAA ATPase)

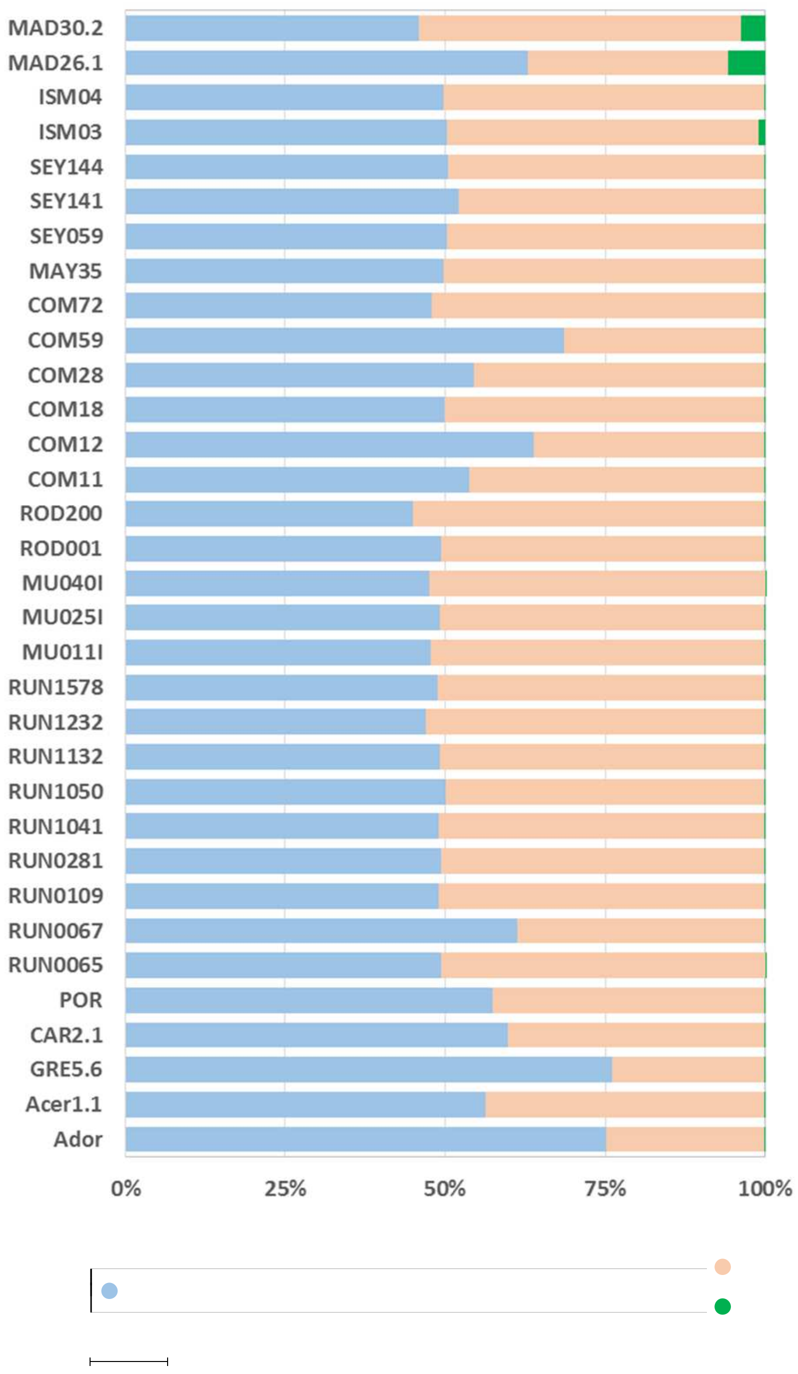

016BR (CR, AAA ATPase)

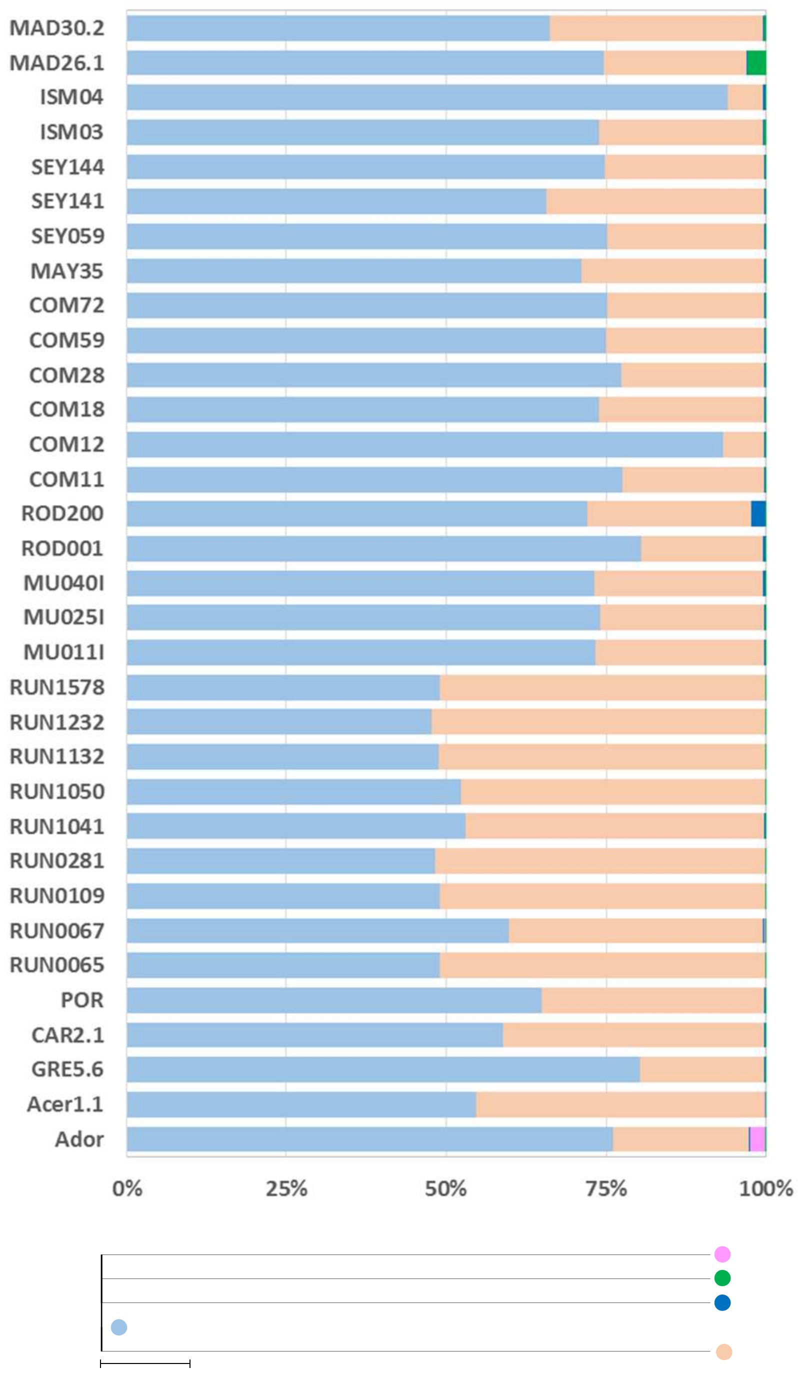

017AF (NC)

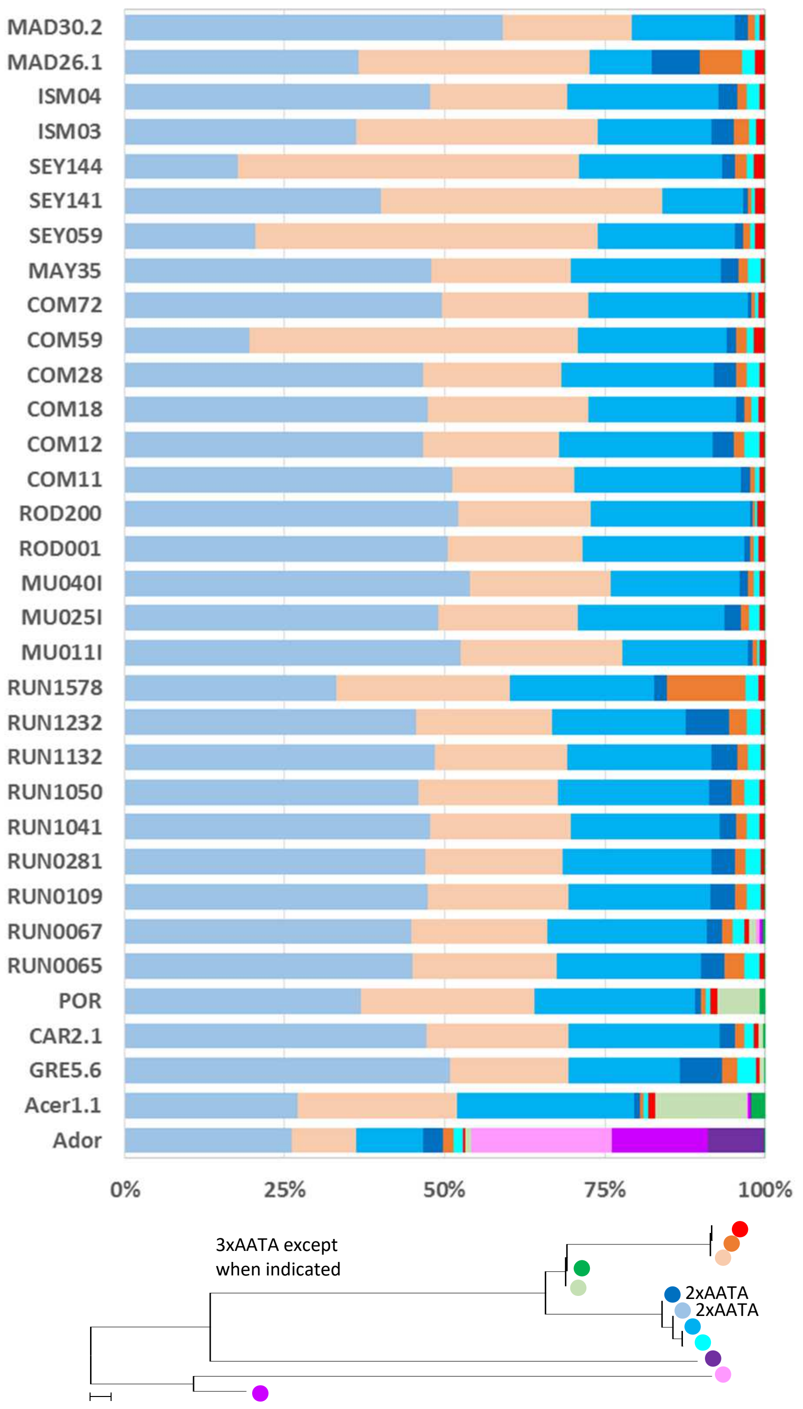

017AR (NC)

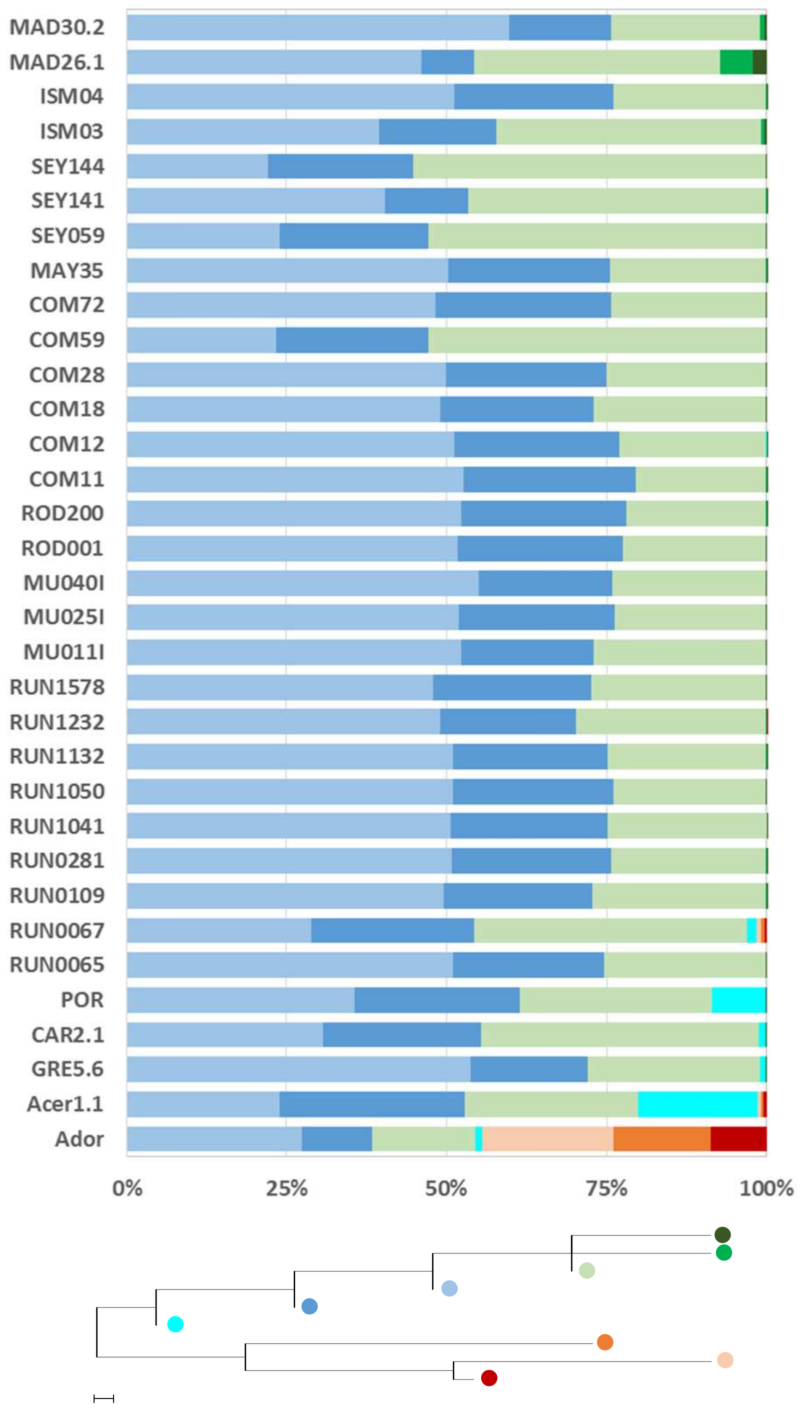

019AF (CR, transcription initiation factor subunit)

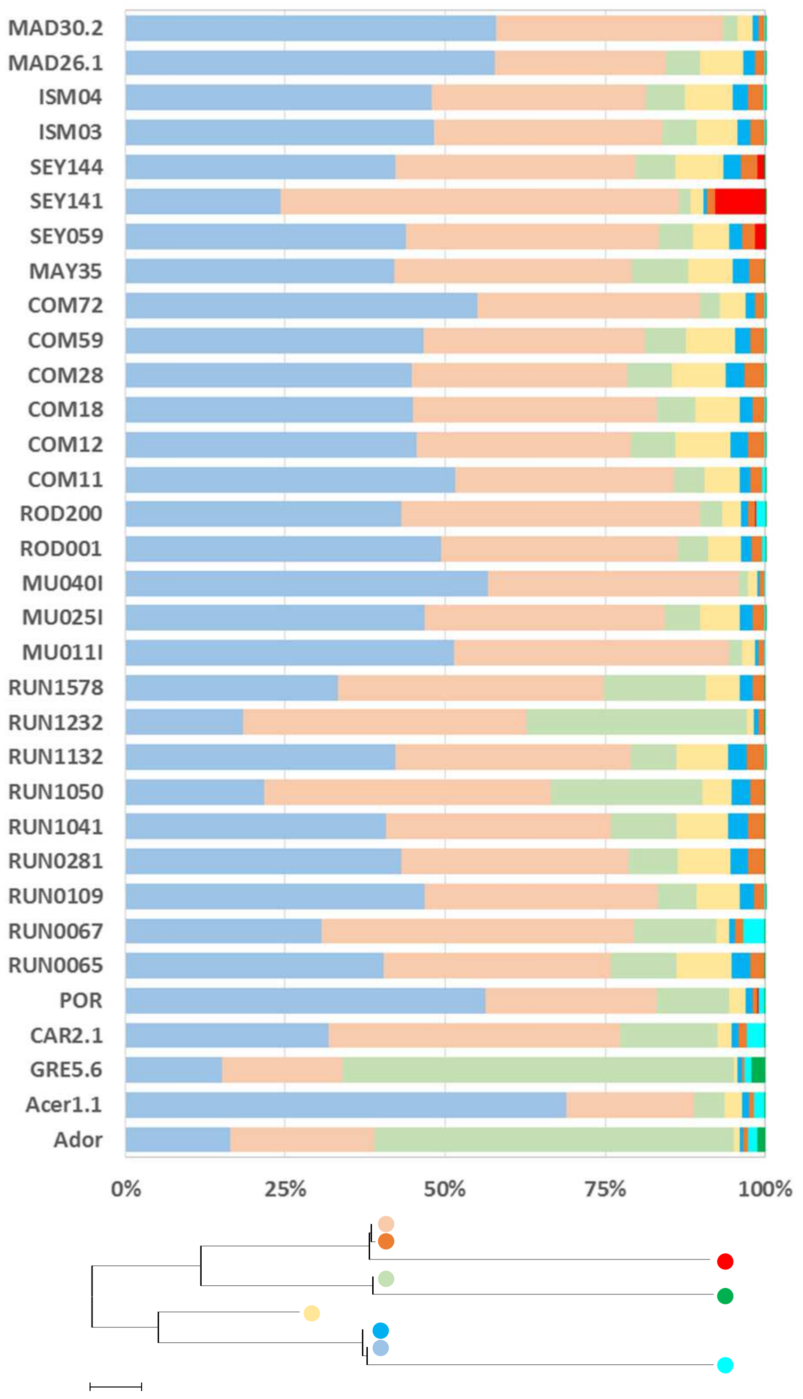

019AR (CR, transcription initiation factor subunit)

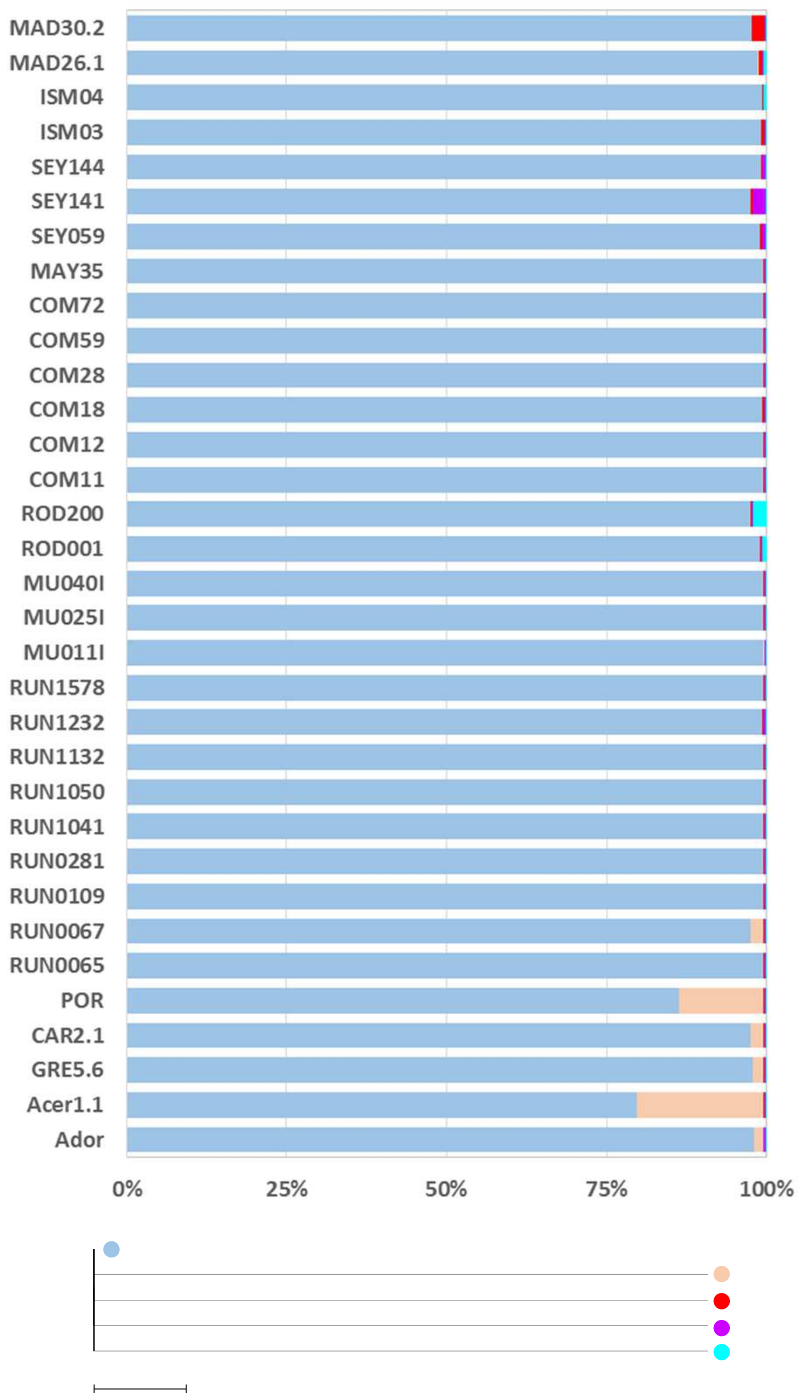

019BF (CR, polar tube protein PTP1)

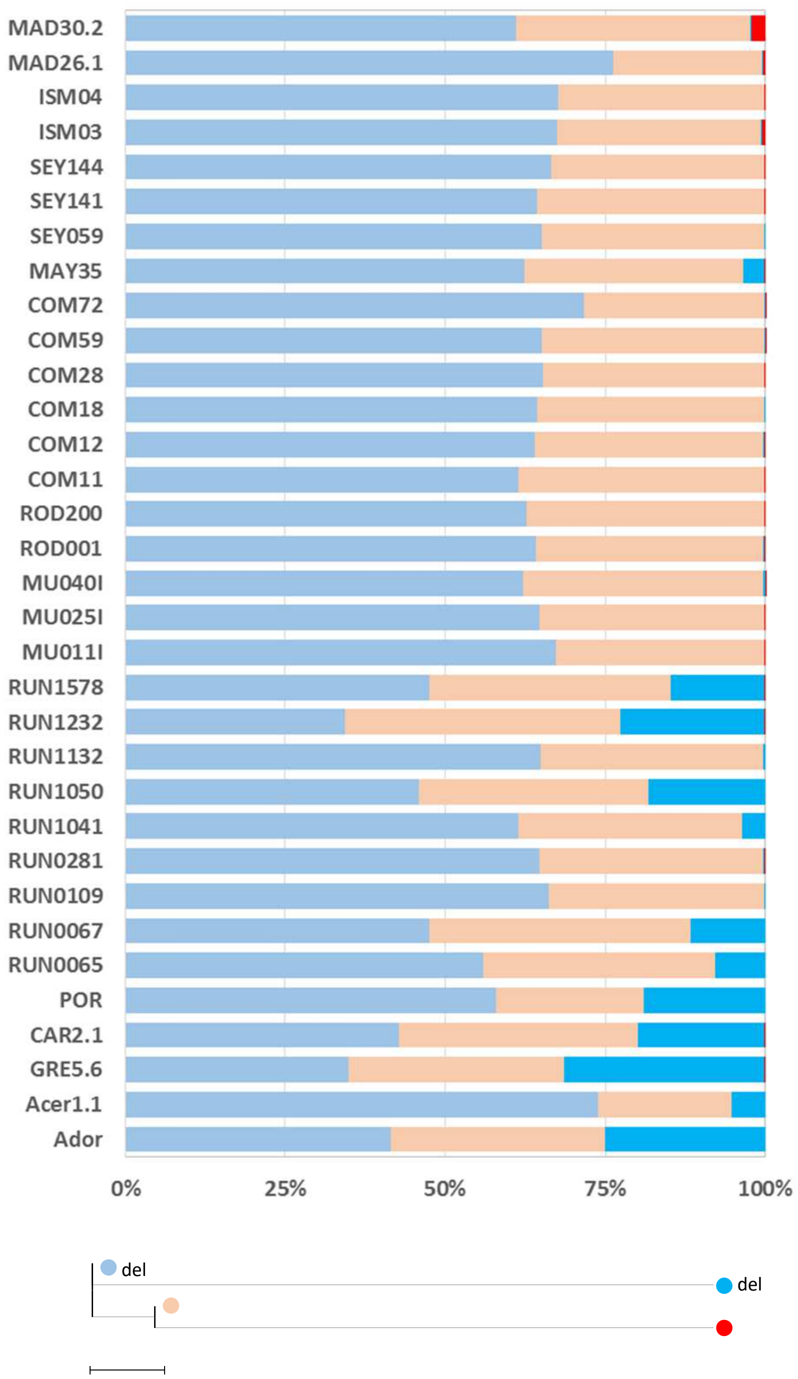

019BR (CR, polar tube protein PTP1)

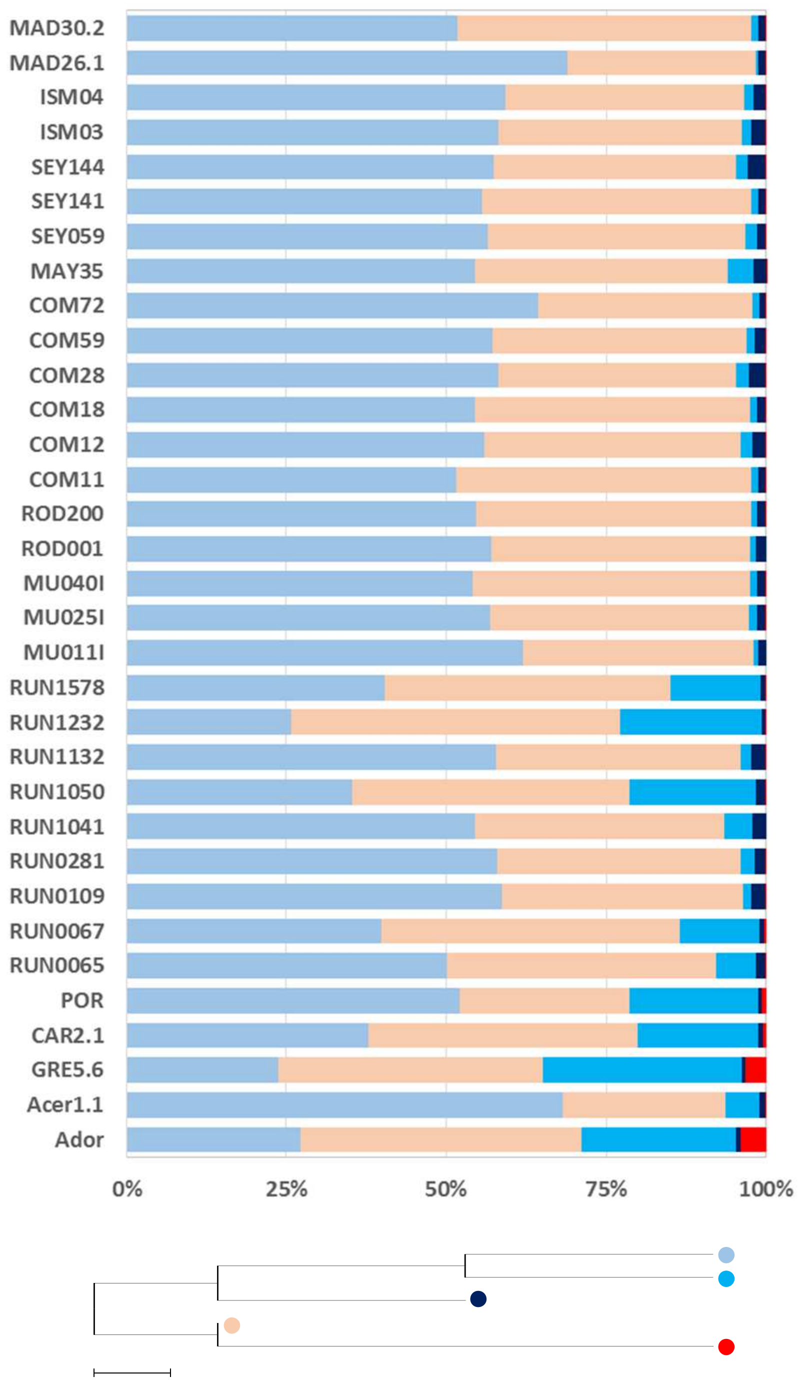

019CF (CR, polar tube protein PTP2)

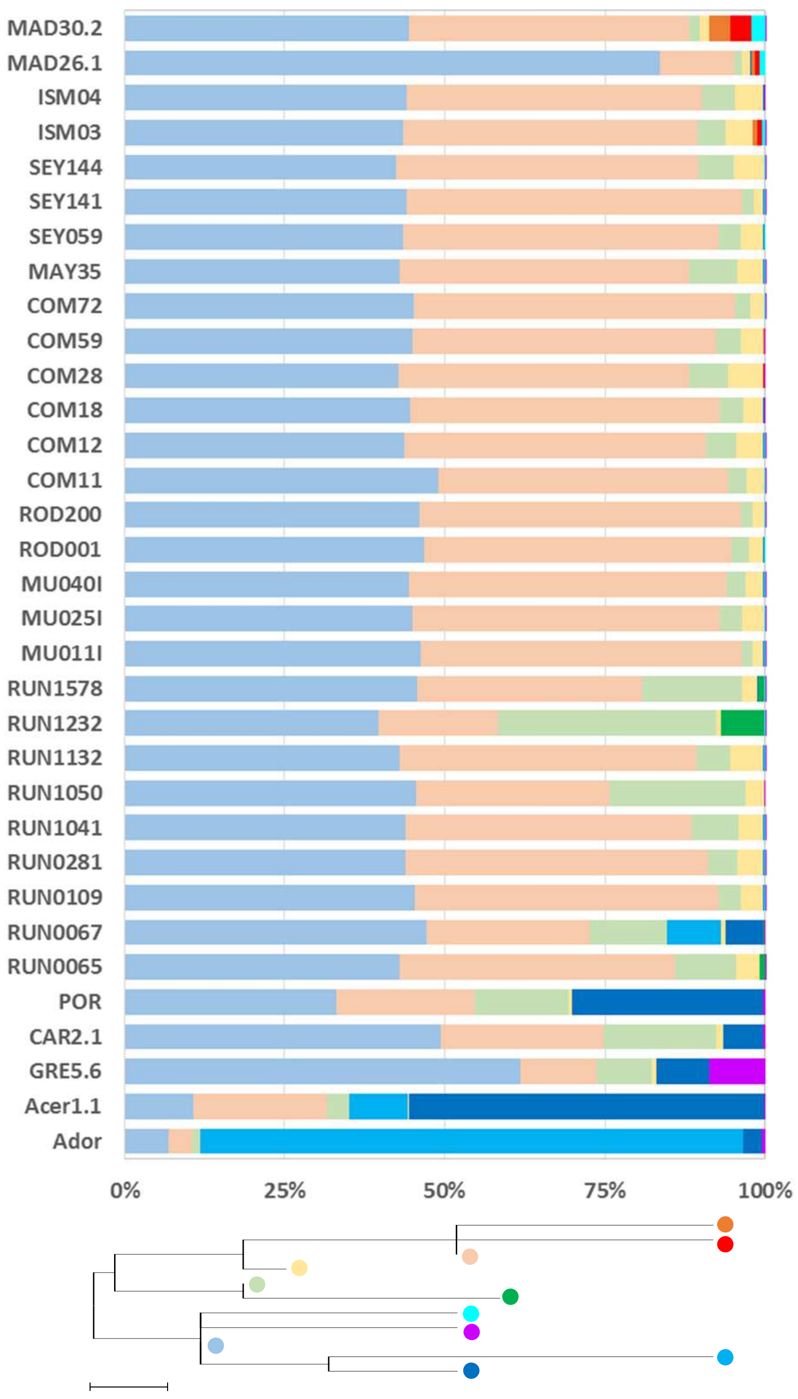

019CR (CR, polar tube protein PTP2)

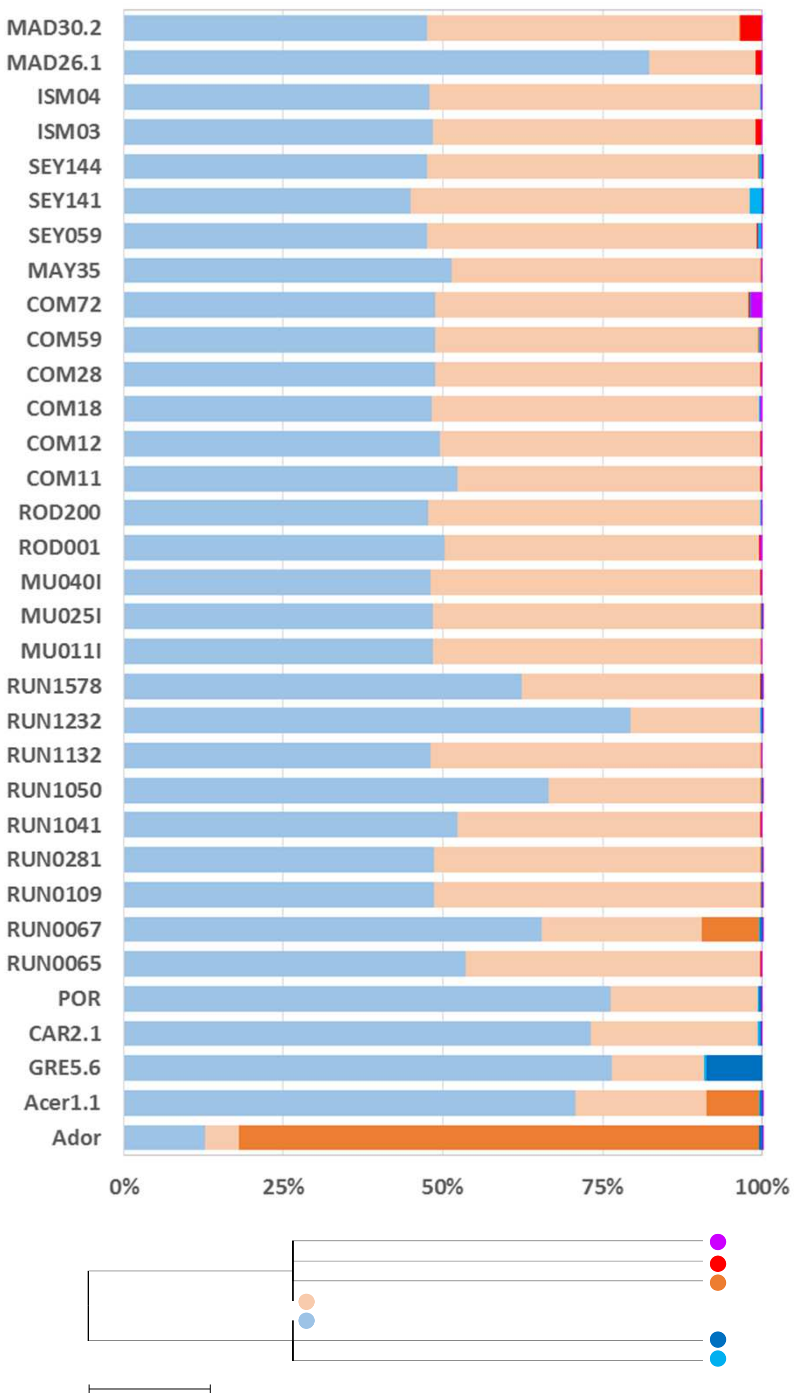

020AF (CR, in ORF AAJ76_2000058386)

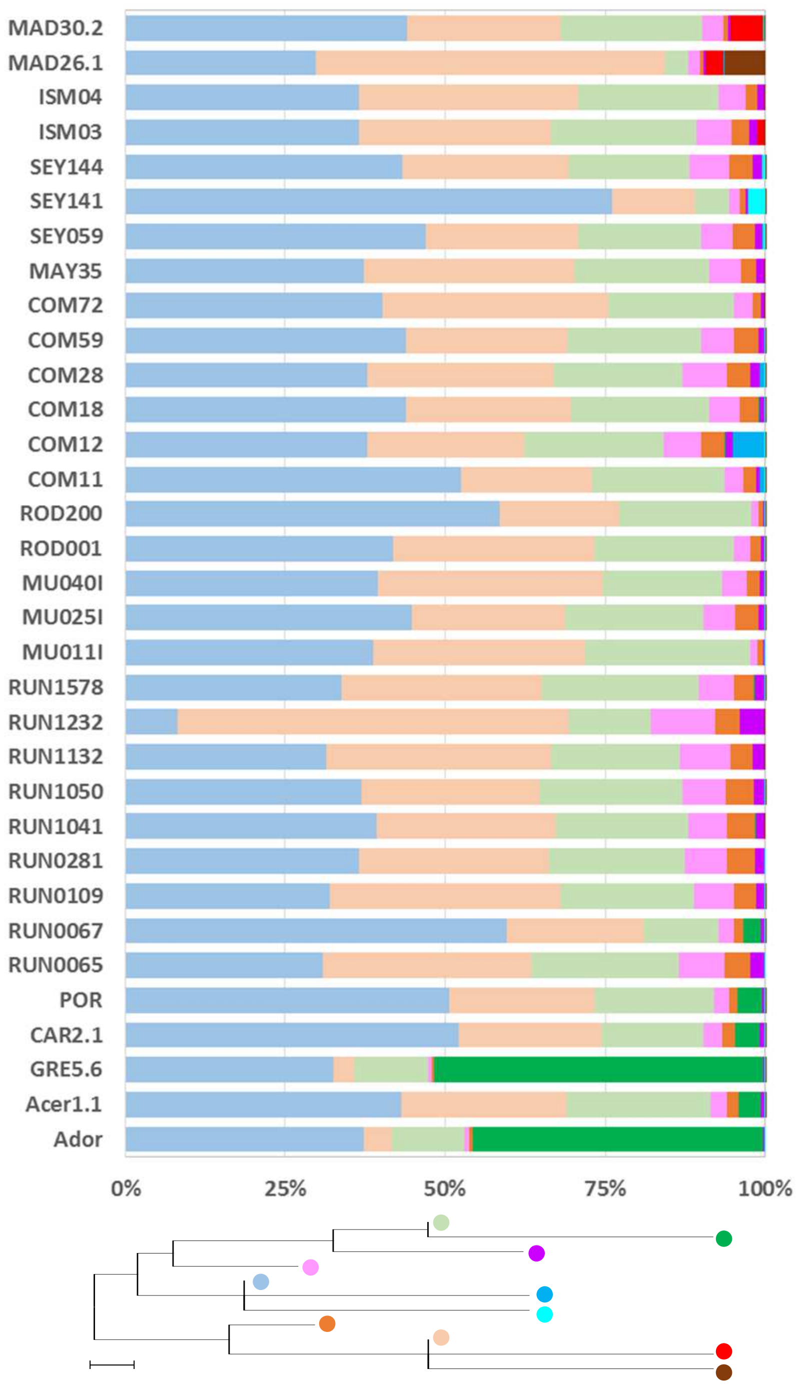

020AR (NC)

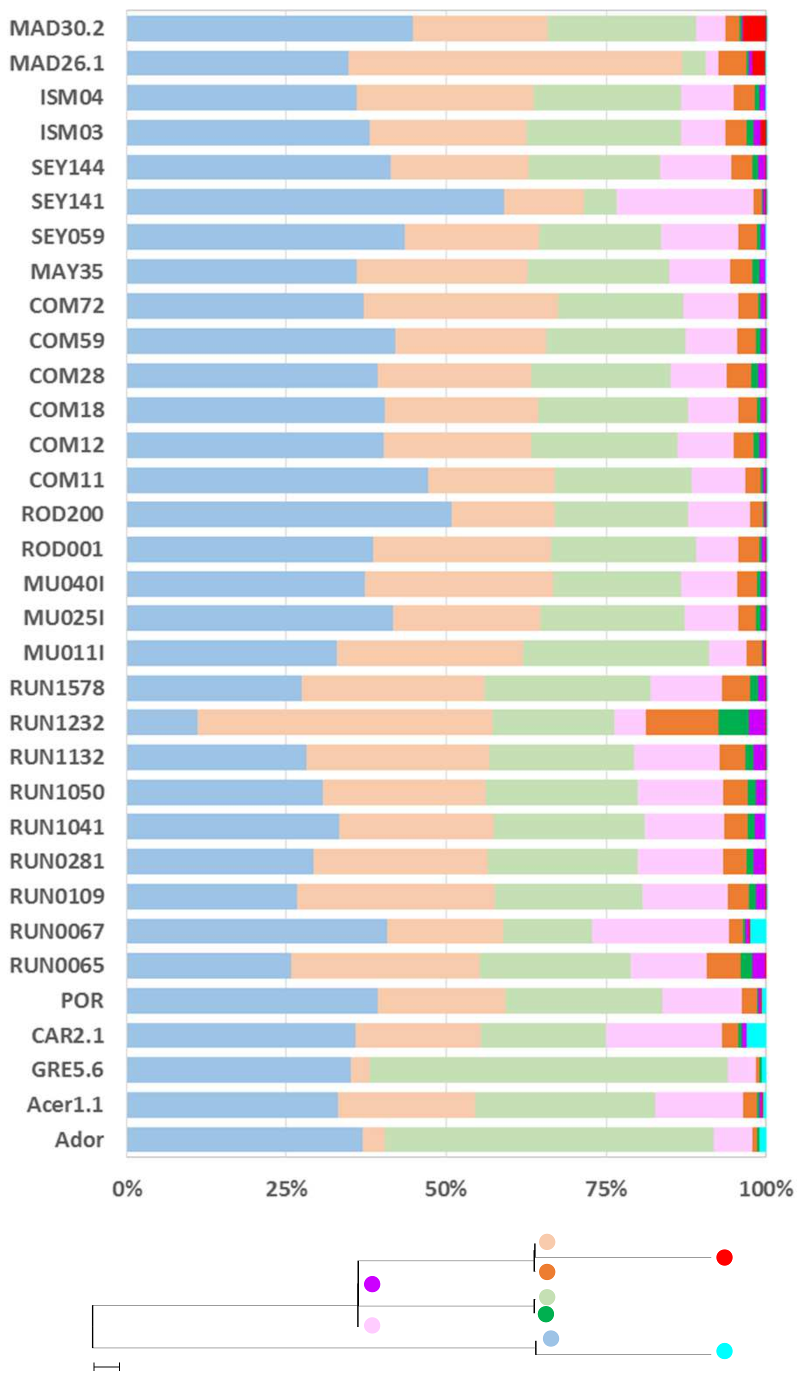

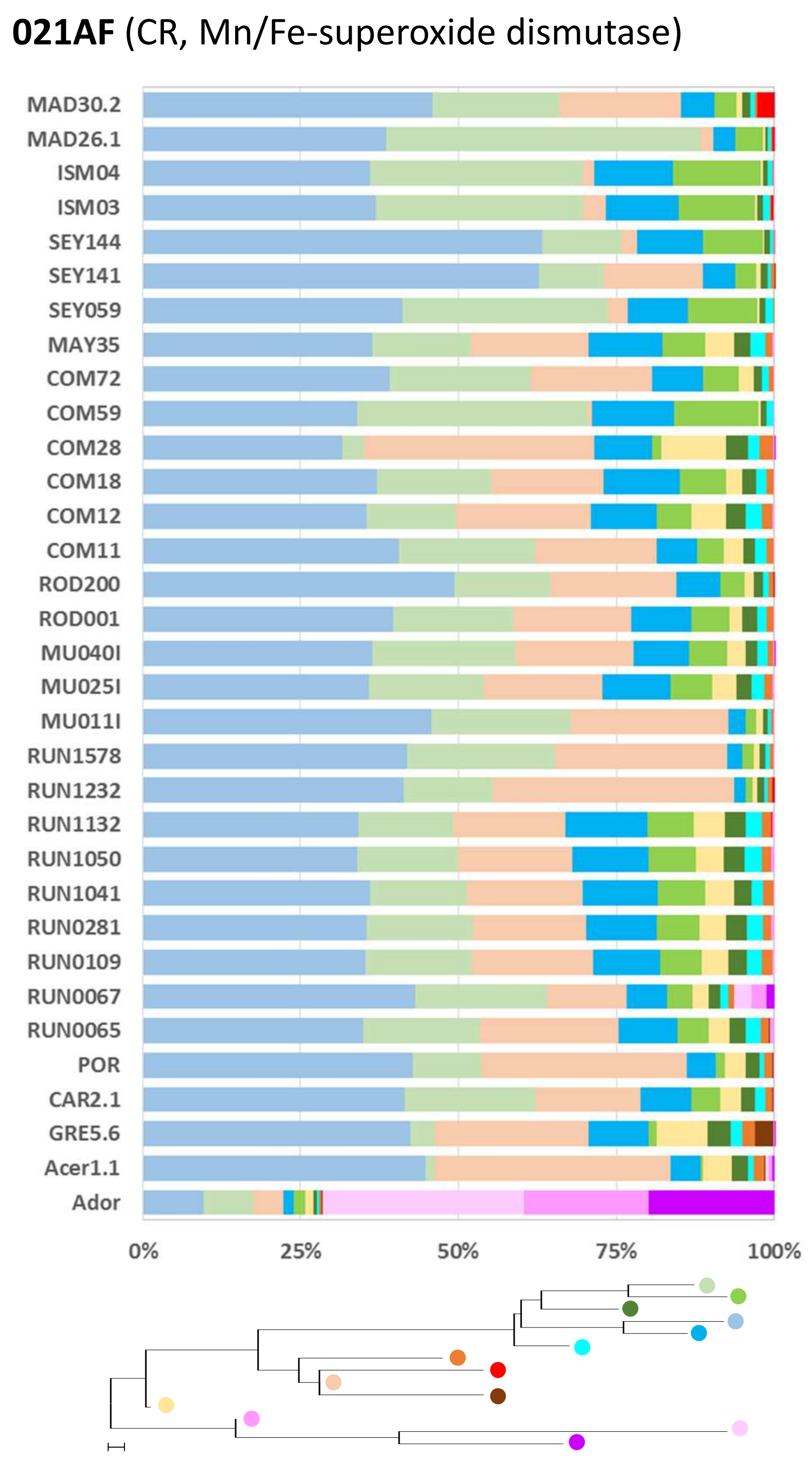
021AF (CR, Mn/Fe-superoxide dismutase)
MAD30.2
MAD26.1
ISM04
ISM03
SEY144
SEY141
SEY059
MAY35
COM72
COM59
COM28
COM18
COM12
COM11
ROD200
ROD001
MU040I
MU025I
MU011I
RUN1578
RUN1232
RUN1132
RUN1050
RUN1041
RUN0281
RUN0109
RUN0067
RUN0065
POR
CAR2.1
GRE5.6
Acer1.1
Ador
0%
25%
50%
75%
100%

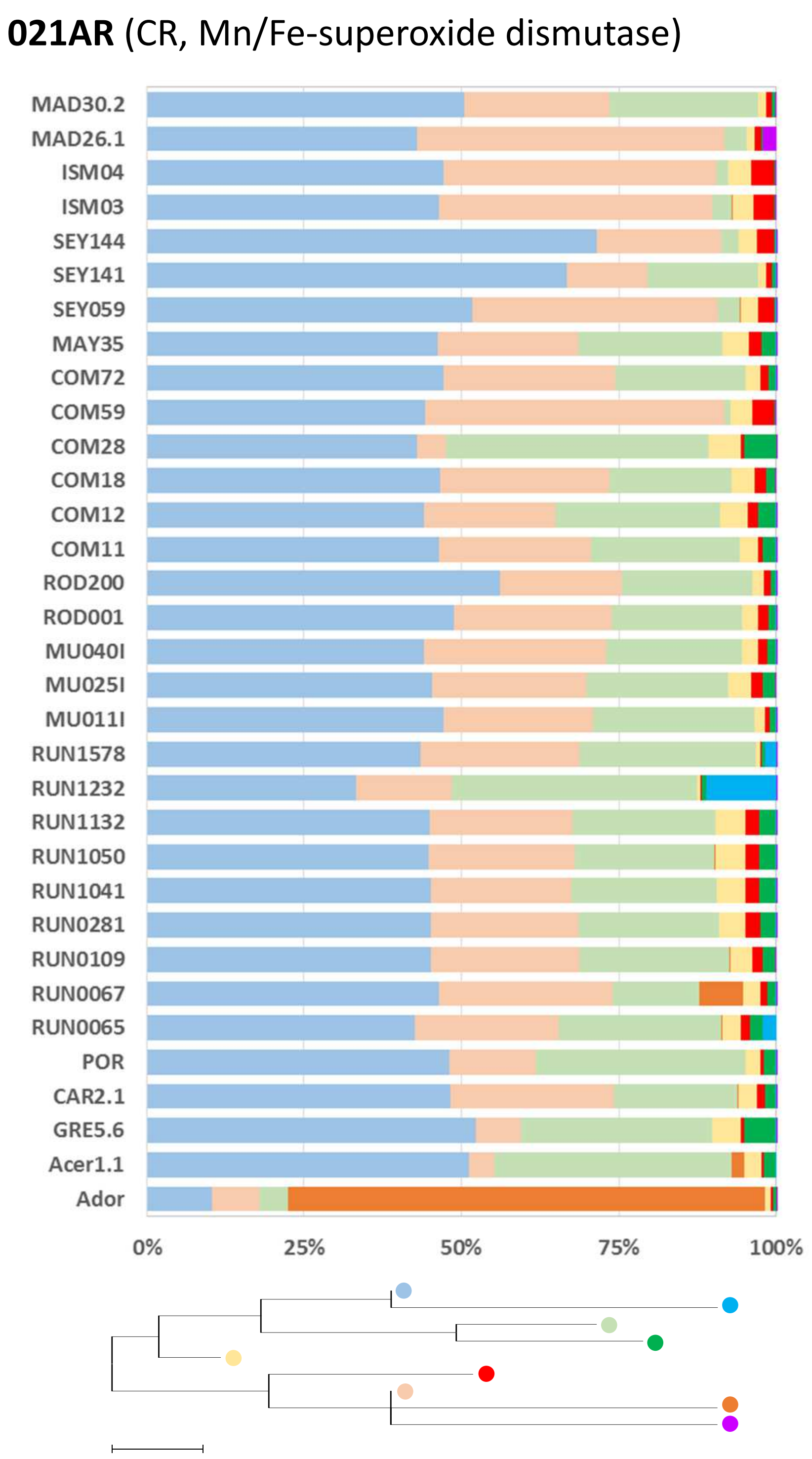
021AR (CR, Mn/Fe-superoxide dismutase)
MAD30.2
MAD26.1
ISM04
ISM03
SEY144
SEY141
SEY059
MAY35
COM72
COM59
COM28
COM18
COM12
COM11
ROD200
ROD001
MU040I
MU025I
MU011I
RUN1578
RUN1232
RUN1132
RUN1050
RUN1041
RUN0281
RUN0109
RUN0067
RUN0065
POR
CAR2.1
GRE5.6
Acer1.1
Ador
0%
25%
50%
75%
100%

021BF (CR, RNA polymerase II largest subunit RPB1)

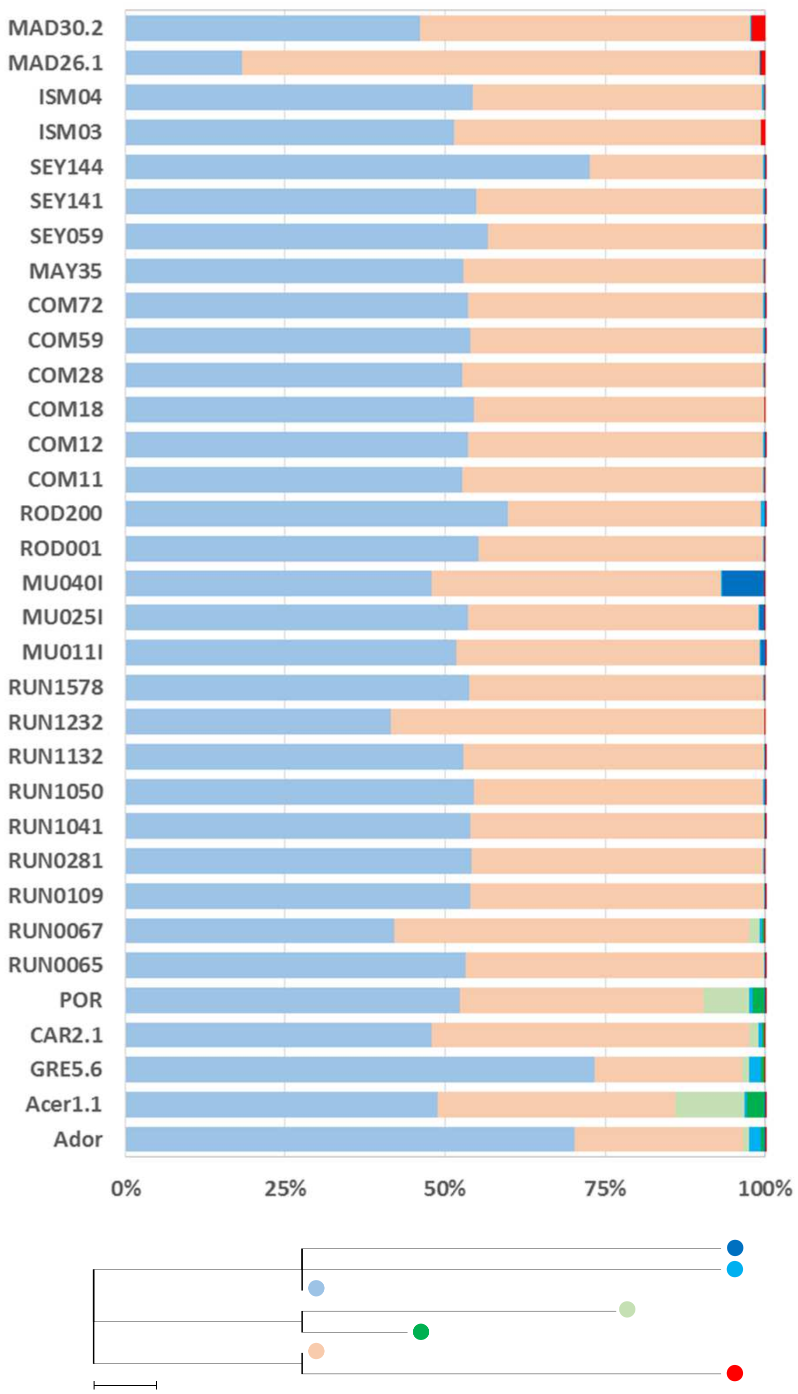

021BR (CR, RNA polymerase II largest subunit RPB1)

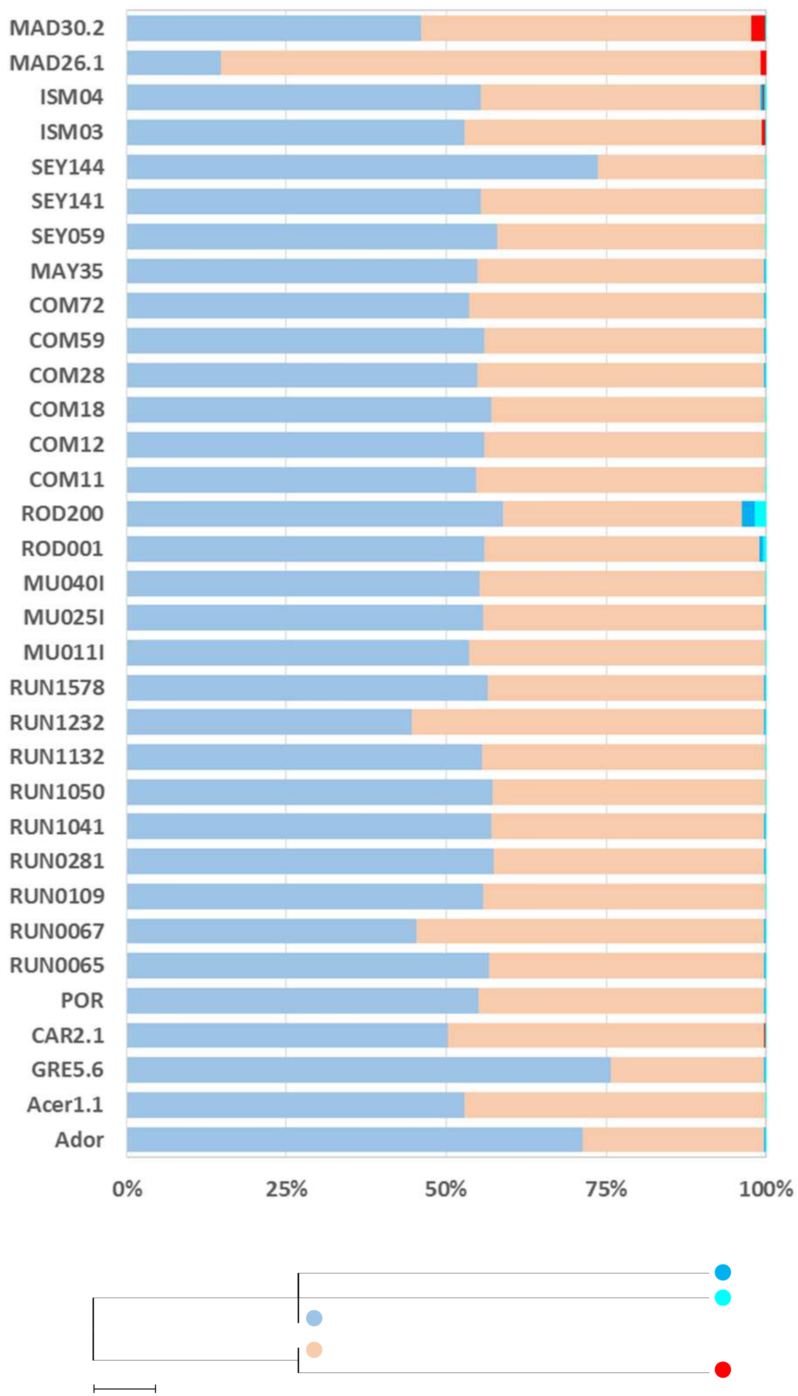

025AF (CR, Asn synthetase)

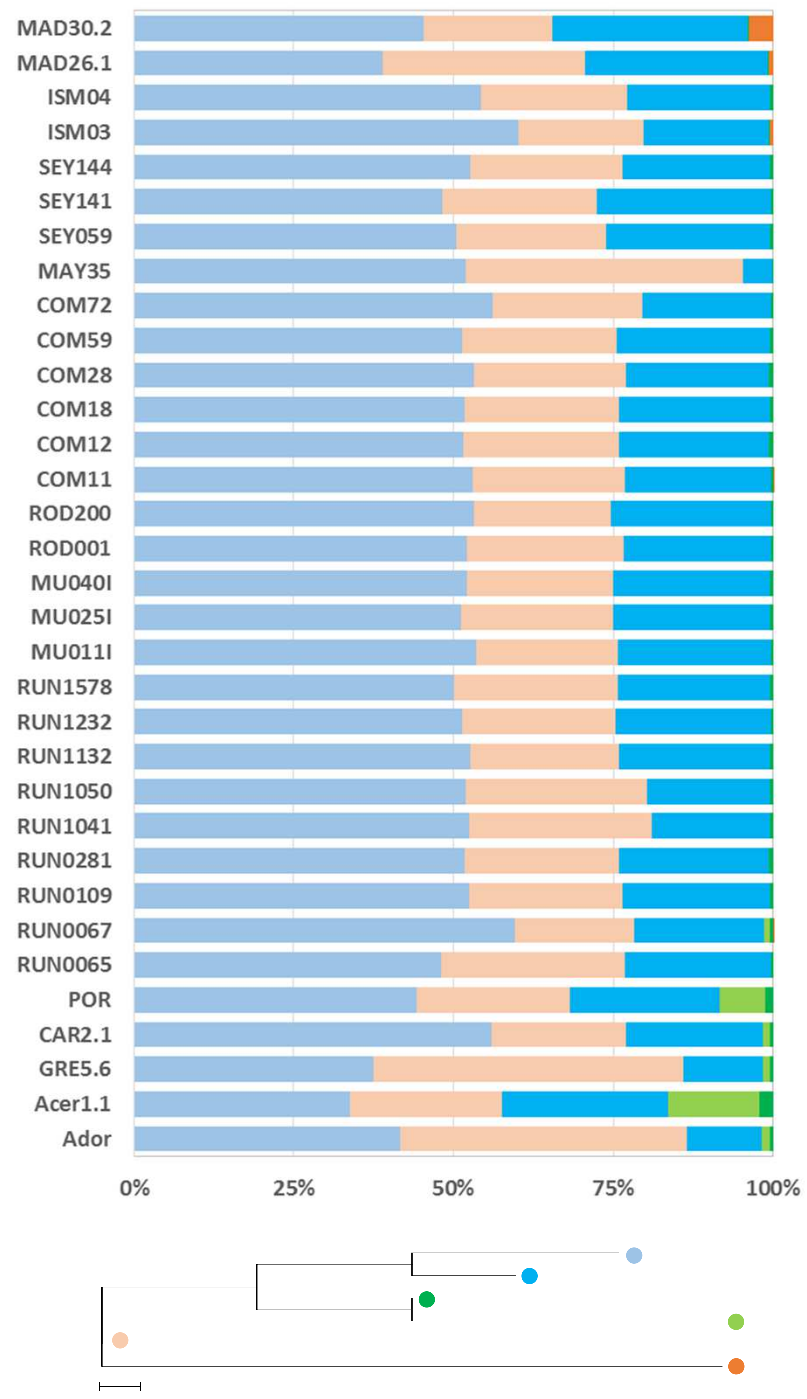

025AR (CR, Asn synthetase)

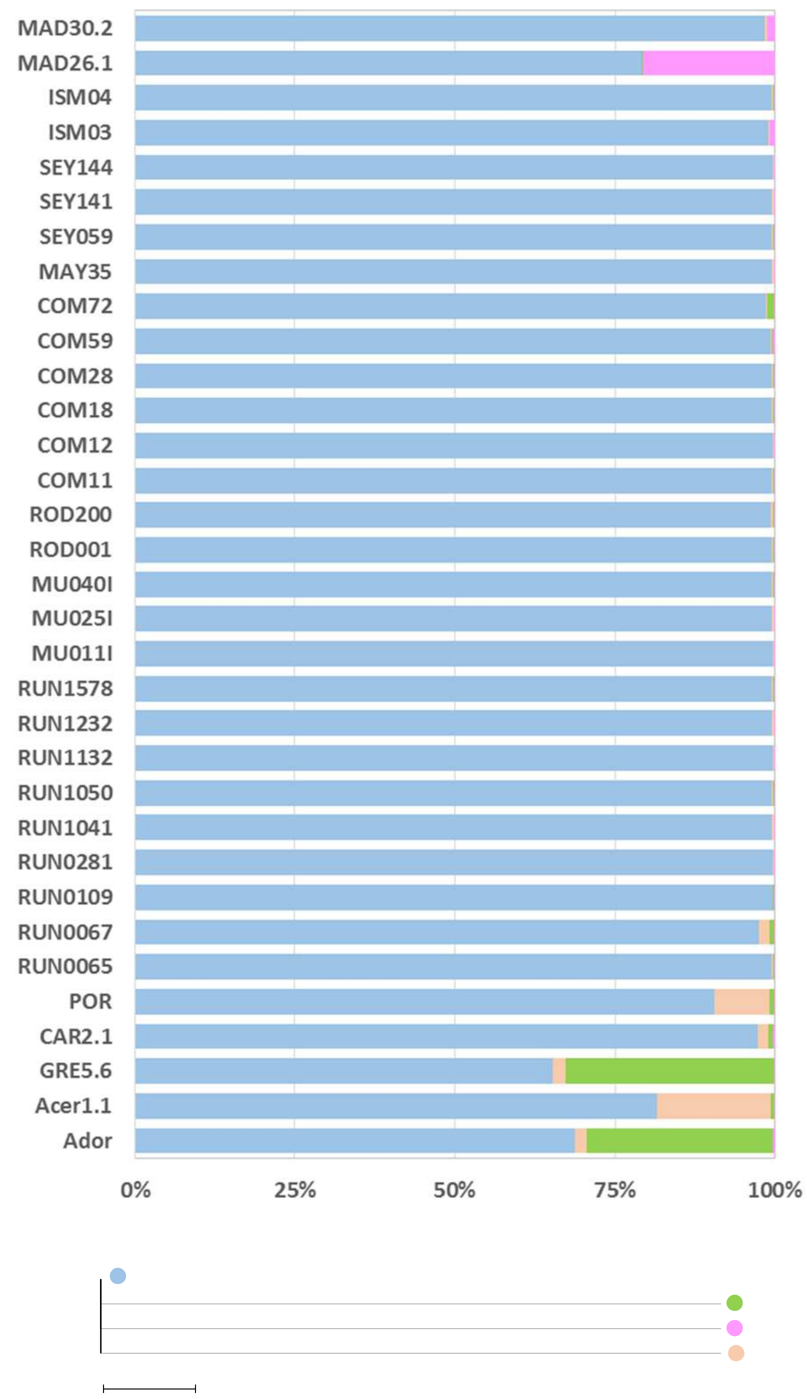

034AF (NC)

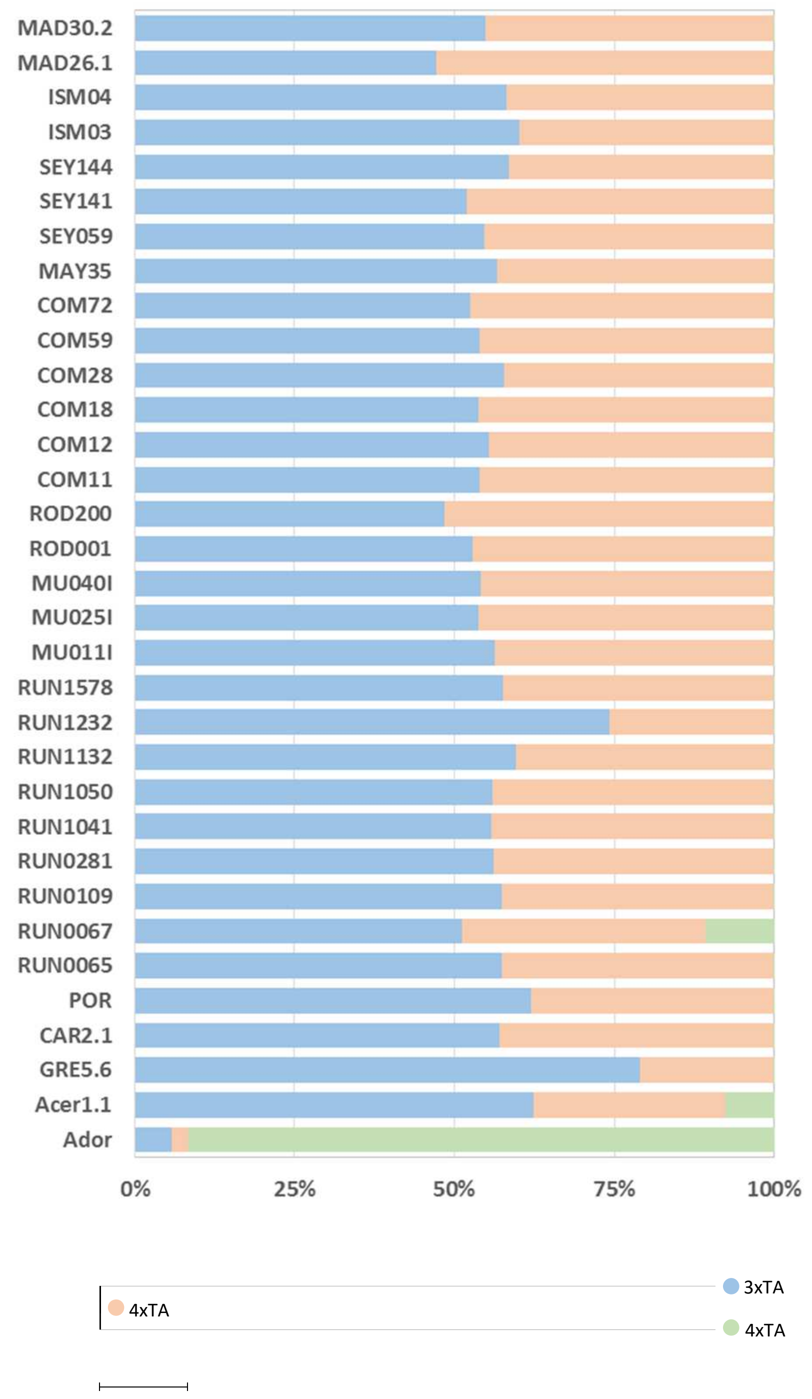

034AR (CR, in ORF AAJ76_3400023038)

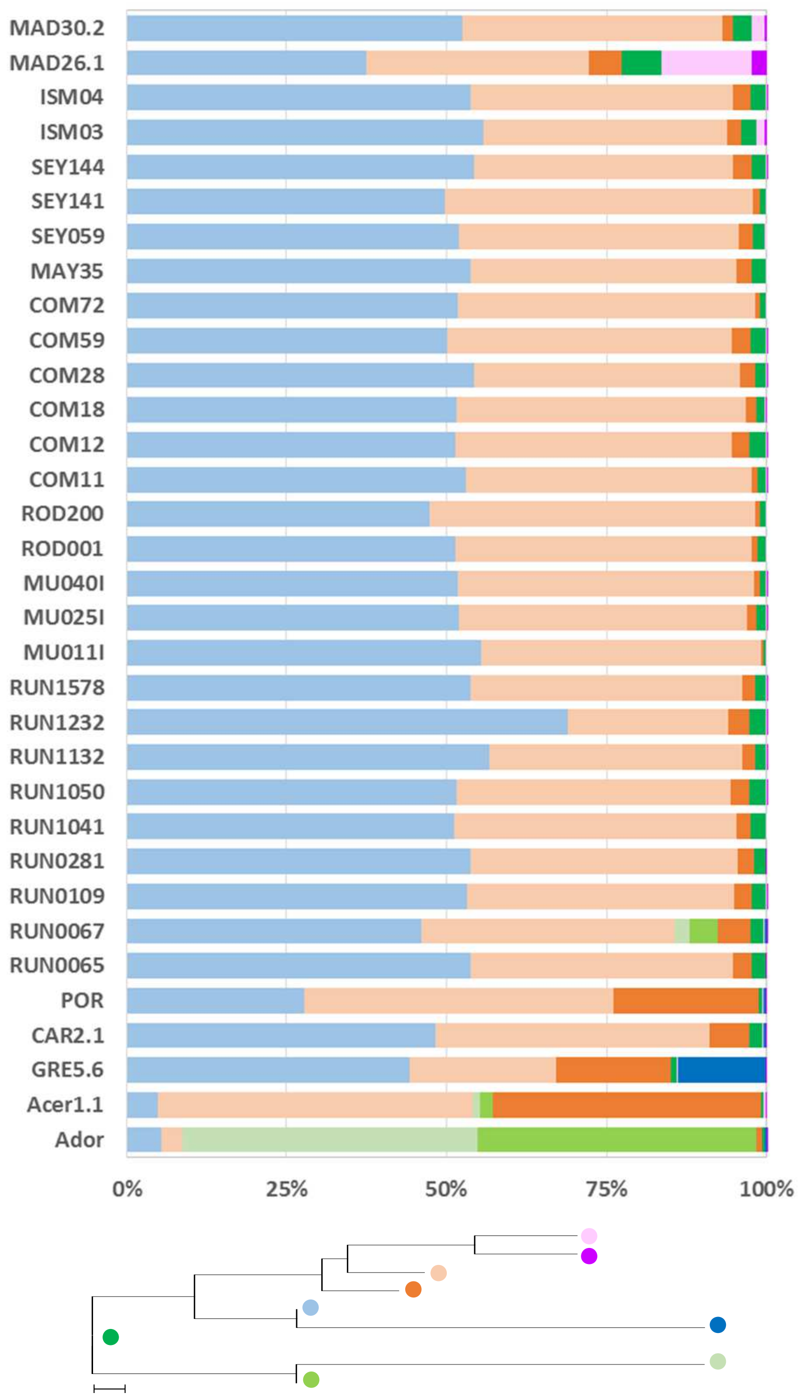

036AF (CR, alpha-tubulin)

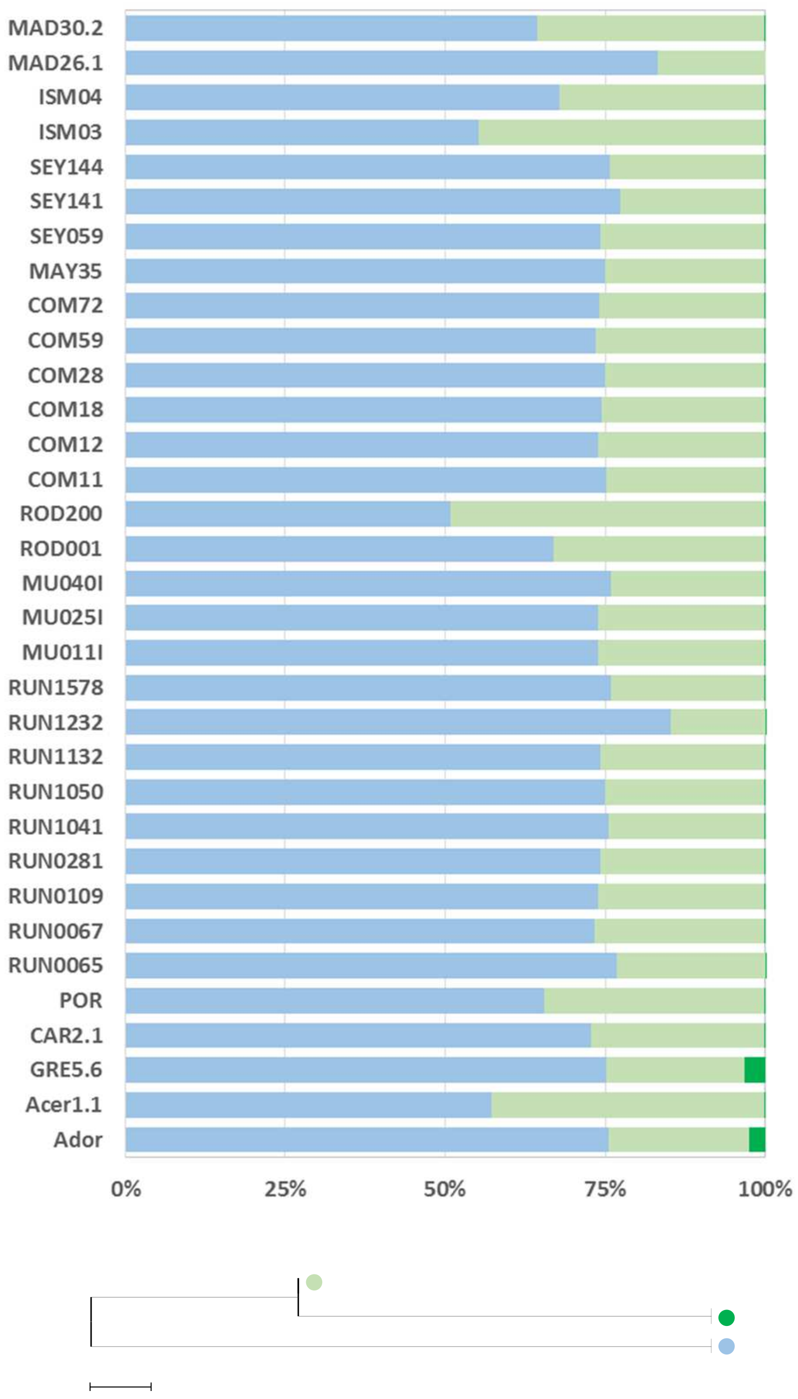

036AR (CR, alpha-tubulin)

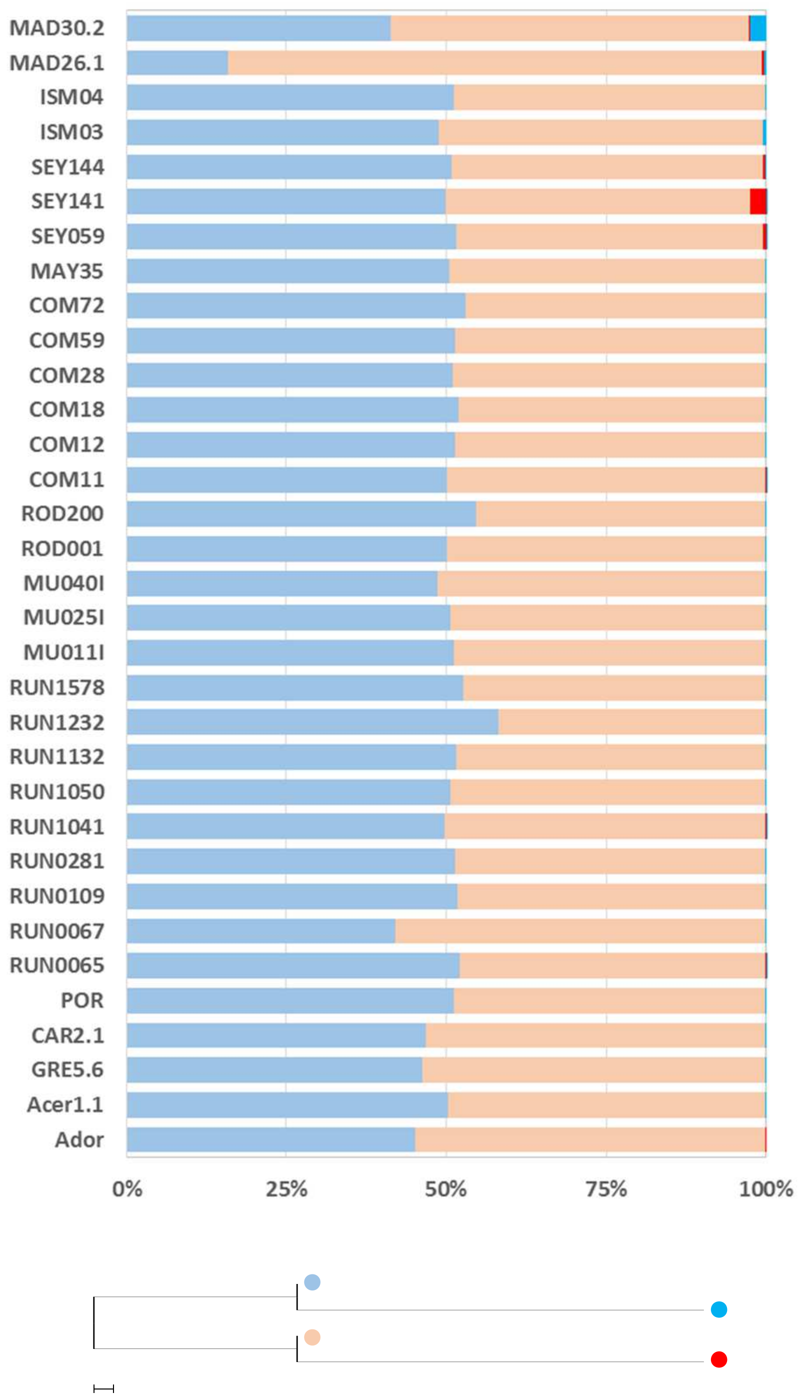

041AF (CR, hexokinase)

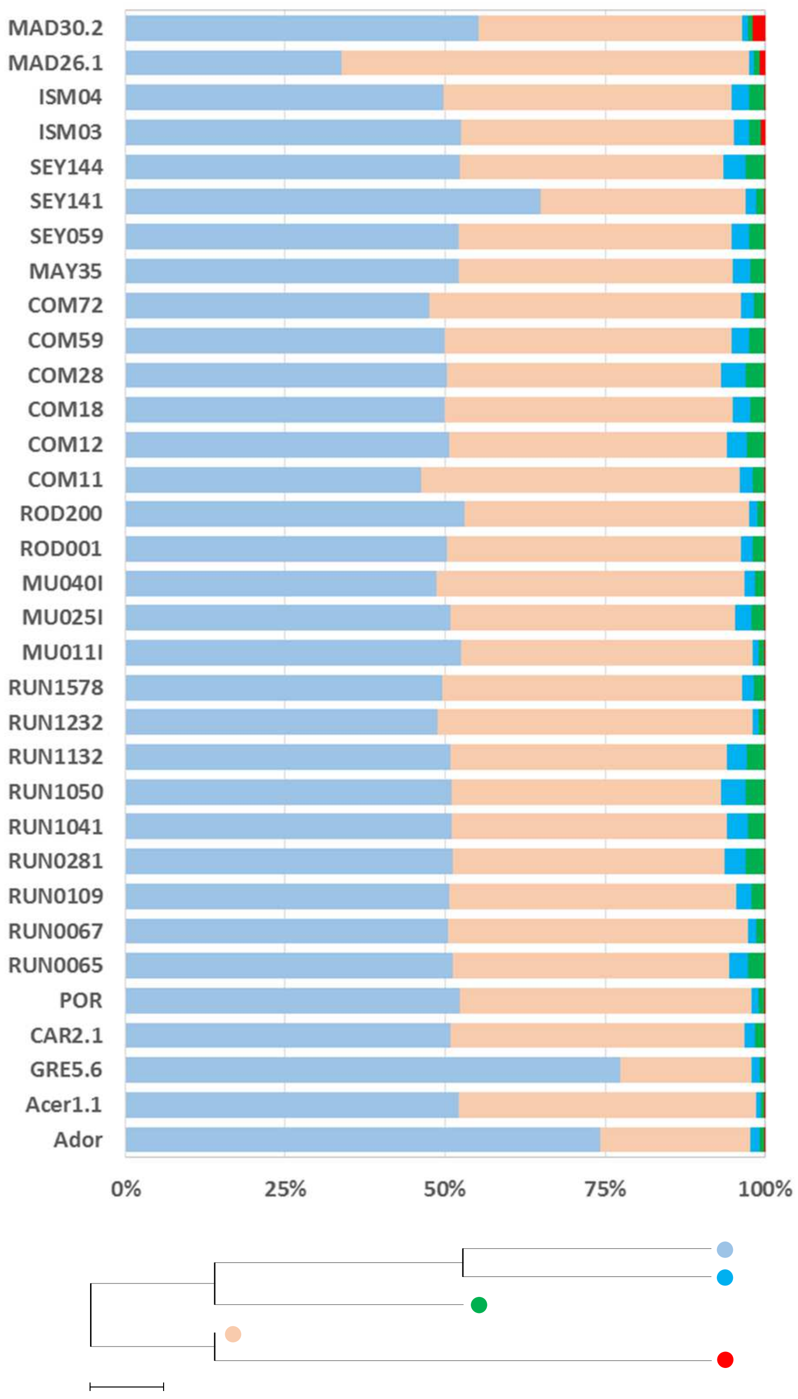

041AR (CR, hexokinase)

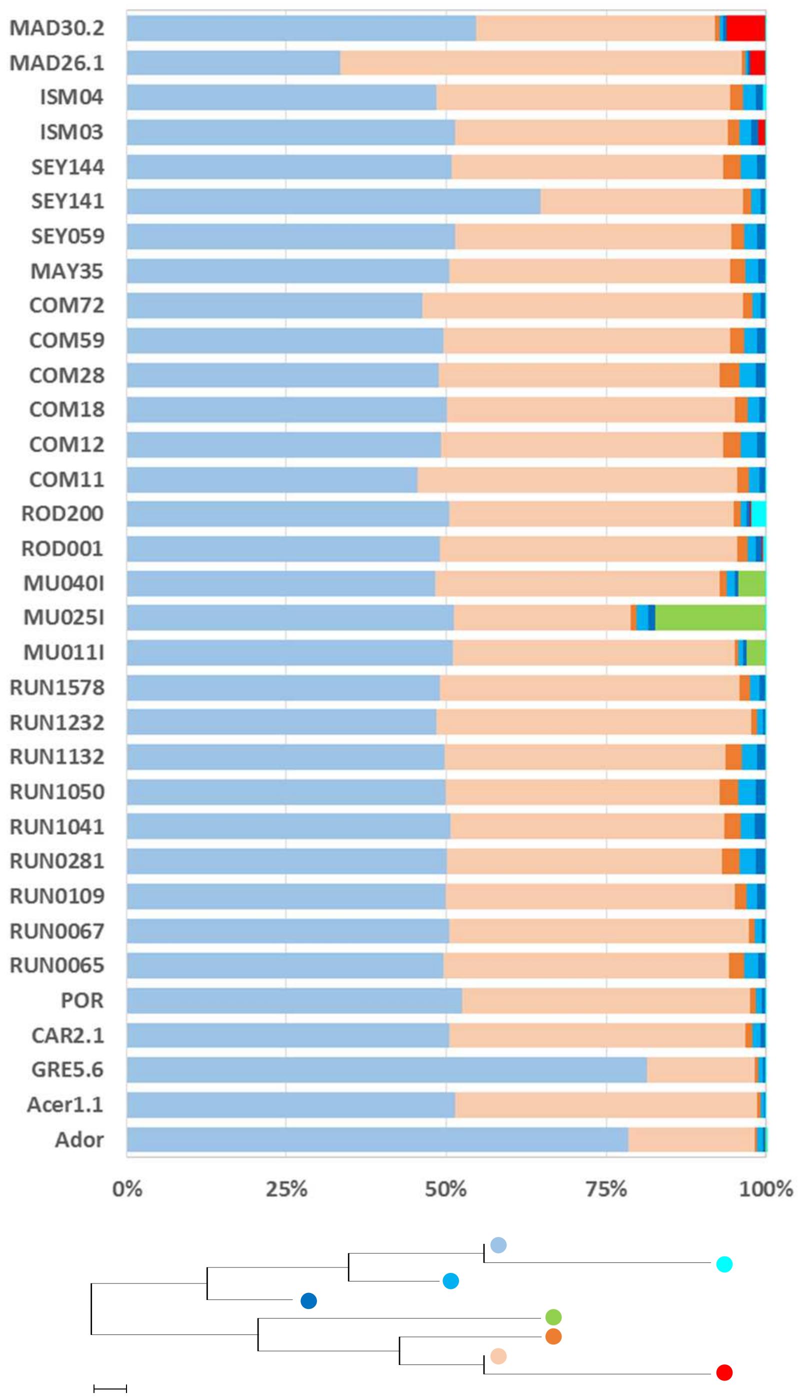

043AF (MS)

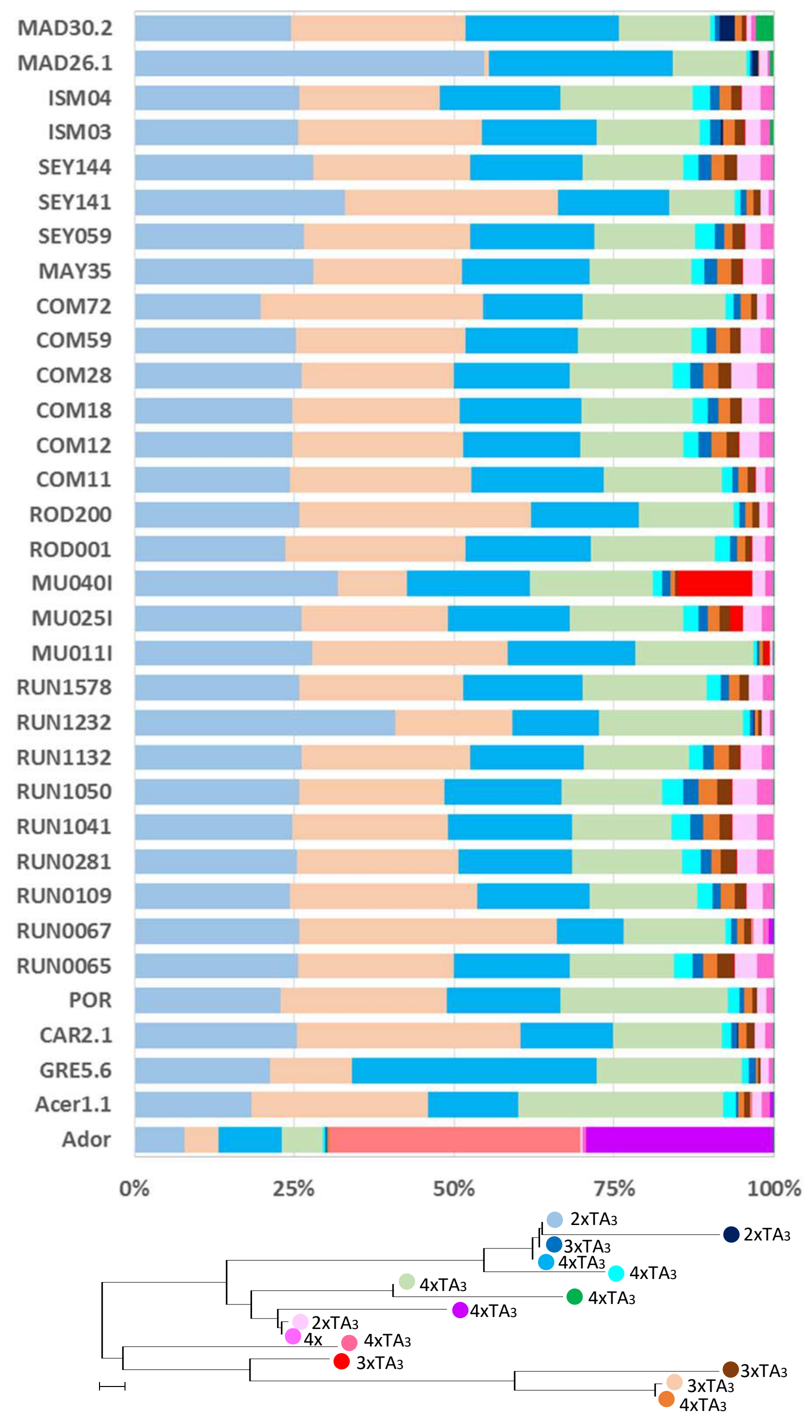

043AR (NC)

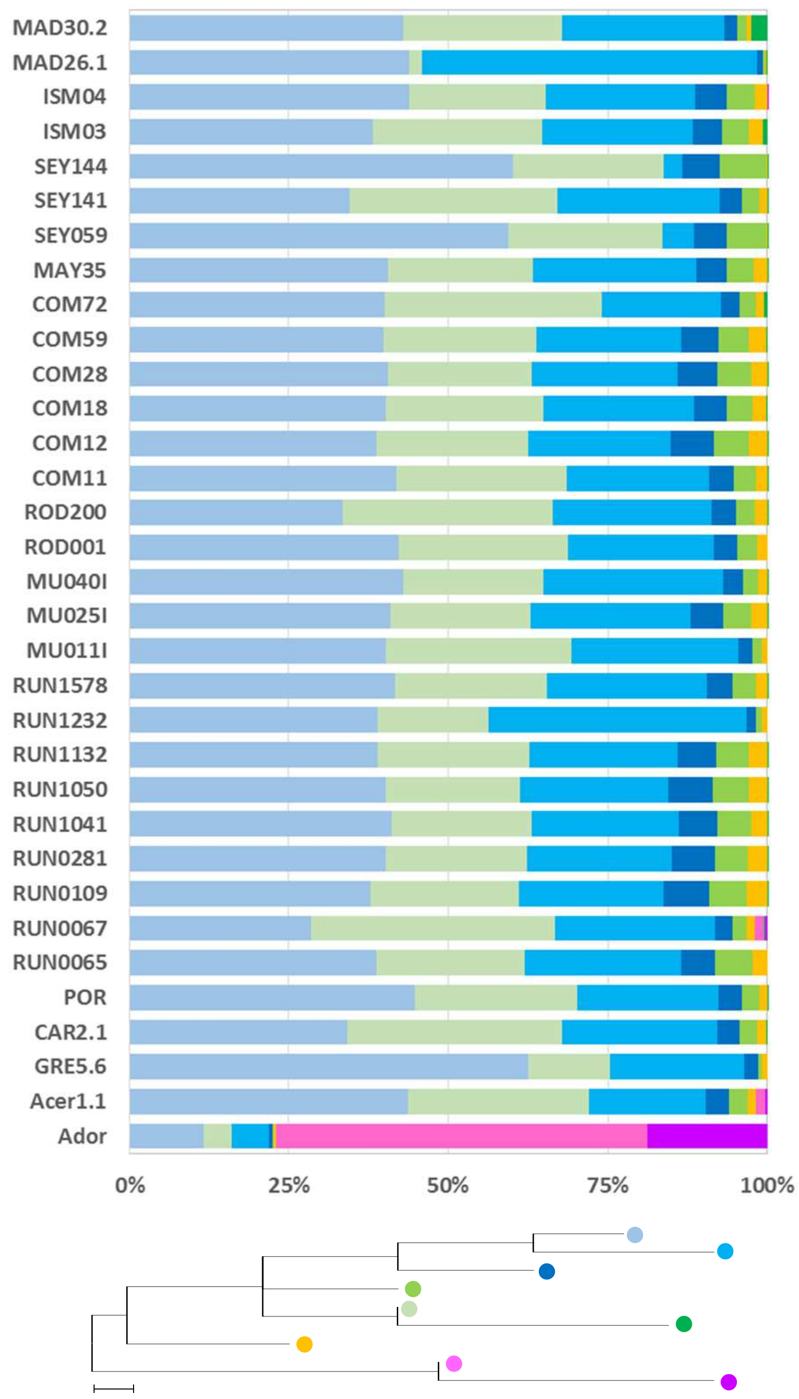

049AF (CR, heat shock protein 70)

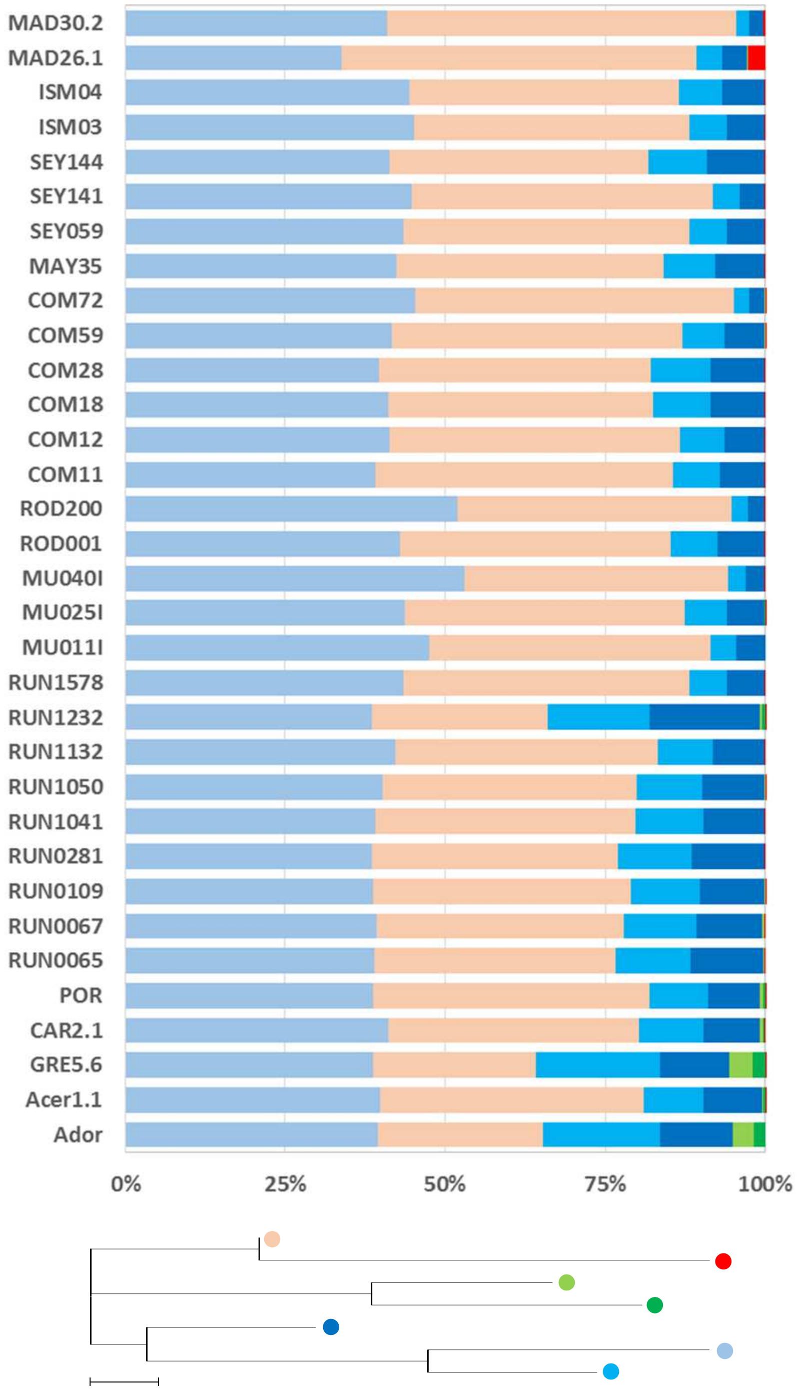

049AR (CR, heat shock protein 70)

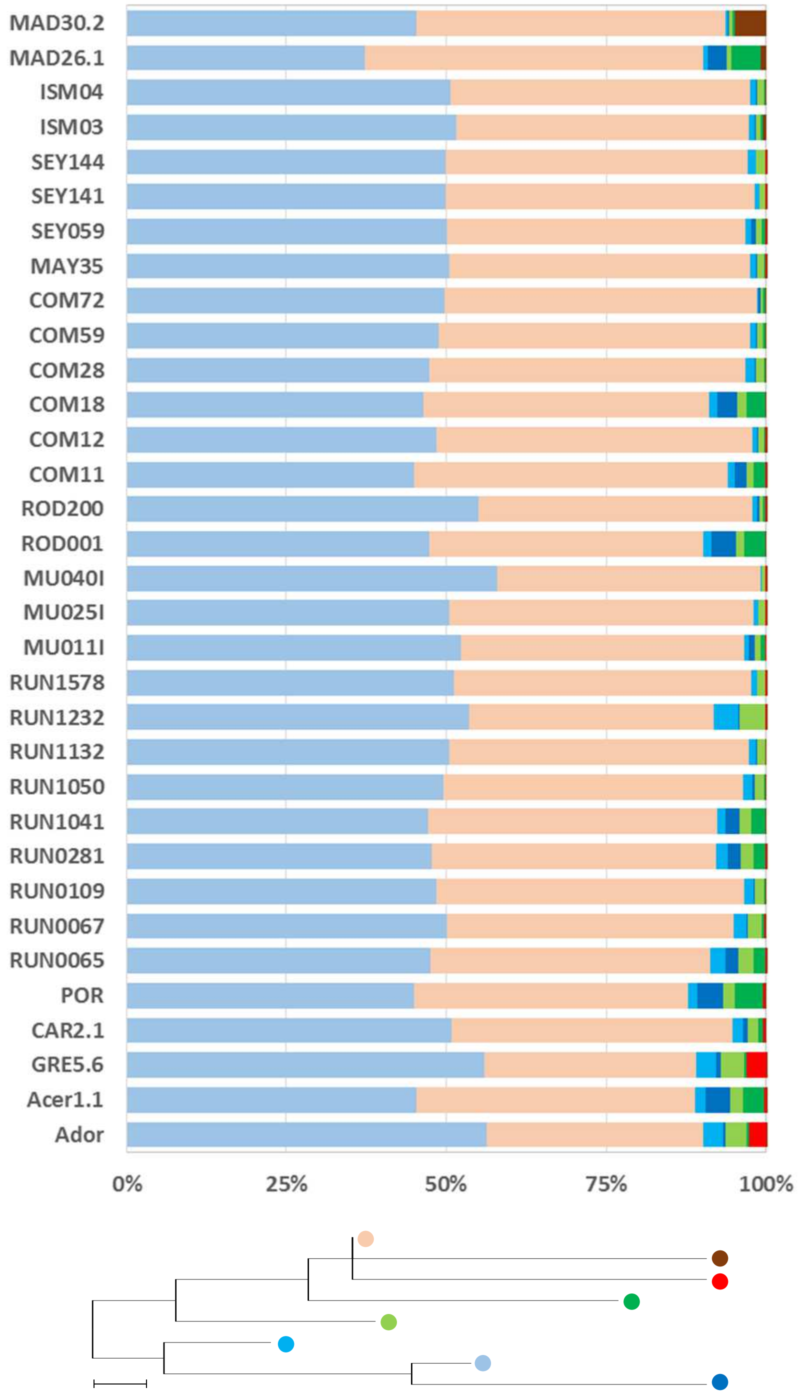

052AF (2 *loci* : MS and NC)

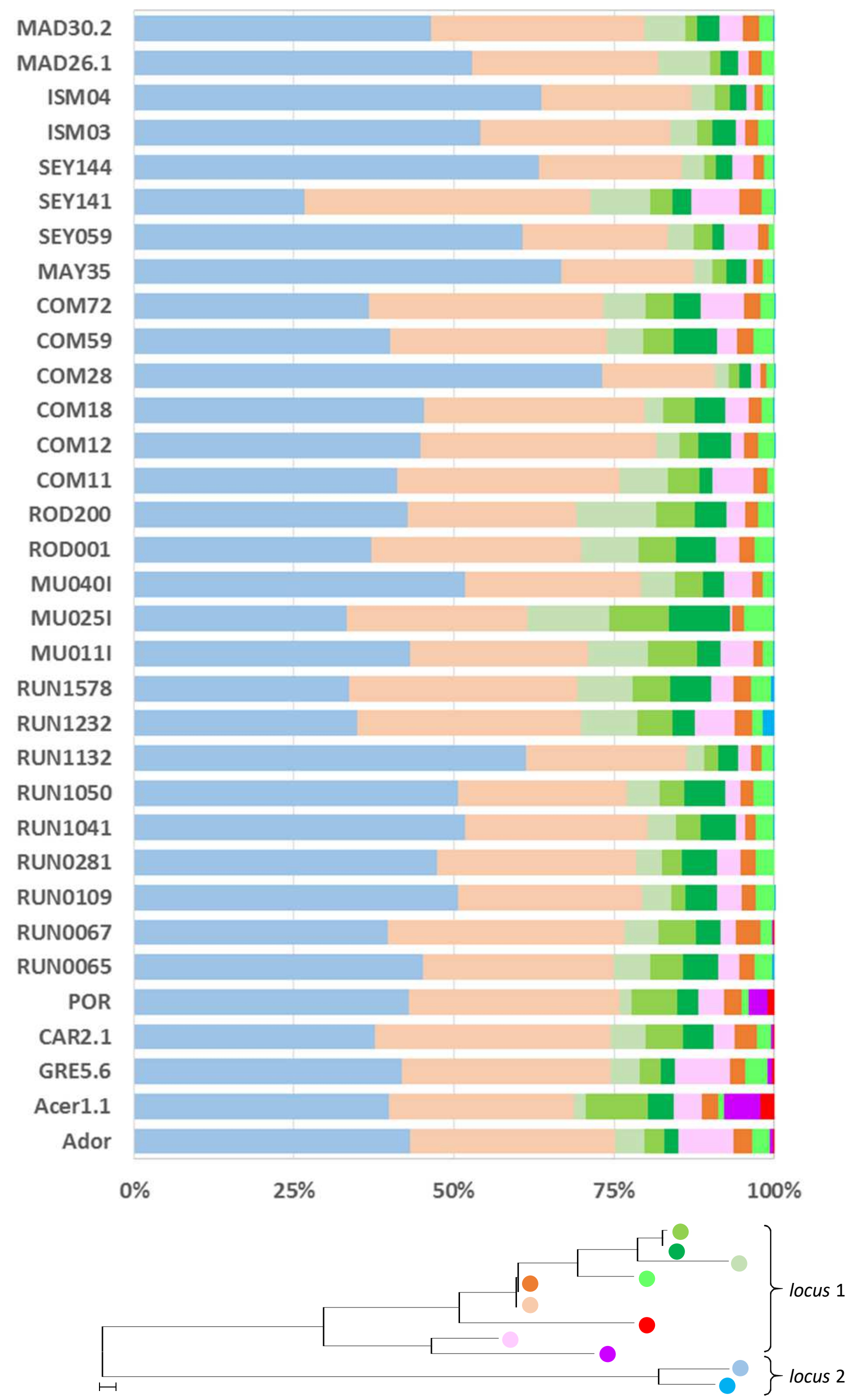

052AR (2 *loci* : NC)

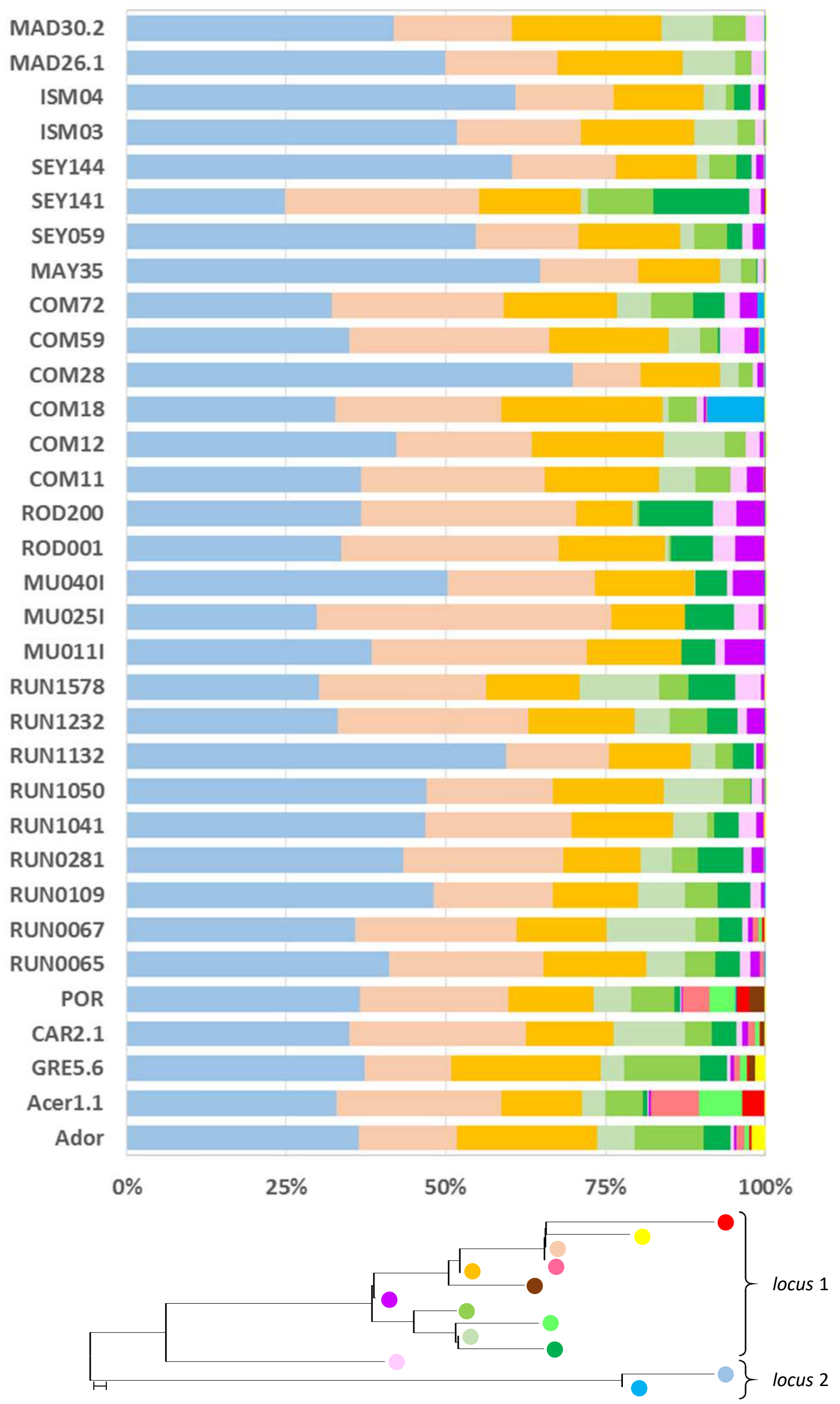

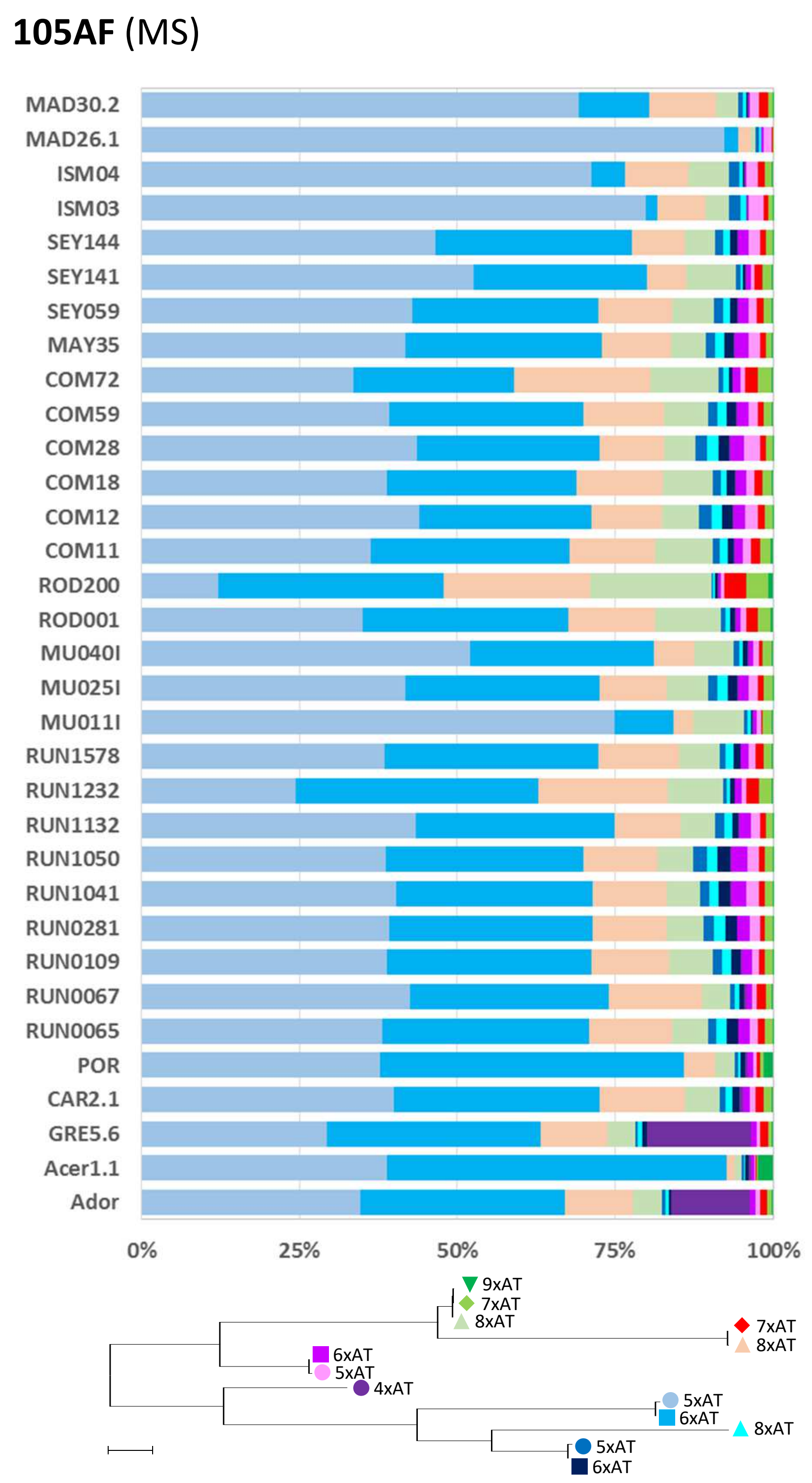
105AF (MS)
MAD30.2
MAD26.1
ISM04
ISM03
SEY144
SEY141
SEY059
MAY35
COM72
COM59
COM28
COM18
COM12
COM11
ROD200
ROD001
MU040I
MU025I
MU011I
RUN1578
RUN1232
RUN1132
RUN1050
RUN1041
RUN0281
RUN0109
RUN0067
RUN0065
POR
CAR2.1
GRE5.6
Acer1.1
Ador
0%
25%
50%
75%
100%
9xAT
7xAT
8xAT
7xAT
8xAT
6xAT
5xAT
4xAT
5xAT
6xAT
8xAT
5xAT
6xAT

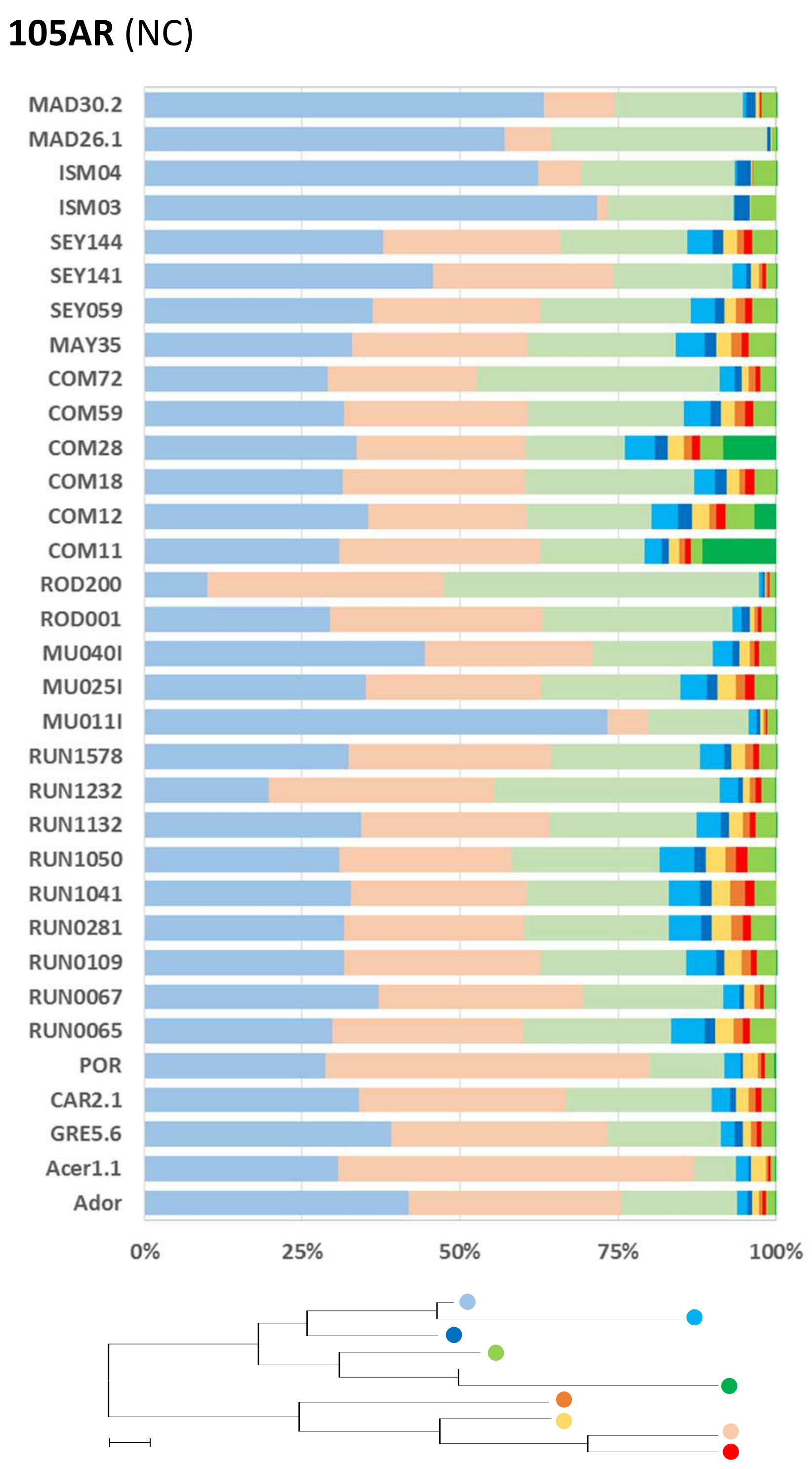
105AR (NC)
MAD30.2
MAD26.1
ISM04
ISM03
SEY144
SEY141
SEY059
MAY35
COM72
COM59
COM28
COM18
COM12
COM11
ROD200
ROD001
MU040I
MU025I
MU011I
RUN1578
RUN1232
RUN1132
RUN1050
RUN1041
RUN0281
RUN0109
RUN0067
RUN0065
POR
CAR2.1
GRE5.6
Acer1.1
Ador
0%
25%
50%
75%
100%

143AF (MS)

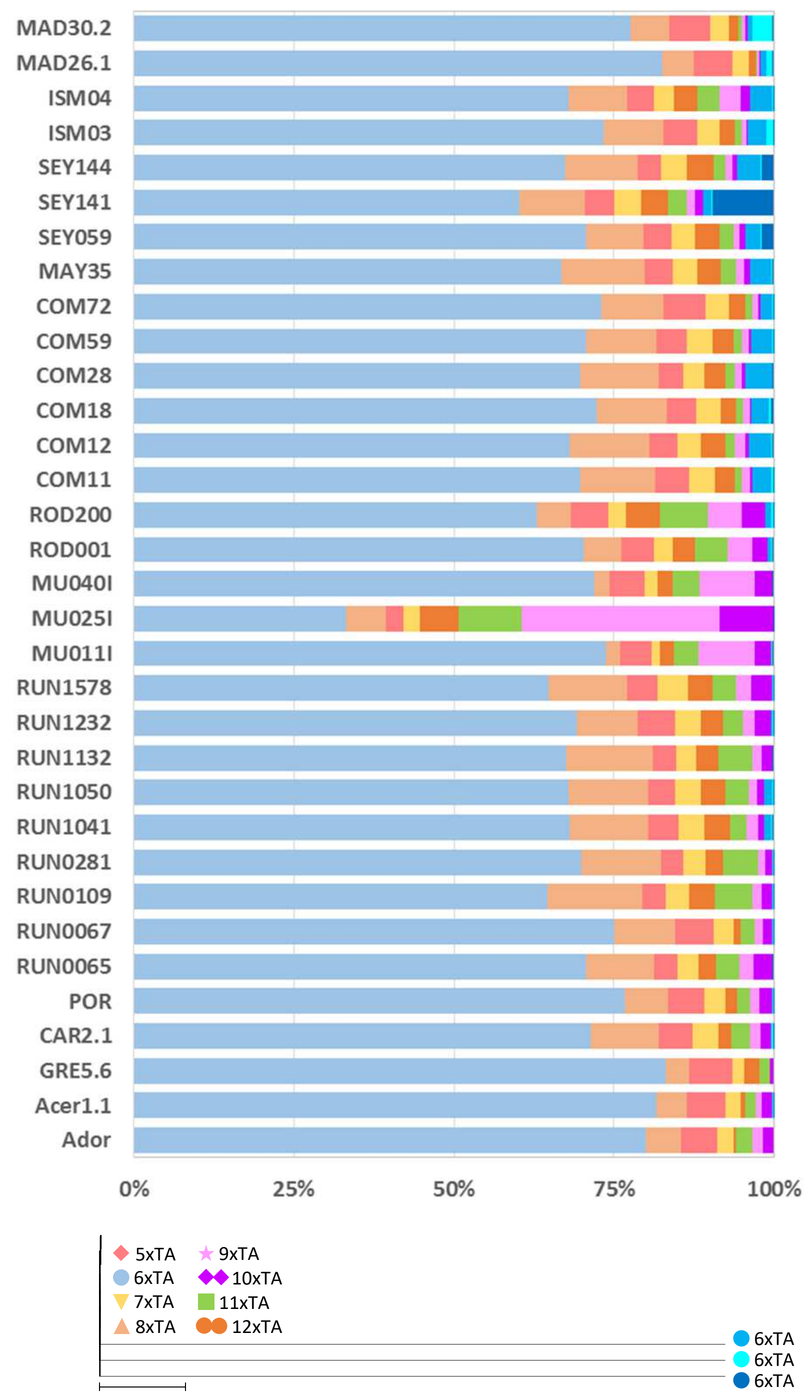

143AR (NC)

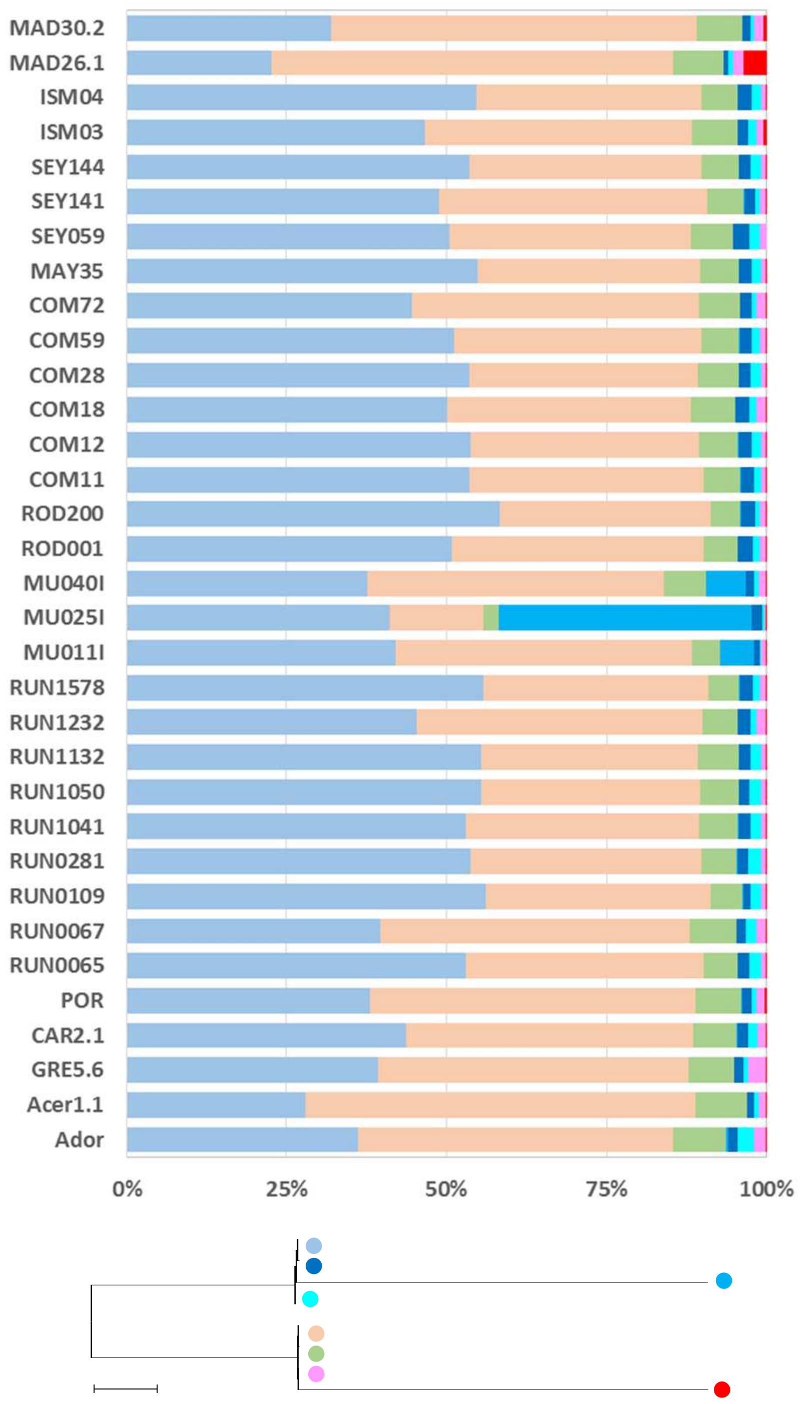

149AF (NC)

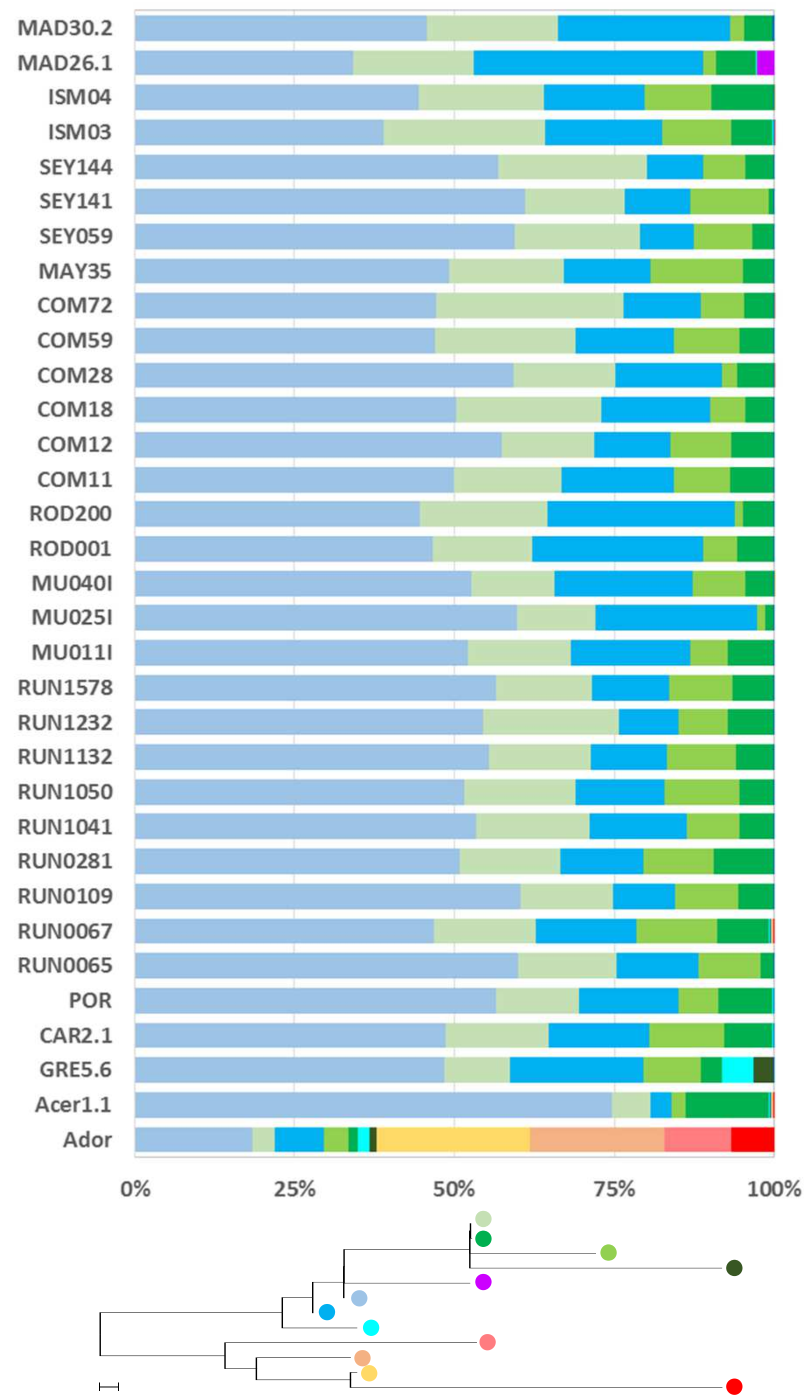

149AR (MS)

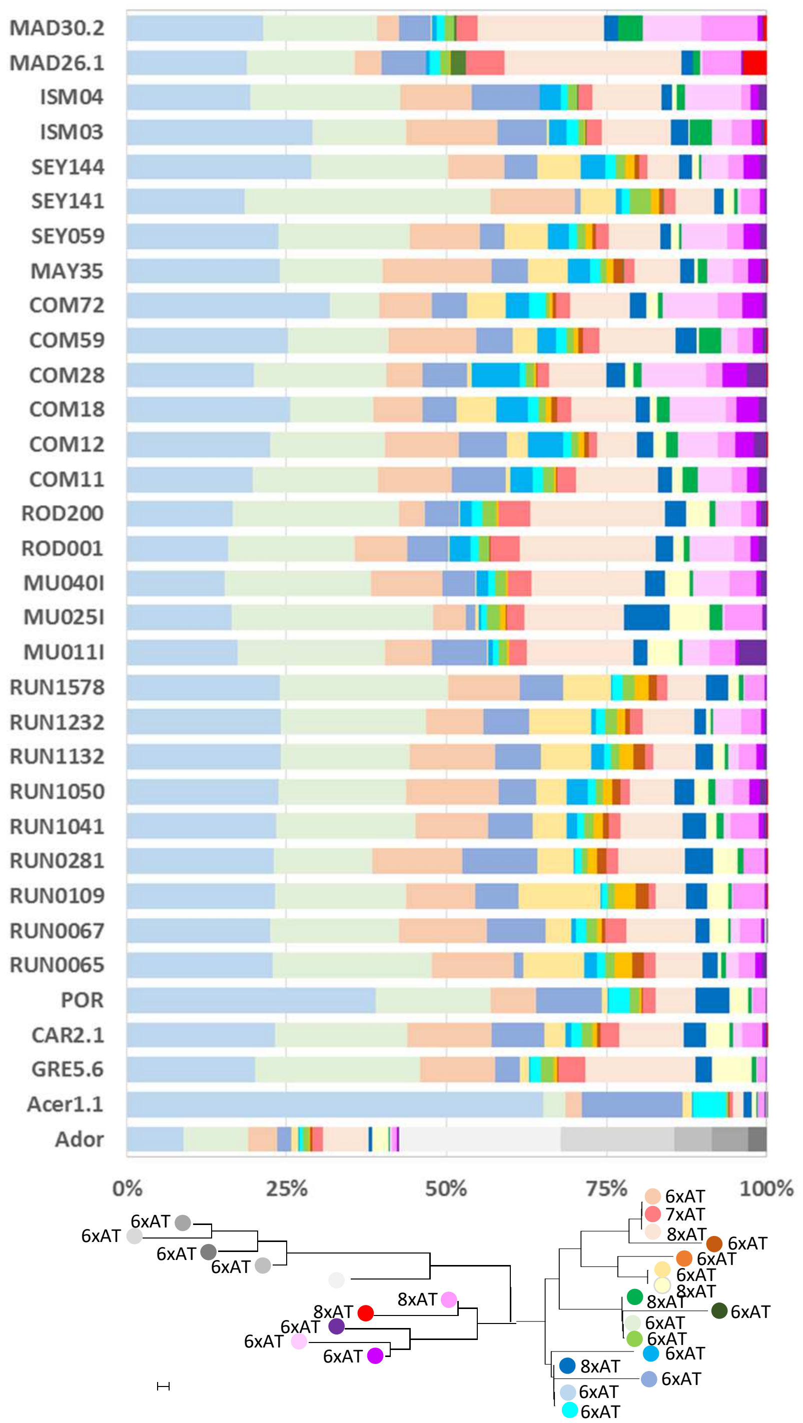

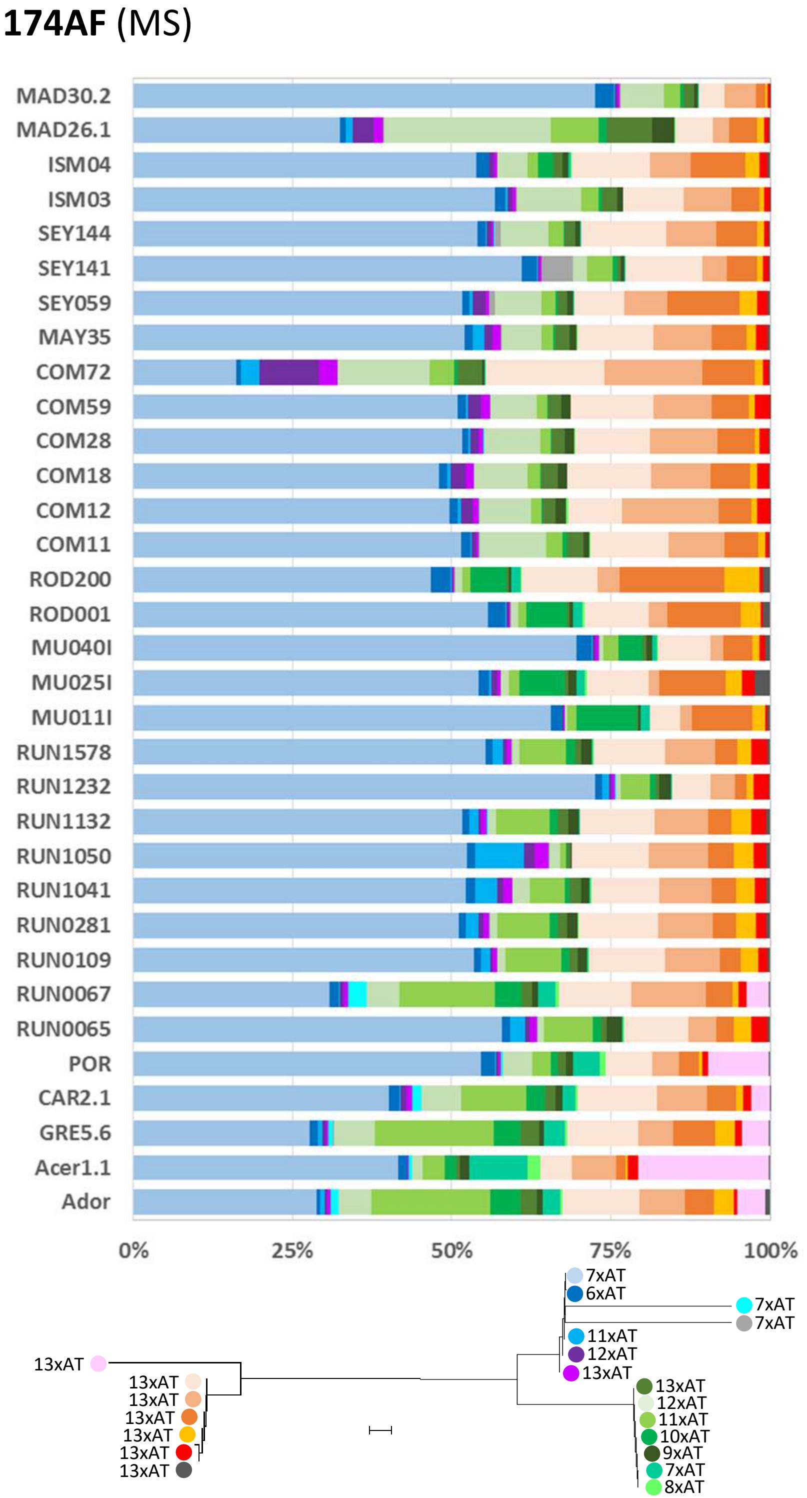
174AF (MS)
MAD30.2
MAD26.1
ISM04
ISM03
SEY144
SEY141
SEY059
MAY35
COM72
COM59
COM28
COM18
COM12
COM11
ROD200
ROD001
MU040I
MU025I
MU011I
RUN1578
RUN1232
RUN1132
RUN1050
RUN1041
RUN0281
RUN0109
RUN0067
RUN0065
POR
CAR2.1
GRE5.6
Acer1.1
Ador
0%
25%
50%
75%
100%
7xAT
6xAT
7xAT
7xAT
11xAT
12xAT
13xAT
13xAT
13xAT
13xAT
13xAT
13xAT
13xAT
13xAT
13xAT
12xAT
11xAT
10xAT
9xAT
7xAT
8xAT

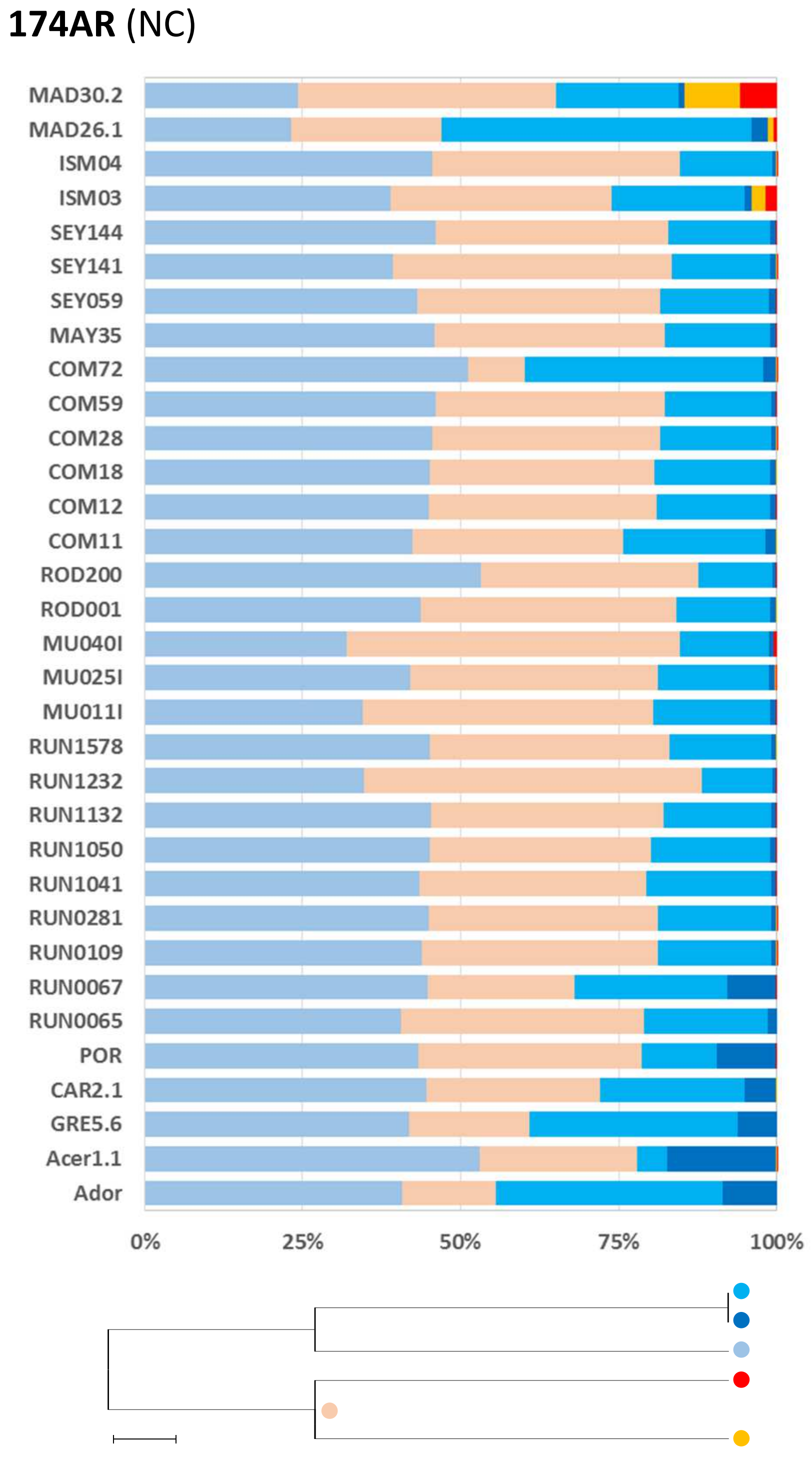
174AR (NC)
MAD30.2
MAD26.1
ISM04
ISM03
SEY144
SEY141
SEY059
MAY35
COM72
COM59
COM28
COM18
COM12
COM11
ROD200
ROD001
MU040I
MU025I
MU011I
RUN1578
RUN1232
RUN1132
RUN1050
RUN1041
RUN0281
RUN0109
RUN0067
RUN0065
POR
CAR2.1
GRE5.6
Acer1.1
Ador
0%
25%
50%
75%
100%

185AF (MS)

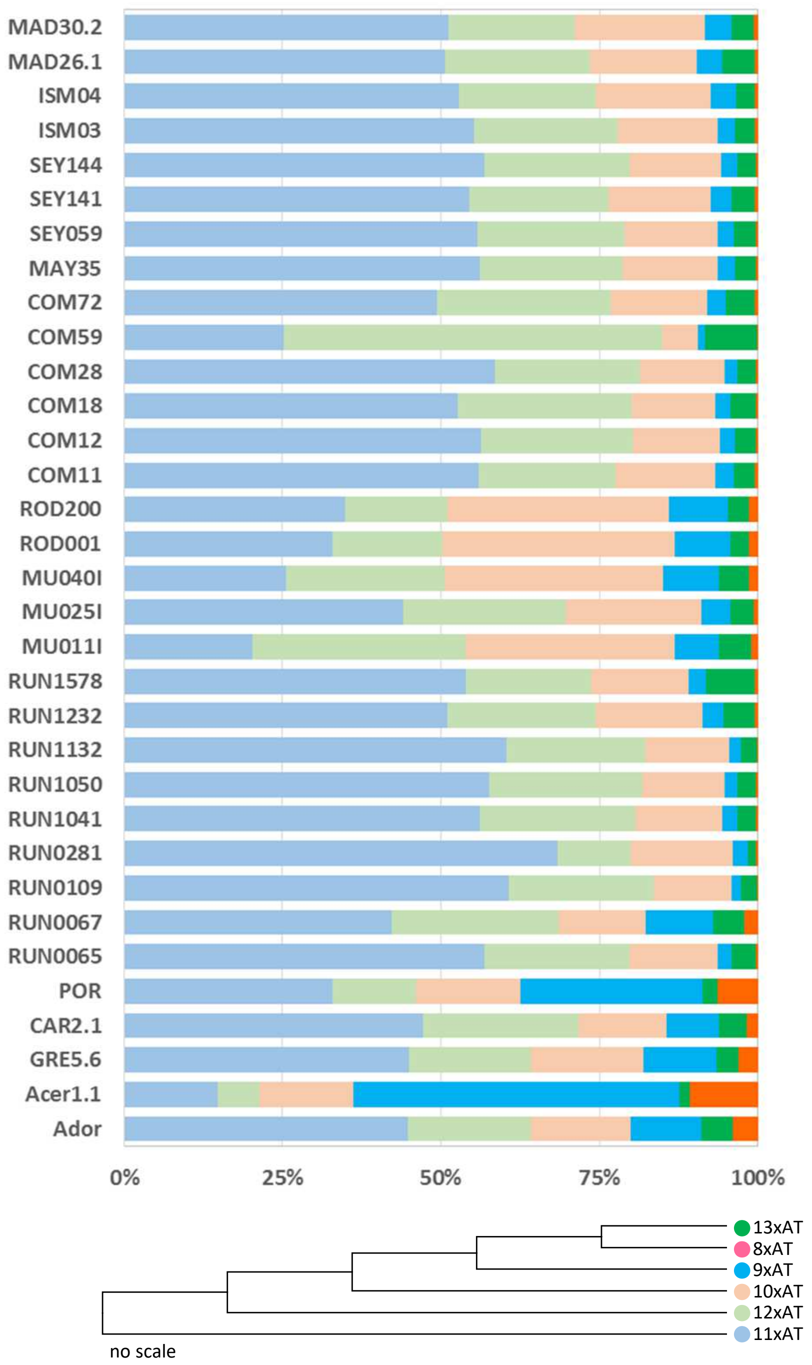

185AR (NC)

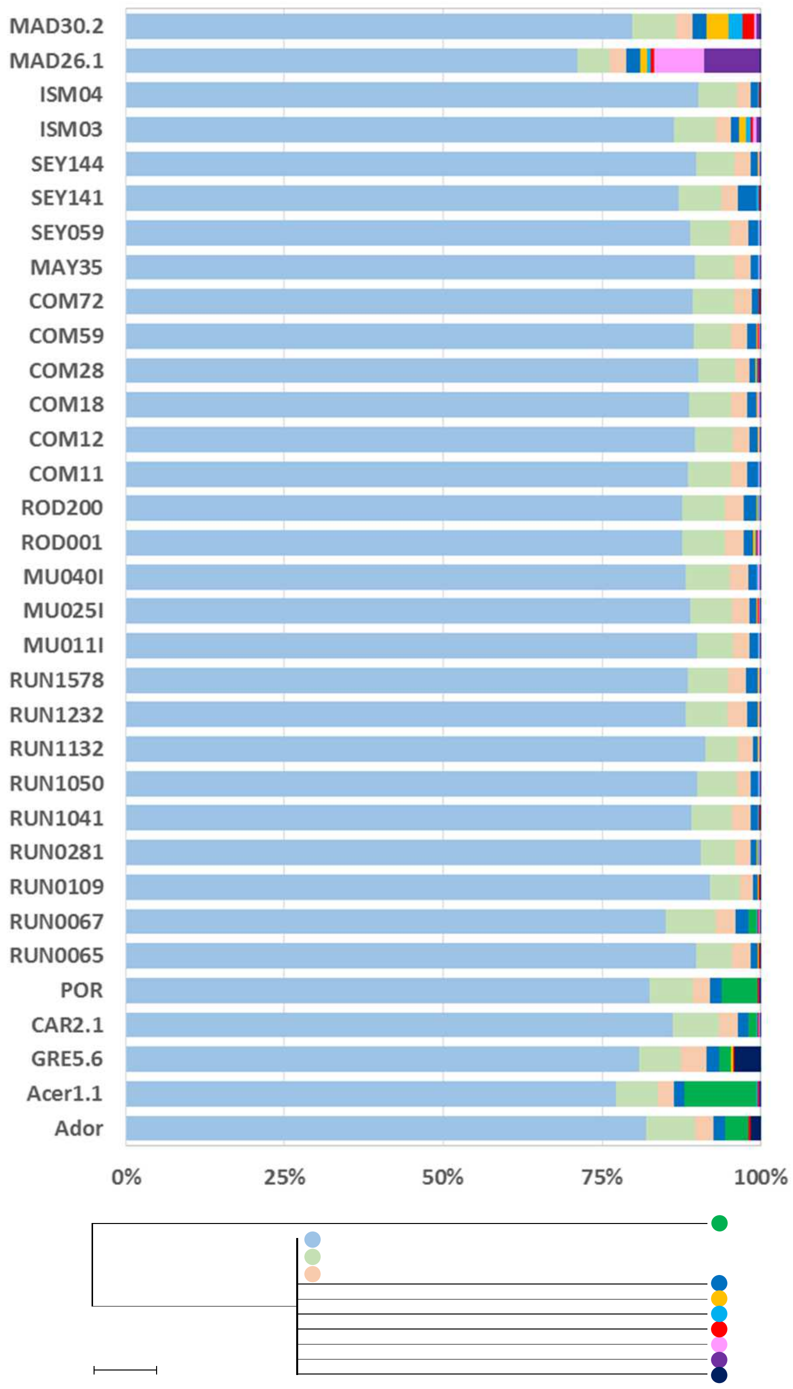

386AF (CR, endospore protein EnPB1)

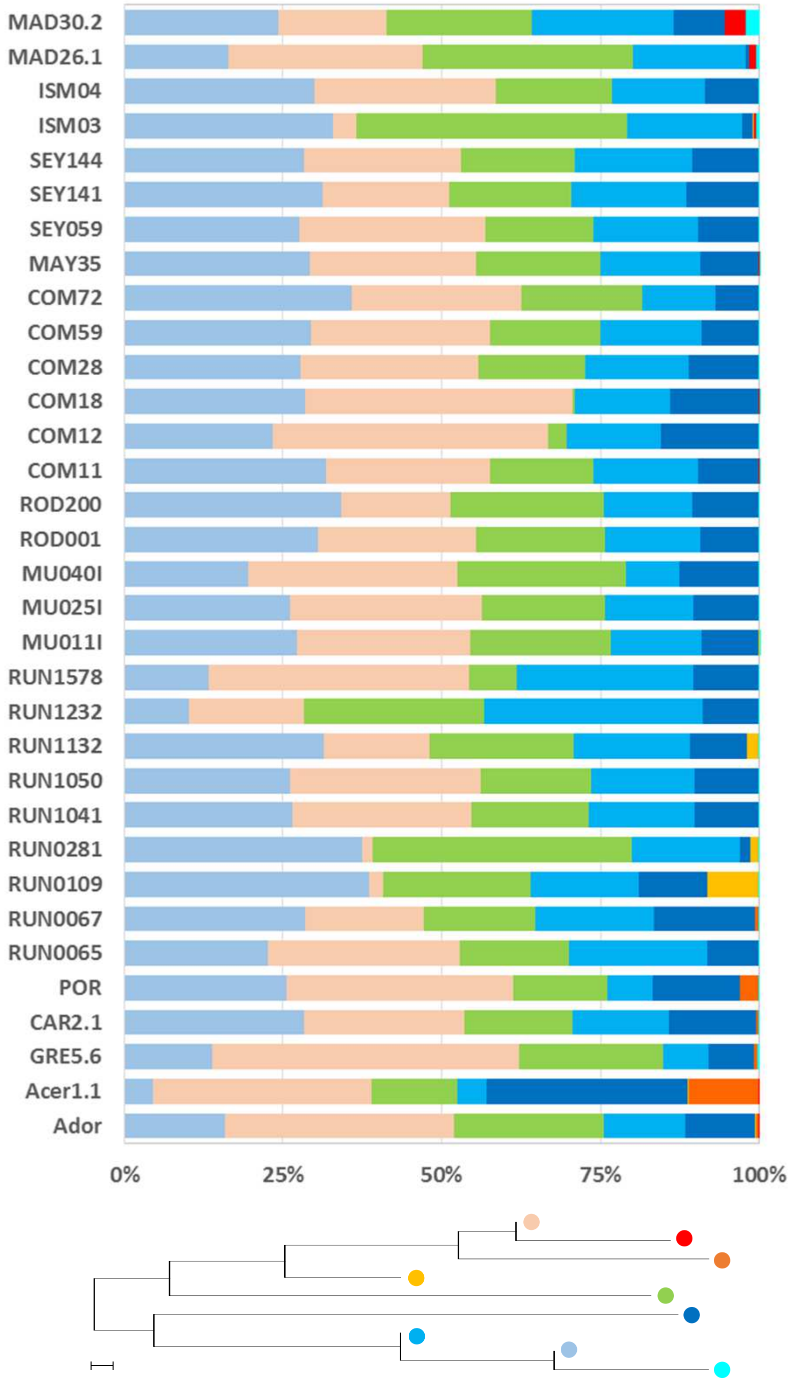

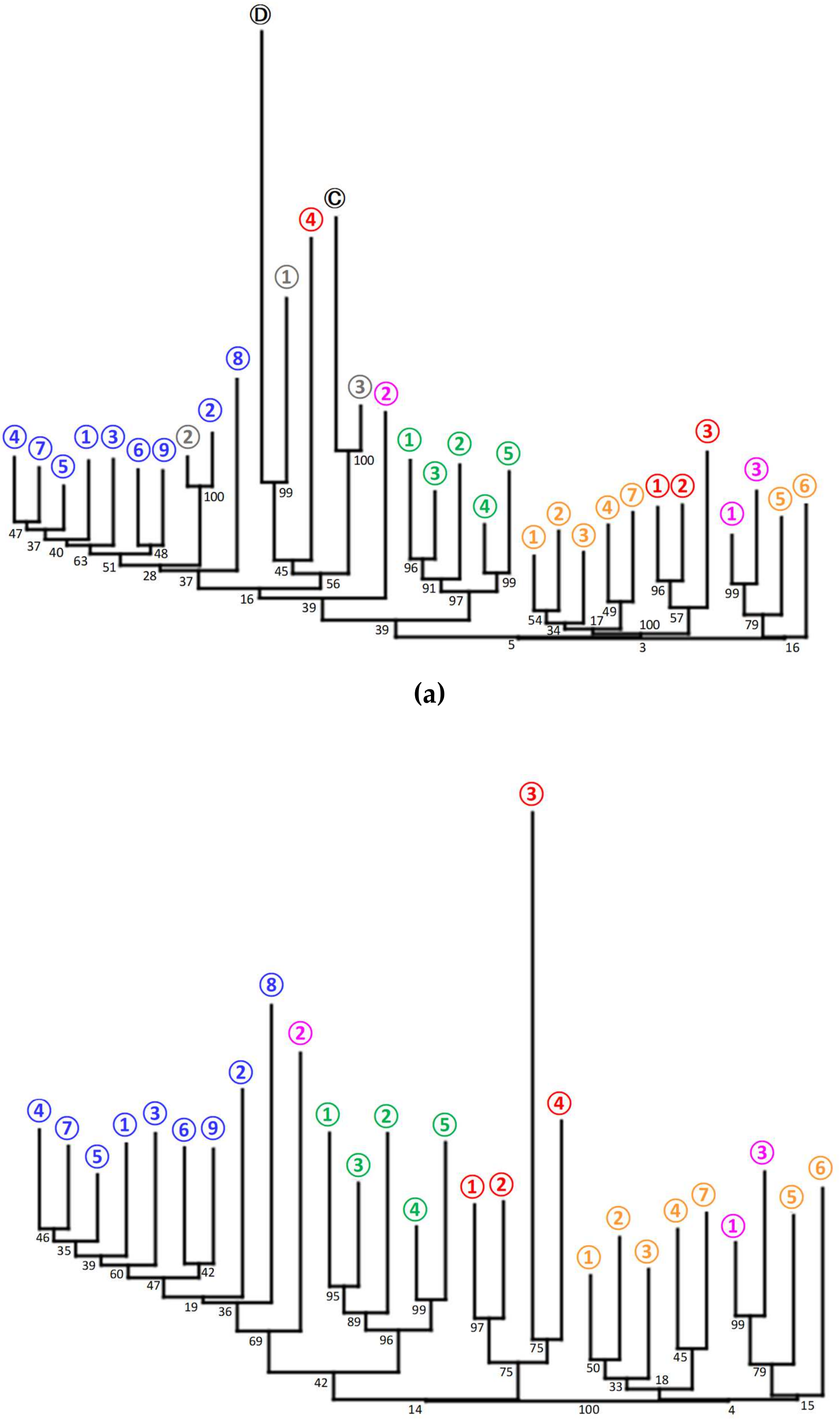

(a)

(b)

Figure S3. Neighbor-Joining clustering of **(a)** all the studied isolates and of **(b)** the SWIO isolates, based on Euclidean similarity matrix computed from the frequencies of 636 haplotypes. Numbers indicate the percentage of replicates supporting the corresponding node, with 10 000 resamplings. Isolates from . European and Asian outgroups are in grey and black respectively. For isolate nomenclature see Table 1.

Table S4. AMOVA analyses of 83 markers in the 28 SWIO isolates, with 5 defined geographic groups: La reunion, Mauritius, the Comoros (incl. Mayotte), the Seychelles, Madagascar (df=4 among groups, df=23 among populations within groups, df=279961 to 279983 within populations, 10000 permutations).

	Variance of component			indices			*p*-values		
Marker	Va	Vb	Vc	FCT	FSC	FST	Va & FCT	Vb & FSC	Va & FCT
001AF	0,00039	0,00055	0,25386	0,0015	0,0022	0,0037	0,007	0	0
001AR	0,00000	0,00010	0,05978	0,0000	0,0016	0,0016	0,403	0	0
001BF	-0,00002	0,00066	0,01510	-0,0012	0,0419	0,0407	0,545	0	0
001BR	0,00067	0,00176	0,25284	0,0026	0,0069	0,0095	0,008	0	0
001CF	0,00226	0,00438	0,24985	0,0088	0,0172	0,0259	0,008	0	0
001CR	0,00003	0,00114	0,01855	0,0017	0,0579	0,0595	0,306	0	0
001DF	0,00071	0,00202	0,24672	0,0028	0,0081	0,0109	0,026	0	0
001DR	0,00000	0,00000	0,00000	0,0000	0,0000	0,0000	1,000	1	1
001EF	0,01595	0,01715	0,27064	0,0525	0,0596	0,1090	0,000	0	0
001ER	-0,00009	0,00114	0,00975	-0,0079	0,1050	0,0979	0,696	0	0
002AF	0,00211	0,00352	0,35528	0,0059	0,0098	0,0156	0,002	0	0
002AR	0,00001	0,00001	0,00458	0,0029	0,0027	0,0056	0,000	0	0
003AF	0,00009	0,00026	0,07995	0,0012	0,0032	0,0044	0,025	0	0
003AR	0,00008	0,00431	0,38499	0,0002	0,0111	0,0113	0,338	0	0
003BF	0,00060	0,00869	0,29176	0,0020	0,0289	0,0309	0,200	0	0
003BR	0,00220	0,01262	0,16857	0,0120	0,0696	0,0808	0,112	0	0
004AF	0,00188	0,00811	0,37327	0,0049	0,0213	0,0261	0,026	0	0
004AR	0,00112	0,00517	0,32682	0,0033	0,0156	0,0189	0,040	0	0
004BF	0,02905	0,00667	0,26185	0,0976	0,0248	0,1200	0,000	0	0
004BR	0,00191	0,00293	0,15485	0,0119	0,0186	0,0303	0,003	0	0
005AF	0,00000	0,00009	0,00744	0,0002	0,0121	0,0123	0,356	0	0
005AR	0,00078	0,00225	0,02029	0,0333	0,1000	0,1300	0,014	0	0
005BF	0,00049	0,00135	0,01846	0,0242	0,0682	0,0907	0,021	0	0
005BR	0,00004	0,00007	0,00802	0,0043	0,0081	0,0124	0,000	0	0
006AF	0,00027	0,00506	0,32848	0,0008	0,0152	0,0160	0,235	0	0
006AR	0,00028	0,00443	0,38601	0,0007	0,0113	0,0120	0,235	0	0
009AF	0,00829	0,00852	0,31970	0,0246	0,0260	0,0500	0,001	0	0
009AR	0,00926	0,00391	0,19872	0,0437	0,0193	0,0621	0,000	0	0
011AF	0,00095	0,00348	0,28956	0,0032	0,0119	0,0151	0,057	0	0
011AR	0,00208	0,00905	0,32842	0,0061	0,0268	0,0328	0,051	0	0
011BF	0,00332	0,00524	0,26084	0,0123	0,0197	0,0318	0,010	0	0
011BR	-0,00071	0,02561	0,33473	-0,0020	0,0711	0,0692	0,556	0	0
013AF	0,01094	0,00053	0,04104	0,2083	0,0127	0,2184	0,000	0	0
013AR	0,00677	0,01369	0,20883	0,0295	0,0615	0,0892	0,018	0	0
013BF	0,00453	0,01293	0,06925	0,0522	0,1573	0,2013	0,014	0	0
013BR	0,02729	0,00660	0,17228	0,1323	0,0369	0,1644	0,000	0	0
014AF	-0,00036	0,00543	0,01077	-0,0224	0,3353	0,3204	0,674	0	0
014AR	-0,00020	0,00360	0,25865	-0,0008	0,0137	0,0130	0,631	0	0
016AF	0,03382	0,00832	0,20797	0,1352	0,0385	0,1685	0,000	0	0
016AR	0,03947	0,01498	0,21637	0,1458	0,0648	0,2011	0,000	0	0
016BF	0,00044	0,00321	0,24835	0,0018	0,0128	0,0145	0,169	0	0
016BR	0,01765	0,00414	0,20235	0,0788	0,0201	0,0972	0,000	0	0
017AF	0,00682	0,00660	0,33081	0,0198	0,0196	0,0390	0,001	0	0
017AR	0,00588	0,00668	0,30946	0,0183	0,0211	0,0390	0,005	0	0
019AF	0,00446	0,00652	0,31645	0,0136	0,0202	0,0335	0,001	0	0
019AR	0,00001	0,00004	0,00853	0,0011	0,0049	0,0060	0,028	0	0
019BF	0,00232	0,00435	0,24099	0,0094	0,0177	0,0269	0,013	0	0
019BR	0,00172	0,00518	0,26538	0,0063	0,0192	0,0254	0,034	0	0
019CF	0,00231	0,00883	0,28860	0,0077	0,0297	0,0372	0,025	0	0
019CR	0,00145	0,00848	0,24430	0,0057	0,0336	0,0391	0,126	0	0
020AF	0,00244	0,01152	0,33828	0,0069	0,0329	0,0396	0,069	0	0
020AR	0,00348	0,00698	0,36020	0,0094	0,0190	0,0282	0,000	0	0
021AF	0,00498	0,00920	0,36501	0,0131	0,0246	0,0374	0,001	0	0
021AR	0,00546	0,00925	0,32083	0,0163	0,0280	0,0438	0,001	0	0
021BF	0,00198	0,00562	0,24630	0,0078	0,0223	0,0299	0,022	0	0
021BR	0,00228	0,00657	0,24265	0,0091	0,0263	0,0352	0,021	0	0
025AF	-0,00008	0,00285	0,30629	-0,0003	0,0092	0,0090	0,580	0	0
025AR	0,00024	0,00130	0,01190	0,0179	0,0986	0,1147	0,106	0	0
034AF	0,00024	0,00227	0,24630	0,0009	0,0091	0,0101	0,217	0	0
034AR	0,00044	0,00241	0,27009	0,0016	0,0089	0,0104	0,047	0	0
036AF	0,00084	0,00409	0,19247	0,0043	0,0208	0,0250	0,083	0	0
036AR	0,00134	0,00407	0,24662	0,0053	0,0162	0,0215	0,028	0	0
041AF	0,00035	0,00207	0,26858	0,0013	0,0077	0,0089	0,123	0	0
041AR	0,00049	0,00310	0,27438	0,0018	0,0112	0,0129	0,084	0	0
043AF	0,00013	0,00593	0,39096	0,0003	0,0150	0,0153	0,352	0	0
043AR	0,00202	0,00625	0,34747	0,0057	0,0177	0,0232	0,012	0	0
049AF	0,00163	0,00195	0,31009	0,0052	0,0062	0,0114	0,000	0	0
049AR	0,00033	0,00103	0,26983	0,0012	0,0038	0,0050	0,006	0	0
052AF	-0,00021	0,00932	0,32734	-0,0006	0,0277	0,0271	0,542	0	0
052AR	0,00217	0,01060	0,34940	0,0060	0,0294	0,0353	0,073	0	0
105AF	0,01585	0,00881	0,32782	0,0450	0,0262	0,0699	0,000	0	0
105AR	0,01014	0,01053	0,34558	0,0277	0,0296	0,0564	0,002	0	0
143AF	0,00216	0,00472	0,25042	0,0084	0,0185	0,0267	0,001	0	0
143AR	0,00195	0,00838	0,29204	0,0065	0,0279	0,0342	0,038	0	0
149AF	0,00367	0,00329	0,32591	0,0110	0,0100	0,0209	0,000	0	0
149AR	0,00367	0,00403	0,42984	0,0084	0,0093	0,0176	0,000	0	0
174AF	0,00274	0,00938	0,33759	0,0078	0,0270	0,0347	0,022	0	0
174AR	0,00133	0,00788	0,31731	0,0041	0,0242	0,0282	0,061	0	0
185AF	0,00756	0,00742	0,31637	0,0228	0,0229	0,0452	0,000	0	0
185AR	0,00039	0,00080	0,10889	0,0035	0,0073	0,0108	0,007	0	0
386AF	0,00173	0,01215	0,37858	0,0044	0,0311	0,0354	0,089	0	0

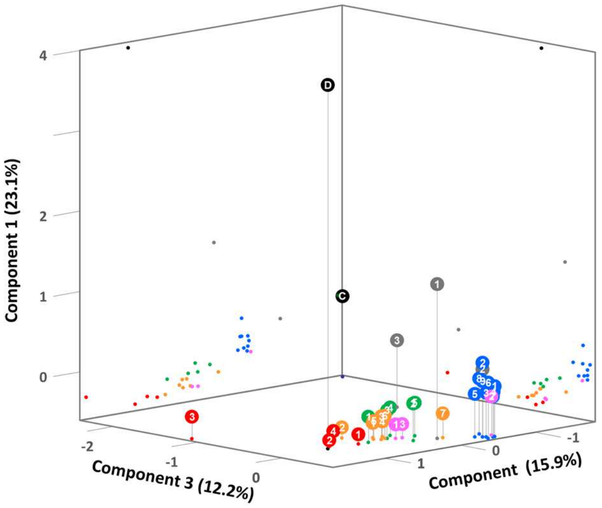

(a)

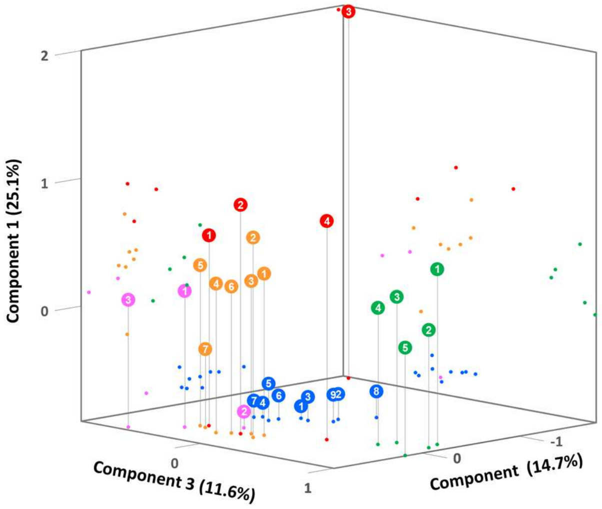

(b)

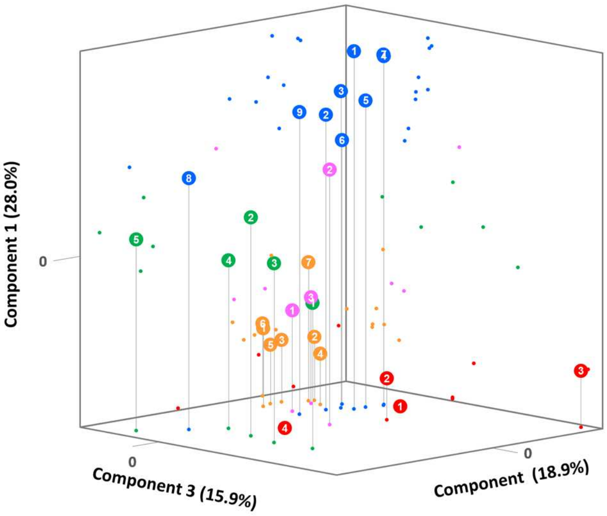

(c)

Figure S4. Effect of the reduction of sequencing coverage on isolates distribution. PCAs obtained considering all **(a)** or only SWIO isolates **(b)** using a model with all the 83 markers but only 20 reads per marker and per isolates with the removal of singletons (unique haplotypes in one isolate). Below 20 reads the SWIO isolates clustered together but with blurred differentiation. In **(c)** only microsatellite haplotypes were used for the SWIO isolates. Same legend as Figure 4.

Table S5 : List of the 485 polymorphic sites detected in the 636 haplotypes. The position is given according to the sequence of the most abundant haplotype (#01) as a reference except for 052AF where it was haplotype 052AF02. Any adjacent polymorphic positions were considered as a single site. Thus every microsatellite was considered as a single polymorphic site with several derived characters. The SP from contig JPQZ01000088 where removeds for 052AF and 052AR data. SNP : single nucleotide polymorphism, xNP : x-nt long polymorphism.

SP	Marker	Marker	SP	Position	Dominant character		Modified character's				
#	name	type	type	(hap1)	sequence	min-max %	sequence	Ts/Tv	Syn	nSyn	% min-max
SP001	001AF	CR	SNP	36	A	97,8-99,9	G	Ts	n	T>A	0,1-2,2
SP002	001AF	CR	SNP	68	T	97,6-99,9	C	Ts	o		0,07-2,4
SP003	001AF	CR	SNP	79	A	98-99,9	G	Ts	n	N>S	0,08-2
SP004	001AF	CR	SNP	122	C	36,9-63	T	Ts	o		37-63,1
SP005	001AF	CR	SNP	143	T	97,5-99,9	C	Ts	o		0,08-2,5
SP006	001AR	CR	SNP	12	A	96,8-97,7	C	Tv	n	L>F	2,3-3,2
SP007	001AR	CR	SNP	54	A	96,9-97,8	C	Tv	o		2,3-3,1
SP008	001AR	CR	SNP	118	G	96,7-100	A	Ts	n	E>K	0,02-3,3
SP009	001AR	CR	SNP	140	A	95,7-99,7	G	Ts	o		0,31-4,3
SP010	001BF	CR	SNP	36	A	96,1-99,9	G	Ts	n	Q>K	0,15-3,9
SP011	001BF	CR	SNP	67	G	94,7-100	A	Ts	o		0,05-5,3
SP012	001BF	CR	SNP	73	G	93,9-100	A	Ts	o		0,03-6,1
SP013	001BF	CR	SNP	77	C	96,3-100	T	Ts	n	stop	0,03-3,7
SP014	001BF	CR	SNP	90	T	83,1-99,9	C	Ts	n	V>A	0,07-16,9
SP015	001BF	CR	SNP	129	C	97,1-100	T	Ts	n	T>I	0,05-2,9
SP016	001BR	CR	SNP	30	C	97,9-100	T	Ts	n	A>V	0,02-2,1
SP017	001BR	CR	SNP	77	G	89,3-100	A	Ts	n	E>K	0-10,7
SP018	001BR	CR	SNP	84	G	97,7-100	A	Ts	o		0-2,4
SP019	001BR	CR	SNP	91	G	93,8-99,9	A	Ts	o		0,07-6,2
SP020	001BR	CR	SNP	107	G	38,8-82,6	A	Ts	o		17,5-61,2
SP021	001CF	CR	SNP	54	A	81,7-100	C	Tv	n	N>T	0-18,3
SP022	001CF	CR	SNP	64	C	27,5-67,9	T	Ts	o		32,1-72,6
SP023	001CF	CR	SNP	148	A	96,5-99,9	G	Ts	o		0,09-3,5
SP024	001CR	CR	SNP	20	A	84,2-100	G	Ts	o		0-15,8
SP025	001CR	CR	SNP	28	G	84,2-100	A	Ts	o		0-15,8
SP026	001CR	CR	SNP	33	A	97,7-99,9	G	Ts	n	D>G	0,13-2,3
SP027	001CR	CR	SNP	47	C	92,6-100	T	Ts	n	L>F	0-7,4
SP028	001CR	CR	SNP	81	A	83,7-99,8	G	Ts	n	D>G	0,18-16,3
SP029	001CR	CR	SNP	110	T	82,4-99,9	C	Ts	n	F>L	0,09-17,6
SP030	001CR	CR	SNP	121	A	82,1-100	G	Ts	o		0,03-17,9
SP031	001DF	CR	SNP	4	G	81,9-100	T	Tv	o	E>D	0-18,1
SP032	001DF	MS	indel/MS	93-117	(T5A)3 TAATTTA	38,4-74,5	(T5A)3 TAATTTA	-	-		20,8-49,4
							or (T5A)2 (TTCTTA) TAATTTA	-	-		0,06-16,7
							or (T5A)2	-	-		0-22
							or (T5A) (TTCTTA) (T5A)2 TAATTTA	-	-		0,01-7,6
							or (TCTTTA) (T5A)3 TAATTTA	-	-		0,07-2,5
SP033	001DF	MS	SNP	131	C	97,6-100	T	Ts	-		0-2,4
SP034	001EF	MS	indel/MS	39-40	-	38,2-98,5	AATA	-	-		1,5-61,8
SP035	001EF	MS	SNP	79	T	35,1-97,8	G	Tv	-		2,3-64,9
SP036	001EF	MS	indel	98	A	34,9-97,7	-	-	-		2,3-65,1
SP037	001EF	MS	SNP	117	A	96,8-100	T	Tv	-		0-3,3
SP038	001EF	MS	SNP	124	G	84,7-100	A	Ts	-		0-15,3
SP039	001EF	MS	indel	143	C	96,7-99,8	-	-	-		0,19-3,3
SP040	001ER	NC	SNP	76	C	98-99,9	T	Ts	-		0,06-2
SP041	001ER	NC	indel	109	T	83,4-100	-	-	-		0-16,6
SP042	002AF	CR	2NP	8-9	CC	66,2-85	TT	Ts	n	P>L	15-33,8
SP043	002AF	CR	SNP	10	A	66,7-100	G	Ts	o		0-33,3
SP044	002AF	CR	SNP	20	A	84,5-100	G	Ts	n	I>V	0-15,5
SP045	002AF	CR	SNP	37	A	53,4-94,4	G	Ts	o		5,6-46,6
SP046	002AF	CR	SNP	119	A	29-71,5	T	Tv	n	Y>N	28,5-71
SP047	002AR	CR	SNP	57	T	85,6-99,8	C	Ts	o		0,21-14,4
SP048	002AR	CR	SNP	66	G	97,6-100	T	Tv	n	R>S	0,01-2,4
SP049	003AF	CR	SNP	29	A	96,5-99,9	G	Ts	n	E>G	0,11-3,5
SP050	003AF	CR	SNP	49	C	74,2-100	T	Ts	n	Q>stop	0,05-25,8
SP051	003AF	CR	SNP	52	G	93,8-100	A	Ts	n	D>N	0,05-6,2
SP052	003AF	CR	SNP	64	C	77,4-100	T	Ts	n	L>F	0,03-22,7
SP053	003AF	NC	indel	79	A	91,1-93,9	-	-	-		4,7-6,8
							or AA	-	-		0,9-2,1
SP054	003AR	MS	SNP	17	T	91,5-100	C	Ts	-		0-8,5
SP055	003AR	MS	SNP	37	A	97-100	G	Ts	-		0-3
SP056	003AR	MS	indel	59-60	AAG	40,6-73,2	AAA	Ts	-		22,4-59,4
							or AAAG	-	-		0-30,3
SP057	003AR	MS	SNP	71	T	97,8-100	C	Ts	-		0-2,2
SP058	003AR	MS	SNP	89	T	97,8-100	C	Ts	-		0-2,2
SP059	003AR	MS	SNP	95	T	98,2-100	C	Ts	-		0,03-1,8
SP060	003AR	MS	SNP	96	T	96,7-100	C	Ts	-		0,01-3,3
SP061	003AR	MS	SNP	106	G	91,5-100	A	Ts	-		0-8,5
SP062	003AR	MS	SNP	123	T	97,5-100	C	Ts	-		0-2,5
SP063	003AR	MS	indel/MS	124-125	TTTA	33,8-81	-	-	-		19-66,3
SP064	003AR	MS	SNP	142	T	68,4-100	C	Ts	-		0,04-31,7
SP065	003AR	MS	SNP	145	T	39,2-79,7	C	Ts	-		20,3-60,8

SP #	Marker name	Marker type	SP type	Position (hap1)	Dominant character sequence	min-max %	Modified character's sequence	Ts/Tv	Syn	nSyn	% min-max
SP066	003BF	NC	SNP	1	A	61,1-89,7	T	Tv	-		10,3-38,9
SP067	003BF	NC	SNP	21	A	98,2-100	G	Ts	-		0-1,8
SP068	003BF	NC	indel	26-27	-	4,9-88,9	ACATTAAAGATGAAT	-	-		11,1-63,2
							or AAATTAAAGATGAAT	-	-		0-57,1
SP069	003BF	NC	SNP	45	G	97,1-100	A	Ts	-		0-2,9
SP070	003BF	NC	SNP	57	G	42,9-100	A	Ts	-		0-57,1
SP071	003BF	NC	SNP	118	A	42,9-100	G	Ts	-		0-57,1
SP072	003BF	NC	indel	122	T	42,9-100	-	-	-		0-57,1
SP073	003BF	NC	SNP	129	T	42,9-100	G	Tv	-		0-57,1
SP074	003BF	NC	SNP	131	G	64,9-98,2	T	Tv	-		1,8-35,1
SP075	003BR	NC	SNP	50	T	6,1-90,1	G	Tv	-		9,9-93,9
SP076	003BR	NC	indel	77-78	-	30,7-90,1	TAACTTTGTTATTTTTT	-	-		9,8-67,6
							or AACTTTATTATTTTTT	-	-		0-1,7
SP077	003BR	NC	indel	116-117	-	38,6-99,7	A	-	-		0,3-61,4
SP078	003BR	NC	SNP	123	G	38,6-99,7	A	Ts	-		0,3-61,4
SP079	003BR	NC	2SP	132-133	TC	38,9-99,7	AG	Tv	-		0,3-61,1
SP080	004AF	NC	SNP	1	C	39,6-93,6	T	Ts	-		6,5-60,4
SP081	004AF	NC	SNP	13	T	42,9-77,9	A	Tv	-		22,1-57,1
SP082	004AF	NC	SNP	52	C	40,1-93,4	T	Ts	-		6,6-59,9
SP083	004AF	NC	SNP	55	C	40,1-93,4	T	Ts	-		6,6-59,9
SP084	004AF	NC	SNP	62	A	61,2-100	G	Ts	-		0,01-38,8
SP085	004AF	NC	SNP	100	T	46,7-79,3	C	Ts	-		20,7-53,3
SP086	004AR	NC	SNP	3	A	46,3-86,5	G	Ts	-		13,6-53,7
SP087	004AR	NC	SNP	5	C	46,3-86,5	T	Ts	-		13,6-53,7
SP088	004AR	NC	SNP	17	C	49-78,6	T	Ts	-		21,4-51,1
SP089	004BF	MS	SNP	46	G	37,7-85,2	A	Ts	-		14,8-62,3
SP090	004BF	MS	SNP	92	A	94,5-99,9	G	Ts	-		0,11-5,5
SP091	004BF	MS	indel/MS	120-122	TG	60,8-98,6	(AT)4	-	-		0,42-22,5
							or (AT)2	-	-		0,87-19,7
							or (AT)8	-	-		0,01-3,5
							or (AT)7	-	-		0,1-4,1
SP092	004BR	NC	SNP	7	C	60,7-98	T	Ts	-		2-39,3
SP093	004BR	NC	2NP	15-16	GC	61,7-99	TT	-	-		1-38,3
SP094	004BR	NC	indel	22-23	GA	61,7-99	-	-	-		1-38,3
SP095	004BR	NC	SNP	27	C	61,7-99	T	Ts	-		1-38,3
SP096	004BR	NC	SNP	39	T	61,7-99	C	Ts	-		1-38,3
SP097	004BR	NC	SNP	46	A	61,7-99	G	Ts	-		1-38,3
SP098	004BR	NC	SNP	48	G	61,7-99	T	Tv	-		1-38,3
SP099	004BR	NC	3NP	53-55	AGA	61,7-99	TAG	-	-		1-38,3
SP100	004BR	NC	SNP	59	G	61,7-99	A	Ts	-		1-38,3
SP101	004BR	NC	indel	77-78	-	76,2-99,4	C	-	-		0,62-23,8
SP102	004BR	NC	SNP	90	G	76,2-99,4	T	Tv	-		0,62-23,8
SP103	004BR	NC	SNP	100	G	96,2-100	A	Ts	-		0-3,8
SP104	004BR	NC	2NP	115-116	AA	83,6-99,9	TT	Tv	-		0,14-16,5
SP105	004BR	NC	SNP	122	A	75,6-97,8	G	Ts	-		2,2-24,4
SP106	005AF	CR	SNP	15	C	98,2-100	T	Ts	n	A>V	0,02-1,8
SP107	005AF	CR	SNP	101	G	98,2-99,9	A	Ts	n	G>S	0,09-1,8
SP108	005AF	CR	SNP	126	G	97-99,9	A	Ts	n	S>N	0,06-3
SP109	005AF	CR	SNP	134	C	98,2-100	T	Ts	n	P>S	0,02-1,8
SP110	005AR	CR	SNP	33	T	95,6-99,9	C	Ts	s		0,12-4,5
SP111	005AR	CR	SNP	55	G	76,5-100	A	Ts	n	R>K	0,03-23,5
SP112	005AR	CR	SNP	90	G	93,2-100	A	Ts	n	G>S	0,04-6,8
SP113	005AR	CR	SNP	119	T	93,9-99,9	C	Ts	s		0,12-6,1
SP114	005AR	CR	SNP	122	C	97,9-100	T	Ts	s		0,03-2,1
SP115	005BF	CR	SNP	40	G	81,2-100	A	Ts	n	M>I	0,05-18,8
SP116	005BF	CR	SNP	90	A	66,6-100	G	Ts	n	Q>R	0-33,5
SP117	005BF	CR	SNP	104	G	93,7-100	A	Ts	n	E>K	0-6,3
SP118	005BF	CR	SNP	106	G	95,3-100	A	Ts	s		0,04-4,7
SP119	005BF	CR	SNP	111	G	79,6-100	A	Ts	n	G>E	0,04-20,5
SP120	005BF	CR	SNP	118	C	66,6-100	T	Ts	s		0-33,5
SP121	005BF	CR	SNP	149	A	98,1-99,9	G	Ts	n	R>G	0,15-1,9
SP122	005BR	CR	SNP	26	T	97,8-99,8	C	Ts	s		0,17-2,2
SP123	005BR	CR	SNP	48	G	98,2-99,9	A	Ts	n	G>R	0,07-1,8
SP124	005BR	CR	SNP	120	C	95,4-99,9	T	Ts	n	H>Y	0,09-4,6
SP125	006AF	NC	SNP	23	C	6,9-94,7	T	Ts	-		5,3-93,1
SP126	006AF	NC	SNP	42	T	6,9-94,6	C	Ts	-		5,4-93,1
SP127	006AF	NC	2NP	46-47	GG	53,4-97,8	AA	Ts	-		2,2-46,6
SP128	006AF	NC	SNP	63	C	94,4-100	T	Ts	-		0-5,6
SP129	006AF	NC	SNP	74	C	9,1-100	T	Ts	-		0-90,9
SP130	006AF	NC	SNP	85	C	60,9-98,2	A	Tv	-		1,8-39,1
SP131	006AF	NC	SNP	101	C	51-100	T	Ts	-		0-49
SP132	006AF	NC	SNP	104	T	53,6-97,8	A	Tv	-		2,2-46,4
SP133	006AF	NC	SNP	113	T	6,9-94,2	C	Ts	-		5,9-93,1
SP134	006AF	NC	SNP	133	C	97,6-100	T	Ts	-		0-2,4
SP135	006AR	NC	SNP	6	A	57,7-97,2	C	Tv	-		2,8-42,3
SP136	006AR	NC	SNP	18	G	7,4-100	A	Ts	-		0-92,6
SP137	006AR	NC	SNP	34	T	7,4-100	A	Tv	-		0-92,6

SP #	Marker name	Marker type	SP type	Position (hap1)	Dominant character sequence	min-max %	Modified character's sequence	Ts/Tv	Syn	nSyn	% min-max
SP138	006AR	NC	SNP	43	C	33,1-97,4	T	Ts	-		2,7-66,9
SP139	006AR	NC	SNP	48	G	97,5-100	A	Ts	-		0-2,6
SP140	006AR	NC	indel	58-69	AAAAAT	4,7-70,8	AAATTT	-	-		0-92,6
							or AAAAATAAATTT	-	-		2,7-66,9
SP141	006AR	NC	SNP	74	G	5,5-88,1	T	Tv	-		11,9-94,5
SP142	006AR	NC	SNP	106	C	55,3-98,9	T	Ts	-		1,1-44,7
SP143	006AR	NC	SNP	110	T	49,8-100	C	Ts	-		0-50,2
SP144	006AR	NC	SNP	113	T	55,3-98,9	C	Ts	-		1,1-44,7
SP145	006AR	NC	SNP	134	A	56-99,1	C	Tv	-		0,88-44
SP146	006AR	NC	SNP	137	T	7,4-100	C	Ts	-		0-92,6
SP147	009AF	CR	SNP	8	T	4,7-100	G	Tv	s		0-95,3
SP148	009AF	CR	SNP	39	A	47,6-100	G	Ts	s		0-52,4
SP149	009AF	CR	SNP	53	C	47,6-100	T	Ts	n	V>I	0-52,4
SP150	009AF	CR	SNP	73	C	4,7-100	T	Ts	n	R>K	0-95,3
SP151	009AF	CR	SNP	80	A	75,4-100	G	Ts	n	S>P	0-24,6
SP152	009AF	CR	SNP	85	C	4,5-100	T	Ts	n	R>H	0-95,5
SP153	009AF	CR	SNP	93	G	3,4-69,6	A	Ts	s		30,4-96,6
SP154	009AF	CR	SNP	107	A	75,4-100	G	Ts	s		0-24,6
SP155	009AF	CR	SNP	117	C	48,2-100	A	Tv	n	M>I	0,01-51,8
SP156	009AF	CR	SNP	138	T	3,6-80,4	A	Tv	s		0-72
							or C	Ts	s		19,6-49,4
SP157	009AF	CR	SNP	141	C	76,7-100	A	Tv	s		0-23,3
SP158	009AR	CR	SNP	3	A	50,5-98,9	G	Ts	n	V>A	1,1-49,5
SP159	009AR	CR	3NP	53-55	GCA	4,1-100	TCG	-	n	C>Y	0-95,2
							or GTA	-	n	C>R	0,04-33,9
SP160	011AF	NC	SNP	67	A	49,9-85,9	T	Tv	-		14,1-50,1
SP161	011AF	NC	indel	95	A	54,4-78,6	AA	-	-		11,1-40,7
							or -	-	-		4,8-10,3
SP162	011AF	NC	SNP	130	G	97,4-100	A	Ts	-		0,03-2,6
SP163	011AR	NC	SNP	5	C	60,4-98,3	A	Tv	-		1,7-39,6
SP164	011AR	NC	4NP	36-39	CCCC	14,5-73,2	TTTT	-	-		14,2-71,2
							or TTTC	-	-		1,8-39,7
SP165	011AR	NC	SNP	46	G	26,8-85,5	A	Ts	-		14,5-73,2
SP166	011AR	NC	SNP	63	G	98-100	A	Ts	-		0-2
SP167	011AR	NC	indel	95-96	AAGCATTTTTTTAT GTCGGG	25,4-85,9	-	-	-		14,1-74,6
SP168	011AR	NC	SNP	102	G	25,4-85,9	T	Tv	-		14,1-74,6
SP169	011AR	NC	SNP	114	A	87,5-100	-	-	-		0-12,6
SP170	011AR	NC	SNP	122	A	25,4-85,9	T	Tv	-		14,1-74,6
SP171	011BF	NC	SNP	9	G	96,4-100	A	Ts	-		0-3,6
SP172	011BF	NC	SNP	18	G	87,3-100	T	Tv	-		0-12,7
SP173	011BF	NC	indel	34-42	TATGATAT	27,3-70,9	ATATGATG	-	-		10,2-71,6
							or ATATG	-			0,47-26,2
SP174	011BF	NC	SNP	62	T	27,3-70,9	C	Ts	-		29,1-72,7
SP175	011BF	NC	SNP	92	A	28,1-70,8	G	Ts	-		29,2-71,9
SP176	011BF	NC	SNP	97	G	28,1-70,8	T	Tv	-		29,2-71,9
SP177	011BF	NC	SNP	127	G	98,1-100	A	Ts	-		0-1,9
SP178	011BR	NC	SNP	31	T	28,9-76,5	C	Ts	-		23,6-71,1
SP179	011BR	NC	SNP	48	C	97,6-100	T	Ts	-		0-2,4
SP180	011BR	NC	SNP	78	A	43,4-98,8	C	Tv	-		1,2-56,6
SP181	011BR	NC	SNP	85	A	43,4-98,8	T	Tv	-		1,2-56,6
SP182	011BR	NC	SNP	91	A	45-96	G	Ts	-		4-55
SP183	011BR	NC	SNP	126	G	43,4-98,9	A	Ts	-		1,1-56,6
SP184	011BR	NC	SNP	139	C	43,3-98,9	T	Ts	-		1,1-56,7
SP185	013AF	CR	SNP	103	G	85,2-100	A	Ts	s		0,03-14,8
SP186	013AF	CR	SNP	125	G	71,2-100	A	Ts	n	D>N	0,03-28,8
SP187	013AF	CR	SNP	131	G	97,3-100	A	Ts	n	D>N	0,03-2,7
SP188	013AF	CR	SNP	146	G	92,6-99,9	A	Ts	n	G>R	0,07-7,5
SP189	013AR	CR	indel	29-46	GATGATGAGGATG CATTT	83,8-99,9	-	-	n	Δ 6 AA	0,03-16,1
							or GATGATGAAGATGCATTT	-	s	ORF	0,03-2,6
SP190	013AR	CR	SNP	55	G	30,5-97,9	A	Ts	s		2,1-69,5
SP191	013AR	CR	SNP	63	T	30,5-97,9	G	Tv	n	F>C	2,1-69,5
SP192	013AR	CR	indel	79-80	-	30,5-97,9	ACTGATGATAAAAATACA	-	n	Δ 6 AA	2,1-69,5
SP193	013AR	CR	SNP	86	G	30,5-97,9	A	Ts	n	D>N	2,1-69,5
SP194	013AR	CR	SNP	112	G	68,1-100	T	Tv	s		0-29
							or A	Ts	s		0-3
SP195	013BF	NC	SNP	9	T	71,6-99,8	C	Ts	-		0,21-28,4
SP196	013BF	NC	SNP	22	C	19,3-100	T	Ts	-		0-80,7
SP197	013BF	NC	SNP	33	T	47,6-100	C	Ts	-		0-52,4
SP198	013BF	NC	SNP	35	A	71,7-100	G	Ts	-		0-28,3
SP199	013BF	NC	SNP	49	C	39-100	T	Ts	-		0,01-61
SP200	013BF	NC	SNP	67	G	71,7-100	A	Ts	-		0-28,3
SP201	013BF	NC	3NP	82-84	GAT	19,3-100	TTC	-	-		0-80,7
SP202	013BF	NC	2NP	101-102	AA	19,2-99,9	AG	Ts	-		0-80,7
							or GG	Ts	-		0,12-27,8
SP203	013BF	NC	SNP	105	C	71,7-100	T	Ts	-		0-28,3

SP #	Marker name	Marker type	SP type	Position (hap1)	Dominant character sequence	min-max %	Modified character's sequence	Ts/Tv	Syn	nSyn	% min-max
SP204	013BF	NC	2NP	129-130	GC	19,3-100	GA	Ts	-		0-52,4
							or AC	Ts			0-28,3
SP205	013BR	NC	SNP	7	A	18-100	T	Tv	-		0-82
SP206	013BR	NC	SNP	31	C	18-100	T	Ts	-		0-82
SP207	013BR	NC	SNP	55	G	46,6-100	A	Ts	-		0-53,4
SP208	013BR	NC	SNP	78	G	46,6-100	A	Ts	-		0-53,4
SP209	013BR	NC	SNP	97	G	18-100	A	Ts	-		0-82
SP210	013BR	NC	indel	142	T	39,2-94,1	-	-	-		5,9-60,8
SP211	013BR	NC	SNP	148	G	8,2-94,1	A	Ts	-		5,9-91,8
SP212	014AF	CR	SNP	75	T	62-100	A	Tv	n	L>H	0,03-38,1
SP213	014AR	NC	SNP	22	C	31,2-89,4	T	Ts	-		10,6-68,9
SP214	014AR	NC	SNP	36	C	85,8-100	T	Ts	-		0,01-14,2
SP215	014AR	NC	indel	107	T	97,1-98,3	-	-	-		1,7-2,9
SP216	016AF	CR	SNP	10	G	97,8-100	A	Ts	n	S>N	0-2,2
SP217	016AF	CR	SNP	12	C	94,8-100	T	Ts	n	Q>stop	0-5,2
SP218	016AF	CR	SNP	25	A	25,2-98,3	G	Ts	n	K>R	1,7-74,8
SP219	016AF	CR	SNP	50	A	25,3-98,2	G	Ts	s		1,8-74,7
SP220	016AF	CR	SNP	66	G	98,2-100	A	Ts	n	E>K	0,02-1,9
SP221	016AF	CR	SNP	106	C	97,1-100	T	Ts	n	S>L	0-2,9
SP222	016AF	CR	SNP	133	A	98-100	G	Ts	n	N>S	0-2
SP223	016AR	NC	indel	38	G	23,7-98,2	A	-	-		1,8-75,7
							or -				0,01-2,6
SP224	016AR	NC	indel	50-53	ATAA	23,7-98,2	-	-	-		1,8-76,3
SP225	016AR	NC	SNP	83	T	23,6-97,8	C	Ts	-		2,2-76,4
SP226	016AR	NC	SNP	121	G	96,8-100	A	Ts	-		0-3,2
SP227	016BF	CR	SNP	16	G	94,3-100	A	Ts	n	G>R	0,01-5,7
SP228	016BF	CR	SNP	42	C	45,1-76,2	T	Ts	s		23,8-55
SP229	016BR	CR	SNP	58	C	97,6-100	T	Ts	n	P>S	0,02-2,4
SP230	016BR	CR	SNP	115	G	97,2-100	A	Ts	n	E>K	0,03-2,8
SP231	016BR	CR	SNP	120	T	97,8-99,9	C	Ts	s		0,09-2,2
SP232	016BR	CR	SNP	126	T	47,9-94,5	C	Ts	s		5,5-52,1
SP233	017AF	NC	SNP	3	T	91,2-100	C	Ts	-		0-8,8
SP234	017AF	NC	3NP	29-31	ATG	63,1-100	GCA	-	-		0-36,9
SP235	017AF	NC	2NP	34-35	TT	41,3-88	TC	Ts	-		12-44,6
							or CC	Ts	-		0-45,7
SP236	017AF	NC	indel	42	A	42,4-79,4	-	-	-		20,6-57,6
SP237	017AF	NC	indel	48-49	AA	78,1-100	G	-	-		0-21,9
SP238	017AF	NC	SNP	55	A	91,2-100	G	Ts	-		0-8,8
SP239	017AF	NC	SNP	58	G	43,2-88,1	A	Ts	-		11,9-56,8
SP240	017AF	NC	indel	83-84	AATATTA	39-80,3	TTA	-	-		19,7-61
							or AGTATAT	-	-		0-30,7
SP241	017AF	NC	indel	138	AG	38,3-77,8	A	-	-		20,6-60,9
							or AAG	-	-		0-14,3
							or GG	-	-		0,37-1,7
SP242	017AR	NC	SNP	25	T	54,5-100	A	Tv	-		0-45,5
SP243	017AR	NC	2SP	50-51	GC	70,8-100	AC	Ts	-		0-29,2
							or GT	Ts	-		0,01-2,1
SP244	017AR	NC	SNP	55	A	64,3-100	G	Ts	-		0-35,7
SP245	017AR	NC	SNP	65	A	79,5-100	G	Ts	-		0-20,5
SP246	017AR	NC	SNP	67	G	55,6-100	T	Tv	-		0-44,4
SP247	017AR	NC	SNP	82	C	84,8-100	T	Ts	-		0-15,2
SP248	017AR	NC	2SP	84-85	GT	43,4-87,1	GC	Ts	-		8,4-56,6
							or AT	Ts	-		0-5,2
SP249	017AR	NC	indel	110	T	39,5-79,7	-	-	-		20,4-60,5
SP250	017AR	NC	SNP	115	A	84,8-100	G	Ts	-		0-15,2
SP251	017AR	NC	SNP	120	T	44,8-83,9	G	Tv	-		16,1-55,2
SP252	017AR	NC	SNP	123	G	70,8-100	A	Ts	-		0-29,2
SP253	019AF	CR	SNP	13	A	26,8-80,1	G	Ts	s		19,9-73,2
SP254	019AF	CR	SNP	67	G	92,3-100	A	Ts	s		0-7,7
SP255	019AF	CR	SNP	72	A	97,9-100	G	Ts	n	D>G	0-2,1
SP256	019AF	CR	SNP	76	A	17,4-74,4	G	Ts	s		25,6-82,6
SP257	019AF	CR	SNP	92	G	96,6-100	A	Ts	n	D>N	0,01-3,4
SP258	019AF	CR	indel	129-130	-	17,2-74,1	AGA	-	n	del E	25,9-82,8
SP259	019AR	CR	SNP	11	G	80,3-100	C	Tv	n	V>L	0,01-19,7
SP260	019AR	CR	SNP	14	A	97,9-99,9	G	Ts	n	I>V	0,11-2,1
SP261	019AR	CR	SNP	95	A	98,1-100	G	Ts	n	K>E	0-1,9
SP262	019AR	CR	SNP	128	G	98-100	A	Ts	n	V>I	0,04-2
SP263	019BF	CR	SNP	30	C	97,8-100	T	Ts	n	P>S	0-2,2
SP264	019BF	CR	SNP	63	A	68,8-99,9	G	Ts	n	T>A	0,07-31,2
SP265	019BF	CR	indel	89-90	-	56,9-79,1	ATGCCAGGTGGAAATTATCC AGGAAGCAAT	-	n	Δ 10 aa	20,9-43,1
SP266	019BR	CR	SNP	58	T	24,2-70	C	Ts	s		30-75,8
SP267	019BR	CR	SNP	73	G	47,7-73,5	A	Ts	s		26,5-52,3
SP268	019BR	CR	SNP	113	C	96-100	T	Ts	n	Q>stop	0-4
SP269	019CF	CR	SNP	20	G	91,4-100	A	Ts	n	R>K	0-8,6
SP270	019CF	CR	SNP	38	C	98,2-100	T	Ts	n	S>L	0-1,8

SP	Marker	Marker	SP	Position	Dominant character		Modified character's				
#	name	type	type	(hap1)	sequence	min-max %	sequence	Ts/Tv	Syn	nSyn	% min-max
SP271	019CF	CR	SNP	86	C	93,2-100	T	Ts	n	P>L	0-6,8
SP272	019CF	CR	SNP	96	A	45,9-96,3	T	Tv	s		3,7-54,1
SP273	019CF	CR	SNP	120	G	12,5-100	T	Tv	s		0-84,6
							or A	Ts	s		0,03-55,3
SP274	019CF	CR	SNP	126	C	5-59,5	T	Ts	s		40,5-95,1
SP275	019CF	CR	SNP	130	G	96,7-100	A	Ts	n	D>N	0-3,3
SP276	019CF	CR	SNP	133	G	96,8-100	A	Ts	n	G>S	0,01-3,2
SP277	019CF	CR	SNP	144	C	15,4-100	T	Ts	s		0-84,6
SP278	019CR	CR	SNP	44	G	98,2-100	A	Ts	n	V>I	0-1,8
SP279	019CR	CR	SNP	73	T	18,6-100	C	Ts	s		0,01-81,4
SP280	019CR	CR	SNP	78	A	98,2-100	G	Ts	n	E>G	0,03-1,8
SP281	019CR	CR	SNP	99	C	96,7-100	T	Ts	n	S>L	0-3,3
SP282	019CR	CR	SNP	110	A	13,4-85,6	G	Ts	n	I>V	14,4-86,7
SP283	019CR	CR	SNP	143	G	91,3-100	A	Ts	n	G>R	0-8,7
SP284	020AF	CR	2NP	15-16	CG	34,8-96,2	TG	Ts	n	S>L	3,8-65,2
							or CA	Ts	s		0-2,7
SP285	020AF	CR	SNP	35	C	94,8-100	T	Ts	n	M>V	0-5,2
SP286	020AF	CR	SNP	38	C	48,6-100	T	Ts	n	M>V	0-51,4
SP287	020AF	CR	SNP	61	T	37-96,1	C	Ts	n	Y>C	3,9-63
SP288	020AF	CR	SNP	63	C	95-100	T	Ts	n	M>I	0-5
SP289	020AF	CR	SNP	106	T	24,7-96	C	Ts	n	E>G	4-75,3
SP290	020AF	CR	SNP	110	C	93,6-100	A	Tv	n	V>F	0-6,4
SP291	020AR	NC	indel	48-49	-	44,8-96,7	A	-	-		3,3-55,3
SP292	020AR	NC	indel	85-86	-	40,8-88,8	GA	-	-		11,2-59,2
SP293	020AR	NC	SNP	90	A	40,8-88,8	G	Ts	-		11,2-59,2
SP294	020AR	NC	SNP	98	T	43,8-96	C	Ts	-		4,1-56,2
SP295	020AR	NC	2NP	103-104	GA	40,8-88,8	GG	Ts	-		11,2-59,1
							or TG	Tv			0-3
SP296	020AR	NC	indel	111	-	40,8-88,8	C	-	-		11,2-59,2
SP297	020AR	NC	2NP	114-115	CA	11,2-59,2	CG	Ts	-		6,7-60,8
							or TG	Ts	-		3,5-58,5
SP298	020AR	NC	SNP	117	T	40,8-88,8	C	Ts	-		11,2-59,2
SP299	020AR	NC	SNP	123	A	96,5-100	G	Ts	-		0,01-3,5
SP300	021AF	CR	SNP	10	A	80,1-100	T	Tv	n	E>V	0-19,9
SP301	021AF	CR	SNP	17	C	18,7-96,8	T	Ts	s		3,2-81,3
SP302	021AF	CR	SNP	35	G	26,3-88,4	A	Ts	s		11,6-73,7
SP303	021AF	CR	SNP	63	A	21,9-98,7	G	Ts	n	I>V	1,3-78,1
SP304	021AF	CR	SNP	68	A	68,1-100	G	Ts	s		0-31,9
SP305	021AF	CR	SNP	77	T	21,9-98,7	C	Ts	s		1,3-78,1
SP306	021AF	CR	SNP	80	G	97,1-100	A	Ts	s		0-2,9
SP307	021AF	CR	SNP	91	C	97,4-100	T	Ts	n	S>L	0-2,6
SP308	021AF	CR	SNP	118	A	48,2-100	G	Ts	n	K>R	0-51,8
SP309	021AF	CR	SNP	124	A	68,1-100	G	Ts	n	N>S	0-31,9
SP310	021AF	CR	SNP	144	T	43,8-84,8	C	Ts	s		15,2-56,2
SP311	021AR	CR	SNP	17	C	24,2-100	T	Ts	s		0-75,8
SP312	021AR	CR	SNP	54	A	97,9-100	G	Ts	n	K>E	0-2,1
SP313	021AR	CR	SNP	66	T	53,1-99	C	Ts	n	S>P	1-46,9
SP314	021AR	CR	SNP	74	G	16,4-95,1	A	Ts	s		5-83,6
SP315	021AR	CR	SNP	100	G	11,3-74,4	A	Ts	n	G>E	25,6-88,7
SP316	021AR	CR	SNP	135	G	88,8-100	A	Ts	n	E>K	0,01-11,2
SP317	021BF	CR	SNP	6	T	86,4-100	G	Tv	n	L>V	0,05-13,6
SP318	021BF	CR	SNP	54	G	97,7-100	A	Ts	n	D>N	0-2,3
SP319	021BF	CR	SNP	86	G	93,4-100	A	Ts	s		0,02-6,6
SP320	021BF	CR	SNP	104	A	98,2-100	G	Ts	s		0-1,8
SP321	021BF	CR	SNP	149	G	18,5-75,9	A	Ts	s		24,1-81,5
SP322	021BR	CR	2NP	4-5	CG	96,3-100	TG	Ts	s		0-2
							or CA	Ts	n	V>I	0-1,7
SP323	021BR	CR	SNP	49	A	14,7-75,9	G	Ts	s		24,1-85,3
SP324	021BR	CR	SNP	147	C	97,8-100	T	Ts	n	S>L	0,01-2,2
SP325	025AF	CR	SNP	15	A	85,8-100	G	Ts	s		0-14,3
SP326	025AF	CR	SNP	32	G	96,2-100	A	Ts	n	R>K	0-3,8
SP327	025AF	CR	SNP	54	C	57,5-95,4	T	Ts	s		4,6-42,5
SP328	025AF	CR	SNP	78	C	49,9-80	T	Ts	s		20-50,1
SP329	025AF	CR	SNP	106	G	96,2-100	A	Ts	n	E>K	0-3,8
SP330	025AR	CR	SNP	28	C	79,4-99,9	T	Ts	n	A>V	0,12-20,7
SP331	025AR	CR	SNP	38	A	82,3-99,9	G	Ts	s		0,1-17,7
SP332	025AR	CR	SNP	140	G	67,4-100	A	Ts	s		0-32,6
SP333	034AF	NC	SNP	80	T	8,3-100	C	Ts			0,03-91,7
SP334	034AF	NC	indel	133-134	A	5,7-79,1	TAT	-			20,9-94,3
SP335	034AR	CR	SNP	23	T	6,8-76,2	C	Ts	s		23,9-93,2
SP336	034AR	CR	SNP	41	G	10,3-100	A	Ts	s		0-89,7
SP337	034AR	CR	SNP	71	G	8,5-95,8	A	Ts	s		4,2-91,6
SP338	034AR	CR	SNP	80	T	83,6-100	C	Ts	s		0,01-16,4
SP339	034AR	CR	2NP	105-106	TC	53,8-100	CT	Ts	n	S>L	0-46,2
SP340	034AR	CR	SNP	114	T	86,2-100	C	Ts	n	S>P	0-13,9
SP341	034AR	CR	SNP	149	T	86,2-100	A	Tv	s		0-13,9
SP342	036AF	CR	SNP	4	A	96,7-100	G	Ts	s		0-3,3

SP	Marker	Marker	SP	Position	Dominant character		Modified character's				
#	name	type	type	(hap1)	sequence	min-max %	sequence	Ts/Tv	Syn	nSyn	% min-max
SP343	036AF	CR	SNP	63	C	50,8-85,2	T	Ts	s		14,8-49,2
SP344	036AF	CR	SNP	68	G	50,8-85,2	A	Ts	s		14,8-49,2
SP345	036AR	CR	SNP	78	G	97,5-100	A	Ts	n	D>N	0-2,5
SP346	036AR	CR	SNP	103	G	97,5-100	A	Ts	n	C>Y	0,02-2,5
SP347	036AR	CR	SNP	110	T	41,8-83,8	C	Ts	s		16,3-58,2
SP348	041AF	CR	SNP	115	C	34,5-78,1	T	Ts	n	L>F	21,9-65,5
SP349	041AF	CR	SNP	129	T	98,1-100	C	Ts	s		0,04-1,9
SP350	041AF	CR	SNP	147	A	34,5-78,5	G	Ts	s		21,5-65,5
SP351	041AR	CR	SNP	30	G	34,4-82,6	A	Ts	n	E>K	17,4-65,6
SP352	041AR	CR	SNP	46	C	93,8-100	T	Ts	n	A>V	0-6,2
SP353	041AR	CR	SNP	63	G	34,3-82,7	A	Ts	n	V>I	17,3-65,7
SP354	041AR	CR	2NP	65-66	TC	34,3-82,7	CC	Ts	s		17,3-65,7
							TT	Ts	n	P>S	0-17,2
SP355	041AR	CR	SNP	78	G	97,6-100	A	Ts	n	A>T	0,02-2,4
SP356	041AR	CR	SNP	81	C	34-82,2	A	Tv	n	L>I	17,8-66,1
SP357	043AF	MS	indel/MS	13-14	TAAATAAA	30,6-85,9	-	-	-		8,4-56,9
							or TAAA	-	-		1,3-42,1
SP358	043AF	MS	2NP	34-35	TG	36,7-87,5	CG	Ts	-		1-42,4
							or TA	Ts	-		6,8-34
SP359	043AF	MS	SNP	46	C	97,6-100	T	Ts	-		0-2,4
SP360	043AF	MS	SNP	49	T	55,2-99	C	Ts	-		1-44,9
SP361	043AF	MS	2NP	63-64	GG	57,6-99	AA	Ts	-		1-42,4
SP362	043AF	MS	SNP	101	A	70,7-100	C	Tv	-		0-29,3
SP363	043AF	MS	SNP	105	C	60,8-100	A	Tv	-		0-39,2
SP364	043AF	MS	SNP	110	G	97,2-100	A	Ts	-		0-2,9
SP365	043AF	MS	2NP	123-124	GA	18,5-85,3	AG	-	-		14,7-81,5
SP366	043AR	NC	SNP	44	A	18-97,5	G	Ts	-		2,6-82
SP367	043AR	NC	indel	51	G	81,2-100	-	-	-		0-18,8
SP368	043AR	NC	SNP	56	T	81,2-100	C	Ts	-		0-18,8
SP369	043AR	NC	SNP	58	G	22,9-100	A	Ts	-		0-77,1
SP370	043AR	NC	SNP	66	G	97,6-100	A	Ts	-		0-2,5
SP371	043AR	NC	SNP	119	T	18,1-96,9	G	Tv	-		3,1-81,9
SP372	043AR	NC	SNP	144	T	16,7-96,9	A	Tv	-		3,2-83,3
SP373	049AF	CR	SNP	28	G	41,9-62,2	A	Ts	s		37,8-58,1
SP374	049AF	CR	SNP	32	G	94,5-100	A	Ts	n	D>N	0-5,5
SP375	049AF	CR	SNP	60	C	97,3-100	T	Ts	n	T>I	0-2,7
SP376	049AF	CR	SNP	138	A	43,9-62,2	G	Ts	n	R>K	37,8-56,1
SP377	049AR	CR	SNP	4	G	40,9-59,9	A	Ts	s		40,1-59,1
SP378	049AR	CR	SNP	23	G	40,9-60,3	A	Ts	n	E>K	39,8-59,1
SP379	049AR	CR	SNP	28	G	97,1-100	A	Ts	s		0,02-2,9
SP380	049AR	CR	SNP	103	G	95,2-100	A	Ts	s		0-4,8
SP381	049AR	CR	SNP	147	T	91,7-99,9	C	Ts	n	I>T	0,14-8,3
SP382	052AF	MS	SNP	9	C	73,7-95,7	T	Ts	-		4,3-26,3
SP383	052AF	MS	SNP	35	T	83-99,5	C	Ts	-		0,52-15,1
							or A	Tv	-		0-9,6
SP384	052AF	MS	indel	41	C	83-99,5	-	-	-		0,52-17
SP385	052AF	MS	SNP	51	C	79,6-99,5	A	Tv	-		0,52-17
							or T	Ts	-		0-3,4
SP386	052AF	MS	indel/MS	75-90	(GT)1 (AT)6	62,4-88,8	(GT)1 (AT)7	-	-		3,4-16,4
							or (AT)3 (GT)1(AT)5	-	-		0,52-15,1
							or (AT)2 (GT)1(AT)5	-	-		0-9,6
							or GT)1 (AT)5	-	-		2,6-6,2
SP387	052AF	MS	SNP	91	T	90,4-100	C	Ts	-		0-9,6
SP388	052AF	MS	SNP	99	A	52,4-82,9	T	Tv	-		17,1-47,6
SP389	052AF	MS	SNP	132	C	96,6-100	T	Ts	-		0-3,4
SP390	052AR	NC	SNP	12	C	94,8-100	T	Ts	-		0-5,2
SP391	052AR	NC	SNP	22	A	80,1-100	G	Ts	-		0-20
SP392	052AR	NC	SNP	31	A	59,3-88,3	T	Tv	-		11,7-40,7
SP393	052AR	NC	SNP	33	C	96,7-100	T	Ts	-		0-3,3
SP394	052AR	NC	SNP	37	A	94,1-99,7	G	Ts	-		0,33-5,9
SP395	052AR	NC	SNP	40	C	94,1-99,7	T	Ts	-		0,33-5,9
SP396	052AR	NC	SNP	48	C	94,1-99,7	T	Ts	-		0,33-5,9
SP397	052AR	NC	SNP	54	C	94,1-99,7	G	Tv	-		0,33-5,9
SP398	052AR	NC	SNP	58	A	94,1-99,7	G	Ts	-		0,33-5,9
SP399	052AR	NC	SNP	71	T	90-100	G	Tv	-		0-10
SP400	052AR	NC	SNP	104	T	94,8-100	C	Ts	-		0-5,2
SP401	052AR	NC	SNP	113	C	34,8-73,2	G	Tv	-		26,8-65,2
SP402	052AR	NC	SNP	116	C	63,5-90,7	T	Ts	-		2,3-29,5
							or A	Tv	-		0-19,3
SP403	052AR	NC	indel	12-129	TT	40-66,2	-	-	-		33,8-60
SP406	105AF	MS	SNP	50	G	48,8-96,2	A	Ts	-		3,9-51,2
SP407	105AF	MS	indel	93	-	72,9-98,3	AG	-	-		1,7-27,1
SP408	105AF	MS	indel	94	-	13-94,3	(AT)1	-	-		2,5-55
							or (AT)2	-	-		1,8-27
							or (AT)3	-	-		0,73-19,1
							or (AT)4	-	-		0-2,4
SP409	105AF	MS	SNP	126	G	48,9-95,6	A	Ts	-		4,4-51,1

SP	Marker	Marker	SP	Position	Dominant character		Modified character's				
#	name	type	type	(hap1)	sequence	min-max %	sequence	Ts/Tv	Syn	nSyn	% min-max
SP410	105AF	MS	SNP	135	G	48,9-95,6	A	Ts	-		4,4-51,1
SP411	105AR	NC	SNP	5	A	48,8-92,2	G	Ts	-		7,8-51,2
SP412	105AR	NC	SNP	62	A	48,7-92,1	G	Ts	-		7,9-51,4
SP413	105AR	NC	SNP	74	A	41,2-98	G	Ts	-		2-58,8
SP414	105AR	NC	SNP	93	G	88,4-100	A	Ts	-		0-11,6
SP415	105AR	NC	SNP	126	G	40,6-98,1	A	Ts	-		1,9-59,4
SP416	105AR	NC	SNP	130	G	40,6-98,1	A	Ts	-		1,9-59,4
SP417	143AF	MS	indel/MS	23-24	(AT)1	33,2-84,7	(AT)3	-	-		2,2-14,8
							or (AT)2	-	-		1,3-4,7
							or (AT)7	-	-		0,42-6
							or (AT)4-6	-	-		0,76-49,2
							or -	-	-		2,8-6,8
SP418	143AF	MS	SNP	38	G	90,5-100	A	Ts	-		0-9,5
SP419	143AF	MS	SNP	88	T	95,8-100	G	Tv	-		0-4,2
SP420	143AF	MS	SNP	100	A	96,9-100	G	Ts	-		0-3,1
SP421	143AR	NC	SNP	33	A	60,4-100	C	Tv	-		0-39,6
SP422	143AR	NC	SNP	59	A	96,5-100	G	Ts	-		0-3,5
SP423	143AR	NC	indel	63	A	30,5-83	-	-	-		0,82-2,5
							or AA	-	-		15,1-67
							or AAA	-	-		0,29-2,7
SP424	143AR	NC	SNP	74	G	24,1-82,6	A	Ts	-		17,4-75,9
SP425	149AF	NC	SNP	25	C	96,8-100	T	Ts	-		0-3,2
SP426	149AF	NC	SNP	29	A	97,3-99,9	G	Ts	-		0,06-2,7
SP427	149AF	NC	SNP	31	T	37,8-100	A	Tv	-		0-62,2
SP428	149AF	NC	SNP	40	C	89,4-100	T	Ts	-		0-10,6
SP429	149AF	NC	SNP	45	A	93,4-100	C	Tv	-		0-6,6
SP430	149AF	NC	indel	51-52	-	78,9-100	T	-	-		0-21,1
SP431	149AF	NC	SNP	62	A	89,4-100	G	Ts	-		0-10,6
SP432	149AF	NC	SNP	80	C	96,8-100	T	Ts	-		0-3,2
SP433	149AF	NC	2NP	96-97	AA	28,4-96,1	TT	-	-		0-62,2
							or A	-	-		3,5-36,1
SP434	149AF	NC	2NP	100-101	GA	72,3-100	AA	-	-		0-21,1
							or TG	-	-		0-6,6
SP435	149AF	NC	SNP	123	C	69,4-100	T	Ts	-		0-30,6
SP436	149AF	NC	2NP	127-128	GG	57,6-88,3	GA	Ts	-		10-42,4
							or AG	Ts	-		0-4,9
SP437	149AF	NC	2NP	142-143	AT	79,2-97,4	AC	-	-		1,3-14,4
							or T	-	-		0,77-13,1
SP438	149AR	MS	SNP	13	G	82,6-99,7	T	Tv	-		0,32-17,4
SP439	149AR	MS	SNP	18	C	51,3-100	T	Ts	-		0-48,7
SP440	149AR	MS	SNP	20	C	97,6-100	T	Ts	-		0-2,4
SP441	149AR	MS	SNP	33	G	56,3-95,9	A	Ts	-		4,1-43,7
SP442	149AR	MS	SNP	37	T	62-95,2	C	Ts	-		4,8-38
SP443	149AR	MS	SNP	55	C	42,5-100	T	Ts	-		0-57,5
SP444	149AR	MS	indel/MS	68-71	(AT)2	50,6-89,1	(AT)4	-	-		4,7-40
							(AT)3	-	-		0,63-6,1
							(AT)1	-	-		1,5-5,4
							-	-	-		0-25,2
SP445	149AR	MS	SNP		T	84,1-98,9	C	-	-		1,1-15,9
SP446	149AR	MS	SNP	84	C	96,4-100	T	Ts	-		0-3,6
SP447	149AR	MS	2NP	86-87	AC	67,8-100	TT	-	-		0-32,2
SP448	149AR	MS	indel	92	A	67,8-100	-	-	-		0-32,2
SP449	149AR	MS	SNP	120	A	42,5-100	G	Ts	-		0-57,5
SP450	149AR	MS	SNP	124	A	41,9-99,8	G	Ts	-		0,24-58,2
SP451	149AR	MS	SNP	129	C	78,6-99,9	T	Ts	-		0,15-21,4
SP452	149AR	MS	SNP	145	T	80,5-98,7	A	Tv	-		1,3-19,5
SP453	174AF	MS	SNP	1	G	54,2-96,5	T	Tv	-		3,6-45,8
							or A	Ts	-		0-20,6
SP454	174AF	MS	SNP	8	A	97,1-100	G	Ts	-		0-2,9
SP455	174AF	MS	SNP	21	T	95-100	C	Ts	-		0,01-5
SP456	174AF	MS	2NP	29	TC	55,3-88,9	GA	-	-		11,2-44,7
SP457	174AF	MS	indel/MS	130-131	(TA)1	17,5-76,7	(TA)6	-	-		2,9-34
							or (TA)7	-	-		5-25,4
							or (TA)5	-	-		2,6-22,6
							or (TA)8	-	-		1,6-15,5
							or (TA)0,2-4	-	-		1,4-14,5
SP458	174AR	NC	SNP	28	A	48,4-88,3	G	Ts	-		11,7-51,7
SP459	174AR	NC	SNP	48-49	-	50,9-95,2	T	-	-		4,8-49,1
SP460	174AR	NC	SNP	62	T	44,6-91,1	A	Tv	-		8,9-55,4
SP461	174AR	NC	SNP	64	C	46,7-76,8	A	Tv	-		23,2-53,3
SP462	174AR	NC	SNP	126	C	91,2-100	T	Ts	-		0-8,8
SP463	174AR	NC	SNP	143	C	94,2-100	T	Ts	-		0-5,8
SP464	185AF	MS	indel/MS	77-82	(AT)3	14,7-68,4	(AT)4	-	-		6,6-59,7
							or (AT)1	-	-		1,1-51,5
							or (AT)2	-	-		5,6-36,5
							or (AT)5	-	-		1,4-8,3
							or -	-	-		0,15-10,7

SP	Marker	Marker	SP	Position	Dominant character		Modified character's				
#	name	type	type	(hap1)	sequence	min-max %	sequence	Ts/Tv	Syn	nSyn	% min-max
SP465	185AR	NC	SNP	30	C	92,1-100	T	Ts	-		0-7,9
SP466	185AR	NC	SNP	37	C	97,8-100	T	Ts	-		0-2,2
SP467	185AR	NC	SNP	45	G	95,9-100	A	Ts	-		0,05-4,1
SP468	185AR	NC	SNP	57	G	88,6-100	A	Ts	-		0-11,4
SP469	185AR	NC	SNP	93	G	96,6-100	A	Ts	-		0-3,4
SP470	185AR	NC	indel	121	A	89,1-93,3	-	-	-		4,7-7,8
							or AA	-	-		2-3,9
SP471	185AR	NC	SNP	134	G	91,3-100	A	Ts	-		0-8,7
SP472	185AR	NC	SNP	139	G	88,6-100	T	Tv	-		0-11,4
SP473	185AR	NC	SNP	146	G	98,1-100	A	Ts	-		0,03-1,9
SP474	185AR	NC	SNP	150	T	97,2-99,4	C	Ts	-		0,65-2,8
SP475	386AF	CR	SNP	2	G	61,3-95,5	A	Ts	s		4,5-38,7
SP476	386AF	CR	3NP	10-12	CAC	43,6-85,7	TAC	-	n	P>L	0,39-42,6
							or CAT	-	s		0,54-31,7
							or CGC	-	s		0-11,1
SP477	386AF	CR	SNP	18	G	96,7-100	A	Ts	n	D>N	0-3,3
SP478	386AF	CR	SNP	25	A	22,7-95,3	C	Tv	n	K>T	4,8-77,3
SP479	386AF	CR	SNP	48	A	54,8-85,7	G	Ts	n	I>V	14,3-45,3
SP480	386AF	CR	2NP	54-55	CC	33,8-95,3	TC	Ts	n	P>S	3,1-48,7
							or TT	Ts	n	P>F	0,54-31,7
SP481	386AF	CR	SNP	79	G	51-98,3	A	Ts	n	S>N	1,8-49
SP482	386AF	CR	SNP	86	A	57,4-99,6	G	Ts	s		0,41-42,6
SP483	386AF	CR	SNP	95	T	28,3-66,6	C	Ts	s		33,4-71,8
SP484	386AF	CR	SNP	106	A	28,3-66,6	C	Tv	n	N>T	33,4-71,8
SP485	386AF	CR	SNP	141	C	97,9-100	T	Ts	n	stop	0-2,1

Table S6. Types of polymorphic sites in isolates.

Isolate	number of transitions for one transversion all markers	non coding markers	coding markers	number of synonymous for one non synonymous change
Ador	3,70	2,74	5,67	1,29
Acer1.1	3,06	1,84	6,81	1,35
GRE5.6	3,69	2,10	7,03	1,08
CAR2.1	3,51	2,00	8,50	1,44
POR	3,14	1,80	7,42	1,36
RUN0065	3,19	1,86	6,51	1,29
RUN0067	3,58	2,08	8,32	1,49
RUN0109	3,32	1,94	7,59	1,38
RUN0281	3,44	1,88	8,26	1,45
RUN1041	3,31	1,88	7,32	1,35
RUN1050	3,60	2,08	7,40	1,31
RUN1132	3,34	1,85	7,71	1,40
RUN1232	3,22	1,59	9,12	1,24
RUN1578	3,24	1,87	6,92	1,24
MU011I	3,27	1,81	7,93	1,36
MU025I	3,15	1,78	7,23	1,32
MU040I	3,28	1,83	7,70	1,36
ROD001	3,30	1,95	7,68	1,40
ROD200	3,57	2,16	8,53	1,54
COM11	3,30	2,01	7,28	1,40
COM12	3,07	1,84	6,52	1,31
COM18	3,15	1,88	6,24	1,29
COM28	3,29	1,86	7,13	1,29
COM59	3,15	1,87	6,64	1,40
COM72	3,24	2,09	6,62	1,35
MAY35	3,49	2,08	7,31	1,38
SEY059	3,16	1,90	6,75	1,44
SEY141	3,29	2,08	6,98	1,50
SEY144	2,99	1,80	6,63	1,42
ISM03	3,25	1,75	7,42	1,46
ISM04	3,10	1,74	6,47	1,37
MAD26.1	3,66	2,10	7,49	1,22
MAD30.2	2,87	1,69	6,25	1,31
Whole dataset	3,30	1,94	7,16	1,35

Table S7. Tags and libraries. Tags were tag0: GTCA, tag5: CGAT, and tag6: TGTG. Libraries contained equimolar amplification products from three samples. The clonal control was untagged and dispatched in all libraries.

Sample	Tag	Library
① RUN0065	Tag0	in library 2
② RUN0067	Tag5	in library 11
③ RUN0109	Tag5	in library 1
④ RUN0281	Tag0	in library 1
⑤ RUN1041	Tag0	in library 3
⑥ RUN1050	Tag5	in library 3
⑦ RUN1132	Tag6	in library 1
⑧ RUN1232	Tag6	in library 2
⑨ RUN1578	Tag5	in library 2
① MU011I–#2	Tag0	in library 4
② MU025I–#1	Tag5	in library 4
③ MU040I–#3	Tag6	in library 4
④ ROD001	Tag0	in library 5
⑤ ROD200	Tag5	in library 5
① COM11_Mi2	Tag0	in library 7
② COM12_Mn1	Tag5	in library 7
③ COM18_Bi1	Tag0	in library 8
④ COM28_V2	Tag6	in library 7
⑤ COM59_A3.1	Tag5	in library 8
⑥ COM72_WaMi1	Tag6	in library 8
⑦ MAY35	Tag6	in library 3
① SEY059	Tag0	in library 6
② SEY141	Tag5	in library 6
③ SEY144	Tag6	in library 6
① ISM03	Tag6	in library 9
② ISM04	Tag6	in library 5
③ MAD26.1	Tag0	in library 9
④ MAD30.2	Tag5	in library 9
① GRE5.6	Tag6	in library 10
② CAR2.1	Tag5	in library 10
③ POR_06	Tag0	in library 10
Ⓒ Acer1.1	Tag0	in library 11
Ⓓ Ador	Tag6	in library 11
Control (Bla)	none	in all libraries
